# Pd‐Catalyzed C─C Bond Borylation of Biphenylenes Leading to Tri‐*Ortho*‐Substituted Biaryls

**DOI:** 10.1002/chem.202503515

**Published:** 2026-02-10

**Authors:** Robyn V. Presland, Konstantin V. Luzyanin, Luke A. Wilkinson, Oliver L. Jacobs, Nathan R. Halcovitch, Alexey G. Sergeev

**Affiliations:** ^1^ Department of Chemistry University of Liverpool Liverpool UK; ^2^ Department of Chemistry Lancaster University Lancaster UK

**Keywords:** biaryls, boronates, carbene ligands, C─C activation, palladium

## Abstract

Ring‐opening diborylation of carbon─carbon (C─C) single bonds is a powerful strategy for installing two versatile functional groups at nonadjacent carbon atoms, enabling skeletal editing of strained ring systems. However, such transformations remain rare for rings larger than cyclopropanes due to kinetic and thermodynamic challenges. Herein, we describe a palladium‐catalyzed diborylation of 1‐substituted biphenylenes enabled by a highly electron‐rich and sterically demanding N‐heterocyclic carbene (NHC) ligand. The reaction proceeds via selective cleavage of the least sterically hindered C─C bond and affords *ortho*‐diborylated biphenyls in 39%–89% isolated yields across a broad range of 1‐substituted biphenylenes with diverse steric and electronic properties. High regioselectivities (up to >20:1) are observed for cleavage of the least sterically hindered C─C bond. Regioselectivity is modulated by both electronic and steric effects: electron‐donating aryl substituents enhance selectivity, as indicated by a Hammett correlation, whereas spherical substituents favour higher selectivity than planar aryl groups. Supporting stoichiometric experiments indicate a pathway involving initial C─C bond activation. The resulting sterically hindered tri‐*ortho*‐substituted biaryls may serve as valuable synthetic intermediates, as demonstrated by selective sequential orthogonal postfunctionalization of a representative example.

## Graphical abstract

Palladium‐catalyzed ring‐opening C─C borylation of 1‐substituted biphenylenes yields a range of *o,o,o′*‐trisubstituted biaryls with two *ortho*‐boryl groups via selective cleavage of the least hindered C─C bond. The resulting diborylated products are readily amenable to sequential, site‐selective postfunctionalization.

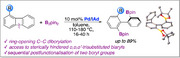

## 1 Introduction

**FIGURE 1:**
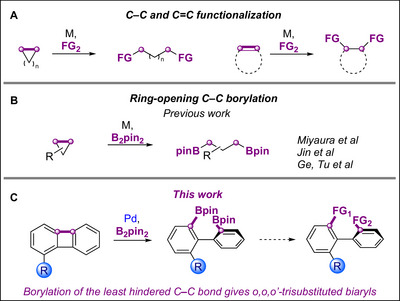
Catalytic ring‐opening borylation of single C─C bonds.

Ring‐opening functionalizations of C─C single bonds are an emerging and powerful approach for skeletal editing [1, 2, 3, 4, 5, 6]. Unlike conventional functionalizations of C─C double and triple bonds, which install functional groups at the vicinal carbons [7], ring‐opening C─C functionalizations enable the installation of two groups at more distant carbons (Figure 1A) [1]. Among these, C─C borylations are particularly useful because the resulting products can undergo a broad range of postfunctionalizations [8, 9, 10, 11, 12, 13, 14]. However, C─C borylations are far more challenging than C─H borylations [15, 16] due to less favorable kinetic and thermodynamic factors [1, 2, 3, 9]. To enhance the reactivity, substrates with strained rings are usually used.

Ring‐opening borylations of cyclopropanes are well established (Figure 1B) [17, 18, 19, 20, 21, 22, 23, 24, 25, 26, 27, 28, 29, 30, 31, 32, 33, 34, 35, 36], yet analogous transformations of four‐membered rings remain scarce [23, 37, 38], with only a single example of diborylation [39]. Extending this chemistry to substituted biphenylenes, especially those bearing substituents in the 1‐position, is particularly appealing as it could yield valuable *o,o,o′*‐substituted biaryls with two versatile boryl functionalities (Figure 1C). Such products might serve as valuable building blocks for the synthesis of a range of *ortho*‐substituted biaryl ligands [40], photoelectronic materials [41], and pharmaceuticals [42].

Despite this potential, most known catalytic C─C functionalizations of biphenylenes involve the unsubstituted parent molecule [38, 43, 44, 45, 46, 47, 48, 49, 50, 51, 52, 53, 54, 55, 56, 57, 58] with only a handful of reports on 1‐substituted derivatives [59, 60, 61], and just two focusing on nondirected C─C functionalizations that yield *o,o,o′*‐substituted biaryl frameworks [62, 63]. Realizing such transformations requires overcoming two key challenges: the inherently low reactivity of 1‐substituted biphenylenes and the need for regioselective cleavage of the least hindered C─C bond.

Herein, we report palladium‐catalyzed diborylation of the strained C─C bonds in 1‐substituted biphenylenes. The reaction occurs preferentially at the least sterically hindered C─C bond and leads to sterically hindered *o,o,o′*‐trisubstituted biaryls. The regioselectivity is strongly affected by the electronic and steric properties of the substituents in biphenylenes. We illustrated the utility of the borylation by selective sequential postfunctionalization of two boryl groups in the resulting products (Figure 1C).

## 2 Results and Discussion

For the initial optimization (Table 1), we examined borylation of 1‐fluorobiphenylene (**1a**), the substrate bearing the smallest 1‐substituent, with B_2_pin_2_ as the borylating agent [64]. We evaluated a range of soluble Pd, Ni, and Ir catalysts with ligands previously reported for C─C activations of biphenylene [65] and borylations of Csp^2^–X bonds [9, 15, 16]. Precatalysts were combined with phosphine (P*^n^* Bu_3_, DPPF, and JohnPhos), carbene (IMes), and phenanthroline (Phen) ligands (Table S1). The reactions were performed in toluene at 110°C with 10 mol% catalyst. Only borylation with Pd catalysts gave the desired product **2a** (Table 1, entries 1–5), with the highest yield obtained with IMes ligand (8%, entry 5). In contrast, Ni and Ir promoted dimerization (**5**) [66, 67, 68] and C─H borylation (**6**) [69], respectively (entries 6 and 7).

**TABLE 1:** Optimization of catalysts in the C─C diborylation of 1‐fluorobiphenylene^a^.

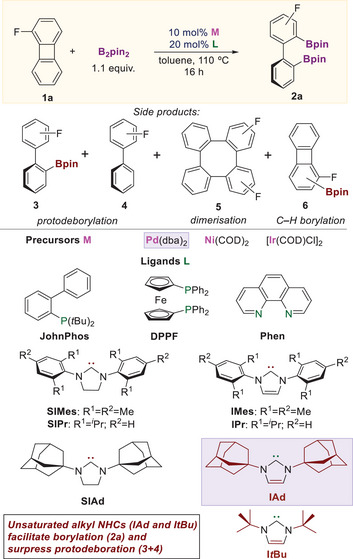								
Entry	Precursor	Ligand^b^	Conv.^c^		Yields, %^c^			
			of 1a, %	2a	3	4	3+4	5
1	Pd(dba)_2_	P(*n‐*Bu)_3_	5	1	1	<1	<2	0
2	Pd(dba)_2_	DPPF	4	0	<1	<1	<2	0
3	Pd(dba)_2_	JohnPhos	99	7	4	<1	<5	77
4	Pd(dba)_2_	Phen	4	2	0	<1	<1	0
5	Pd(dba)_2_	IMes	80	8	10	16	26	0
6	Ni(cod)_2_	IMes	99	<1	5	18	23	59
7	[Ir(cod)Cl]_2_	IMes	56^d^	0	10	20	30	0
8	Pd(dba)_2_	SIMes	61	0	16	20	36	0
9	Pd(dba)_2_	SIPr	99	<1	24	11	35	0
10	Pd(dba)_2_	IPr	85	2	23	8	31	0
11	Pd(dba)_2_	I*t*Bu	51	27	10	2	12	0
12	Pd(dba)_2_	SIAd	15	1	11	2	13	0
**13**	**Pd(dba)_2_**	**IAd**	**99**	**64**	**9**	**2**	**11**	**0**
**14** ^e^	**Pd(dba)_2_**	**IAd**	**100**	**86**	**8**	**2**	**10**	**0**

^a^ Reaction conditions: **1a** (0.1 mmol), B_2_pin_2_ (0.11 mmol), metal precursor (10 mol% M), ligand (20 mol% for monodentate and 10% for bidentate ligands), dodecane (GC internal standard, 0.05 mmol), toluene (1 mL), argon atmosphere, 110 °C, 16 h.

^b^ All carbene ligands were generated in situ from the corresponding HCl or HBF_4_ salts using *^t^* BuOK (22 mol%).

^c^ Conversions and yields were measured by GC.

^d^ **6** and C─H borylated solvent (toluene) are the main side products.

^e^ 2 equiv. (0.2 mmol) of B_2_pin_2_ was used.

**FIGURE 2:**
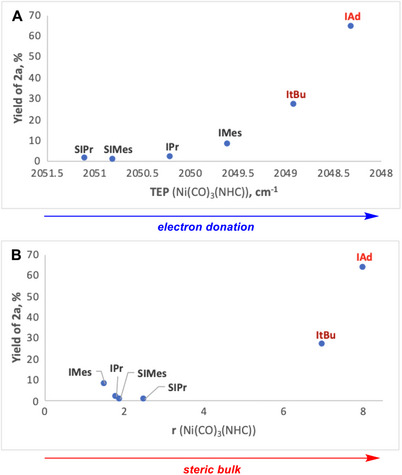
Dependence of the yields of **2a** on (A) TEP [73] and (B) Gusev's repulsiveness parameter (r) [75] of the tested NHC ligands.

Building on the Pd/IMes result (entry 5), we evaluated additional NHC ligands (entries 8–13). The N‐alkyl NHC carbene IAd gave the highest yield (65%, entry 13), which improved to 86% upon increasing the amount of B_2_pin_2_ from 1.1 equiv to 2 equiv (entry 14). In general, N‐alkyl NHCs (except SIAd) gave higher yields of **2a** than N‐aryl NHCs, and unsaturated carbenes (IMes, IPr, and IAd) gave higher yields than their saturated analogues (SIMes, SIPr and SIAd) [70, 71, 72]. Using N‐alkyl NHCs instead of N‐aryl NHCs also reduced the formation of biaryl side products **3** and **4**, likely resulting from protodeborylation of **2a** (entries 11–14). Higher yields of the C─C borylation with N‐alkyl NHCs (ItBu and IAd) correlate with their stronger electron donation and steric bulk. As shown in Figure 2, the product yield increases with decreasing Tolman electronic parameter (TEP) [73, 74] and increasing steric Gusev's repulsiveness parameter (r) [75, 76, 77, 78, 79, 80] of the NHCs [81]. These data suggest that IAd uniquely combines strong electron‐donating properties with very high steric bulk, which together promote efficient C─C borylation while suppressing competing side reactions.

Application of the optimized catalyst Pd(dba)_2_(10 mol %)/IAd (20 mol%) for the C─C borylation of a range of 1‐substituted biphenylenes with B_2_pin_2_ at 140°C afforded diborylbiphenyls in 39%–89% yields (Table 2). The borylation occurred preferentially at the least sterically hindered C─C bond yielding sterically hindered *o,o,o′*‐trisubstituted biphenyls as the major regioisomers. The scope encompassed 1‐substituted biphenylenes with methyl (**1d**), trimethylsilyl (**1p**), pinacolboryl (**1q**), and fluoro (**1a**) substituents, as well as aryl groups featuring neutral/alkyl (phenyl **1e**; α‐naphthyl **1f**; **1h**–**1j**, **1m**), donor (methoxy **1g**, **1k**; dimethylamino **1l**), or acceptor (*tert*‐butoxycarbonyl **1o**; trifluoromethyl **1n**) functionalities. A heteroaryl 3‐thienyl substituent (**1r**) was also tolerated, although the borylation gave a lower yield (43%) of the product compared to the phenyl (**1e**, 82%) partly due to the competing formation of phenanthro[9,10‐b]thiophene (10%) [82]. Unexpectedly, biphenylenes with usually inert methyl (**1d**) and trimethylsilyl (**1p**) substituents gave modest yields of the borylated products (48% and 39%, respectively). Substrates with directing groups such as 2‐pyridyl (**1s**) and diphenylphosphino (**1t**) failed to afford diborylated products even under forcing conditions (180°C, 40 h), likely due to strong heteroatom coordination. For the pyridine substrate **1s**, this resulted in catalyst deactivation and low conversion (25%). In contrast, the bulky phosphine substrate **1t** underwent full conversion but predominantly formed unidentified products. Similarly, 1‐chlorobiphenylene (**1b**) showed low conversion (22%) without diborylation, likely from catalyst decomposition, whereas the fluoro analogue (**1a**) gave the diborylated product in 64% isolated yield. Modest to low isolated yields for certain substrates arise primarily from competitive side processes involving the formation of unidentified side products (**1d**, **1p, 1r**, and **1t**).

**TABLE 2:** Palladium‐catalyzed borylation of 1‐substituted biphenylenes.

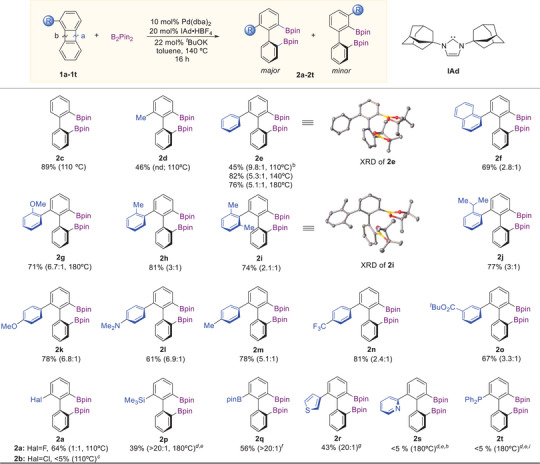

^a^ Reaction conditions: 1‐substituted biphenylene (0.4 mmol), B_2_pin_2_ (0.8 mmol), Pd(dba)_2_ (10 mol%, 0.04 mmol) IAd·HBF_4_ (20 mol%, 0.08 mmol), *^t^* BuOK (0.088 mmol), toluene (4 mL), 140°C, 16 h; Yields shown are isolated yields; the ratios in the parentheses are the ratios of major to minor isomers; all reactions were conducted to the full conversion of the corresponding biphenylenes unless noted otherwise.

^b^ Incomplete conversion: 22% of the starting **1d** was recovered.

^c^ Incomplete conversion (23%), chloroborylbiphenyls as main products and traces of 1‐Bpinbiphenylene.

^d^ 20 mol% Pd(dba)_2_, 40 mol% IAd·HBF_4_.

^e^ 40 h.

^f^ Diborylbiphenyl (13%) is a side product.

^g^ Phenanthro[9,10‐b]thiophene (10%) as a side product.

^h^ No reaction (75% of **1t** was recovered).

^i^ Full conversion, mixture of unidentified products.

The reactivity of biphenylenes generally decreased with increasing steric bulk of the 1‐substituent. Substrates with smaller substituents H (**1c**), F (**1a**), and Me (**1d**) reacted at 110°C within 16 h, whereas those with aryl substituents (**1e** and **1f**–**1o**) required a higher temperature of 140°C. This trend, however, was not uniform, and aryl substituents were clear outliers. Thus, increasing the steric bulk of aryl substituents by replacing phenyl (**1e**) or *para*‐substituted aryl groups (**1k**–**1n)** with *ortho*‐substituted aryls (**1g**–**1i**) did not significantly affect the reactivity or product yields (Table 2) [83]. In contrast, replacing planar aryl substituents (**1e**–**1o**) with the smaller but more spherically shaped trimethylsilyl group (**1p**) [84, 85, 86, 87, 88] reduced reactivity and required much harsher reaction conditions (180°C, 40 h vs. 140°C, 16 h). The ability to borylate even highly sterically congested substrates such as **1f–1j** with 69%–81% yields demonstrates excellent steric tolerance of the Pd/IAd system. This robustness enables access to *o,o,o′‐*trisubstituted biaryls that are otherwise challenging to prepare via conventional cross‐coupling routes.

The regioselectivity of borylation was highest at lower temperatures, as shown for 1‐phenylbiphenylene (**1e**): the major/minor isomer ratio was 9.8:1 at 110°C (conversion 78%); the ratio decreased to 5.3:1 at 140°C and levelled off at 5.1:1 at 180°C. Although the origin of this temperature‐dependent regioselectivity remains unclear, it may arise from a change in the identity of the active catalytic species and/or partial thermodynamic control (e.g., through pre‐equilibration of C─C activation intermediates). To balance high selectivity with full conversion within 16 h, reactions with most substrates were conducted at 140°C.

**FIGURE 3:**
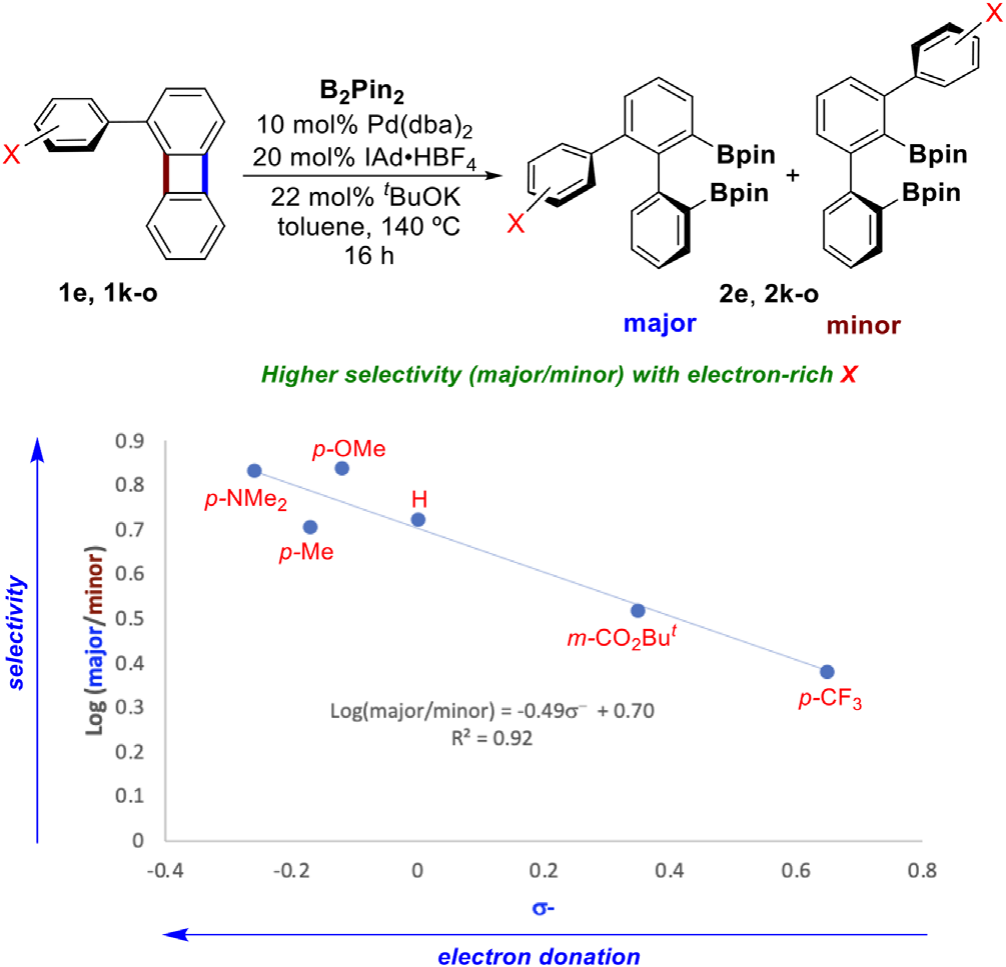
Correlation of the selectivity of the C─C borylation (major/minor) with the electronic properties of *para*‐ and *meta*‐substituents in 1‐arylbiphenylenes [96].

Beyond the temperature, regioselectivity was strongly influenced by the steric and electronic properties of the 1‐substituents (Table 2). For isotropic (spherical) substituents, selectivity predictably increased with the steric bulk from F (**1a**, 1:1) to SiMe_3_ (**1p**, 20:1), reflecting decreasing accessibility of the most hindered C─C bond. In sharp contrast, for anisotropic (planar) substituents, the selectivity decreased from phenyl (**1e**, 5.3:1) to bulkier groups such as α‐naphthyl (**1f**, 2.8:1), *o*‐methylphenyl (**1h**, 3:1), *o*‐isopropylphenyl (**1j**, 3:1), and *o,o*‐dimethylphenyl (**1i**, 2.1:1). We hypothesize that the counterintuitive reduced regioselectivity observed for these sterically demanding aryl substrates results from the interplay of opposing steric demands in different steps of the catalytic cycle (e.g., C─C activation and C─B bond‐forming reductive elimination). Electronic effects also played a major role: replacing *o*‐methylphenyl (**1h**) with the electron‐rich *o*‐methoxyphenyl (**1g**) gave a more than two‐fold increase in selectivity from 3:1 to 6.7:1. The role of electron donation is even more evident for the *para*‐substituted aryl groups: the selectivity increases from *p*‐CF_3_ (**1n**, 2.5:1) through *p*‐H (**1e**, 5.3:1) to *p*‐NMe_2_ (**1l**, 6.9:1) and *p*‐MeO (**1k**, 6.8:1). A good correlation was observed between the log(selectivity) and the σ^−^ Hammett constants (Figure 3) [89].

This Hammett correlation supports a mechanistic model in which the observed influence of electronic effects on selectivity indicates a greater propensity for the cleavage of more sterically hindered C─C bonds when the adjacent aryl substituent is more electron‐poor. This behavior can be rationalized by the increased preference of the Pd(0) center to coordinate to the more electron‐deficient aryl substituent [90, 91, 92], bringing the metal closer to the sterically hindered C─C bond and making its cleavage less unfavorable. Alternatively, this trend may be attributed to an increased propensity for metal‐mediated cleavage of the more electron‐poor and sterically hindered C─C bond. A similar effect was observed for insertion of Pt(0) complexes into the most electron‐poor and sterically hindered C─C bonds of 1,1,2,2‐tetracyanocyclopropane and 1,1,2,2‐tetracyanocyclobutanes. This can be rationalized by the weakening of the electron‐poor C─C bonds accompanied by the strengthening of the corresponding M─C bonds [93, 94].

**FIGURE 4:**
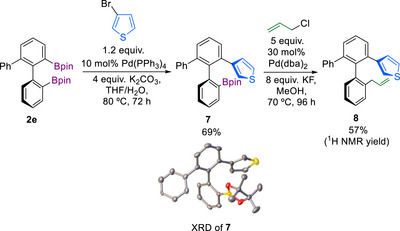
Catalytic postfunctionalization of diborylbiphenyl **2e**.

To exemplify the utility of the obtained products for further modification, we conducted difunctionalization of the three *ortho‐*substituted biaryl **2e** as a model compound (Figure 4). The two Bpin groups in **2e** differ in steric accessibility, making **2e** and its congeners an appealing target for sequential functionalization. Suzuki–Miyaura coupling of **2e** with 1.2 equiv. of 3‐bromothiophene led to selective arylation of the least sterically hindered Bpin group to yield **7** in 69% isolated yield. Subsequent Pd‐catalyzed allylation gave the difunctionalized product **8** in 57% yield [95].

**FIGURE 5:**
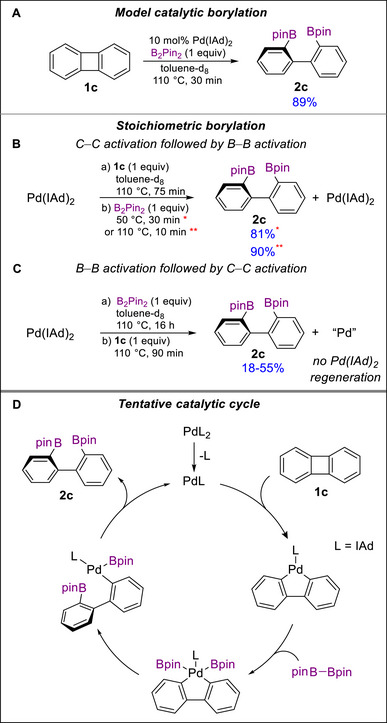
Results of catalytic and stoichiometric borylation experiments and the proposed catalytic cycle.

Our preliminary stoichiometric studies indicate that ring‐opening borylation proceeds via slower C─C cleavage followed by faster B─B cleavage (Figure 5). We probed this pathway using the well‐defined complex Pd(IAd)_2_ [97], which catalyzed the model borylation of biphenylene with B_2_pin_2_ (10 mol% Pd, toluene, 110°C, 16 h) to give **2c** in 89% yield (Figure 5A), identical to that obtained with the standard precatalyst Pd(dba)_2_/IAd·HBF_4_/*^t^* BuOK (Table 2). The stoichiometric reaction of Pd(IAd)_2_ with biphenylene (1 equiv) in toluene‐d_8_ at 110°C was complete within 75 min (Figure 5B). Subsequent treatment of the resulting species [98] with B_2_pin_2_ (1 equiv) at 50°C for 30 min regenerated Pd(IAd)_2_, and gave **2c** in 81% yield. Conducting the reaction with B_2_pin_2_ at an elevated temperature of 110°C for 10 min increased the yield of **2c** to 90%, comparable to that in the catalytic reaction (89%). In contrast, reversing the order of addition led to a much slower reaction (Figure 5C): Pd(IAd)_2_ reacted with 1 equiv B_2_pin_2_ at 110°C for 16 h with noticeable decomposition of the starting complex [99, 100, 101, 102]. Subsequent reaction with biphenylene at 110°C for 1.5 h gave lower and variable yields of **2c** (18%–55%) with no Pd(IAd)_2_ regeneration. These results suggest that the pathway involving initial B─B activation is less competitive than that involving initial C─C activation (Figure 5D).

Although these preliminary results suggest that C─C activation may be a rate‐determining step, this interpretation likely represents an oversimplification and does not fully account for the observed trends, including the counterintuitive effects of sterics on regioselectivity. More detailed kinetic studies under catalytic conditions, complemented by computational investigations, will be required to elucidate the full catalytic cycle, identify the turnover‐limiting step, and rationalize the origin of the observed selectivity, which may result from contributions of multiple elementary steps.

## 3 Conclusion

We report a catalytic strategy for ring‐opening borylation of C─C single bonds in four‐membered systems, providing efficient access to sterically congested *o,o,o*′‐trisubstituted biaryls that are otherwise challenging to obtain. An electron‐rich, sterically demanding Pd(0)/NHC catalyst system is essential for high reactivity and broad scope, enabling selective cleavage of the least hindered C─C bond while suppressing C─H and C─F borylation and reductive pathways. Regioselectivity is dictated by the steric and electronic properties of the 1‐substituent: electron‐rich aryl groups increase selectivity, whereas enhanced steric bulk in anisotropic aryl substituents counterintuitively reduces it; in contrast, selectivity rises with the size of isotropic substituents. The diborylated products may serve as versatile intermediates, as demonstrated by the selective sequential functionalisation of the two Bpin groups in a representative example. Further work will focus on elucidating the identity of the reactive intermediates, rationalizing the observed reactivity and regioselectivity, and designing more selective and active catalytic systems.

## Conflicts of Interest

The authors declare no conflict of interest.

## Supporting information




**Supporting File 1**: Experimental procedures, characterization data, NMR spectra for new compounds, crystallographic data for **2e** (CIF), **2g** (CIF), **2i** (CIF) and **7** (CIF). Deposition Number(s) 2496927 (for **2e**), 2496928 (for **2g**), 2496929 (for **2i**), 2496926 (for **7**) contain(s) the supplementary crystallographic data for this paper. These data are provided free of charge by the joint Cambridge Crystallographic Data Centre and Fachinformationszentrum Karlsruhe Access Structures service. Additional references cited within the Supporting Information [103, 104, 105, 106, 107, 108, 109, 110, 111, 112, 113, 114, 115, 116, 117, 118].

## Data Availability

The data that support the findings are available in the supplementary material of this article.
