# Supplementary material for: Pd‐Catalyzed C─C Bond Borylation of Biphenylenes Leading to Tri‐Ortho‐Substituted Biaryls
Source: Chemistry. 2026 Feb 10;32(16):e03515. doi: 10.1002/chem.202503515 (PMC13109692; doi:10.1002/chem.202503515)
Supplement: Supplementary file 1 — Supporting File 1: Experimental procedures, characterization data, NMR spectra for new compounds, crystallographic data for 2e (CIF), 2g (CIF), 2i (CIF) and 7 (CIF). Deposition Number(s) 2496927 (for 2e), 2496928 (for 2g), 2496929 (for 2i), 2496926 (for 7) contain(s) the supplementary crystallographic data for this paper. These data are provided free of charge by the joint Cambridge Crystallographic Data Centre and Fachinformationszentrum Karlsruhe Access Structures service. Additional references cited within the Supporting Information [103, 104, 105, 106, 107, 108, 109, 110, 111, 112, 113, 114, 115, 116, 117, 118]. [file CHEM-32-e03515-s001.pdf]

Pd-Catalyzed C–C Bond Borylation of Biphenylenes Leading to Tri-*ortho*-Substituted Biaryls

Robyn V. Presland,[1] Konstantin V. Luzyanin,[1] Luke A. Wilkinson,[1] Oliver L. Jacobs,[1] Nathan R. Halcovitch[2] and Alexey G. Sergeev[1]*

[1]Department of Chemistry, University of Liverpool, Crown Street, Liverpool L69 7ZD, United Kingdom
[2]Department of Chemistry, Lancaster University, Lancaster, 2LA1 4YB, United Kingdom

Table of Contents

1. General experimental details S2
2. Synthesis of 1-substituted biphenylenes S3
   Figure S1 S3
   Figure S2 S8
   Figure S3 S10
3. Screening conditions for the ring opening diborylation of 1-fluorobiphenylene S18
   Table S1 S18
   Figure S4 S20
   Figure S5 S20
   Figure S6 S21
4. Scope of the palladium-catalyzed C–C diborylation of biphenylenes with B_2pin_2 S22
   Figure S7 S43
5. Variation of diboranes in the C–C diborylation of 1-phenylbiphenylene (**1e**). S45
   Table S2 S46
6. Postfunctionalization of diborylation product **2e** S48
7. Stoichiometric experiments on borylation of biphenylene S53
   Table S3 S53
   Table S4 S54
   Table S5 S54
   Table S6 S56
   Table S7 S57
   Table S8 S59
   Figure S8 S60
8. NMR spectra S61
9. References S113

1. General experimental details

All air sensitive reactions were conducted under an atmosphere of argon using standard Schlenk techniques or in an Innovative Technologies glovebox. Argon (Pureshield, >99.998%) was purchased from BOC and used as received. All glassware was heated to 120 °C in an oven and cooled under vacuum prior to use. Unless otherwise noted the stated reaction temperature is the temperature of the oil bath.

Chemicals and reagents. Unless otherwise stated, all chemicals were obtained from commercial suppliers and used without further purification. Pd_2dba_3, $Pd(PPh_3)_4$, (3-bromo-2-fluorophenyl) boronic acid, 2-bromoiodobenzene, and XPhos were obtained from Fluorochem. 1-fluorobiphenylene (**1a**) and 1-chlorobiphenylene (**1b**) were prepared according to the literature procedure[103, 104]. $Pd(IAd)_2$ was prepared from $[(\eta^3\text{-}C_3H_5)PdCl]_2$ and sodium diethylmalonate.[97]

Solvents. Unless otherwise stated all solvents were dried over the appropriate drying agent and distilled under argon. Dioxane and THF were dried with sodium using benzophenone as an indicator and stored over activated 4 Å molecular sieves. Toluene and *n*-hexane were dried with calcium hydride. *m*-Xylene was purchased as 'extra dry' from Acros Organics and used as received. *n*-Dodecane and deuterated toluene were degassed by freeze-pump-thaw technique (3 cycles) and stored over molecular sieves. Anhydrous $CDCl_3$ was purchased from Merck and used as received; C_6D_6 was purchased from Apollo Scientific and distilled over calcium hydride.

Nuclear magnetic resonance (NMR) spectroscopy. NMR spectra were recorded on Bruker AVIII HD 500 MHz, Bruker AVI 400 MHz, and Bruker AVIII HD 400 MHz spectrometers at ambient temperature. Chemical shifts are reported in ppm relative to a residual solvent peak ($CDCl_3$, δ 7.26 ppm for ^1H and 77.16 ppm for ^{13}C); 0.0 ppm chemical shifts of $CFCl_3$ and H_3PO_4 were used for referencing of ^{19}F and ^{31}P, respectively . The splitting patterns are reported as follows s (singlet), d (doublet), t (triplet), q (quartet), spt (septet), m (multiplet), dd (doublet of doublets), dt (doublet of triplets), br. s (broad singlet). The coupling constants *J* are given in Hertz.

High-resolution mass spectrometry (HRMS). HRMS were recorded using the analytical service in the Chemistry Department at the University of Liverpool, using an Agilent QTOF 7200 or (chemical ionization, CI) or an Agilent QTOF 6540 (electrospray ionisation, ESI).

Gas chromatography (GC). GC analyses of the Suzuki–Miyaura cross-coupling of 1-chlorobiphenylene were conducted on an Agilent 7890 Gas chromatograph fitted with a HP-5 column (length: 30 m, ID: 0.32 mm, FT: 0.25 μm) and an FID detector. Helium was used as the carrier gas. The following GC oven temperature programme was used: 80 °C hold for 5 min, ramp 40 °C/min to a final temperature of 150 °C and hold for 5 min, ramp 15 °C/min to a final temperature of 250 °C and hold for 10 min.

Gas chromatography-mass spectrometry (GC-MS). GC-MS analyses were conducted on a Thermo Scientific ISQ single quadrupole instrument equipped with a HP-1 column (length: 30m, ID: 0.32 mm,

FT: 0.25 µm). Helium was used as the carrier gas. EI was used as the ionisation mode. The following GC oven temperature programme was used: 80 °C hold for 2 min, ramp 20 °C/min to a final temperature of 250 °C and hold for 7 min.

Chromatography. Analytical thin-layer chromatography (TLC) was performed on Merck F254 TLC silica gel 60 TLC plates, visualised with UV light, 254 nm. Column chromatography was performed using VWR silica gel 40-63 µm.

X-ray crystalography. Single crystals were selected and mounted on a Mitegen loop using Paratone-N oil on a SuperNova, Dual, Cu at home/near, AtlasS2 diffractometer. The crystal was kept at 100(1) K during data collection. Using Olex2,[105] the structures were solved with the SHELXT[106] structure solution program using Intrinsic Phasing and refined with the SHELXL refinement package using Least Squares minimisation. Data are available free of charge from the CCDC (https://www.ccdc.cam.ac.uk/) the deposition numbers are: 2496926 (**7**), 2496927 (**2e**), 2496928 (**2g**) and 2496929 (**2i**).

2. Synthesis of 1-substituted biphenylenes

2.1 Preparation of 1-monosubstituted biphenylenes via aryne cyclization: lithiation of 2',3-dibromo-2-fluoro-1,1'biphenyl followed by quenching with electrophiles

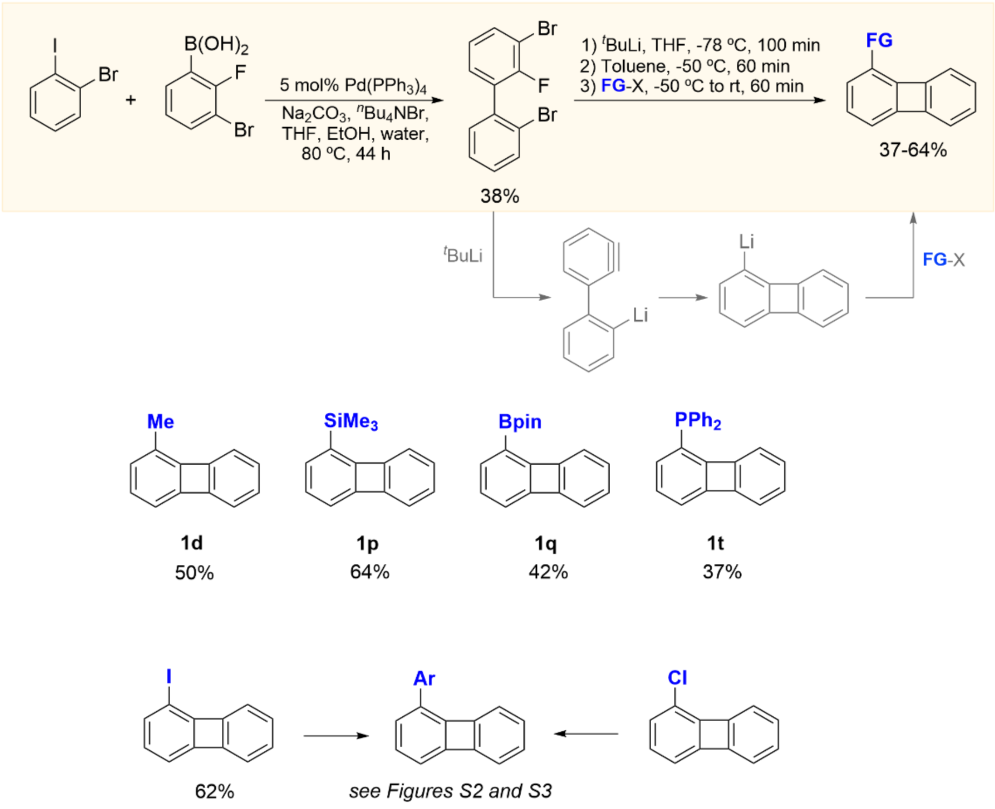

Figure S1. Preparation of 1-monosubstituted biphenylenes via i*n situ* generated 1-biphenylenyl lithium.

2’,3-dibromo-2-fluoro-1,1’biphenyl

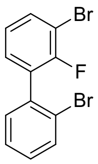

A three-neck round bottom flask equipped with a condenser, dropping funnel and a magnetic stir bar was evacuated, filled with argon and charged with $Pd(PPh_3)_4$ (0.520 g, 0.450 mmol), Na_2CO_3 (2.38 g, 22.48 mmol), $^nBu_4NBr$ (2.90 g, 9.00 mmol), (3-bromo-2-fluorophenyl) boronic acid (2.36 g, 10.78 mmol), and 2-bromoiodobenzene (2.58 g, 9.11 mmol). A mixture of THF (30 mL), EtOH (15 mL) and water (15 mL) was degassed by sparging with argon for 15 minutes and then added to the reaction flask. The mixture was stirred to give a yellow suspension. The reaction mixture was stirred (600 rpm) at 80 °C for 44 h and turned beige during this time. After cooling to room temperature, the organic solvents was removed under reduced pressure. The resulting residue was diluted with distilled water (60 mL) and the resulting mixture was extracted with EtOAc (3x60 mL). The combined organic layers were washed with water (2 x 60 mL), dried over $MgSO_4$, filtered, and the solvent was removed under reduced pressure. The crude product was purified by flash column chromatography (silica gel, hexane).

Yield of 2’,3-dibromo-2-fluoro-1,1’biphenyl: 38% (1.13 g, 3.42 mmol); a colourless oil. The reaction conditions/yield were not optimized.

2’,3-dibromo-2-fluoro-1,1’biphenyl

^{1}H NMR (500 MHz, $CDCl_3$) δ 7.69 (d, *J* = 8.0 Hz, 1H, CH), 7.60 (t, *J* = 7.2 Hz, 1H, CH), 7.38 (t, *J* = 7.5 Hz, 1H, CH), 7.30 – 7.21 (m, 3H, CH), 7.1 (t, *J* = 7.8 Hz, 1H, CH).

^{19}F{^{1}H} NMR (377 MHz, $CDCl_3$) δ - 107.26.

^{13}C{^{1}H} NMR (126 MHz, $CDCl_3$) δ 156.0 (d, *J* = 248.3 Hz, C), 136.3 (C), 133.5 (CH), 133.0 (CH), 131.6 (CH), 130.8 (d, *J* = 2.5 Hz, CH), 130.3 (d, *J* = 17.2 Hz, C), 130.0 (CH), 127.4 (CH), 124.8 (d, *J* = 4.6 Hz, CH), 123.7 (C), 109.6 (d, *J* = 21.7, C).

HRMS (CI+) m/z: calculated for $[C_{12}H_7Br_2F+H]^+$ 328.8977, found 328.8972.

Note: a mixture of the starting 2-bromoiodobenzene (41%) and the product of protodeborylation, 2-fluorobromobenzene (11% relative to the starting (3-bromo-2-fluorophenyl) boronic acid) were also isolated (a colourless oil, 1.27 g).

1-Iodobiphenylene

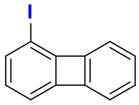

A Schlenk flask equipped with a septum and a magnetic stir bar and was evacuated, filled with argon and charged with 2’,3-dibromo-2-fluoro-1,1’-biphenyl (2.10 g, 6.36 mmol) and THF (32 mL). The resulting solution was cooled to -78 °C and a 1.7 M solution of *t*-BuLi in pentane (15.0 mL, 25.4 mmol) was added dropwise over a period of 40 min to give a yellow reaction mixture. The mixture was stirred

(500 rpm) at -78 ºC for 1 h. Toluene (95 mL) was then added slowly over 25 min and the reaction mixture was allowed to warm to -30 ºC and stir (500 rpm) for an additional 1 h. Iodine (1.94 g, 7.64 mmol) was added and then the reaction mixture was allowed to warm to room temperature and stired (500 rpm) for an additional 1 h. An aqueous solution of sodium thiosulfate (60 mL, 1M) was added and the solution turned pale yellow. The two phases were separated and the aqueous phase was extracted with EtOAc (2 x 60 mL). The combined organic layers were washed with water (2 x 60 mL), dried over $MgSO_4$, filtered, and the solvent was removed under reduced pressure. The crude product was purified by flash column chromatography (silica gel, hexane).

Yield of 1-iodobiphenylene: 62% (1.10 g, 3.96 mmol); pale-yellow oil.

^{1}H NMR (500 MHz, $CDCl_3$) δ 6.95 (d, *J* = 8.6 Hz, 1H, CH), 6.84 – 6.73 (m, 3H, CH), 6.67 – 6.65 (m, 1H, CH), 6.58 (d, *J* = 6.7 Hz, 1H, CH), 6.45 (dd, *J* = 9.1, 6.8 Hz, 1H, CH).

^{13}C{^{1}H} NMR (126 MHz, CDCl3) δ 156.8 (C), 152.9 (C), 151.2 (C), 149.7 (C), 136.3 (CH), 129.9 (CH), 129.3 (CH), 128.8 (CH), 117.9 (CH), 116.6 (2CH), 80.8 (C).

HRMS (CI+) m/z calculated for $(C_{12}H_7I+H)^+$ 278.9671, found 278.9661. The ^{1}H NMR data were consistent with that reported in the literature.[107]

1-Methylbiphenylene (1d)

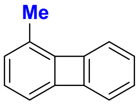

A Schlenk flask equipped with a septum and a magnetic stir bar was evacuated, filled with argon and charged with 2’,3-dibromo-2-fluoro-1,1’-biphenyl (495 mg, 1.50 mmol) and THF (7.5 mL). The resulting solution was cooled to -78 °C and a 1.7 M solution of *t*-BuLi in pentane (3.5 mL, 6.0 mmol) was added dropwise to give a yellow solution. The solution was stirred (600 rpm) at -78 ºC for 1 h and then toluene (22.5 mL) was slowly added over a period of 10 min. The reaction mixture was warmed to -30 ºC and stirred (600 rpm) at that temperature for 1 h, becoming dark green. Iodomethane (187 µL, 3.00 mmol) was then added dropwise at -30 ºC. After warming to room temperature, the mixture was stirred (600 rpm) for 1 h, and distilled water (15 mL) was then added. The two phases were separated and the aqueous phase was extracted with EtOAc (2 x 15 mL). The combined organic layers were washed with water (2 x 15 mL), dried over $MgSO_4$, filtered, and the solvent was removed under reduced pressure. The crude product was purified by flash column chromatography (silica gel, hexane).

Yield 1d: 50% (126 mg, 0.758 mmol); yellow oil.

^{1}H NMR (400 MHz, $CDCl_3$) δ 6.75 – 6.59 (m, 5H, CH), 6.54 (d, *J* = 8.3 Hz, 1H, CH), 6.48 (d, *J* = 6.7 Hz, 1H, CH), 2.1 (s, 3H, CH_3). The ^{1}H NMR data were consistent with that reported in the literature.[108]

1-(Trimethylsilyl)biphenylene (1p)

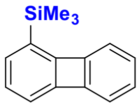

A Schlenk flask was evacuated, filled with argon, and charged with 2',3-dibromo-2-fluoro-1,1'-biphenyl (331 mg, 1.00 mmol) and THF (5 mL). The resulting solution was cooled to -78 °C and a 1.7 M solution of *t*-BuLi in pentane (2.4 mL, 4.0 mmol) was added dropwise to give a yellow reaction mixture. The mixture was stirred (600 rpm) at -78 ºC for 1 h and toluene (15 mL) was then slowly added at -78 ºC over a period of 5 min. The reaction mixture was warmed to -30 ºC and stirred (600 rpm) at that temperature for 1 h turning dark green. TMS-Cl (254 µL, 2.00 mmol) was then added dropwise (no colour change was observed). After warming to room temperature, the reaction mixture was stirred (600 rpm) for 1 h and then quenched with water (10 mL). The two phases were separated and the aqueous phase was extracted with EtOAc (2 x 10 mL). The combined organic layers were washed with water (2 x 10 mL), dried over $MgSO_4$, filtered, and the solvent was removed under reduced pressure. The crude product was purified by flash column chromatography (silica gel, hexane).

Yield of 1p: 64% (145 mg, 0.646 mmol); yellow oil.

^{1}H NMR (400 MHz, $CDCl_3$) δ 6.82 (d, *J* = 8.0 Hz, 1H, CH), 6.74 - 6.69 (m, 3H, CH), 6.66 - 6.60 (m, 3H, CH), 0.26 (s, 9H, CH_3). The ^{1}H NMR spectrum is consistent with that reported in the literature.[109]

1-(4,4,5,5-tetramethyl-1,3,2-dioxaborolan-2-yl)biphenylene (1q)

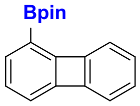

A Schlenk flask equipped with a septum and a magnetic stir bar was charged with 2',3-dibromo-2-fluoro-1,1'-biphenyl (990 mg, 3.00 mmol) and THF (15 mL). The resulting solution was cooled to -78 °C and a 1.7 M solution of *t*-BuLi in pentane (7.0 mL, 12 mmol) was added dropwise to give a yellow reaction mixture. The reaction mixture was stirred (600 rpm) at -78 ºC for 1 h and toluene (45 mL) was slowly added at -78 ºC over a period of 15 min. The reaction mixture was warmed to -30 ºC and stirred (600 rpm) at that temperature for 1 h. At -30 ºC isopropoxyboronic acid pinacol ester (1.3 mL, 6.4 mmol) was added dropwise to give a brown reaction mixture. After warming to room temperature, the reaction mixture was stirred (600 rpm) for 1 h and quenched with water (30 mL). The two phases were separated and the aqueous phase was extracted with EtOAc (2 x 30 mL). The combined organic layers were washed with water (2 x 30 mL), dried over $MgSO_4$, filtered, and the solvent was removed under reduced pressure. The crude product was purified by flash column chromatography (silica gel, 4% EtOAc in hexane).

Yield of 1q: 42% (276 mg, 0.992 mmol); yellow oil.

^{1}H NMR (500 MHz, $CDCl_3$) δ 7.05 (d, *J* = 8.1 Hz, 1H, CH), 6.85 (d, *J* = 6.2 Hz, 1H, CH), 6.79 – 6.70 (m, 3H, CH), 6.66 (d, *J* = 6.5 Hz, 1H, CH), 6.63 (d, *J* = 6.3 Hz, 1H, CH).

$^{13}C\{^1H\}$ **NMR** (126 MHz, $CDCl_3$) δ 159.9 (C), 152.6 (C), 152.0 (C), 151.0 (C), 132.7 (CH), 128.7 (CH), 128.5 (CH), 127.5 (CH), 119.1 (CH), 119.0 (CH), 117.5 (CH), 83.8 (2C), 25.1 (CH_3). One quaternary carbon signal is not observed.

HRMS (CI+) m/z: calculated for $(C_{18}H_{19}BO_2+H)^+$ 279.1556, found 279.1553.

1-Diphenylphosphinebiphenylene (1t)

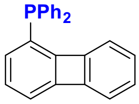

A Schlenk flask equipped with a septum and a magnetic stir bar was charged with 2’,3-dibromo-2-fluoro-1,1’-biphenyl (497 mg, 1.51 mmol) and THF (7.5 mL). The resulting solution was cooled to -78 °C and a 1.7 M solution of *t*-BuLi in pentane (3.5 mL, 6.0 mmol) was added dropwise over a period of 10 min to give a yellow reaction mixture. The reaction mixture was stirred (600 rpm) at -78 ºC for 1 h and toluene (22.5 mL) was slowly added at -78 ºC over a period of 10 min. The reaction mixture was warmed to -30 ºC and stirred (600 rpm) at that temperature for 1 h. At -30 ºC diphenylchlorophosphine (0.55 mL, 3.0 mmol) was added dropwise to give a dark brown reaction mixture. After warming to room temperature, the reaction mixture was stirred (600 rpm) for 1 h and quenched with water (15 mL). The two phases were separated and the aqueous phase was extracted with EtOAc (2 x 15 mL). The combined organic layers were washed with water (2 x 15 mL), dried over $MgSO_4$, filtered, and the solvent was removed under reduced pressure. The crude product was purified by flash column chromatography (silica gel, 1% EtOAc in hexane) followed by recrystallization in EtOH.

Yield of 1t: 37% (186 mg, 0.553 mmol); yellow solid.

^1H **NMR** (400 MHz, $CDCl_3$) δ 7.51- 7.46 (m, 4H, CH), 7.38 – 7.36 (m, 6H, CH), 6.72 – 6.57 (m, 5H, CH), 6.50 (t, J = 5.0 Hz, 1H, CH), 5.32 (d, J = 6.9 Hz, 1H, CH).

$^{13}C\{^1H\}$ **NMR** (100 MHz, $CDCl_3$) δ 155.7 (d, J = 12.0 Hz, C), 151.7 (C), 151.65 (C), 151.6 (C), 151.0 (C), 136.3 (d, J = 10.2 Hz, 2C), 133.9 (d, J = 19.9 Hz, 4CH), 132.5 (d, J = 20.7 Hz, CH), 129.0 (2CH), 128.8 (d, J = 7.2 Hz, 4CH), 128.5 (2CH), 128.3 (CH), 118.9 (CH), 117.2 (CH), 116.9 (CH).

$^{31}P\{^1H\}$ **NMR** (162 MHz $CDCl_3$) δ -12.0.

HRMS (CI+) m/z: calculated for $(C_{24}H_{17}P+H)^+$ 337.1146, found 337.1154

2.2 Synthesis of 1-(hetero)arylbiphenylenes

2.2.1 Preparation of 1-arylbiphenylenes by Suzuki coupling of 1-chlorobiphenylene and arylboronic acids

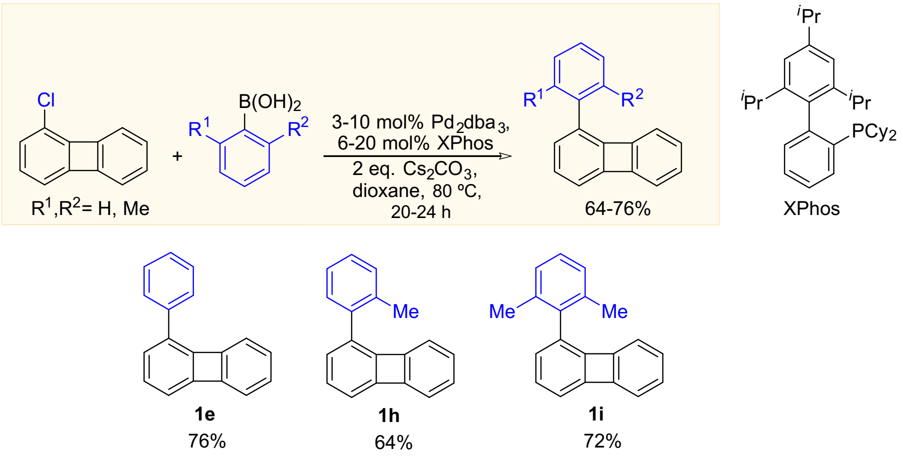

Figure S2. Preparation of 1-arylbiphenylenes from 1-chlorobiphenylene.

General procedure

A Schlenk bomb was charged with $Pd_2(dba)_3$ (3-10 mol%), XPhos (6-20 mol%), Cs_2CO_3 (2 eq.), and arylboronic acid (1.5 eq.), a magnetic stir bar, then evacuated and filled with argon. A solution of 1-chlorobiphenylene (1 eq.) in dioxane (0.33 M) was added. The Schlenk bomb was sealed and the reaction mixture was stirred (600 rpm) at 80 °C for the stated time. After cooling to room temperature, the reaction mixture was filtered through a plug of celite and the plug was washed with EtOAc until no compound was visible by UV on a TLC plate. The solvent was removed under reduced pressure to give the crude product. The crude product was purified by flash column chromatography.

1-Phenylbiphenylene (1e)

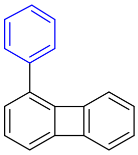

The reaction was conducted according to the general procedure using $Pd_2(dba)_3$ (35.5 mg, 39 μmol), XPhos (37 mg, 78 μmol), Cs_2CO_3 (0.85 g, 2.6 mmol), phenylboronic acid (238 mg, 1.95 mmol), 1-chlorobiphenylene (241 mg, 1.3 mmol), and dioxane (3.9 mL). The reaction mixture was stirred (600 rpm) at 80 °C for 20 h. The crude product was purified by flash column chromatography (silica gel, 0-5% EtOAc in hexane) to give **1e** as a yellow oil (224 mg, 0.98 mmol) in 76% yield.

^{1}H NMR (400 MHz, $CDCl_3$) δ 7.60 (d, J = 7.48 Hz, 2H, CH), 7.45 (t, J = 7.52 Hz, 2H, CH), 7.35 (t, J = 7.24 Hz, 1H, CH), 7.00 (d, J = 8.44 Hz, 1H, CH), 6.87 – 6.84 (m, 1H, CH), 6.79 – 6.76 (m, 3H, CH),

6.70 – 6.66 (m, 1H, CH), 6.62 (d, *J* = 6.68 Hz, 1H, CH). The ^{1}H NMR spectrum is consistent with that reported in the literature.[109]

1-(2-Methylphenyl)biphenylene (1h)

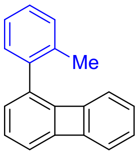

The reaction was conducted according to the general procedure using $Pd_2(dba)_3$ (41.2 mg, 45 µmol), XPhos (42.5 mg, 90 µmol), Cs_2CO_3 (0.39 g, 1.2 mmol), 2-methylphenylboronic acid (122 mg, 0.9 mmol), 1-chlorobiphenylene (112 mg, 0.6 mmol), and dioxane (1.8 mL). The reaction mixture was stirred (600 rpm) at 80 °C for 24 h. The crude product was purified by flash column chromatography (silica gel, hexane).

Yield of 1h: 64% (93 mg, 0.38 mmol); yellow solid.

^{1}H NMR (500 MHz, $CDCl_3$) δ 7.29 – 7.24 (m, 4H, CH), 6.81 (t, *J* = 7.48 Hz, 1H, CH), 6.74 – 6.61 (m, 5H, CH), 6.39 (d, *J* = 6.60 Hz, 1H, CH), 2.37 (s, 3H, CH_3).

$^{13}C\{^1H\}$ NMR (126 MHz, $CDCl_3$) δ 151.7 (C), 151.4 (C), 151.1 (C), 149.4 (C), 137.8 (C), 135.7 (C), 132.0 (C), 130.6 (CH), 130.0 (CH), 129.1 (CH), 128.6 (CH), 128.3 (CH), 128.3 (CH), 127.8 (CH), 126.0 (CH), 117.9 (CH), 117.3 (CH), 116.1 (CH), 20.0 (CH_3).

HRMS (CI^+) m/z: calculated for $(C_{19}H_{14}+H)^+$ 243.1174, found 243.1178.

1-(2,6-Dimethylphenyl)biphenylene (1i)

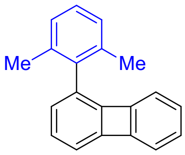

The reaction was conducted according to the general procedure using $Pd_2(dba)_3$ (54.8 mg, 60 µmol), XPhos (56.3 mg, 120 µmol), Cs_2CO_3 (0.39 g, 1.2 mmol), 2,6-dimethylphenylboronic acid (135 mg, 0.9 mmol), 1-chlorobiphenylene (113 mg, 0.6 mmol), and dioxane (1.8 mL). Reaction mixture was stirred (600 rpm) at 80 °C for 24 h. The crude product was purified by flash column chromatography (silica gel, hexane).

Yield of 1i : 72% (112 mg, 0.44 mmol); yellow oil.

^{1}H NMR (500 MHz, $CDCl_3$) δ 7.17 – 7.09 (m, 3H, CH), 6.82 – 6.78 (m, 1H, CH), 6.74 – 6.63 (m, 4H, CH), 6.56 (d, *J* = 6.70 Hz, 1H, CH), 6.24 (d, *J* = 6.65 Hz, 1H, CH), 2.21 (s, 6H, CH_3).

$^{13}C\{^1H\}$ NMR (126 MHz, $CDCl_3$) δ 151.7 (C), 151.5 (C), 151.3 (C), 149.6 (C), 137.3 (C), 136.2 (2C), 130.8 (C), 130.3 (CH), 128.6 (CH), 128.4 (CH), 128.3 (CH), 127.5 (2CH), 127.4 (CH), 117.5 (CH), 117.4 (CH), 116.1 (CH), 20.6 ($2CH_3$).

HRMS (CI^+) m/z: calculated for $(C_{20}H_{16}+H)^+$ 257.1330, found 257.1332.

2.2.2 Preparation of 1-(hetero)arylbiphenylenes by Suzuki coupling of 1-iodobiphenylene and arylboronic acids

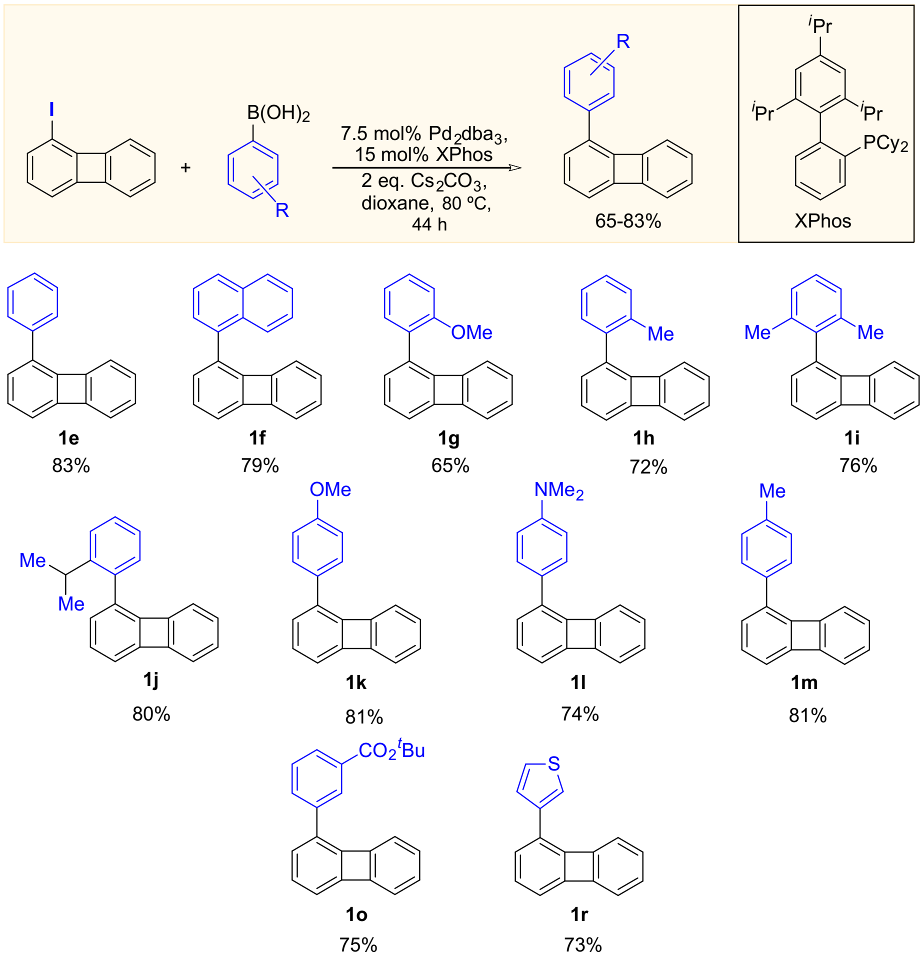

Figure S3. Preparation of 1-arylbiphenylenes from 1-chlorobiphenylene.

General procedure

A Schlenk bomb was charged with $Pd_2(dba)_3$ (7.5 mol%), XPhos (15 mol%), Cs_2CO_3 (1.5 – 2 eq.), arylboronic acid (1.5 eq.), a magnetic stir bar, and then evacuated and backfilled with argon. A solution of 1-iodobiphenylene (1 eq.) in dioxane was then added. The Schlenk bomb was sealed and the reaction mixture stirred (600 rpm) at 80 °C for 24 h. After cooling to room temperature, the reaction mixture was filtered through a plug of celite and the plug was washed with EtOAc (~200 mL) until no compound was visible by UV on a TLC plate. The solvent was removed under reduced pressure to give the crude product. The crude product was purified by flash column chromatography.

1-Phenylbiphenylene (1e)

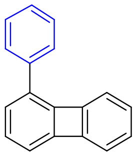

The reaction was conducted according to the general procedure, using $Pd_2(dba)_3$ (42 mg, 0.045 mmol), XPhos (43 mg, 0.091 mmol), Cs_2CO_3 (391 mg, 1.20 mmol), phenylboronic acid (110 mg, 0.905 mmol), 1-iodobiphenylene (167 mg, 0.600 mmol), and dioxane (1.4 mL). The crude product was purified by flash column chromatography (silica gel, hexane).

Yield of 1e: 83% (114 mg, 0.499 mmol); pale-yellow oil

^{1}H NMR (400 MHz, $CDCl_3$) δ 7.60 (d, *J* = 7.5 Hz, 2H, CH), 7.45 (t, *J* = 7.5 Hz, 2H, CH), 7.35 (t, *J* = 7.2 Hz, 1H, CH), 7.00 (d, *J* = 8.4 Hz, 1H, CH), 6.87 – 6.84 (m, 1H, CH), 6.79 – 6.76 (m, 3H, CH), 6.70 – 6.66 (m, 1H, CH), 6.62 (d, *J* = 6.7 Hz, 1H, CH). The ^{1}H NMR spectrum is consistent with that reported in the literature.[109]

1-(Naphthalen-1-yl)biphenylene (1f)

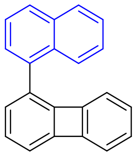

The reaction was conducted according to the general procedure, using $Pd_2(dba)_3$ (56 mg, 0.061 mmol), XPhos (57 mg, 0.12 mmol), Cs_2CO_3 (521 mg, 1.60 mmol), naphthalene boronic acid (206 mg, 1.20 mmol), 1-iodobiphenylene (223 mg, 0.802 mmol), and dioxane (2.4 mL). The crude product was purified by flash column chromatography (silica gel, hexane).

Yield of 1f: 79% (177 mg, 0.636 mmol); yellow oil.

^{1}H NMR (400 MHz, $CDCl_3$) δ 8.08 (d, *J* = 7.8 Hz, 1H, CH), 7.95 – 7.88 (m, 2H, CH), 7.56 – 7.53 (m, 4H, CH), 6.99 – 6.91 (m, 2H, CH), 6.74 – 6.73 (m, 3H, CH), 6.65 – 6.61 (m, 1H, CH), 6.02 (d, *J* = 6.8 Hz, 1H, CH).

^{13}C{^{1}H} NMR (100 MHz, $CDCl_3$) δ 151.6 (C), 151.4 (C), 151.3 (C), 150.1 (C), 136.1 (C), 134.0 (C), 130.7 (C), 130.6 (C), 130.5 (CH), 129.0 (CH), 128.5 (CH), 128.4 (CH), 128.3 (CH), 128.3 (CH), 126.5 (CH), 126.4 (CH), 126.1 (CH), 126.0 (CH), 125.6 (CH), 118.6 (CH), 117.4 (CH), 116.3 (CH).

HRMS (CI+) m/z: calculated for $(C_{22}H_{14}+H)^+$ 279.1174, found 279.1179.

1-(2-Methoxyphenyl)biphenylene (1g)

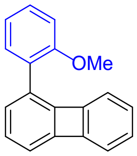

The reaction was conducted according to the general procedure, using $Pd_2(dba)_3$ (55 mg, 0.060 mmol), XPhos (57 mg, 0.120 mmol), Cs_2CO_3 (392 mg, 1.20 mmol), 2-methoxyphenyl boronic acid (183 mg, 1.20 mmol), 1-iodobiphenylene (225 mg, 0.809 mmol), and dioxane (1.8 mL). The crude product was purified by flash column chromatography (silica gel, 1% EtOAc in hexane) and recrystallised from hexane.

Yield of 1g: 65% (134 mg, 0.519 mmol); yellow solid.

^{1}H NMR (500 MHz, $CDCl_3$) δ 7.40 (dd, *J* = 7.5, 1.7 Hz, 1H, CH), 7.35 – 7.31 (m, 1H, CH), 7.03 (td, *J* = 7.5, 3.2 Hz, 1H, CH), 6.99 (d, *J* = 3.7 Hz, 1H, CH), 6.97 (d, *J* = 3.8 Hz, 1H, CH), 6.81 (dd, *J* = 8.4, 6.8 Hz, 1H, CH), 6.75 – 6.69 (m, 2H, CH), 6.66 – 6.65 (m, 1H, CH), 6.61 (d, *J* = 6.8 Hz, 1H, CH), 6.48 – 6.47 (m, 1H, CH), 3.85 (s, 3H, CH_3).

^{13}C{^{1}H} NMR (126 MHz, $CDCl_3$) δ 156.8 (C), 152.4 (C), 151.5 (C), 151.2 (C), 149.9 (C), 129.9 (CH), 129.8 (CH), 129.2 (CH), 128.5 (C), 128.4 (CH), 128.2 (CH), 128.1 (CH), 126.7 (C), 120.8 (CH), 118.1 (CH), 117.1 (CH), 116.0 (CH), 111.1 (CH), 55.5 (CH_3).

HRMS (CI+) m/z: calculated for $(C_{19}H_{14}O+H)^+$ 259.1123, found 259.1128.

1-(2-Methylphenyl)biphenylene (1h)

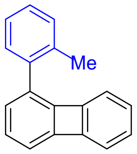

The reaction was conducted according to the general procedure, using $Pd_2(dba)_3$ (55 mg, 0.060 mmol), XPhos (57 mg, 0.119 mmol), Cs_2CO_3 (522 mg, 1.60 mmol), *o*-tolylboronic acid (162.0 mg, 1.19 mmol), 1-iodobiphenylene (223 mg, 0.802 mmol), and dioxane (2.4 mL). The crude product was purified by flash column chromatography (silica gel, 2% EtOAc in hexane).

Yield of 1h: 72% (140 mg, 0.578 mmol); yellow solid.

^{1}H NMR (500 MHz, $CDCl_3$) δ 7.29 – 7.24 (m, 4H, CH), 6.81 (t, *J* = 7.5 Hz, 1H, CH), 6.74 – 6.61 (m, 5H, CH), 6.39 (d, *J* = 6.6 Hz, 1H, CH), 2.37 (s, 3H, CH_3).

^{13}C{^{1}H} NMR (126 MHz, $CDCl_3$) δ 151.7 (C), 151.4 (C), 151.1 (C), 149.4 (C), 137.8 (C), 135.7 (C), 132.0 (C), 130.6 (CH), 130.0 (CH), 129.1 (CH), 128.6 (CH), 128.3 (CH), 128.3 (CH), 127.8 (CH), 126.0 (CH), 117.9 (CH), 117.3 (CH), 116.1 (CH), 20.0 (CH_3).

HRMS (CI+) m/z: calculated for $(C_{19}H_{14}+H)^+$ 243.1174, found 243.1178

1-(2,6-Dimethylphenyl)biphenylene (1i)

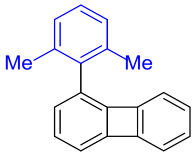

The reaction was conducted according to the general procedure, using $Pd_2(dba)_3$ (55 mg, 0.06 mmol), XPhos (57 mg, 0.120 mmol), Cs_2CO_3 (388 mg, 1.19 mmol), (2,6- dimethyl)phenylboronic acid (137 mg, 0.914 mmol), 1-iodobiphenylene (225 mg, 0.809 mmol), and dioxane (1.8 mL). The crude product was purified by flash column chromatography (silica gel, hexane).

Yield of 1i: 76% (156 mg, 0.609 mmol); yellow oil.

^{1}H NMR (500 MHz, $CDCl_3$) δ 7.17 – 7.09 (m, 3H, CH), 6.82 – 6.78 (m, 1H, CH), 6.74 – 6.63 (m, 4H, CH), 6.56 (d, J = 6.7 Hz, 1H, CH), 6.24 (d, J = 6.7 Hz, 1H, CH), 2.21 (s, 6H, CH_3).

^{13}C{^{1}H} NMR (126 MHz, $CDCl_3$) δ 151.7 (C), 151.5 (C), 151.3 (C), 149.6 (C), 137.3 (C), 136.2 (2C), 130.8 (C), 130.3 (CH), 128.6 (CH), 128.4 (CH), 128.3 (CH), 127.5 (2CH), 127.4 (CH), 117.5 (CH), 117.4 (CH), 116.1 (CH), 20.6 (2CH_3).

HRMS (CI+) m/z: calculated for $(C_{20}H_{16}+H)^+$ 257.1330, found 257.1332.

1-(2-Isopropylphenyl)biphenylene (1j)

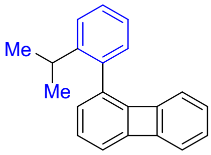

The reaction was conducted according to the general procedure, using $Pd_2(dba)_3$ (55 mg, 0.060 mmol), XPhos (57 mg, 0.120 mmol), Cs_2CO_3 (521 mg, 1.60 mmol), (2- *iso*propyl)phenyl boronic acid (197 mg, 1.20 mmol), 1-iodobiphenylene (225 mg, 0.809 mmol), and dioxane (2.4 mL). The crude product was purified by flash column chromatography (silica gel, hexane - 1.5 % EtOAc in hexane).

Yield of 1j: 80% (174 mg, 0.644 mmol); yellow oil.

^{1}H NMR (400 MHz, $CDCl_3$) δ 7.42 – 7.41 (m, 1H, CH), 7.38 – 7.36 (m, 1H, CH), 7.25 – 7.21 (m, 2H, CH), 6.82 (dd, J = 8.6, 6.8 Hz, 1H, CH), 6.77 – 6.64 (m, 5H, CH), 6.39 – 6.37 (m, 1H, CH), 3.29 (spt, J = 6.8 Hz, 1H, CH), 1.21 (d, J = 6.9 Hz, 6H, CH_3).

^{13}C{^{1}H} NMR (100 MHz, $CDCl_3$) δ 151.6 (C), 151.4 (C), 151.2 (C), 149.3 (C), 146.6 (C), 136.9 (C), 132.0 (C), 130.3 (CH), 129.3 (CH), 128.4 (CH), 128.3 (CH), 128.3 (CH), 128.2 (CH), 125.7 (CH), 125.7 (CH), 117.6 (CH), 117.5 (CH), 116.1 (CH), 29.5 (CH), 24.2 (2CH_3).

HRMS (CI+) m/z: calculated for $(C_{21}H_{18}+H)^+$ 271.1487, found 271.1477.

1-(4-Methoxyphenyl)biphenylene (1k)

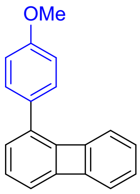

The reaction was conducted according to the general procedure, using $Pd_2(dba)_3$ (55 mg, 0.060 mmol), XPhos (57 mg, 0.120 mmol), Cs_2CO_3 (390 mg, 1.20 mmol), 4- methoxyphenyl boronic acid (182 mg, 1.198 mmol), 1-iodobiphenylene (225 mg, 0.809 mmol), and dioxane (1.8 mL). The crude product was purified by flash column chromatography (silica gel, 2% EtOAc in hexane).

Yield of 1k: 81% (168 mg, 0.650 mmol); yellow solid.

^{1}H NMR (500 MHz, $CDCl_3$) δ 7.54 (d, *J* = 8.6 Hz, 2H, CH), 7.00 (d, *J* = 8.7 Hz, 2H, CH), 6.97 (d, *J* = 8.6 Hz, 1H, CH), 6.86 – 6.84 (m, 1H, CH), 6.79 – 6.76 (m, 3H, CH), 6.69 – 6.68 (m, 1H, CH), 6.60 (d, *J* = 6.8 Hz, 1H, CH), 3.86 (s, 3H, CH_3).

^{13}C{^{1}H} NMR (126 MHz, $CDCl_3$) δ 159.4 (C), 151.6 (C), 151.5 (C), 151.3 (C), 147.2 (C), 131.2 (C), 129.9 (C), 129.4 (CH), 128.4 (CH), 128.2 (CH), 127.7 (2CH), 126.8 (CH), 117.7 (CH), 117.3 (CH), 115.7 (CH), 114.3 (2CH), 55.4 (CH_3).

HRMS (CI+) m/z: calculated for $(C_{19}H_{14}O+H)^+$ 259.1123, found 259.1128.

1-(4-(Dimethylamino)phenyl)biphenylene (1l)

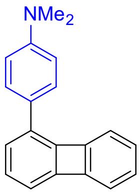

The reaction was conducted according to the general procedure, using $Pd_2(dba)_3$ (41 mg, 0.045 mmol), XPhos (43 mg, 0.090 mmol), Cs_2CO_3 (391 mg, 1.20 mmol), *p*-dimethylaminophenyl boronic acid (149 mg, 0.903 mmol), 1-iodobiphenylene (162.0 mg, 0.583 mmol), and dioxane (1.4 mL). The crude product was purified by flash column chromatography (silica gel, 5% EtOAc in hexane).

Yield of 1l: 74% (117 mg, 0.431 mmol); orange solid.

^{1}H NMR (400 MHz, $CDCl_3$) δ 7.51 – 7.49 (m, 2H, CH), 6.98 (d, *J* = 8.4 Hz, 1H, CH), 6.84 – 6.74 (m, 6H, CH), 6.66 – 6.65 (m, 1H, CH), 6.55 (d, *J* = 6.7 Hz, 1H, CH), 3.01 (s, 6H, CH_3).

^{13}C{^{1}H} NMR (100 MHz, $CDCl_3$) δ 152.0 (C), 151.5 (C), 151.4 (C), 150.2 (C), 146.6 (C), 131.8 (C), 129.2 (CH), 128.2 (CH), 128.1 (CH), 127.4 (2CH),126.6 (CH), 125.2 (C), 117.6 (CH), 117.1 (CH), 115.1 (CH), 112.6 (2CH), 40.6 (2CH_3).

HRMS (ESI+) m/z: calculated for $(C_{20}H_{17}N+H)^+$ 272.1439, found 272.1440.

1-(4-Methylphenyl)biphenylene (1m)

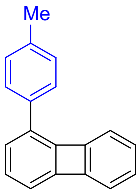

The reaction was conducted according to the general procedure, using $Pd_2(dba)_3$ (55 mg, 0.060 mmol), XPhos (57 mg, 0.120 mmol), Cs_2CO_3 (521 mg, 1.60 mmol), *p*-tolylboronic acid (163 mg, 1.20 mmol), 1-iodobiphenylene (226 mg, 0.813 mmol), and dioxane (2.4 mL). The crude product was purified by flash column chromatography (silica gel, hexane).

Yield of 1m: 81% (157 mg, 0.648 mmol); yellow solid.

^{1}H NMR (500 MHz, $CDCl_3$) δ 7.47 (d, J = 7.9 Hz, 2H, CH), 7.24 – 7.22 (m, 2H, CH), 6.96 (d, J = 8.5 Hz, 1H, CH), 6.81 (t, J = 7.6 Hz, 1H, CH), 6.74 – 6.73 (m, 3H, CH), 6.65 – 6.64 (m, 1H, CH), 6.57 (d, J = 6.7 Hz, 1H, CH), 2.38 (s, 3H, CH_3).

^{13}C{^{1}H} NMR (126 MHz, $CDCl_3$) δ 151.6 (2C), 151.4 (C), 147.7(C), 137.7 (C), 134.6 (C), 131.5 (C), 129.6 (2CH), 129.3 (CH), 128.5 (CH), 128.3 (CH), 127.0 (CH), 126.5 (2CH), 117.9 (CH), 117.4 (CH), 116.0 (CH), 21.4 (CH_3).

HRMS (CI+) m/z: calculated for $(C_{19}H_{14}+H)^+$ 243.1174, found 243.1160.

***Tert*-butyl 3-(biphenylen-1-yl)benzoate (1o)**

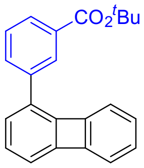

The reaction was conducted according to the general procedure, using $Pd_2(dba)_3$ (55 mg, 0.060 mmol), XPhos (57 mg, 0.120 mmol), Cs_2CO_3 (392 mg, 1.20 mmol), (3- *tert*butoxycarbonyl)phenyl boronic acid (266 mg, 1.20 mmol), 1-iodobiphenylene (225 mg, 0.809 mmol), and dioxane (1.8 mL). The crude product was purified by flash column chromatography (silica gel, 2.5 % EtOAc in hexane).

Yield of 1o: 75% (201 mg, 0.612 mmol); yellow oil.

^{1}H NMR (400 MHz, $CDCl_3$ δ 8.26 (t, J = 1.6 Hz, 1H, CH), 7.98 (dt, J = 8.0, 1.4 Hz, 1H, CH), 7.76 – 7.74 (m, 1H, CH), 7.50 (t, J = 7.7 Hz, 1H, CH), 7.05 (d, J = 8.4 Hz, 1H, CH), 6.90 – 6.84 (m, 2H, CH), 6.80 – 6.78 (m, 2H, CH), 6.70 – 6.67 (m, 1H, CH), 6.62 (d, J = 6.7 Hz, 1H, CH), 1.64 (s, 9H, CH_3).

^{13}C{^{1}H} NMR (100 MHz, $CDCl_3$) δ 165.7 (C), 151.7 (C), 151.3 (2C), 148.3 (C), 137.2 (C), 132.7 (C), 130.4 (C), 129.9 (CH), 129.5 (CH), 128.9 (CH), 128.7 (2CH), 128.4 (CH), 127.6 (CH), 126.5 (CH), 118.2 (CH), 117.5 (CH), 116.5 (CH), 81.4 (C), 28.4 ($3CH_3$).

HRMS (ESI+) m/z: calculated for $(C_{23}H_{20}O_2+Na)^+$ 351.1361, found 351.1357.

1-(Thiophen-3-yl)biphenylene (1r)

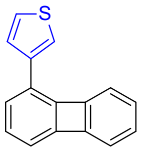

The reaction was conducted according to the general procedure, using $Pd_2(dba)_3$ (55 mg, 0.060 mmol), XPhos (57 mg, 0.120 mmol), Cs_2CO_3 (388 mg, 1.19 mmol), 3-thiophene boronic acid (154 mg, 1.20 mmol), 1-iodobiphenylene (225 mg, 0.809 mmol), and dioxane (1.8 mL). The crude product was purified by flash column chromatography (silica gel, hexane).

Yield of **1r**: 73% (137 mg, 0.585 mmol); yellow solid.

^{1}H NMR (400 MHz, $CDCl_3$) δ 7.45 – 7.44 (m, 1H, CH), 7.41 – 7.39 (m, 1H, CH), 7.36 – 7.35 (m, 1H, CH), 6.97 (d, *J* = 8.5 Hz, 1H, CH), 6.83 – 6.77 (m, 4H, CH), 6.69 – 6.66 (m, 1H, CH), 6.57 (d, *J* = 6.7 Hz, 1H, CH).

^{13}C{^{1}H} NMR (100 MHz, $CDCl_3$) δ 151.5 (C), 151.3 (C), 151.1 (C), 147.2 (C), 138.9 (C), 129.3 (CH), 128.5 (CH), 128.4 (CH), 126.8 (CH), 126.5 (C), 126.3 (CH), 126.2 (CH), 121.4 (CH), 117.8 (CH), 117.4 (CH), 116.0 (CH).

HRMS (CI+) m/z: calculated for $(C_{16}H_{10}S+H)^+$ 235.0581, found 235.0586.

2.2.3 Preparation of 1-(2-pyridylbiphenylene (1s) and 1-(4-(Trifluoro-methyl)phenyl)biphenylene (1n)

1-(2-Pyridyl)biphenylene (1s)

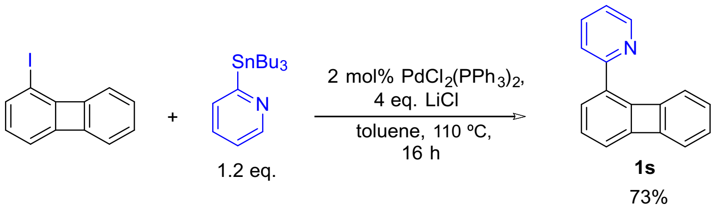

The synthesis was conducted using a modified literature procedure.[110] A Schlenk bomb was evacuated, filled with argon and charged with $PdCl_2(PPh_3)_2$ (12 mg, 0.016 mmol), LiCl (135 mg, 3.18 mmol), 1-iodobiphenylene (222 mg, 0.80 mmol), a magnetic stir bar and toluene (4 mL). To this suspension was added 2-(tributylstannyl)pyridine (0.31 mL, 0.96 mmol) to give a dark brown reaction mixture. The Schlenk bomb was sealed and the reaction mixture stirred (600 rpm) at 110 °C for 16 h. After cooling to room temperature, a saturated solution of KF (4 mL) was added. The reaction mixture was stirred (600 rpm) at room temperature for 30 min, then filtered and washed with DCM (2 x 4 mL). The two phases were separated and the aqueous phase was extracted with DCM (2 x 4 mL). The combined organic layers were dried over $MgSO_4$, filtered, and the solvent was removed under reduced pressure. The crude product was purified by flash column chromatography (silica gel, 5% EtOAc in hexane).

Yield of 1s: 73% (135 mg, 0.589 mmol); yellow oil.

^{1}**H NMR** (400 MHz, $CDCl_3$) δ 8.72 – 8.70 (m, 1H, CH), 7.76 (td, *J* =7.6, 3.4 Hz, 1H, CH), 7.63 (d, *J* = 7.9 Hz, 1H, CH), 7.41 (d, *J* = 8.5 Hz, 1H, CH), 7.22 (dd, J= 7.5, 1.9 Hz, 1H, CH), 6.95 – 6.93 (m, 1H, CH), 6.88 (dd, J = 8.5, 5.1 Hz, 1H, CH), 6.80 (dd, J = 5.2, 2.6 Hz, 2H, CH), 6.70 – 6.68 (m, 1H, CH), 6.66 (d, J = 6.6 Hz, 1H, CH). The ^{1}H NMR spectrum is consistent with that reported in the literature.[110]

1-(4-(Trifluoromethyl)phenyl)biphenylene (1n)

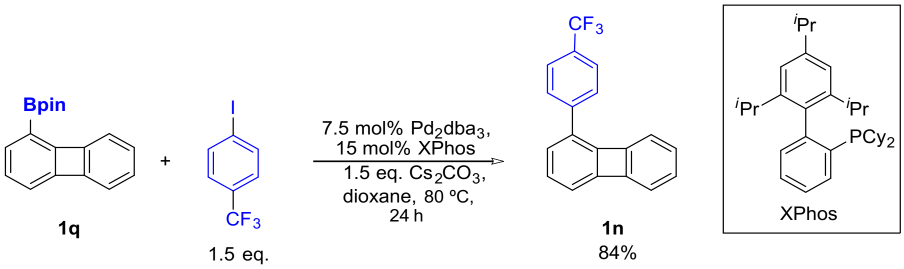

A Schlenk bomb was charged with $Pd_2(dba)_3$ (54 mg, 0.059 mmol), Cs_2CO_3 (381 mg, 1.17 mmol), XPhos (56 mg, 0.117 mmol), 4-iodotrifluoromethylbenzene (175 µL, 1.17 mmol) and a magnetic stir bar. A solution of 1-(4,4,5,5-tetramethyl-1,3,2-dioxaborolan-2-yl)biphenylene (217 mg, 0.780 mmol) in dioxane (1.8 mL) was then added to give a brown suspension. The Schlenk bomb was sealed and the reaction mixture stirred (600 rpm) at 80 °C for 24 h. The reaction mixture was cooled to room temperature, filtered through a plug of celite and the plug was washed with EtOAc (~200 mL) until no compound was visible by UV on a TLC plate. The solvent was removed under reduced pressure and the crude product was purified by flash column chromatography (silica gel, hexane).

Yield of 1n: 84% (195 mg, 0.658 mmol); yellow solid.

^{1}**H NMR** (400 MHz, $CDCl_3$) δ 7.71 – 7.67 (m, 4H, CH), 6.98 (d, *J* = 8.5 Hz, 1H, CH), 6.88 (t, *J* = 7.6 Hz, 1H, CH), 6.82 – 6.73 (m, 3H, CH), 6.69 (d, *J* = 6.2 Hz, 1H, CH), 6.66 (d, *J* = 6.8 Hz, 1H, CH).

^{19}**F{^{1}H} NMR** (377 MHz, $CDCl_3$) δ -62.45.

^{13}**C{^{1}H} NMR** (126 MHz, $CDCl_3$) δ 151.8 (C), 151.3 (C), 150.9 (C), 148.7 (C), 141.1 (C), 130.0 (C), 129.8 (q, *J* = 32.6 Hz, C), 129.7 (CH), 129.0 (CH), 128.6 (CH), 126.9 (CH), 126.8 (2CH), 125.9 (q, *J* = 3.8 Hz, 2CH), 139.3 (q, *J* = 272.0 Hz, C), 118.1 (CH), 117.8 (CH), 117.0 (CH).

HRMS (CI+) m/z: calculated for $(C_{19}H_{11}F_3+H)^+$ 297.0891, found 297.0887.

3. Screening conditions for the ring opening diborylation of 1-fluorobiphenylene

Table S1. Initial screening of Ir, Ni and Pd precursors and ligands in the C–C diborylation of 1-fluorobiphenylene.

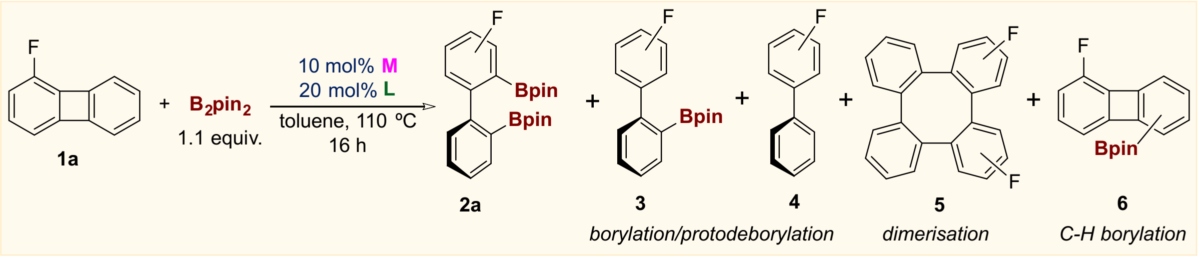

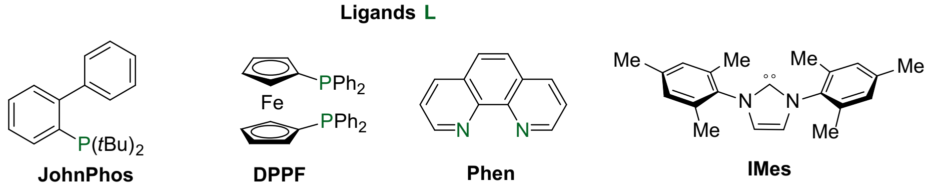

Entry	Metal	Ligand	Conversion[a] of **1a** (%)	Yield,[a] %			
				**2a**	**3**	**4**	**5**
1	Pd(dba)$_2$	P(*n*-Bu)$_3$	5	1	1	<1	0
2[b]	Pd(dba)$_2$	P(*n*-Bu)$_3$	11	5	1	<1	0
3	Pd(dba)$_2$	DPPF	4	0	<1	<1	0
4[c]	Pd(dba)$_2$	IMes	80	8	10	16	0
5	Pd(dba)$_2$	JohnPhos	99	7	4	<1	77
6	Pd(dba)$_2$	Phen	4	2	0	<1	0
7	[Ir(cod)Cl]$_2$	DPPF	13	<1	<1	<1	0
8[c]	[Ir(cod)Cl]$_2$	IMes	56	0	10	20	0
9	[Ir(cod)Cl]$_2$	JohnPhos	24	<1	3	3	0
10[d]	[Ir(cod)Cl]$_2$	Phen	29	0	0	0	0
11	[Ir(cod)Cl]$_2$	P(*n*-Bu)$_3$	1	0	0	0	0
12	Ni(cod)$_2$	DPPF	11	0	<1	2	0
11[c]	Ni(cod)$_2$	IMes	99	<1	5	18	59
14	Ni(cod)$_2$	JohnPhos	1	0	0	0	0

[a] All conversions and yields are based on GC analysis using n-dodecane as the internal standard (0.1 mmol scale). [b] Reaction was conducted in *m*-xylene. [c] IMes was generated *in situ* by the treatment of the chloride salt with 22 mol% KO^{t}Bu. [d] 98% conversion of B_2Pin_2. C-H borylated toluene and **1a** (**6**) are the main products.

3.1 General procedure for the screening of catalysts in the ring opening diborylation of 1-fluorobiphenylene

In an argon-filled glovebox, a Schlenk bomb with a Teflon tap was charged with catalyst (10 µmol for $Pd(dba)_2$ and $Ni(cod)_2$, 5 µmol for $[Ir(cod)Cl]_2$), ligand (20 µmol for monodentate ligands and 10 µmol for bidentate ligands), and B_2pin_2 (0.11 mmol for all experiments except Entry 14, Table 1). All carbene ligands were used as either the chloride or the tetrafluoroborate salt and were released in-situ by adding a slight excess of KO^{t}Bu (22 µmol). 1 mL of stock solution of 1-fluorobiphenylene (0.1 mmol/mL) and n-dodecane (internal standard for GC, 0.05 mmol/mL) in toluene was added to the reaction vessel. The Schlenk bomb was sealed by closing the Teflon tap and the reaction mixture stirred at room temperature for 10 min. The reaction vessel was then removed from the glovebox and the reaction mixture was then stirred in an oil bath at 110 °C for 16 h. After cooling to room temperature, the reaction mixture was filtered through a plug of celite, and the plug was washed with EtOAc, until no compound was visible on a TLC plate in UV light. The filtrate was analyzed by GC using an internal standard (dodecane). GC yields were calculated using the response factor of 0.8 for both isomers of the desired product **2a** and 0.9 for all isomers of the monoborylated product **3**. The response factors of all isomers of the reduced product **4** and the tetraphenylene product **5** were assumed to be the same as those for 1-fluorobiphenylene (**1a**).

3.2 Additional correlations of the product yield with electronic and steric parameters of NHC ligands in the model C–C borylation of 1a (Table 1).

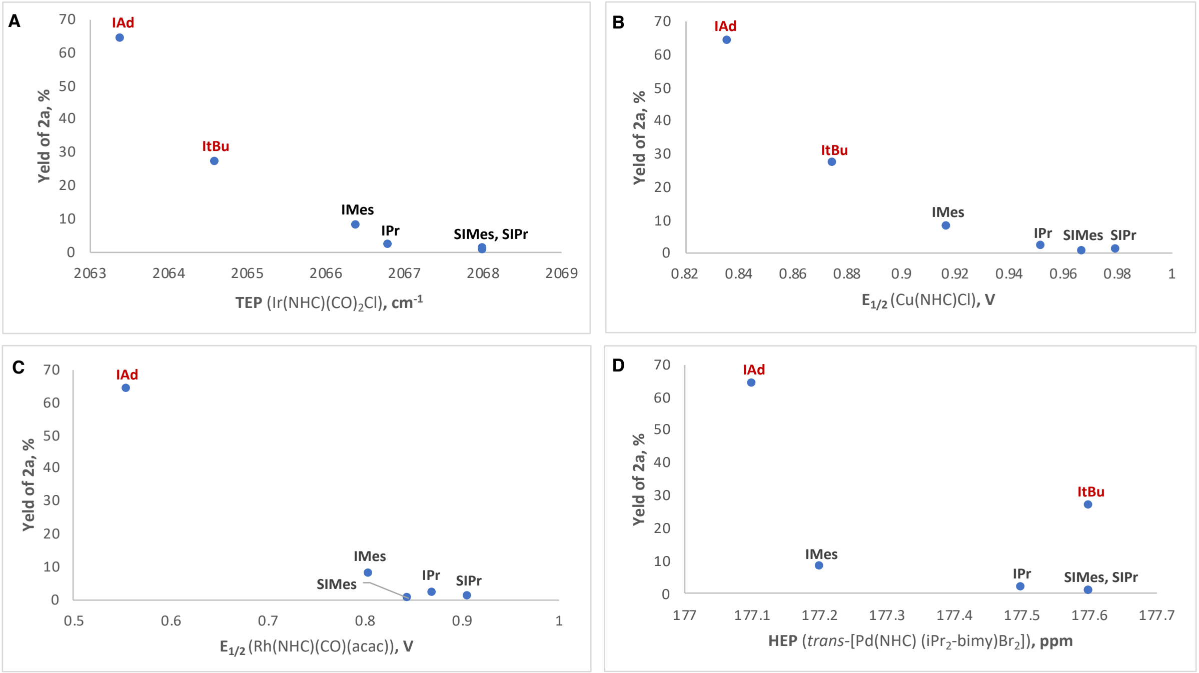

Figure S4. Correlation of yields of **2a** with electron-donating properties of NHC ligands quantified as (**A**) Tolman Electronic Parameter (TEP, cm^{-1}) for $Ir(NHC)(CO)_2Cl$[111] (✓);
(**B**) $E_{1/2}$ (V) for Cu(NHC)Cl[112] (✓);
(**C**) $E_{1/2}$ (V) for Rh(NHC)(CO)(acac)[112] (✓);
(**D**) Huynh Electronic Parameter (HEP, ppm) for *trans*-[Pd(NHC) (iPr_2-bimy)Br_2][113] (×).

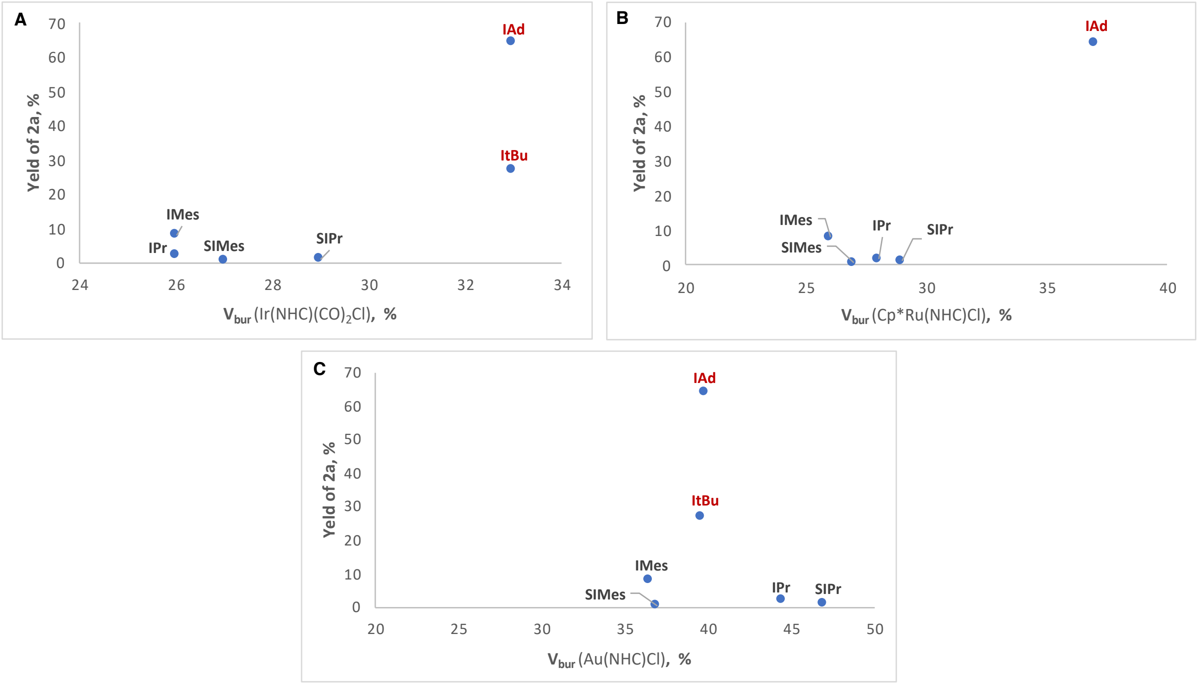

Figure S5. Correlation of yields of **2a** with steric properties of NHC ligands quantified as buried volume (V_{bur}, %) for (**A**) $Ir(NHC)(CO)_2Cl$[79] (≈); (**B**) Cp*Ru(NHC)Cl[78] (≈); (**C**) Au(NHC)(Cl)[80] (×).

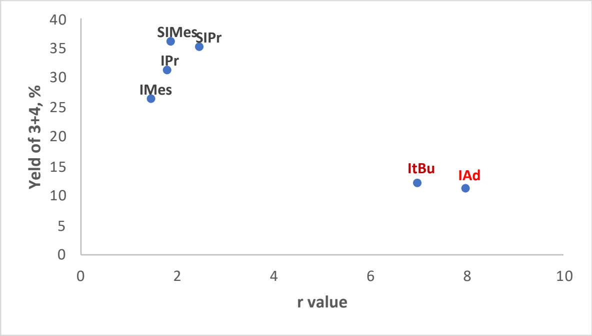

Figure S6. Correlation of yields of side products **3** and **4** with repulsiveness parameter (r)[75] of the tested NHC ligands.

4. Scope of the palladium-catalyzed C–C diborylation of biphenylenes with B_2pin_2

4.1 General procedure for the C–C diborylation of 1-monosubstituted biphenylenes

In an argon-filled glovebox, a Schlenk bomb with a Teflon tap was charged with $Pd(dba)_2$ (40 µmol), 1,3-bis(1-adamantylimidazolium)tetrafluoroborate (80 µmol), KO*t*Bu (88 µmol), B_2pin_2 (0.8 mmol) and a magnetic stir bar. A solution of biphenylene (0.4 mmol) in toluene (4 mL) was added to give a wine-red suspension. The Schlenk bomb was sealed by closing the Teflon tap and the reaction mixture was stirred (600 rpm) at room temperature for 10 minutes. The Schlenk bomb was removed from the glovebox and the reaction was stirred (600 rpm) at the specified temperature in an oil bath for 16 h. Upon heating, the mixture changed colour from wine-red to khaki green. The reaction mixture was then cooled to room temperature, filtered through celite and the plug was washed with EtOAc until no compound was visible on a TLC plate in UV light. The solvent was removed under reduced pressure to give the crude product, which was purified by flash column chromatography.

Notes: When the substrate was a 1-arylbiphenylene, except for 1-(thiophen-3-yl)biphenylene (**1r**) and 1-(4-methoxyphenyl)biphenylene (**1k**), both regioisomers had a similar R_f values and could not be completely separated by column chromatography. The main regioisomer was obtained in pure form and fully characterized. A mixture of regioisomers was also isolated, and the yields of the two regioisomers were calculated from their ratio determined by integration of the ^1H NMR spectra. In most cases, the minor regioisomer, separated from the major regioisomer, contained small impurities and the accurate yield was calculated by adding a known amount of standard, either 1,3,5-trimethoxybenzene or durene. The ratio of regioisomers was determined from the isolated yields, but where possible, i.e. when there were non-overlapping signals in the NMR spectrum of the crude compound, the ratio determined from isolated yields was checked against the ratio determined from the crude NMR spectrum to ensure that there were no significant differences.

The identities of the major and minor regioisomers resulting from the ring-opening diborylation of all 1-arylbiphenylenes and 1-methylbiphenylene were assigned by ^1H NMR spectroscopy on the basis of the XRD structure of the major tri-*ortho*-substituted regioisomer **2e**, obtained from borylation of 1-phenylbiphenylene (**1e**). The major regioisomer of **2e** had four Bpin methyl environments, whereas the di-*ortho* substituted minor regioisomer of **2e** had two. The same trend holds for the regioisomers resulting from borylation of all other 1-arylbiphenylenes and 1-methylbiphenylene and their identities were therefore assigned based on the number of Bpin methyl environments.

In all products of the ring-opening diborylation of 1-aryl, 1-TMS, 1-Bpin, 1-F, and 1-Me biphenylenes two quaternary carbon signals were not observed in ^{13}C NMR spectra. These signals correspond to the quaternary carbon atoms bonded to boron, which are not observed because of the rapid quadrupolar relaxation of ^{11}B.

C–C diborylation of 1-fluorobiphenylene (1a) at 110 °C (Figure 2)

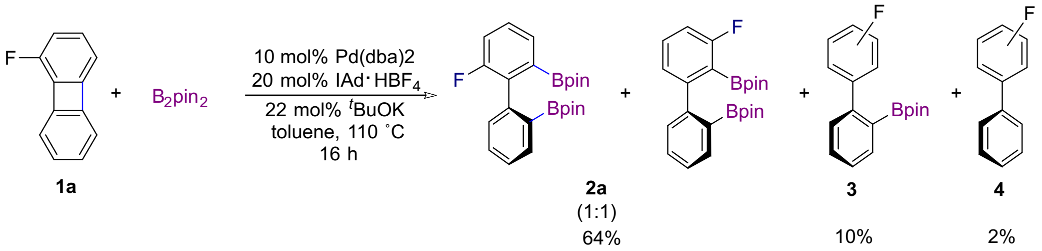

The reaction was conducted according to the general procedure, using 1-fluorobiphenylene (85.1 mg, 0.500 mmol), B_2pin_2 (251 mg, 1.00 mmol), $Pd(dba)_2$ (28.5 mg, 0.0496 mmol), 1,3-bis(1-adamantyl)imidazolium tetrafluoroborate (42.3 mg, 0.10 mmol), KO^{t}Bu (12.3 mg, 0.110 mmol), and toluene (5 mL). The reaction mixture was stirred at 110 °C for 16 h. The ratio of the two regioisomers in the crude reaction mixture was 1:1, as determined by GC. The crude product was purified by flash chromatography on silica gel (two columns: 5 % EtOAc/hexanes; then 20–50 % DCM/hexanes).

Yield 2a (both regioisomers): 64% (134 mg, 0.313 mmol). Two regioisomers were not fully separated and isolated as the following mixed fractions: White solid (99 mg, 0.230 mmol, 47 %; reg. 1/reg. 2 = 3:1) from the first column. Colourless oil (35 mg, 0.083 mmol, 17 %; reg. 1/reg. 2 = 1:20) from the second column after re-chromatography of the mixed fraction. Regioisomer ratios were determined by ^{1}H NMR (signals at 6.96 ppm and 7.10 ppm).

Side products: **3** (mixture of isomers, colourless oil, 16 mg, 0.054 mmol, 10%); **4** (mixture of isomers, colourless oil, 2 mg, 0.01 mmol, 2%).

Characterization of 2a (both regioisomers).

^{1}H NMR (400 MHz, $CDCl_3$) δ 7.76 (dd, *J* = 7.4, 1.0 Hz, 1H reg. 1, CH), 7.73 – 7.71 (m, 1H reg. 2, CH), 7.44 – 7.37 (m, 2H reg. 1, 1H reg. 2, CH), 7.34 – 7.23 (m, 3H reg. 1, 3H reg. 2, CH), 7.10 (t, *J* = 8.9 Hz, 1H reg. 1, CH), 7.04 (d, *J* = 7.4 Hz, 1H reg. 2, CH), 6.96 (t, *J* = 8.5 Hz, 1H reg. 2, CH), 1.14 (s, 12H reg. 2, CH_3), 1.11 (s, 6H reg. 1, CH_3), 1.07 – 1.05 (m, 18H reg. 1, 12H reg. 2, CH_3).

^{19}F{^{1}H} NMR (377 MHz, $CDCl_3$) δ -107.0 (reg. 2), -115.7 (reg. 1).

^{13}C{^{1}H} NMR (100 MHz, $CDCl_3$) δ 165.2 (d, *J* = 242.6 Hz, reg. 2, C), 160.0 (d, *J* = 243.4 Hz, reg. 1, C), 150.4 (d, *J* = 9.0 Hz, reg. 2, C), 148.1 (reg. 2, C), 142.5 (reg. 1, C), 136.1 (d, *J* = 16.2 Hz, reg. 1, C), 134.5 (reg. 2, CH), 134.4 (reg. 1, CH), 130.1 (d, *J* = 8.5 Hz, reg. 2, CH), 130.0 (reg. 1, CH), 129.7 (reg. 2, CH), 129.6 (reg. 1, CH), 129.3 (reg. 2, CH), 129.2 (d, *J* = 3.5 Hz, reg. 1, CH), 127.9 (d, *J* = 7.8 Hz, reg. 1, CH), 126.6 (reg. 1, CH), 126.4 (reg. 2, CH), 124.9 (d, *J* = 2.8 Hz, reg. 2, CH), 116.7 (d, *J* = 23.2 Hz, reg. 1, CH), 112.7 (d, *J* = 24.2 Hz, reg. 2, CH), 83.3 (reg. 1, 2C), 83.4 (reg. 2, 2C), 83.5 (reg. 1, 2C), 83.8 (reg. 2, 2C), 24.5 (reg. 1, 2CH_3), 24.6 (reg. 1, 2CH_3, reg. 2, 4CH_3), 24.7 (reg. 1, 2CH_3), 24.8 (reg. 2, 4CH_3), 24.9 (reg. 1, 2CH_3).

HRMS (ESI+) m/z: calcd for $[C_{24}H_{31}B_2O_4F+Na]^+$ 447.2290; found 447.2304.

Attempted C–C diborylation of 1-chlorobiphenylene (1a) at 110 and 140 °C (Figure 2)

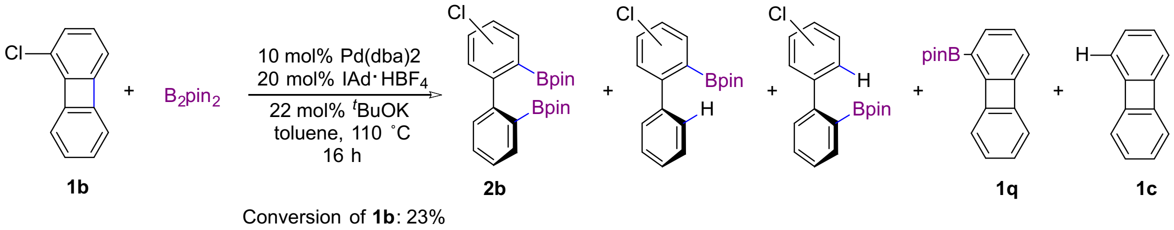

The reaction was conducted according to the general procedure, using 1-chlorobiphenylene (18.6 mg, 0.0997 mmol), n-dodecane (5.4 mg, 30 µmol) B_2pin_2 (50 mg, 0.20 mmol), KO*t*Bu (2.6 mg, 23 µmol), 1,3-bis(1-adamantylimidazolium) tetrafluoroborate (8.6 mg, 20 µmol), $Pd(dba)_2$ (5.9 mg, 10 µmol), and toluene (1 mL). The reaction mixture was stirred at 110 °C for 16 h. The reaction mixture was then cooled to room temperature, filtered through celite and the celite plug was then washed with EtOAc until no compound was visible on a TLC plate in UV light. GC analysis of the resulting filtrate showed that the conversion of **1b** was 23%. According to GC-MS, only traces of the expected product of diborylation (**2b**) was observed with a mixture of products of monoborylation of the C–C bonds, borylation and reduction of the C–Cl bond (**1q** and biphenylene). Conducting the reaction at an elevated temperature of 140 °C for 16 h, led to a comparable conversion (21%) and similar array of products.

C–C diborylation of biphenylene (1c) at 110 °C (Figure 2)

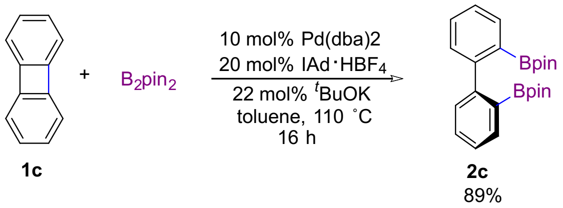

The reaction was conducted according to the general procedure, using biphenylene, **1c** (60.9 mg, 0.400 mmol), B_2pin_2 (205 mg, 0.807 mmol), $Pd(dba)_2$ (23.1 mg, 0.0402 mmol), 1,3-bis(1-adamantyl)imidazolium tetrafluoroborate (34.1 mg, 0.0804 mmol), KO^{t}Bu (10.0 mg, 0.0891 mmol), and toluene (4 mL). The reaction mixture was stirred at 110 °C for 16 h. The crude product was purified by flash column chromatography (silica gel, 4% EtOAc in hexane).

Yield of 2c: 89%; white solid (144 mg, 0.353 mmol).

Side product: traces of C–C monoborylation product (~4%, mixture of isomers, identified by GC-MS).

Characterization of 2c.

^{1}H NMR (400 MHz, $CDCl_3$) δ 7.66 (dd, *J* = 7.4, 2.8 Hz, 2H, CH), 7.38 (td, *J* = 7.5, 3.3 Hz, 2H, CH), 7.31 – 7.25 (m, 4H, CH), 1.09 (s, 24H, CH_3). The ^{1}H NMR data were consistent with that reported in the literature.[39, 114, 115]

C–C diborylation of 1-methylbiphenylene (1d) at 110 °C (Figure 2)

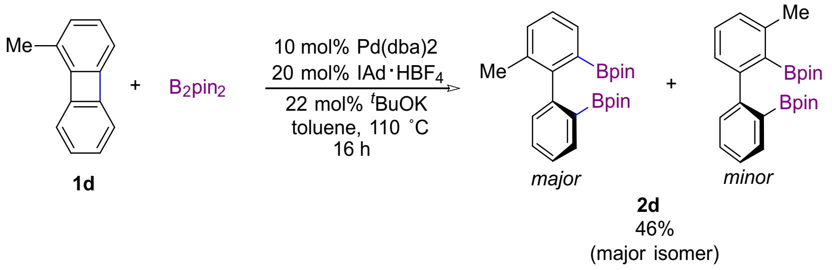

The reaction was conducted according to the general procedure, using **1d** (66.8 mg, 0.402 mmol), B_2pin_2 (203 mg, 0.800 mmol), $Pd(dba)_2$ (23.2 mg, 0.0403 mmol), 1,3-bis(1-adamantyl)imidazolium tetrafluoroborate (34.1 mg, 0.0804 mmol), KO*t*Bu (10.2 mg, 0.0909 mmol), and toluene (4 mL). The reaction mixture was stirred at 110 °C for 16 h. The crude product was purified by flash column chromatography (silica gel, 2-4% EtOAc in hexane).

Yield of 2d (major): 46% (77 mg, 0.19 mmol); white solid.

Minor isomer **2d**: mixture with an unidentified product (yellow oil, 30 mg); not assigned unambiguously and nor quantified. **HRMS** (ESI^+) m/z: calcd for $[C_{25}H_{34}B_2O_4+Na]^+$ 443.2541; found 443.2545.

Characterization of the major isomer of 2d.

^{1}H NMR (500 MHz, $CDCl_3$) δ 7.69 (d, *J* = 7.34 Hz, 1H, CH), 7.47 (d, *J* = 7.03 Hz, 1H, CH), 7.37 (td, *J* = 7.54, 3.28 Hz, 1H, CH), 7.28 (td, *J* = 7.49, 2.81 Hz, 1H, CH), 7.23 – 7.22 (d, *J* = 6.79 Hz, 1H, CH), 7.18 (t, *J* = 7.37 Hz, 1H, CH), 7.14 (d, *J* = 7.51 Hz, 1H, CH), 2.02 (s, 3H, CH_3), 1.06 (s, 6H, CH_3), 1.02 (s, 6H, CH_3), 1.01 (s, 6H, CH_3), 1.00 (s, 6H, CH_3).

$^{13}C\{^1H\}$ NMR (126 MHz, $CDCl_3$) δ 148.8 (2C), 135.6 (C), 133.9 (CH), 131.1 (CH), 130.9 (CH), 129.4 (CH), 129.3 (CH), 126.0 (CH), 125.6 (CH), 83.0 (4C), 24.8 ($4CH_3$), 24.5 ($2CH_3$), 24.4 ($2CH_3$), 20.8 (CH_3).

HRMS (ESI^+) m/z: calcd for $[C_{25}H_{34}B_2O_4+Na]^+$ 443.2541; found 443.2546.

C–C diborylation of 1-methylbiphenylene (1d) at 140 °C (Figure 2)

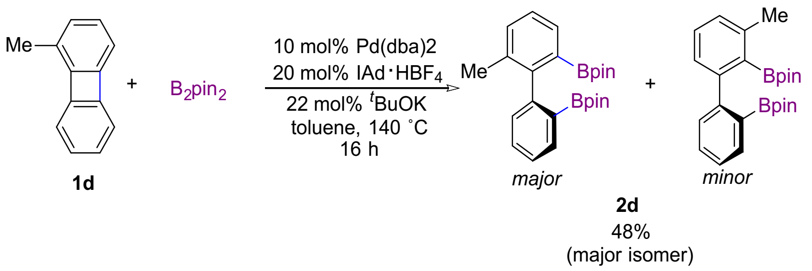

The reaction was conducted according to the general procedure, using **1d** (66.5 mg, 0.400 mmol), B_2pin_2 (205 mg, 0.808 mmol), $Pd(dba)_2$ (23.0 mg, 0.0400 mmol), 1,3-bis(1-adamantyl)imidazolium tetrafluoroborate (34.3 mg, 80 μmol), KO^{t}Bu (10.0 mg, 0.0891 mmol), and toluene (4 mL). The reaction mixture was stirred at 140 °C for 16 h. The crude product was purified by flash column chromatography (silica gel, 2-4% EtOAc in hexane).

Yield of 2d (major): 48%; white solid (81 mg, 0.19 mmol); white solid. Characterization: see the previous section.

Minor isomer of **2d**: mixture with an unidentified product (pale-yellow oil, 26 mg); not assigned unambiguously and not quantified.

C–C borylation of 1-phenylbiphenylene (1e) at 110 °C (Figure 2)

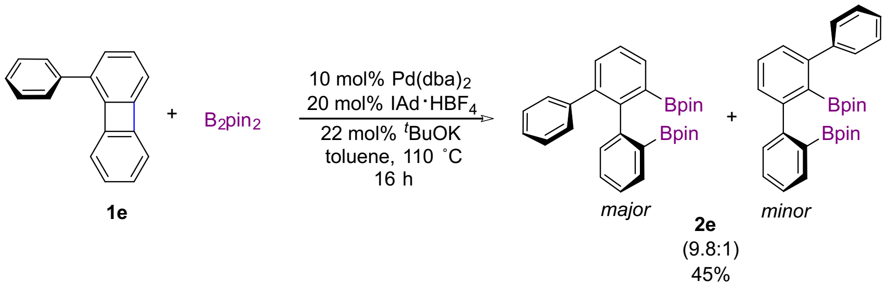

The reaction was conducted according to the general procedure, using 1- phenylbiphenylene, **1e** (45.6 mg 0.200 mmol), B_2pin_2 (102 mg, 0.402 mmol), $Pd(dba)_2$ (11.5 mg, 0.0200 mmol), 1,3-bis(1-adamantyl)imidazolium tetrafluoroborate (17.2 mg, 0.0405 mmol), KO^{t}Bu (4.9 mg, 0.044 mmol), and toluene (2 mL). The reaction mixture was stirred at 110 °C for 16 h. The crude product was purified by flash column chromatography (silica gel, 8% EtOAc in hexane).

Recovered starting material 1e: 22 % (10 mg, 0.044 mmol).

Yield of 2e (major+minor): 45% (0.0895 mmol); major:minor regioisomer ratio = 9.8:1 (0.0812:0.0083).

Major regioisomer 2e: 41% (0.0812 mmol). White solid (36 mg, 0.075 mmol, 37.5%); an additional 3% (0.0062 mmol) could be obtained from the mixed fractions (see below).

Minor regioisomer 2e: 4% (0.083 mmol). Not isolated as a pure individual compound. Yield determined by ^{1}H NMR of mixed fractions using 1,3,5-trimethoxybenzene as an internal standard (see below).

Mixed fractions: (a) a colourless oil (1.0 mg) containing 0.0021 mmol of the minor isomer; (b) a colourless oil (6.0 mg) containing 0.0062 mmol of the major isomer and 0.0062 mmol of the minor isomer.

Characterization of the major isomer 2e.

^{1}H NMR (400 MHz, $CDCl_3$) δ 7.62 – 7.59 (m, 2H, CH), 7.39 – 7.35 (m, 2H, CH), 7.18 – 7.07 (m, 7H, CH), 6.92 – 6.91 (m, 1H, CH), 1.15 (s, 6H, CH_3), 1.08 (s, 6H, CH_3), 1.06 (s, 6H, CH_3), 1.02 (s, 6H, CH_3).

$^{13}C\{^1H\}$ NMR (100 MHz, $CDCl_3$) δ 148.4 (C), 147.4 (C), 142.6 (C), 140.6 (C), 133.8 (CH), 132.3 (CH), 131.3 (CH), 130.4 (CH), 130.1 (2CH), 129.0 (CH), 127.3 (2CH), 126.1 (CH), 125.9 (CH), 125.3 (CH), 83.3 (2C), 83.2 (2C), 25.1 (2CH_3), 25.0 (2CH_3), 24.6 (2CH_3), 24.4 (2CH_3).

HRMS (ESI+) m/z: calcd for $(C_{30}H_{36}B_2O_4+Na)^+$ 505.2697; found 505.2706. Crystals for XRD analysis were grown by slow evaporation of a concentrated hexane solution at room temperature.

Characterization of the minor isomer 2e.

^{1}H NMR (400 MHz, $CDCl_3$) δ 7.76 (d, J = 7.7 Hz, 1H, CH), 7.70 – 7.65 (m, 3H, CH), 7.56 – 7.52 (m, 2H, CH), 7.44 -7.38 (m, 3H, CH), 7.34 – 7.30 (m, 3H, CH), 1.11 (s, 12H, CH_3), 1.06 (m, 12H, CH_3), **^{13}C{^{1}H} NMR** (126 MHz, $CDCl_3$) δ 150.2 (C), 149.5 (C), 141.7 (C), 141.4 (C), 134.4 (CH), 133.7 (CH), 129.3 (2CH), 128.8 (2CH), 128.3 (CH), 127.4 (CH), 127.3 (2CH), 125.9 (CH), 124.5 (CH), 83.3 (4C), 24.7 (8CH_3).

HRMS (ESI+) m/z: calcd for $[C_{30}H_{36}B_2O_4+Na]^+$ 505.2697; found 505.2704.

C–C borylation of 1-phenylbiphenylene (1e) at 140 °C (Figure 2)

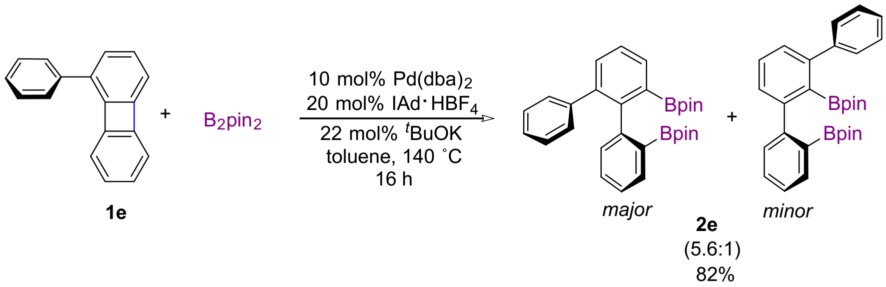

The reaction was conducted according to the general procedure, using 1-phenylbiphenylene, **1e** (126 mg, 0.553 mmol), B_2pin_2 (279 mg, 1.10 mmol), $Pd(dba)_2$ (31.6 mg, 0.0549 mmol), ,3-bis(1-adamantyl)imidazolium tetrafluoroborate (46.8 mg, 0.109 mmol), KO^{t}Bu (13.6 mg, 0.121 mmol), 1 and toluene (5.5 mL). The reaction mixture was stirred at 140 °C for 16 h. The crude product was purified by flash column chromatography (silica gel, 5% EtOAc in hexane).

Yield of 2e (major+minor): 82% (0.451 mmol); major:minor regioisomer ratio = 5.6:1 (0.383 mmol:0.068 mmol).

Major regioisomer 2e: 69% (0.383 mmol). White solid (177 mg, 0.367 mmol, 66%); an additional 3% could be obtained from the mixed fractions (see below).

Minor regioisomer 2e: 12% (0.068 mmol). Not isolated as a pure individual compound. Yield determined by ^{1}H NMR of mixed fractions using1,3,5-trimethoxybenzene as an internal standard.

Mixed fractions: (a) a colourless oil (28 mg) containing 0.053 mmol of the minor isomer; (b) a colourless oil (20 mg) containing 0.016 mmol of the major isomer and 0.015 mmol of the minor isomer. Spectroscopic characterization: see the previous section.

C–C borylation of 1-phenylbiphenylene (1e) at 180 °C (Figure 2)

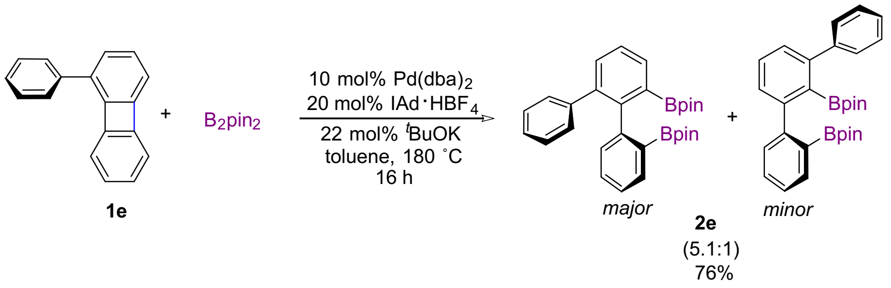

The reaction was conducted according to the general procedure, using 1-phenylbiphenylene, **1e** (91.3 mg 0.400 mmol), B_2pin_2 (204 mg, 0.803 mmol), $Pd(dba)_2$ (23.3 mg, 0.0405 mmol), 1,3-bis(1-adamantyl)imidazolium tetrafluoroborate (33.9 mg, 0.0799 mmol), KO^{t}Bu (9.9 mg, 0.088 mmol), and toluene (4 mL). The reaction mixture was stirred at 180 °C for 16 h. The crude product was purified by flash column chromatography (silica gel, 5% EtOAc in hexane).

Yield of 2e (major+minor): 76% (0.304 mmol); major:minor regioisomer ratio = 5.1:1 (0.254 mmol: 0.05 mmol).

Major regioisomer of 2e: 64% (0.254 mmol). White solid (106 mg, 0.220 mmol, 55%); an additional 9% could be obtained from the mixed fractions (see below).

Minor regioisomer of 2e: 12% (0.05 mmol). Not isolated as a pure individual compound. Yield determined by ^{1}H NMR of mixed fractions using1,3,5-trimethoxybenzene as an internal standard.

Mixed fractions: (a) a colourless oil (9 mg) containing 0.02 mmol of the minor isomer; (b) a colourless oil (31 mg) containing 0.034 mmol of the major isomer and 0.030 mmol of the minor isomer.

Spectroscopic characterization: see the previous section.

C–C borylation of 1-(naphthalen-1-yl)biphenylene (1f) at 140 °C (Figure 2)

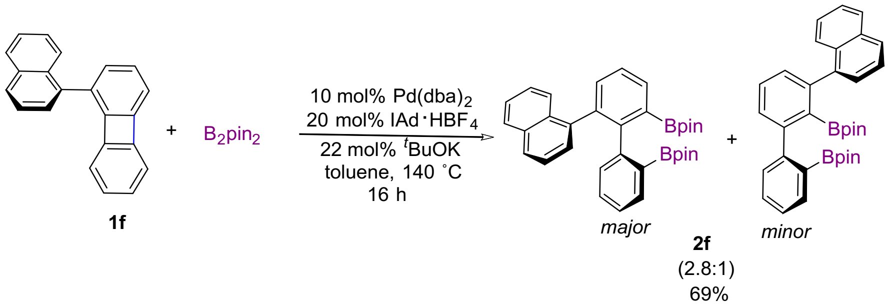

The reaction was conducted according to the general procedure, using 1-(naphthalen-1-yl)biphenylene, **1f** (111 mg 0.400 mmol), B_2pin_2 (203 mg, 0.799 mmol), $Pd(dba)_2$ (23 mg, 0.040 mmol), 1,3-bis(1-adamantyl)imidazolium tetrafluoroborate (34 mg, 0.080 mmol), KO^{t}Bu (10 mg, 0.088 mmol), and toluene (4 mL). The reaction mixture was stirred at 140 °C for 16 h. The crude product was purified by flash column chromatography (silica gel, 7.5% Et_2O in hexane).

Yield of 2f (major+minor): 69% (0.274 mmol); major:minor regioisomer ratio = 2.8:1 (0.201 mmol:0.073 mmol).

Major regioisomer of 2f: 50% (0.201 mmol). White solid (73 mg, 0.14 mmol, 35%), a a mixture of two diastereomers (dr = 2.3:1, based on integrals of the signals at 7.99 ppm and 6.8 ppm); an additional 15% (0.061 mmol) was obtained from the mixed fractions (see below).

Minor regioisomer of 2f: 18% (0.073 mmol). Not isolated as a pure individual compound. Yield determined by ^{1}H NMR of mixed fractions using 1,3,5-trimethoxybenzene as an internal standard.

Mixed fractions: (a) a colourless oil (39 mg) containing 0.054 mmol of the major isomer and 0.020 mmol of the minor isomer; (b) a colourless gum (36 mg) containing mainly minor isomer: 0.0072 mmol of the major isomer and 0.053 mmol of the minor isomer.

Characterization of major regioisomer 2f (dia.1 = major diastereomer, dia.2 = minor diastereomer, un = unassigned)**.**

^{1}H NMR (500 MHz, $CDCl_3$) δ 7.99 (d, *J* = 8.5 Hz, 0.32H dia.2, CH), 7.78 – 7.77 (m, 1.44H un, CH), 7.71 – 7.64 (m, 1.56H, CH), 7.60 (d, *J* = 8.2 Hz, 0.68H dia.1, CH), 7.56 (d, *J* = 7.3 Hz, 0.68H dia.1, CH), 7.48 (d, *J* = 7.4 Hz, 0.29H dia.2, CH), 7.44 – 7.36 (m, 4.14H un, CH), 7.32 - 7.27 (m, 0.9H un, CH), 7.19 – 7.13 (m, 1.39H un, CH), 7.02 – 6.92 (m, 1.47H un, CH), 6.80 (t, *J* = 7.4 Hz, 0.75H dia.1, CH), 6.72 (d, *J* = 7.6 Hz, 0.75H dia.1, CH), 1.27 (s, 1.8H dia.2, CH_3), 1.23 (s, 3.9H dia.1, CH_3), 1.17 - 1.16 (m, 6.2H dia.1 and dia.2, CH_3), 1.09 - 1.08 (m, 6.3H dia.1 and dia.2, CH_3), 1.03 - 1.02 (m, 6.3H dia.1 and dia.2, CH_3).

^{13}C{^{1}H} NMR (100 MHz, $CDCl_3$) δ 148.5 (C dia.1), 148.3 (C dia.1), 147.9 (C dia.2), 147.8 (C dia.2), 140.2 (C dia.2), 139.9 (C dia.1), 139.2 (C dia.2), 138.4 (C dia.1), 134.8 (CH dia.2), 133.7 (CH dia.1), 133.3 (C dia.1, C dia.2), 133.2 (C dia.1), 132.7 (CH dia.2), 132.6 (CH dia.1), 132.5 (CH dia.2), 132.3 (CH dia.1), 130.9 (CH dia.2), 128.8 (CH dia.1), 128.7 (CH dia.2), 128.5 (CH dia.1), 128.4 (CH dia.2), 128.3 (CH dia.2), 128.0 (CH dia.1), 127.9 (CH dia.1), 127.4 (CH dia.2), 127.0 (CH dia.2), 126.8 (CH dia.1), 126.6 (CH dia.1), 125.6 (CH dia.2), 125.5 (CH dia.1), 125.4 (CH dia.1), 125.3 (2CH dia.2), 125.2 (2CH dia.1), 125.0 (CH dia.1), 124.8 (CH dia.2), 124.5 (CH dia.2), 83.3 (4C dia.1, 2C dia.2), 83.2 (2C dia.2), 25.5 (2CH_3 dia.2), 25.2 (2CH_3 dia.1), 25.1 (2CH_3 dia.1), 25.0 (2CH_3 dia.2), 24.7 (2CH_3 dia.1), 24.6 (2CH_3 dia.2), 24.3 (2CH_3 dia.1), 24.2 (2CH_3 dia.2). Three quaternary carbon signals are not observed for the minor diastereomer.

HRMS (ESI+) m/z: calcd for $[C_{34}H_{38}B_2O_4+Na]^+$ 555.2854; found 555.2864.

Characterization of minor regioisomer 2f.

^{1}H NMR (500 MHz, $CDCl_3$) δ 8.07 (d, *J* = 8.5 Hz, 1H, CH), 7.89 (d, *J* = 8.1 Hz, 1H, CH), 7.85 (d, *J* = 7.9 Hz, 1H, CH), 7.81 (d, *J* = 7.5 Hz, 1H, CH), 7.73 (d, *J* = 7.4 Hz, 1H, CH), 7.54 – 7.44 (m, 5H, CH), 7.41 – 7.38 (m, 2H, CH), 7.33 – 7.26 (m, 2H, CH), 1.14 (s, 12H, CH), 1.12 (m, 12H, CH).

^{13}C{^{1}H} NMR (126 MHz, $CDCl_3$) δ 149.7 (2C), 141.5 (C), 140.7 (C), 133.9 (C), 133.8 (2CH), 131.0 (C), 130.9 (CH), 129.4 (CH), 128.9 (CH), 128.2 (CH), 127.6 (CH), 127.5 (CH), 126.8 (CH), 126.5 (CH), 126.0 (CH), 125.9 (CH), 125.8 (CH), 125.4 (CH), 83.4 (2C), 83.3 (2C), 24.8 (4CH_3), 24.7 (4CH_3).

HRMS (ESI+) m/z: calcd for $[C_{34}H_{38}B_2O_4+Na]^+$ 555.2854; found 555.2864.

C–C borylation of 1-(2-methoxyphenyl)biphenylene (1g) at 180 °C (Figure 2)

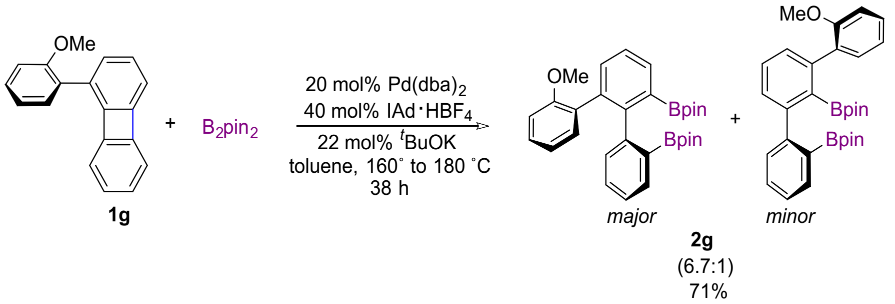

The reaction was conducted according to the general procedure, using 1-(2-methoxyphenyl)biphenylene (104 mg 0.401 mmol), B_2pin_2 (203 mg, 0.799 mmol), $Pd(dba)_2$ (23 mg, 0.040 mmol), 1,3-bis(1-adamantyl)imidazolium tetrafluoroborate (34 mg, 0.080 mmol), KO^{t}Bu (10 mg, 0.090 mmol), and toluene (4 mL). The reaction mixture was stirred at 160 °C for 16 h. The reaction mixture was cooled to room temperature and $Pd(dba)_2$ (23 mg, 0.040 mmol), 1,3-bis(1-adamantyl)imidazolium tetrafluoroborate (34 mg, 0.080 mmol), and KO^{t}Bu (10 mg, 0.086 mmol) were added under flow of argon. The reaction mixture was stirred at 160 °C for a further 6 h and then at 180 °C for an additional 16 h. The crude product was purified by flash column chromatography (silica gel, 6% EtOAc in hexane).

Yield of 2g (major+minor): 71% (0.284 mmol); major:minor regioisomer ratio = 6.7:1 (0.247 mmol:0.037 mmol).

Major regioisomer of 2g: 62% (0.247 mmol). White solid (91 mg, 0.18 mmol, 45%); an additional 17% (0.067 mmol%) could be obtained from the mixed fractions (see below).

Minor regioisomer of 2g: 9% (0.037 mmol). Not isolated as a pure individual compound. Yield determined by ^{1}H NMR of mixed fractions using 1,3,5-trimethoxybenzene or durene as an internal standard.

Mixed fractions: (a) a colourless oil (15 mg) containing 0.0080 mmol of the minor isomer; (b) a colourless oil (54 mg) containing 0.067 mmol of the major isomer and 0.029 mmol of the minor isomer.

Characterization of major regioisomer 2g.

^{1}H NMR (500 MHz, $CDCl_3$) δ 7.61 (dd, J = 6.2, 2.3 Hz, 1H, CH), 7.54 – 7.52 (m, 1H, CH), 7.34 – 7.30 (m, 2H, CH), 7.08 – 7.04 (m, 5H, CH), 6.70 – 6.67 (m, 2H, CH), 3.67 (br s, 3H, CH_3), 1.15 (s, 6H, CH_3), 1.10 (s, 6H, CH_3), 1.05 (s, 6H, CH_3), 1.01 (s, 6H, CH_3).

^{13}C{^{1}H} NMR (126 MHz, $CDCl_3$) δ 156.7 (C), 148.6 (C), 148.1 (C), 137.3 (C), 133.5 (CH), 132.4 (CH), 132.0 (CH), 131.8 (CH), 131.5 (C), 129.3 (CH), 128.5 (CH), 127.8 (CH), 125.5 (CH), 125.1 (CH), 119.6 (CH), 110.1 (CH), 83.1 (4C), 55.2 (CH_3), 25.1 ($2CH_3$), 25.0 ($2CH_3$), 24.6 ($2CH_3$), 24.3 ($2CH_3$).

HRMS (CI+) m/z: calcd for $[C_{31}H_{38}B_2O_5+NH_4]^+$ 530.3249; found 530.3249. Crystals for XRD analysis were grown by slow evaporation of a concentrated methanol solution of **2g** at room temperature

Characterization of minor regioisomer 2g

¹H NMR (500 MHz, $CDCl_3$) δ 7.72 (d, *J* = 7.7 Hz, 1H, CH), 7.67 – 7.65 (m, 1H, CH), 7.50 (dd, *J* = 7.7, 1.7 Hz, 1H, CH), 7.40 (d, *J* = 1.5 Hz, 1H, CH), 7.38 – 7.34 (m, 2H, CH), 7.32 – 7.27 (m, 3H, CH), 7.00 (td, *J* = 7.5, 1.0 Hz, 1H, CH), 6.96 (d, *J* = 8.3 Hz, 1H, CH), 3.77 (s, 3H, CH_3), 1.10 (s, 12H, CH_3), 1.07 (s, 12H, CH_3).

$^{13}C\{^1H\}$ NMR (126 MHz, $CDCl_3$) δ 156.8 (C), 149.7 (C), 149.4 (C), 139.5 (C), 133.5 (2CH), 131.2 (C), 131.1 (CH), 130.4 (CH), 129.3 (CH), 129.2 (CH), 128.6 (CH), 127.2 (CH), 125.8 (CH), 120.9 (CH), 111.7 (CH), 83.2 (4C), 55.8 (CH_3), 24.7 (8CH_3).

HRMS (CI+) m/z: calcd for $[C_{31}H_{38}B_2O_5+NH_4]^+$ 530.3249; found 530.3298.

C–C borylation of 1-(o-tolyl)biphenylene (1h) at 140 °C (Figure 2)

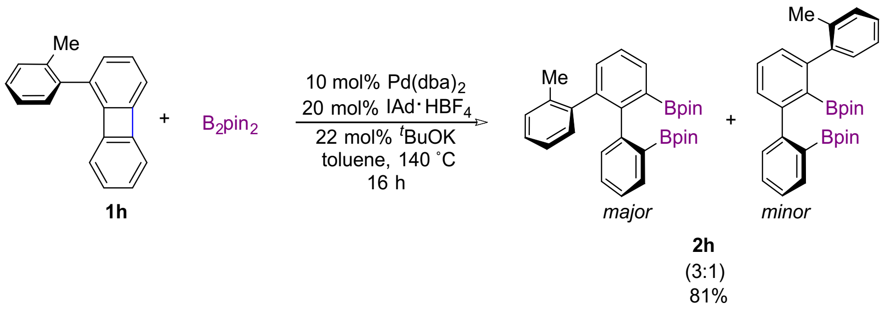

The reaction was conducted according to the general procedure, using 1-(*o*-tolyl)biphenylene, **1h** (97 mg 0.400 mmol), B_2pin_2 (204 mg, 0.803 mmol),), $Pd(dba)_2$ (23 mg, 0.040 mmol), 1,3-bis(1-adamantyl)imidazolium tetrafluoroborate (34 mg, 0.080 mmol, KO^{t}Bu (10 mg, 0.089 mmol), and toluene (4 mL). The reaction mixture was stirred at 140 °C for 16 h. The crude product was purified by flash column chromatography (silica gel, 4% EtOAc in hexane).

Yield of 2h (major+minor): 81% (0.323 mmol); major:minor regioisomer ratio = 3:1 (0.242 mmol:0.0812 mmol).

Major regioisomer of 2h: 61% (0.242 mmol). White solid (108 mg, 0.218 mmol, 55%), a mixture of two diastereomers (dr = 3:1); an additional 6% (0.024 mmol) could be obtained from the mixed fractions (see below).

Minor regioisomer of 2h: 20% (0.0812 mmol). Not isolated as a pure compound. Yield determined by ¹H NMR of mixed fractions using 1,3,5-trimethoxybenzene as an internal standard.

Mixed fractions: (a) a colourless oil (40 mg) containing 0.072 mmol of the minor isomer; (b) a colourless oil (20 mg) containing 0.024 mmol of the major isomer and 0.0092 mmol of the minor isomer.

Characterization of major regioisomer 2h; dia.1 = major diastereomer, dia.2 = minor diastereomer.

¹H NMR (500 MHz, $CDCl_3$) δ 7.63 – 7.56 (m, 2H dia.1, 2H dia.2, CH), 7.33 – 7.29 (m, 1H dia.1, 1H dia.2, CH), 7.25 – 7.23 (m, 1H dia.1, CH), 7.17 – 7.15 (m, 1H dia.2, CH), 7.09 – 7.03 (m, 4H dia.1, 6H dia.2, CH), 6.98 – 6.95 (m, 1H dia.1, CH), 6.94 – 6.92 (m, 1H dia.2, CH), 6.86 – 6.80 (m, 2H dia.1, CH), 2.21 (s, 3H dia.1, CH_3), 1.97 (s, 3H dia.2, CH_3), 1.18 (s, 6H dia.1, CH_3), 1.13 (s, 6H dia.2, CH_3),

1.11 (br s, 6H dia.1, 6H dia.2, CH_3), 1.06 (br s, 6H, dia.1, 6H dia.2, CH_3), 1.01 (s, 6H dia.1, CH_3), 1.00 (s, 6H dia.2, CH_3).

^{13}C{^{1}H} NMR (126 MHz, $CDCl_3$) δ 148.5 (C dia.1), 148.0 (C dia.2), 147.6 (C dia.1), 147.0 (C dia.2), 141.9 (C dia.1), 141.7 (C dia.2), 140.4 (C dia.2), 139.7 (C dia.1), 136.5 (C dia.2), 135.6 (C dia.1), 134.8 (CH dia.2), 133.8 (CH dia.1), 132.3 (CH dia.2), 132.2 (CH dia.1), 131.5 (2CH dia.2), 131.4 (CH dia.1), 131.2 (CH dia.2), 130.6 (CH, dia.1), 129.5 (CH dia.2), 129.3 (CH dia.1), 129.0 (CH dia.1), 128.6 (CH dia.1), 128.5 (CH dia.2), 126.6 (CH dia.2), 126.3 (CH dia.1), 125.7 (CH dia.2), 125.5 (CH dia.1), 125.3 (CH dia.1, CH dia.2), 124.7 (CH dia.1), 124.2 (CH dia.2), 83.3-83.2 (4C dia.1, 4C dia.2), 25.3 (2CH_3 dia.2), 25.1 (2CH_3 dia.1, 2CH3 dia.2), 25.0 (2CH_3 dia.1), 24.6 (2CH_3 dia.1, 2CH_3 dia.2), 24.3 (2CH_3 dia.1), 24.2 (2CH_3 dia.2), 20.7 (CH_3 dia.1), 20.6 (CH_3 dia.2).

HRMS (ESI+) m/z: calcd for $(C_{31}H_{38}B_2O_4+Na)^+$ 519.2854; found 519.2853.

Characterization of minor regioisomer 2h.

^{1}H NMR (500 MHz, CD_2Cl_2) δ 7.69 (d, *J* = 7.6 Hz, 1H, CH), 7.65 (dd, *J* = 7.4, 2.8 Hz, 1H, CH), 7.38 (td, *J* = 7.6, 1.5 Hz, 1H, CH), 7.30 (td, *J* = 7.5, 1.2 Hz, 1H, CH), 7.27 – 7.20 (m, 6H, CH), 7.17 – 7.16 (m, 1H, CH), 2.28 (s, 3H, CH_3), 1.11 (s, 12H, CH_3), 1.07 (s, 12H, CH_3).

^{13}C{^{1}H} NMR (126 MHz, $CDCl_3$) δ 149.7 (C), 149.6 (C), 142.8 (C), 142.2 (C), 135.6 (C), 133.8 (CH), 133.5 (CH), 130.3 (CH), 130.0 (CH), 129.8 (CH), 129.4 (CH), 129.0 (CH), 127.3 (CH), 126.7 (CH), 125.9 (CH), 125.7 (CH), 83.3 (4C), 24.7 (8CH_3), 20.6 (CH_3).

HRMS (ESI+) m/z: calcd for $[C_{31}H_{38}B_2O_4+Na]^+$ 519.2854; found 519.2855.

C–C borylation of 1-(2,6-dimethylphenyl)biphenylene (1i) at 140 °C (Figure 2)

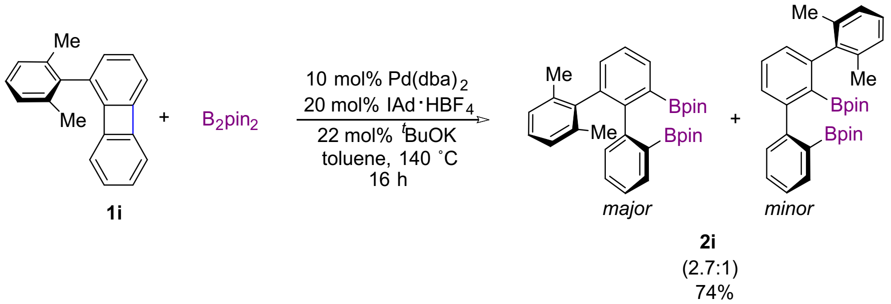

The reaction was conducted according to the general procedure, using 1-(2,6-dimethylphenyl)biphenylene (102 mg, 0.398 mmol), B_2pin_2 (203 mg, 0.799 mmol), Pd(dba)$_2$ (24 mg, 0.041 mmol), 1,3-bis(1-adamantyl)imidazolium tetrafluoroborate (34 mg, 0.080 mmol), KO^{t}Bu (10 mg, 0.087 mmol), and toluene (4 mL). The reaction mixture was stirred at 140 °C for 16 h. The crude product was purified by flash column chromatography (silica gel, 5% EtOAc in hexane).

Yield of 2i (major+minor): 74% (150 mg, 0.294 mmol, pale-yellow solid); major:minor regioisomer ratio = 2.7:1.

^{1}H NMR (500 MHz, CD_2Cl_2) δ 7.68 (d, *J* = 7.5 Hz, 1H reg. 1, CH), 7.67 – 7.61 (m, 1H reg. 1, 2H reg. 2, CH), 7.38 (td, *J* = 7.5, 1.5 Hz, 1H reg. 1, CH), 7.35 (t, *J* = 7.5 Hz, 1H reg. 2, CH), 7.29 (td, *J* = 7.4, 1.9 Hz, 1H reg. 1, CH), 7.24 – 7.23 (m, 1H reg. 1, CH), 7.13 – 7.07 (m, 4H reg. 1, 2H reg. 2, CH), 7.03

(td, J = 7.5, 1.6 Hz, 1H reg. 2, CH), 7.00 – 6.99 (m, 1H reg. 1, 1H reg. 2, CH), 6.96 (t, J = 7.5 Hz, 1H reg. 2, CH), 6.88 (d, J = 7.4 Hz, 1H reg. 2, CH), 6.76 (d, J = 7.3 Hz, 1H reg. 2, CH), 2.10 (s, 3H reg. 2, CH_3), 2.08 (s, 6H reg. 1, CH_3), 1.84 (s, 3H reg. 2, CH_3), 1.14 (s, 6H reg. 2, CH_3), 1.12 (s, 6H reg. 2, CH_3), 1.09 (s, 12H reg. 1, CH_3), 1.08 (s, 12H reg. 1, CH_3), 1.06 (s, 6H reg. 2, CH_3), 0.99 (s, 6H reg. 2, CH_3).

^{13}C{^{1}H} NMR (126 MHz, $CDCl_3$) δ 150.1 (C reg. 1), 150.0 (C reg. 1), 148.1 (C reg. 2), 146.4 (C reg. 2), 142.2 (2C reg. 1), 141.4 (C reg. 2), 139.0 (C reg. 2), 136.9 (C reg. 2), 136.6 (C reg. 2), 136.1 (C reg. 1), 134.9 (CH un), 134.0 (CH reg. 1), 133.7 (CH reg. 1), 132.5 (CH un), 131.2 (CH un), 129.5 (CH reg. 1), 129.4 (CH reg. 1), 128.8 (CH reg. 1), 128.7 (2CH un), 127.2 (CH un), 127.0 (CH reg. 1), 126.9 (CH un), 126.6 (CH un), 126.5 (CH reg. 1, CH un), 126.1 (CH, un), 125.9 (CH reg. 1), 125.4 (CH un), 83.3 (4C reg. 1), 83.2 (4C reg. 2), 25.2 (CH_3 un), 24.9 (CH_3 un), 24.7 (CH_3 un), 24.1 (CH_3 un), 21.6 (CH_3 reg. 2), 21.2 (CH_3 reg. 2), 21.0 (CH_3 reg. 1). Three quaternary carbon signals are not observed for reg. 1. Of the 11 unassigned CH signals, 10 correspond to reg. 2 and one corresponds to two reg. 1 CHs.

HRMS (ESI+) m/z: calcd for $[C_{32}H_{40}B_2O_4+Na]^+$ 533.3010; found 533.3019.

C–C borylation of 1-(2-*iso*propylphenyl)biphenylene (1j) at 140 °C (Figure 2)

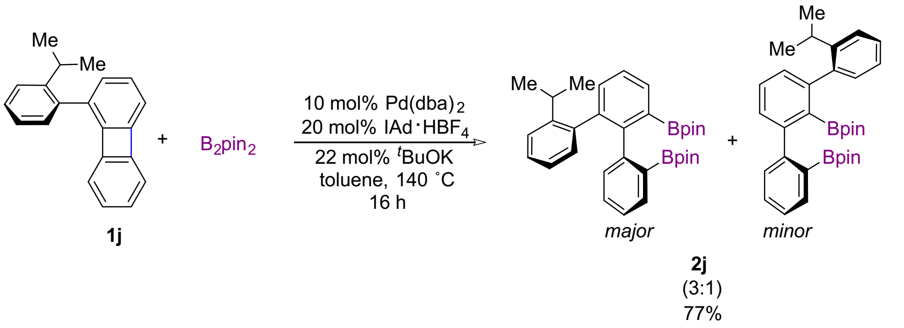

The reaction was conducted according to the general procedure, using 1-(2-*iso*propylphenyl)biphenylene, **1j** (108 mg, 0.399 mmol), B_2pin_2 (204 mg, 0.803 mmol), KO^{t}Bu (10 mg, 0.089 mmol), 1,3-bis(1-adamantyl)imidazolium tetrafluoroborate (34 mg, 0.080 mmol), Pd(dba)$_2$ (23 mg, 0.040 mmol), and toluene (4 mL). The reaction mixture was stirred at 140 °C for 16 h. The crude product was purified by flash column chromatography (silica gel, 4-6% EtOAc in hexane).

Yield of 2j (major+minor): 77% (0.309 mmol); major:minor regioisomer ratio = 3:1 (0.231 mmol:0.0775 mmol).

Major regioisomer of 2j: 58% (0.231 mol). White solid (54 mg, 0.100 mmol, 25%), a mixture of two diastereomers (dr = 1.8:1, based on integrals of 7.64 ppm and 6.8ppm); an additional 33% (0.131 mmol) could be obtained from the mixed fractions (see below).

Minor regioisomer of 2j: 19% (0.0775 mmol). Not isolated as a pure individual compound. Yield determined by ^{1}H NMR of mixed fractions using1,3,5-trimethoxybenzene as an internal standard.

Mixed fractions: (a) a colourless oil (12 mg) containing 0.016 mmol of the minor isomer; (b) a colourless oil (101 mg) containing 0.131 mmol of the major isomer and 0.0615 mmol of the minor isomer.

Characterization of major regioisomer 2j; dia.1 = major diastereomer, dia.2 = minor diastereomer, un = unassigned.

^{1}H NMR (500 MHz, $CDCl_3$) δ 7.64 (dd, J = 7.5, 2.8 Hz, 0.35H dia.2, CH), 7.61 – 7.56 (m, 1.52H un, CH), 7.33 – 7.28 (m, 0.97H un, CH), 7.25 – 7.22 (m, 1.25H un, CH), 7.18 – 7.14 (m, 1.35H un, CH), 7.11 – 7.00 (m, 4H un, CH), 6.94 – 6.89 (m, 1H un, CH), 6.80 (td, J = 7.5, 3.2 Hz, 0.62H dia.1, CH), 3.04 – 2.96 (m, 1H dia.1 and dia.2, CH), 1.19 – 1.17 (m, 11.9H un, CH_3), 1.11 – 1.089 (m, 5.6H un, CH_3), 1.05 (s, 3.5H un, CH_3), 1.00 (s, 5.9H un, CH_3), 0.87 (d, J = 6.8 Hz, 1.1H un, CH_3), 0.51 (d, J = 6.8 Hz, 1.1H un, CH_3).

^{13}C{^{1}H} NMR (126 MHz, $CDCl_3$) δ 148.5 (C dia.1), 147.9 (C dia.2), 147.7 (C dia.1), 147.1 (C dia.2), 146.7 (C dia.2), 146.5 (C, dia.1), 140.7 (C dia.2), 140.6 (C dia.1), 140.5 (C dia.2), 139.7 (C dia.1), 135.0 (CH dia.2), 133.9 (CH dia.1), 132.6 (CH dia.2), 132.1 (CH dia.1), 131.9 (CH dia.2), 131.6 (CH dia.2), 131.5 (CH, dia.1), 130.6 (CH dia.1, CH dia.2), 129.1 (CH dia.1), 128.9 (CH dia.1), 128.6 (CH dia.2), 127.2 (CH dia.2), 126.8 (CH dia.1), 125.7 (CH dia.2), 125.3 (2CH dia.1, CH dia.2), 124.9 (CH dia.2), 124.8 (CH dia.1), 124.3 (CH dia.1, CH dia.2), 83.3 (C un), 83.2 (C un), 29.9 (CH dia.1), 28.8 (CH dia.2), 25.7 (CH_3 un), 25.5 (CH_3 un), 25.2 (CH_3 un), 25.1 (CH_3 un), 25.0 (CH_3 un), 24.6 (CH_3 un), 24.2 (CH_3 un), 24.1 (CH_3 un), 23.3 (CH_3 un), 22.6 (CH_3 un).

HRMS (ESI+) m/z: calcd for $[C_{33}H_{42}B_2O_4+Na]^+$ 547.3167; found 547.3178.

Characterization of minor regioisomer 2j.

^{1}H NMR (500 MHz, $CDCl_3$) δ 7.70 – 7.68 (m, 2H, CH), 7.40 – 7.36 (m, 2H, CH), 7.33 – 7.27 (m, 3H, CH), 7.24 (dd, *J* = 7.7, 1.6 Hz, 1H, CH), 7.21 – 7.20 (m, 2H, CH), 7.16 – 7.15 (m, 1H, CH), 3.18 (sept, *J* = 6.9 Hz, 1H, CH), 1.15 (d, *J* = 6.9 Hz, 6H, CH_3), 1.10 (s, 12H, CH_3), 1.09 (s, 12H, CH_3).

^{13}C{^{1}H} NMR (126 MHz, $CDCl_3$) δ 149.8 (C), 149.6 (C), 146.5 (C), 143.0 (C), 141.5 (C), 133.8 (CH), 133.3 (CH), 130.0 (CH), 129.9 (CH), 129.4 (CH), 129.0 (CH), 127.6 (CH), 126.8 (CH), 125.9 (CH), 125.5 (CH), 125.3 (CH), 83.3 (2C), 83.2 (2C), 29.3 (CH), 24.8 (4CH_3), 24.7 (4CH_3), 24.5 (2CH_3).

HRMS (ESI+) m/z: calcd for $[C_{33}H_{42}B_2O_4+Na]^+$ 547.3167; found 547.3179.

C–C borylation of 1-(4-methoxyphenyl)biphenylene (1k) at 140 °C (Figure 2)

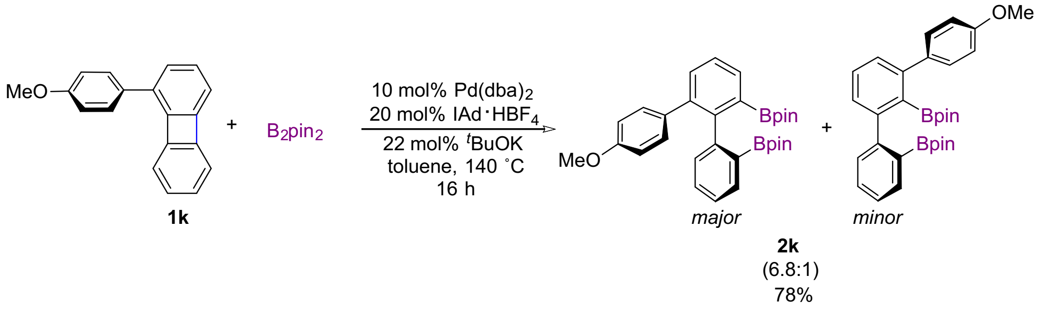

The reaction was conducted according to the general procedure, using 1-(4-methoxyphenyl)biphenylene (103.5 mg 0.4007 mmol), B_2pin_2 (205 mg, 0.807 mmol), $Pd(dba)_2$ (23.0 mg, 0.0400 mmol), 1,3-bis(1-adamantyl)imidazolium tetrafluoroborate (33.9 mg, 0.0799 mmol), KO^tBu (10.2 mg, 0.0909 mmol), and toluene (4 mL). The reaction mixture was stirred at 140 °C for 16 h. The crude product was purified by flash column chromatography (silica gel, 6-10% EtOAc in hexane).

Yield of 2k (major+minor): 78% (0.311 mmol); major:minor regioisomer ratio = 6.8:1 (0.271 mmol: 0.040 mmol).

Major regioisomer 2k: 68% (139 mg, 0.271 mmol). White solid.

Minor regioisomer 2k: 10% (0.040 mmol). Not isolated as a pure individual compound. Yield determined by ^{1}H NMR of a pale-yellow (33 mg) using durene as an internal standard.

Characterization of major regioisomer 2k.

^{1}H NMR (400 MHz, $CDCl_3$) δ 7.62 – 7.60 (m, 1H, CH), 7.57 (dd, J = 6.8, 2.1 Hz, 1H, CH), 7.37 – 7.31 (m, 2H, CH), 7.14 – 7.12 (m, 2H, CH), 7.09 -7.07 (m, 2H, CH), 6.93 – 6.91 (m, 1H, CH), 6.67 – 6.65 (m, 2H, CH), 3.72 (s, 3H, CH_3), 1.14 (s, 6H, CH_3), 1.07 (s, 6H, CH_3), 1.06 (s, 6H, CH_3), 1.01 (s, 6H, CH_3).

$^{13}C\{^1H\}$ NMR (126 MHz, $CDCl_3$) δ 157.9 (C), 148.6 (C), 147.4 (C), 140.2 (C), 135.0 (C), 133.8 (CH), 132.0 (CH), 131.4 (CH), 131.1 (2CH), 130.4 (CH), 129.0 (CH), 126.1 (CH), 125.3 (CH), 121.8 (2CH), 83.2 (4C), 55.1 (CH_3), 25.1 ($2CH_3$), 24.9 ($2CH_3$), 24.6 ($2CH_3$), 24.3 ($2CH_3$).

HRMS (ESI+) m/z: calcd for $[C_{31}H_{38}B_2O_5+Na]^+$ 535.2803; found 535.2793.

Characterization of minor regioisomer 2k.

^{1}H NMR (500 MHz, $CDCl_3$) δ 7.73 (d, J = 7.7 Hz, 1H, CH), 7.69 – 7.68 (m, 1H, CH), 7.61 – 7.58 (m, 2H, CH), 7.52 – 7.50 (m, 1H, CH), 7.48 – 7.47 (m, 1H, CH), 7.41 – 7.37 (m, 1H, CH), 7.32 – 7.30 (m, 2H, CH), 7.97 – 7.95 (m, 2H, CH), 3.84 (s, 3H, CH_3), 1.11 (s, 12H, CH_3), 1.06 (s, 12H, CH_3).

$^{13}C\{^1H\}$ NMR (100 MHz, $CDCl_3$) δ 159.3 (C), 150.1 (C), 149.5 (C), 141.3 (C), 134.5 (CH), 133.9 (C), 133.7 (CH), 129.3 (CH), 129.2 (CH), 128.3 (2CH), 127.8 (CH), 125.9 (CH), 124.0 (CH), 114.2 (2CH), 83.3 (4C), 55.4 (CH_3), 24.7 ($8CH_3$).

HRMS (ESI+) m/z: calcd for $[C_{31}H_{38}B_2O_5+Na]^+$ 535.2803; found 535.2799.

C–C borylation of 1-(4-(dimethylamino)phenyl)biphenylene (1l) at 140 °C (Figure 2)

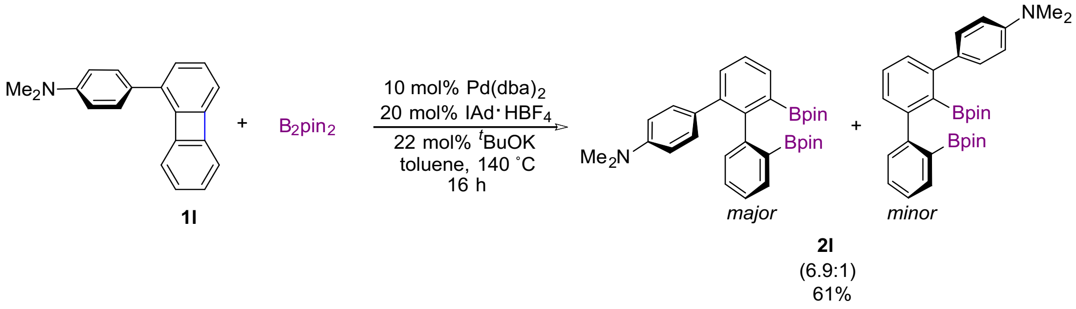

The reaction was conducted according to the general procedure, using 1-(4-(dimethylamino)phenyl)biphenylene, **1l** (108 mg 0.398 mmol), B_2pin_2 (204 mg, 0.803 mmol), KO^tBu (10 mg, 0.088 mmol), 1,3-bis(1-adamantyl)imidazolium tetrafluoroborate (34 mg, 0.079 mmol), $Pd(dba)_2$ (23 mg, 0.040 mmol), and toluene (4 mL). The reaction mixture was stirred at 140 °C for 16 h. The crude product was purified by flash column chromatography (silica gel, 8% EtOAc in hexane).

Yield of 2l (major+minor): 61% (0.244 mmol); major:minor regioisomer ratio = 6.9:1 (0.213 mmol:0.031 mmol).

Major regioisomer 2l: White solid (87 mg, 0.170 mmol, 43%); an additional 11% (0.043 mmol) could be obtained from mixed fractions (see below).

Minor regioisomer 2l: 7% (0.031 mmol). Not isolated as a pure compound. Yield determined by ^{1}H NMR of mixed fractions using 1,3,5-trimethoxybenzene as an internal standard.

Mixed fractions: (a) pale-yellow solid (22 mg) containing 0.012 mmol of the minor isomer; (b) colourless oil (40 mg) containing 0.043 mmol of the major isomer and 0.019 mmol of the minor isomer.

Characterization of major regioisomer 2l.

^{1}H NMR (500 MHz, $CDCl_3$) δ 7.65 – 7.63 (m, 1H, CH), 7.55 (dd, J = 7.3, 1.4 Hz, 1H, CH), 7.38 (dd, J = 7.8, 1.4 Hz, 1H, CH), 7.32 (t, J = 7.5 Hz, 1H, CH), 7.17 – 7.12 (m, 2H, CH), 7.06 – 7.04 (m, 2H, CH), 6.97 – 6.95 (m, 1H, CH), 6.53 – 6.51 (m, 2H, CH), 2.86 (s, 6H, CH_3), 1.15 (s, 6H, CH_3), 1.09 (s, 6H, CH_3), 1.07 (s, 6H, CH_3), 1.03 (s, 6H, CH_3).

$^{13}C\{^1H\}$ NMR (126 MHz, $CDCl_3$) δ 149.0 (C), 148.8 (C), 147.3 (C), 140.5 (C), 133.8 (CH), 131.6 (CH), 131.5 (CH), 130.9 (C), 130.7 (2CH), 130.4 (CH), 129.1 (CH), 126.0 (CH), 125.1 (CH), 111.8 (2CH), 83.1 (4C), 40.7 ($2CH_3$), 25.1 ($2CH_3$), 25.0 ($2CH_3$), 24.6 ($2CH_3$), 24.3 ($2CH_3$).

HRMS (ESI+) m/z: calcd for $[C_{32}H_{41}B_2O_4N+H]^+$ 526.3300; found 526.3315.

Characterization of minor regioisomer 2l.

^{1}H NMR (500 MHz, $CDCl_3$) δ 7.71 (d, J = 7.7 Hz, 1H, CH), 7.67 – 7.65 (m, 1H, CH), 7.58 – 7.56 (m, 2H, CH), 7.51 (dd, J = 7.9, 1.7 Hz, 1H, CH), 7.48 – 7.47 (m, 1H, CH), 7.39 – 7.35 (m, 1H, CH), 7.31 – 7.29 (m, 2H, CH), 6.79 – 6.77 (m, 2H, CH), 2.98 (s, 6H, CH_3), 1.10 (s, 12H, CH_3), 1.06 (s, 12H, CH_3),

$^{13}C\{^1H\}$ NMR (126 MHz, $CDCl_3$) δ 150.2 (C), 150.1 (C), 149.8 (C), 141.7 (C), 134.5 (CH), 133.5 (CH), 129.3 (C), 129.3 (CH), 129.2 (CH), 127.9 (2CH), 127.2 (CH), 125.8 (CH), 123.5 (CH), 112.8 (2CH), 83.3 (2C), 83.1 (2C), 40.7 ($2CH_3$), 25.15 ($8CH_3$).

HRMS (ESI+) m/z: calcd for $[C_{32}H_{41}B_2O_4N+H]^+$ 526.3300; found 526.3315.

C–C borylation of 1-(*p*-tolyl)biphenylene (1m) at 140 °C (Figure 2)

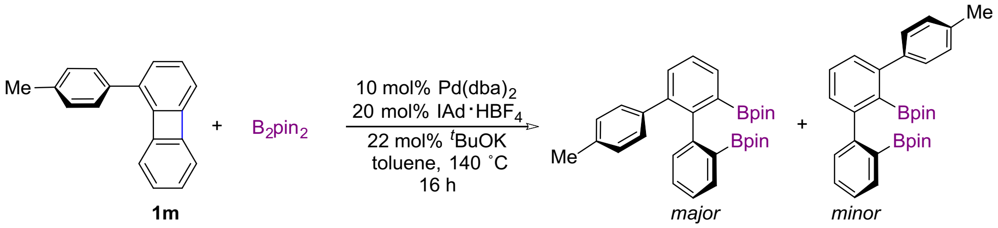

2m
(5.1:1)
78%

The reaction was conducted according to the general procedure, using 1-(*p*-tolyl)biphenylene, **1m** (97 mg, 0.40 mmol), B_2pin_2 (204 mg, 0.803 mmol), $Pd(dba)_2$ (23 mg, 0.040 mmol), 1,3-bis(1-adamantyl)imidazolium tetrafluoroborate (34 mg, 0.081 mmol), KO^{t}Bu (10 mg, 0.089 mmol), and toluene (4 mL). The reaction mixture was stirred at 140 °C for 16 h. The crude product was purified by flash column chromatography (silica gel, 4% EtOAc in hexane).

Yield of 2m (major+minor): 78% (0.313 mmol); major:minor regioisomer ratio = 5.1:1 (0.262 mmol:0.0512 mmol).

Major regioisomer 2m: 65% (0.262 mmol). White solid (124 mg, 0.250 mmol, 62%); an additional 3% (0.012 mmol) could be obtained from the mixed fractions (see below).

Minor regioisomer of 2m: 13% (0.0512 mmol). Not isolated as a pure compound. Yield determined by ^{1}H NMR of mixed fractions using 1,3,5-trimethoxybenzene as an internal standard.

Mixed fractions: (a) colourless oil (11 mg) containing 0.012 mmol of the major isomer and 0.0032 mmol of the minor isomer; (b) colourless gum (27 mg) containing 0.048 mmol of the minor isomer.

Characterization of major regioisomer 2m.

^{1}H NMR (500 MHz, $CDCl_3$) δ 7.64 – 7.59 (m, 2H, CH), 7.38 – 7.33 (m, 2H, CH), 7.15 – 7.13 (m, 2H, CH), 7.08 (d, J = 7.9 Hz, 2H, CH), 6.96 – 6.93 (m, 3H, CH), 2.24 (s, 3H, CH3), 1.15 (s, 6H, CH3), 1.09 (s, 6H, CH3), 1.07 (s, 6H, CH_3), 1.03 (s, 6H, CH_3).

^{13}C{^{1}H} NMR (126 MHz, $CDCl_3$) δ 148.6 (C), 147.4 (C), 140.5 (C), 139.6 (C), 135.3 (C), 133.8 (CH), 132.1 (CH), 131.4 (CH), 130.4 (CH), 129.9 (2CH), 129.0 (CH), 128.1 (2CH), 126.0 (CH), 125.2 (CH), 83.2 (2C), 83.1 (2C), 25.1 (2CH_3), 25.0 (2CH_3), 24.6 (2CH_3), 24.4 (2CH_3), 21.2 (CH_3).

HRMS (ESI+) m/z: calcd for $(C_{31}H_{38}B_2O_4+Na)^+$ 519.2854; found 519.2861.

Characterization of minor regioisomer 2m.

^{1}H NMR (500 MHz, $CDCl_3$) δ 7.75 (d, J = 7.7 Hz, 1H, CH), 7.69 (d, J = 7.2 Hz, 1H, CH), 7.57 – 7.51 (m, 4H, CH), 7.41 – 7.38 (m, 1H, CH), 7.33 (m, 2H, CH), 7.24 (d, 7.9 Hz, 2H, CH), 2.39 (s, 3H, CH_3), 1.15 (s, 12H, CH_3), 1.07 (s, 12H, CH_3).

^{13}C{^{1}H} NMR (126 MHz, $CDCl_3$) δ 150.1 (C), 149.6 (C), 141.7 (C), 138.5 (C), 137.2 (C), 134.4 (CH), 133.7 (CH), 129.5 (2CH), 129.3 (2CH), 128.0 (CH), 127.2 (2CH), 125.9 (CH), 124.3 (CH), 83.3 (4C), 24.7 (8CH_3), 21.2 (CH_3).

HRMS (ESI+) m/z: calcd for $[C_{31}H_{38}B_2O_4+Na]^+$ 519.2854; found 519.2865.

C–C borylation of 1-(4-(trifluoromethyl)phenyl)biphenylene (1n) at 140 °C (Figure 2)

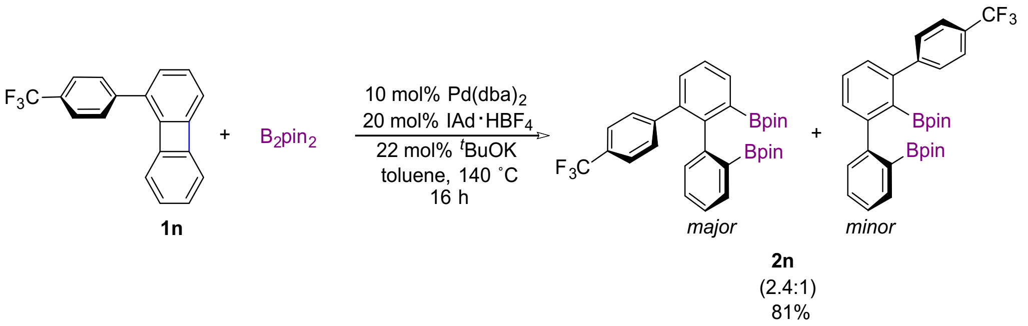

The reaction was conducted according to the general procedure, using 1-(4-trifluoromethyl)phenylbiphenylene (118 mg, 0.397 mmol), B_2pin_2 (206 mg, 0.811 mmol), KO^{t}Bu (10 mg, 0.089 mmol), 1,3-bis(1-adamantylimidazolium)tetra fluoroborate (34 mg, 0.081 mmol), $Pd(dba)_2$ (23 mg, 0.040 mmol), and toluene (4 mL). The reaction mixture was stirred at 140 °C for 16 h. The crude product was purified by flash column chromatography (silica gel, 5% EtOAc in hexane).

Yield of 2n (major+minor): 81% (0.319 mmol); major:minor regioisomer ratio = 2.4:1 (0.228 mmol:0.094 mmol).

Major regioisomer of 2n: 57%. White solid (101 mg, 0.184 mmol, 46%); an additional 11% (0.044 mmol) could be obtained from mixed fractions (see below).

Minor regioisomer of 2n: 24% (0.094 mmol). Not isolated as a pure compound. Yield determined by ^{1}H NMR of mixed fractions using 1,3,5-trimethoxybenzene as an internal standard.

Mixed fractions: (a) colourless oil (56 mg) containing 0.044 mmol of the major isomer and 0.050 mmol of the minor isomer; (b) pale-yellow oil (24 mg) containing 0.044 mmol of the minor isomer.

Characterization of major regioisomer 2n.

^{1}H NMR (400 MHz, $CDCl_3$) δ 7.65 – 7.61 (m, 2H, CH), 7.40 – 7.28 (m, 6H, CH), 7.15 – 7.13 (m, 2H, CH), 6.91 (m, 1H, CH), 1.15 (s, 6H, CH_3), 1.08 (s, 6H, CH_3), 1.06 (s, 6H, CH_3), 1.02 (s, 6H, CH_3).

^{19}F NMR (377 MHz, $CDCl_3$) δ -62.32.

^{13}C{^{1}H} NMR (100 MHz, $CDCl_3$) δ 147.8 (C), 147.4 (C), 146.3 (C), 139.3 (C), 134.0 (CH), 133.0 (CH), 131.1 (CH), 130.3 (3CH), 129.2 (CH), 128.1 (q, J = 32.0 Hz, C), 126.3 (CH), 125.7 (CH), 124.3 (q, J = 3.8 Hz, 2CH), 124.5 (q, J = 272.0 Hz, CF_3), 83.4 (2C), 83.3 (2C), 25.1 ($2CH_3$), 24.9 ($2CH_3$), 24.5 ($2CH_3$), 24.4 ($2CH_3$).

HRMS (ESI+) m/z: calcd for $[C_{31}H_{35}B_2O_4F_3+Na]^+$ 573.2571; found 573.2579.

Characterization of minor regioisomer 2n.

^{1}H NMR (500 MHz, $CDCl_3$) δ 7.79 – 7.74 (m, 3H, CH), 7.72 – 7.66 (m, 3H, CH), 7.56 – 7.53 (m, 2H, CH), 7.42 – 7.39 (m, 1H, CH), 7.34 (m, 2H, CH), 1.11 (s, 12H, CH_3), 1.06 (s, 12H, CH_3).

^{19}F NMR (377 MHz, $CDCl_3$) δ -62.38.

^{13}C{^{1}H} NMR (126 MHz, $CDCl_3$) δ 150.4 (C), 149.2 (C), 145.0 (C), 140.1 (C), 134.5 (CH), 133.9 (CH), 129.4 (CH), 129.4 (q, J = 32.6 Hz, C), 129.2 (CH), 128.3 (CH), 127.6 (2CH), 126.1 (CH), 125.7 (q, J = 3.7 Hz, 2CH), 124.5 (CH), 124.5 (q, J = 271.6 Hz, CF_3), 83.5 (2C), 83.3 (2C), 24.7 ($8CH_3$).

HRMS (ESI+) m/z: calcd for $[C_{31}H_{35}B_2O_4F_3+Na]^+$ 573.2571; found 573.2577.

C–C borylation of *tert*-butyl-3-(biphenylen-1-yl)benzoate (1o) at 140 °C (Figure 2)

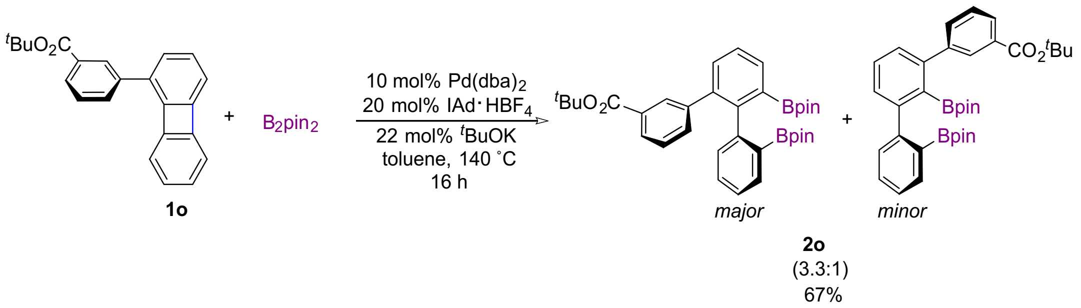

The reaction was conducted according to the general procedure, using *tert*-butyl-3-(biphenylen-1-yl)benzoate, **1o** (131 mg 0.399 mmol), B_2pin_2 (203 mg, 0.799 mmol), KO^{t}Bu (10 mg, 0.089 mmol), 1,3-bis(1-adamantyl)imidazolium tetrafluoroborate (34 mg, 0.080 mmol), $Pd(dba)_2$ (23 mg, 0.040 mmol), and toluene (4 mL). The reaction mixture was stirred at 140 °C for 16 h. The crude product was purified by flash column chromatography (silica gel, 6% EtOAc in hexane).

Yield of 2o (major+minor): 67% (0.269 mmol); a major:minor regioisomer ratio = 3.3:1 (0.207 mmol:0.062 mmol).

Major regioisomer of 2o: 52%. White solid (103 mg, 0.177 mmol, 44%); an additional 8% (0.030 mmol) could be obtained from the mixed fractions (see below).

Minor regioisomer of 2o: 15% (0.062 mmol). Not isolated as a pure compound. Yield determined by ^{1}H NMR of mixed fractions using 1,3,5-trimethoxybenzene as an internal standard.

Mixed fractions: (a) a pale-yellow oil (48 mg) containing 0.030 mmol of the major isomer and 0.038 mmol of the minor isomer; (b) yellow oil (26 mg) containing 0.024 mmol of the minor isomer.

Characterization of major regioisomer 2o.

^{1}H NMR (500 MHz, $CDCl_3$) δ 7.81 (br. s, 1H, CH), 7.72 (d, J = 7.8 Hz, 1H, CH), 7.63 (dd, J = 6.6, 1.8 Hz, 1H, CH), 7.59 (d, J = 6.7 Hz, 1H, CH) , 7.40 – 7.35 (m, 2H, CH), 7.28 (d, J = 7.7 Hz, 1H, CH), 7.16 – 7.09 (m, 3H, CH), 6.97 (d, J = 3.7 Hz, 1H, CH), 1.54 (s, 9H, CH_3), 1.14 (s, 6H, CH_3), 1.05 (s, 6H, CH_3), 1.04 (s, 6H, CH_3), 1.01 (s, 6H, CH_3).

^{13}C{^{1}H} NMR (126 MHz, $CDCl_3$) δ 166.1 (C), 148.1 (C), 147.5 (C), 142.5 (C), 139.9 (C), 134.3 (CH), 134.0 (CH), 132.6 (CH), 131.2 (CH), 131.1 (CH), 130.5 (CH), 129.1 (CH), 127.1 (2CH), 126.2 (CH), 125.5 (CH), 83.3 (2C), 83.1 (2C), 80.7 (C), 28.3 ($3CH_3$), 25.1 ($2CH_3$), 24.9 ($2CH_3$), 24.5 ($2CH_3$), 24.4 ($2CH_3$). Three quaternary carbon signals are not observed.

HRMS (ESI+) m/z: calcd for $[C_{35}H_{44}B_2O_6+Na]^+$ 605.3222; found 605.3232.

Characterization of minor regioisomer 2o.

^{1}H NMR (500 MHz, $CDCl_3$) δ 8.26 (br. S, 1H, CH), 7.95 (d, J = 7.8 Hz, 1H, CH), 7.80 (d, J = 8.1 Hz, 1H, CH), 7.77 (d, J = 7.7 Hz, 1H, CH), 7.70 – 7.69 (m, 1H, CH), 7.57 (dd, J = 7.8, 1.7 Hz, 1H, CH), 7.53 – 7.52 (m, 1H, CH), 7.46 (t, J = 7.7 Hz, 1H, CH), 7.40 (td, J = 7.4, 1.4 Hz, 1H, CH), 7.33 – 7.29 (m, 2H, CH), 1.61 (s, 9H, CH_3), 1.10 (s, 12H, CH_3), 1.06 (s, 12H, CH_3).

^{13}C{^{1}H} NMR (126 MHz, $CDCl_3$) δ 166.0 (C), 150.3 (C), 149.4 (C), 141.5 (C), 140.9 (C), 134.5 (CH), 133.8 (CH), 132.6 (C), 131.3 (CH), 129.3 (2CH), 128.7 (CH), 128.4 (CH), 128.2 (2CH), 126.0 (CH), 124.5 (CH), 83.4 (2C), 83.3 (2C), 81.3 (C), 28.4 ($3CH_3$), 25.2 ($4CH_3$), 24.7 ($4CH_3$).

HRMS (ESI+) m/z: calcd for $[C_{35}H_{44}B_2O_6+Na]^+$ 605.3222; found 605.3231.

C–C borylation of 1-(trimethylsilyl)biphenylene (1p) at 180 °C (Figure 2)

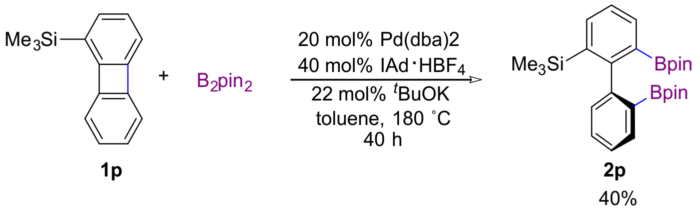

The reaction was conducted according to the general procedure, using 1-(trimethylsilyl)biphenylene, **1p** (88.0 mg, 0.392 mmol), B_2pin_2 (200 mg, 0.788 mmol), $Pd(dba)_2$ (22.6 mg, 0.0393 mmol), 1,3-bis(1-adamantyl)imidazolium tetrafluoroborate (33.1 mg, 0.0780 mmol), KO^{t}Bu (9.4 mg, 0.086 mmol), and toluene (3.9 mL). The reaction mixture was stirred at 180 °C for 16 h, cooled to room temperature and KO^{t}Bu (9.8 mg, 0.086 mmol), 1,3-bis(1-adamantyl)imidazolium tetrafluoroborate (33.4 mg, 0.0787 mmol), and $Pd(dba)_2$ (22.6 mg, 0.0393 mmol) were added under argon atmosphere. The reaction mixture was stirred at 180 °C for further 24 h. The crude product was purified by flash column chromatography (silica gel, 0-20% EtOAc in hexane).

Yield of 2p: 40%. Pale-yellow oil (75 mg, 0.157 mmol). Only the major isomer was isolated.

^{1}H NMR (500 MHz, $CDCl_3$) δ 7.67 (d, J = 7.3 Hz, 1H, CH), 7.58 (d, J = 7.7 Hz, 2H, CH), 7.34 (td, J = 7.5, 1.5 Hz, 1H, CH), 7.29 – 7.24 (m, 2H, CH), 7.20 (d, J = 7.5 Hz, 1H, CH), 1.05 (s, 6H, CH_3), 1.02 (s, 12H, CH_3), 0.98 (s, 6H, CH_3), -0.10 (s, 9H, CH_3).

^{13}C{^{1}H} NMR (126 MHz, $CDCl_3$) δ 155.0 (C), 150.4 (C), 137.8 (C), 135.4 (CH), 133.6 (CH), 133.5 (CH), 130.4 (CH), 128.8 (CH), 125.9 (CH), 124.9 (CH), 83.0 (2C), 82.9 (2C), 24.8 ($2CH_3$), 24.6 ($4CH_3$), 24.4 ($2CH_3$), 0.7 ($3CH_3$).

HR-MS (ESI+) m/z: calcd for $[C_{27}H_{40}B_2O_4+Na]^+$ 501.2780; found 501.2779.

C–C borylation of 1-Bpinbiphenylene (1q) at 140 °C (Figure 2)

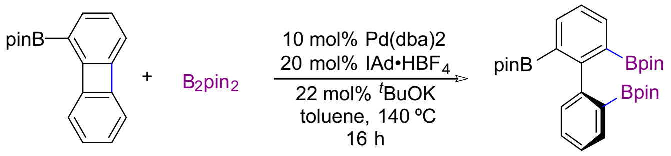

1q **2q** 56%

The reaction was conducted according to the general procedure using 1- Bpinbiphenylene, **1q** (111 mg, 0.400 mmol), B_2pin_2 (204 mg, 0.803 mmol), $Pd(dba)_2$ (23 mg, 0.039 mmol), 1,3-bis(1-adamantyl)imidazolium tetrafluoroborate (34 mg, 0.080 mmol), KO^{t}Bu (10 mg, 0.091 mmol), and toluene (4 mL). The reaction mixture was stirred at 140 °C for 16 h. The crude product was purified by flash column chromatography (silica gel, 3-8% EtOAc in hexane).

Yield of 2q: 56% (0.223 mmol). White solid (44 mg, 0.083 mmol, 21%); an additional 35% (0.014 mmol) of the major isomer was obtained from the mixed fractions (see below).

2,2'-Bis(pinacolatoboron)biphenyl: 13%. White solid (13 mg, 0.032 mmol, 8%). An additional 5% of the side product was obtained from mixed fraction (see below).

Mixed fraction: Colourless oil (84 mg) consisting of **2q** (0.14 mmol) and 2,2′-bis(pinacolatoboron)biphenyl (0.019 mmol).

Characterization of 2q.

^{1}H NMR (500 MHz, $CDCl_3$) δ 7.66 (d, J = 7.4 Hz, 2H, CH), 7.64 (d, J = 7.2 Hz, 1H, CH), 7.29 – 7.21 (m, 3H, CH), 7.14 (d, J = 7.4 Hz, 1H, CH), 1.03 (s, 12H, CH_3), 1.02 (s, 12H, CH_3), 1.01 (s, 12H, CH_3).

^{13}C{^{1}H} NMR (126 MHz, $CDCl_3$) δ 154.9 (C), 150.6 (C), 134.9 (2CH), 133.1 (CH), 129.9 (CH), 128.4 (CH), 125.2 (CH), 124.8 (CH), 83.0 (4C), 82.7 (2C), 24.7 ($4CH_3$), 24.6 ($4CH_3$), 24.4 ($4CH_3$). Three quaternary carbon signals are not observed.

HRMS (ESI+) m/z: calcd for $[C_{30}H_{43}B_3O_6+K]^+$ 571.2976; found 571.2992

Characterization of 2,2'-bis(pinacolatoboron)biphenyl.

^{1}H NMR (500 MHz, $CDCl_3$) δ 7.66 (d, J = 2.7 Hz, 2H, CH), 7.38 (td, J = 7.6 Hz, 1.38 Hz, 2H, CH), 7.29 (td, J = 7.5 Hz, 1.06 Hz, 2H, CH), 7.26 (d, J = 7.7 Hz, 2H, CH). The ^{1}H NMR data are consistent with that reported in the literature.[39, 114, 115]

C–C borylation of 1-(3-thiophene)biphenylene (1r) at 140 °C (Figure 2)

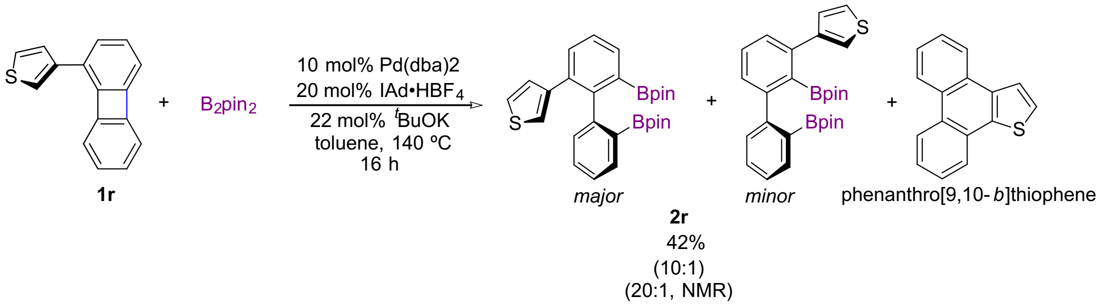

The reaction was conducted according to the general procedure using 1-(3-thiophene)biphenylene, **1r** (88 mg 0.376 mmol), B_2pin_2 (191 mg, 0.752 mmol), $Pd(dba)_2$ (22 mg, 0.038 mmol), 1,3-bis(1-adamantyl)imidazolium tetrafluoroborate (32 mg, 0.075 mmol), KO^{t}Bu (10 mg, 0.083 mmol), and toluene (3.8 mL). The reaction mixture was stirred at 140 °C for 16 h. The crude product was purified by flash column chromatography (silica gel, 0.5 – 50% EtOAc in hexane).

Yield of 2r (major+minor): 42%; major:minor regioisomer ratio = 10:1 (based on isolated yields), 20:1 (based on ^{1}H NMR spectrum of the crude product).

The major isomer of 2r: 40% (0.15 mmol). Yellow oil (94 mg) containing the product with an unidentified impurity. Yield determined by ^{1}H NMR using 1,3,5-trimethoxybenzene as an internal standard.

The minor isomer of 2r: Pale-yellow oil (10 mg) containing an impurity. The yield of **2r** (2%, 0.0075 mmol) was determined by ^{1}H NMR using 1,3,5-trimethoxybenzene as an internal standard.

Characterization of major regioisomer 2r.

^{1}H NMR (500 MHz, $CDCl_3$) δ 7.65-7.63 (m, 1H, CH), 7.58 (dd, *J* = 7.5 Hz, 1.1 Hz, 1H, CH), 7.46 (dd, *J* = 7.7 Hz, 1.2 Hz, 1H, CH), 7.32 (t, *J* = 7.5 Hz, 1H, CH), 7.20 – 7.19 (m, 2H, CH), 7.02-7.00 (m, 1H, CH), 6.99-6.97 (m, 1H, CH), 6.87 (m, 1H, CH), 6.82 (d, *J* = 5.0 Hz, 1H, CH), 1.11 (s, 6H, CH_3), 1.05 (s, 12H, CH_3), 1.02 (s, 6H, CH_3).

^{13}C{^{1}H} NMR (126 MHz, $CDCl_3$) δ 148.6 (C), 147.4 (C), 142.8 (C), 135.4 (C), 133.8 (CH), 132.3 (CH), 130.9 (CH), 130.0 (CH), 129.6 (CH), 129.2 (CH), 126.1 (CH), 125.6 (CH), 123.6 (CH), 123.0 (CH), 83.2 (2C), 83.1 (2C), 25.0 ($2CH_3$), 24.9 ($2CH_3$), 24.5 ($2CH_3$), 24.4 ($2CH_3$).

HRMS (ESI+) m/z: clad for $[C_{28}H_{34}B_2O_4S+H]^+$ 489.2442; found 489.2453.

Characterization of minor regioisomer 2r.

^{1}H NMR (500 MHz, $CDCl_3$) δ 1.10 and 1.07 (characteristic Bpin CH_3 signals). Complete NMR assignment was not possible due to sample impurity.

HRMS (ESI+) m/z: calcd for $[C_{28}H_{34}B2O_4S+Na]^+$ 511.2262; found 511.2270.

Phenanthro[9,10-b]thiophene: white solid (17 mg) containing an impurity. Yield: 10% (0.038 mmol) was determined by ^{1}H NMR using 1,3,5-trimethoxybenzene as an internal standard.

^{1}**H NMR** (500 MHz, $CDCl_3$) δ 8.71 – 8.68 (m, 2H, CH), 8.33- 8.31 (m, 1H, CH), 8.16-8.14 (m, 1H, CH), 7.97 (d, J = 5.3 Hz, 1H, CH), 7.68-7.62 (m, 4H, CH), 7.56 (d, J = 5.3 Hz, 1H, CH). The ^{1}H NMR data was consistent with that reported in the literature.[116]

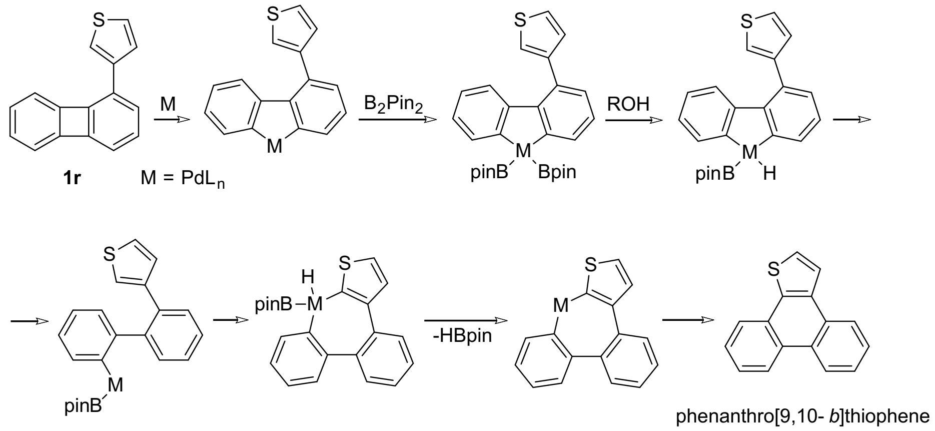

Figure S7. Proposed mechanism for the formation of phenanthro[9,10-*b*]thiophene.

Attempted C–C borylation of 1-(2-pyridyl)biphenylene (1s) at 140 °C (Figure 2)

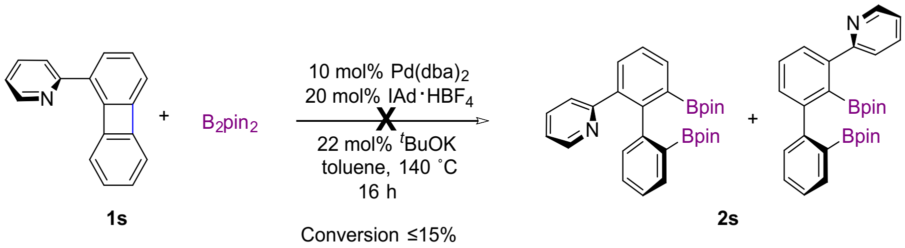

The reaction was conducted according to the general procedure using 1-(2- pyridyl)biphenylene (92.0 mg, 0.401 mmol), B_2pin_2 (206 mg, 0.811 mmol), $Pd(dba)_2$ (22.5 mg, 0.0391 mmol), 1,3-bis(1-adamantyl)imidazolium tetrafluoroborate (35.5 mg, 0.0837 mmol), KO^{t}Bu (9.9 mg, 0.088 µmol), and toluene (4 mL). The reaction mixture was stirred at 140 °C for 16 h. The crude product was purified by flash column chromatography (silica gel, 5% EtOAc in hexane). No expected product was isolated. Starting material was recovered as a yellow oil (82 mg, 85%).

Attempted C–C borylation of 1-(2-pyridyl)biphenylene (1s) at 180 °C (Figure 2)

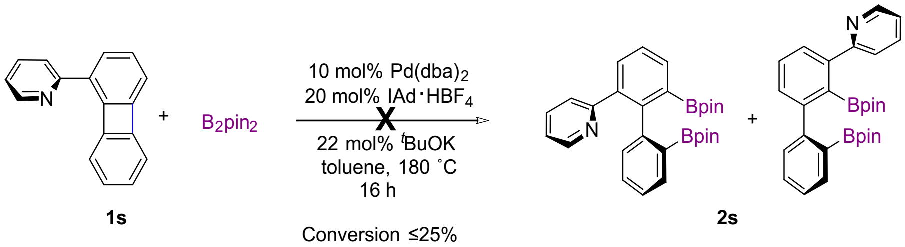

The reaction was conducted according to the general procedure using 1-(2- pyridyl)biphenylene, **1s** (92.0 mg, 0.401 mmol), B_2pin_2 (203 mg, 0.799 mmol), $Pd(dba)_2$ (22.5 mg, 0.0391 mmol), 1,3-bis(1-adamantyl)imidazolium tetrafluoroborate (33.0 mg, 0.0778 mmol), KO^{t}Bu (9.9 mg, 0.088 mmol), and

toluene (4 mL). The reaction mixture was stirred at 180 °C for 16 h. The crude product was purified by flash column chromatography (silica gel, 5% EtOAc in hexane). No expected product was isolated. Starting material was recovered as a yellow oil (72 mg, 75%).

Attempted C–C borylation of 1-diphenylphosphinobiphenylene (1t) at 140 °C (Figure 2)

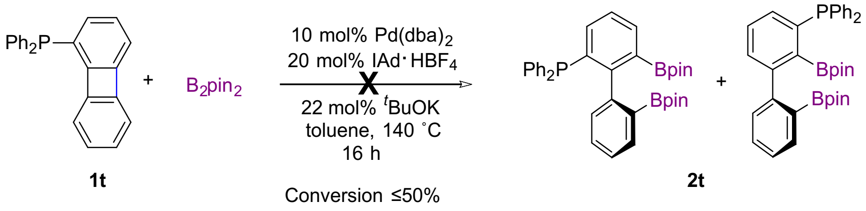

The reaction was conducted according to the general procedure using 1-diphenylphosphinobiphenylene, **1t** (123.0 mg, 0.3657 mmol), B_2pin_2 (186 mg, 0.732 mmol), $Pd(dba)_2$ (21.0 mg, 0.0366 mmol), 1,3-bis(1-adamantyl)imidazolium tetrafluoroborate (31.0 mg, 0.0731 mmol), KO^{t}Bu (9.2 mg, 0.082 mmol), and toluene (3.7 mL). The reaction mixture was stirred at 140 °C for 16 h. It was dark brown at room temperature and remained dark brown upon heating. The crude product was purified by flash column chromatography (silica gel, 1-20% EtOAc in hexane). No expected product was isolated. A yellow oil (70 mg) was obtained, which contained starting material (~50%) and an unknown side product. An orange gum (46 mg) was also isolated but could not be identified.

Attempted C–C borylation of 1-diphenylphosphinobiphenylene (1t) at 180 °C (Figure 2)

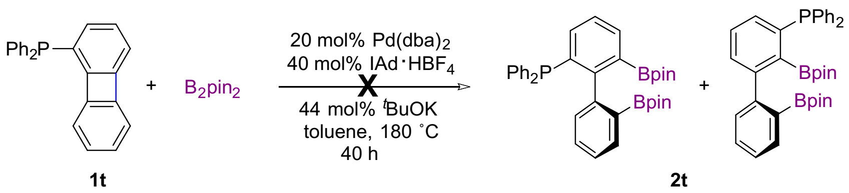

The reaction was conducted according to the general procedure using 1-diphenylphosphinobiphenylene, **1t** (134.0 mg, 0.3984 mmol), B_2pin_2 (203 mg, 0.799 mmol), $Pd(dba)_2$ (23.0 mg, 0.0400 mmol), 1,3-bis(1-adamantyl)imidazolium tetrafluoroborate (34.3 mg, 0.0808 mmol), KO^{t}Bu (9.9 mg, 0.088 mmol), and toluene (4 mL). The reaction mixture was stirred at 180 °C for 16 h. It was dark brown in colour at room temperature and remained dark brown upon heating. The reaction mixture was cooled to room temperature, and $Pd(dba)_2$ (23.2 mg, 0.0403 mmol), 1,3-bis(1-adamantyl)imidazolium tetrafluoroborate (40.0 mg, 0.0943 mmol), and KO^{t}Bu (10.0 mg, 0.0891 mmol) were added. The mixture was then stirred at 180 °C for a further 24 h. The crude product was purified by flash column chromatography (silica gel, 0.5-0.75% EtOAc in hexane). No expected product was isolated. A yellow solid (33 mg), white solid (12 mg) and a brown oil (116 mg) were isolated, but none could be identified.

5. Variation of diboranes in the palladium-catalyzed C–C diborylation of 1-phenylbiphenylene (1e).

To improve the regioselectivity and product yields of the palladium-catalyzed C–C diborylation, we evaluated five different commercially available diboron esters $B_2(OR)_2$ with varying steric bulk (Table S2) using the optimized reaction conditions found for borylation of 1-fluorobiphenylene (Table S1). We hypothesized that the use of bulkier analogues of B_2Pin_2 might lead to more selective formation of the major regioisomer of the diboronate product, which results from the cleavage of the least sterically hindered C–C bond in the starting 1-phenylbiphenylene; while formation of the minor isomer will be hampered because it requires the cleavage of the more sterically hindered C–C bond. However, none of the tested diboron esters gave improved selectivity or yield.

The borylation with a more sterically hindered ethyl analogue of B_2Pin_2 (Table S2, Entry 2) occurred with a low conversion and failed to give any diboronate product. The less sterically hindered and more electron-poor B_2Cat_2, which is often more reactive in C=C borylation reactions,[117] was unreactive under the reaction conditions (Table S2, Entry 3). A similar result was observed with bis(neopentylglycolato)diboron (B_2Neop_2) (Table S2, Entry 4).

Surprisingly, a bulkier homologue of B_2Pin_2 with six-membered rings was found to be the most reactive among the diboron esters tested (Table S2, Entry 5): the reaction went to completion, yet the yield of the diboronate product as well as the regioselectivity were lower compared to B_2Pin_2: 39% vs 44% and 3.8:1 vs l0:1 (Table S2, Entries 5 and 1).

Notably, attempted borylation of 1-phenylbiphenylene with the two bulkiest diboron esters (Table S2, Entries 2 and 5) led to triphenylene (**B**) as the main side product. A potential mechanism for the formation of triphenylene is similar to that in Figure S7.

General procedure

In an argon-filled glovebox, a Schlenk bomb with a Teflon tap was charged with $Pd(dba)_2$ (10 mol%), 1,3-di-1-(adamantyl)imidazolium tetrafluoroborate (20 mol%), KO*t*Bu (22 mol%), diborane (0.8 mmol) and a magnetic stir bar. A solution of 1-phenylbiphenylene (0.4 mmol) in toluene (4 mL) was then added. The Schlenk bomb was sealed by closing the Teflon tap and the reaction mixture stirred (600 rpm) at room temperature for 10 min. The reaction vessel was then removed from the glovebox and the reaction mixture was then stirred (600 rpm) in an oil bath at 110 °C for 16 h. The reaction mixture was filtered through a celite pad and the pad was then washed with EtOAc until no compound was visible on a TLC plate in UV light. The solvent was removed under reduced pressure and the crude product purified by flash column chromatography.

Table S2. Variation of boranes in the palladium-catalyzed C–C diborylation of 1-phenylbiphenylene.

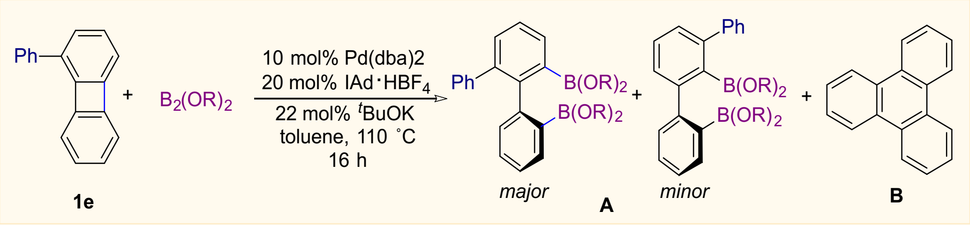

Entry	$B_2(OR)_2$	Conversion of **1e**, %[a]	Yield of **A**, %[b]	Regioselectivity (**A** , major/minor)	Yield of **B**, %[b]
1	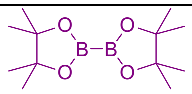	78	44	10:1	0
2	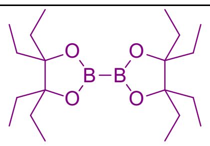	32	0	n/a	8
3	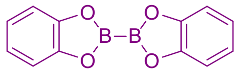	8	0	n/a	0
4	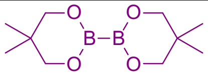	28	0	n/a	0
5	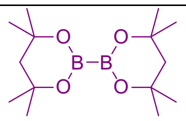	100	39[c]	3.8:1[d]	14

IAd was generated *in situ* by the deprotonation of 1,3-bis(1-adamantyl)imidazolium tetrafluoroborate with 22 mol% KO^{t}Bu. [a] Calculated on the basis of the recovered starting material **1e** ; [b] isolated yields; [c] Resin-like product, likely oligomer or polymer of **1e**, was formed as a side product; [d] The exact assignment of the identity of the two regioisomers (major/minor) was not performed.

Palladium-catalyzed C–C diborylation of 1-phenylbiphenylene (1e) with bis(pinacolato)diboron (Table S2, Entry 1).

See page S25.

Palladium-catalyzed C–C diborylation of 1-phenylbiphenylene (1e) with 4,4,4’,4’,6,6,6’,6’-octamethyl-2,2’-bi(1,3,2-dioxaborinane) (Table S2, Entry 2)

The reaction was conducted according to the general procedure using $Pd(dba)_2$ (21.8 mg, 38 μmol), 1,3-di-1-(adamantyl)imidazolium tetrafluoroborate (32.2 mg, 76 μmol), KO*t*Bu (9.5 mg, 83.6 μmol), 4,4,4',4',5,5,5',5'-octaethyl-2,2'-bi(1,3,2-dioxaborolane) (277.5 mg, 0.76 mmol), 1-phenylbiphenylene (86.8 mg, 0.38 mmol), and toluene (3.8 mL). At room temperature, the reaction mixture was wine-red in colour, but turned dark brown upon heating. The crude product was purified by flash column chromatography (silica gel, 0-5% EtOAc in hexane). Starting material **1e** was recovered with a 50% yield (43 mg, 0.19 mmol). Adittionally, a yellow oil (26 mg) was isolated; it contained a mixture of

starting material **1e** (18%), triphenylene, **B** (8%), along with a small aliphatic impurity, which was most probably a decomposition product of the carbene ligand.

Palladium-catalyzed C–C diborylation of 1-phenylbiphenylene (1e) with bis(catecholato)diboron (Table S2, Entry 3)

The reaction was conducted according to the general procedure using $Pd(dba)_2$ (11.3 mg, 20 µmol), 1,3-di-1-(adamantyl)imidazolium tetrafluoroborate (16.6 mg, 40 µmol), KO*t*Bu (5 mg, 44 µmol), bis(catecholato)diboron (95mg, 0.4 mmol), 1-phenylbiphenylene (44.8 mg, 0.2 mmol), and toluene (2 mL). At room temperature, the reaction mixture was dark brown in colour, but upon heating a black solid crashed out. The crude product was purified by flash column chromatography (silicagel, 0-30% EtOAc in hexane). Starting material **1e** was recovered with a 92% yield (41 mg, 0.18 mmol.

Palladium-catalyzed C–C diborylation of 1-phenylbiphenylene (1e) with bis(neopentylglycolato)diboron (Table S2, Entry 4)

The reaction was conducted according to the general procedure using $Pd(dba)_2$ (11.6 mg, 20 µmol), 1,3-di-1-(adamantyl)imidazolium tetrafluoroborate (17.1 mg, 40 µmol), KO*t*Bu (5.2 mg, 44 µmol), bis(neopentylglycolato)diboron (92 mg, 0.4 mmol), 1-phenylbiphenylene (45.8 mg, 0.2 mmol), and toluene (2 mL). At room temperatureб the reaction mixture was wine-red in colour, but upon heating the it turned dark brown. The crude product was purified by flash column chromatography (silica gel, 0-5% EtOAc in hexane). Starting material **1e** was recovered with a 72% yield (33 mg, 0.14 mmol).

Palladium-catalyzed C–C diborylation of 1-phenylbiphenylene (1e) with 4,4,4',4',6,6,6',6'-octamethyl-2,2'-bi(1,3,2-dioxaborinane) (Table S2, Entry 5)

The reaction was conducted according to the general procedure using $Pd(dba)_2$ (23.3 mg, 40 µmol), 1,3-di-1-(adamantyl)imidazolium tetrafluoroborate (34 mg, 80 µmol), KO*t*Bu (9.9 mg, 88 µmol), 4,4,4',4',6,6,6',6'-octamethyl-2,2'-bi(1,3,2-dioxaborinane) (226 mg, 0.8 mmol), 1-phenylbiphenylene (91 mg, 0.4 mmol), and toluene (4 mL). At room temperature, the reaction mixture was wine-red in colour; upon heating it turned dark brown. The crude product was purified by flash column chromatography (silica gel, 0-2% EtOAc in hexane).

Product **2e'** was isolated as a white solid (78 mg, 0.15 mmol), in 39% yield. A mixture of two inseparable regioisomers (ratio 3.8:1, based on the integrals of 1.71 ppm and 1.68 ppm).

Characterization of 2e'(major regioisomer = reg.1, minor regioisomer = reg.2, un = unassigned).

^{1}H NMR (400 MHz, $CDCl_3$) δ 7.77 (d, J = 7.73 Hz, 0.21H reg.2, CH), 7.70 (dd, J = 7.23, 1.16 Hz, 0.21H reg.2, CH), 7.65 – 7.57 (m, 2.13H un, CH), 7.51 (dd, J = 7.85, 1.82 Hz, 0.23H reg.2, CH), 7.45 – 7.39 (m, 0.92H un, CH), 7.33 – 7.30 (m, 1.95H un, CH), 7.23 – 7.17 (m, 1.66H un, CH), 7.15 – 7.02 (m, 4.25H un, CH), 6.86 (m, 0.8H reg.1, CH), 1.71 (s, 1.5H reg.1, CH2), 1.68 (s, 0.38H reg.2, CH2), 1.65 (s, 0.38H reg.2, CH_2), 1.63 (s, 1.5H reg.1, CH_2), 1.17 (s, 4H un, CH_3), 1.11 – 1.05 (m, 20H un, CH_3).

$^{13}C\{^1H\}$ **NMR** (100 MHz, $CDCl_3$) δ 151.0 (C reg.2), 150.3 (C reg.2), 148.6 (C reg.1), 147.8 (C reg.1), 143.2 (C, reg.1), 141.9 (C reg.2), 140.8 (C reg.2), 140.3 (C reg.1), 133.7 (CH reg.2), 133.3 (CH reg.1), 133.1 (CH reg.2), 131.9 (CH reg.1), 130.4 (CH reg.1), 130.3 (CH reg.1), 130.1 (2CH reg.1), 129.1 (CH reg.2), 128.7 (2CH reg.2), 127.7 (CH reg.2), 127.6 (CH reg.2), 128.0 (CH reg.1), 127.3 (2CH reg.2), 127.2 (2CH reg.1), 127.0 (CH reg.2), 125.6 (CH reg.1), 125.5 (CH reg.1), 125.2 (CH reg.2), 124.8 (CH reg.1), 123.9 (CH reg.2), 70.4 (2C reg.1, 2C reg.2), 70.3 (2C reg.1, 2C reg.2), 48.9 (CH_2 reg.1), 48.8 (CH_2 reg.1, 2CH_2 reg.2), 31.7, 31.6, 31.5 (8CH_3 reg.1, 8CH_3 reg.2).

HRMS (ESI^+) m/z: calculated for $(C_{32}H_{40}B_2O_4+Na)^+$ 533.3010, found 533.3025.

Product **B**, triphenylene, was isolated as a white solid (13 mg, 57 μmol) in 14% yield. ^{1}**H NMR** (400 MHz, $CDCl_3$) δ 8.68 – 8.67 (m, 6H, CH), 7.68 – 7.66 (m, 6H, CH). The ^{1}H NMR spectrum is consistent with the literature data.[118]

6. Postfunctionalization of diborylation product 2e

General procedure for arylation of 2e with 3-bromothiophene

3-Bromothiophene and water were degassed by purging with argon for 5 min. A Schlenk bomb was charged with **2e** (1 equiv.), aryl halide (1.2-4.0 equiv.), $Pd(PPh_3)_4$ (10-20% mol%), K_2CO_3 (4 equiv.), water (2 mL/mmol), and THF (8 mL/mmol). The Schlenk bomb was sealed and the reaction mixture stirred (600 rpm) at the stated temperature for the stated time. The reaction mixture was diluted with water (90 mL), aqueous HCl (2M, 3mL), and EtOAc (90 mL). The two phases were separated. The aqueous phase was extracted with EtOAc (2 x 90 mL). The combined organics were dried over $MgSO_4$, filtered, and the solvent was removed under reduced pressure. The crude product was purified by flash column chromatography

Arylation of 2e with 2.4 equiv. 3-bromothiophene at 80 °C

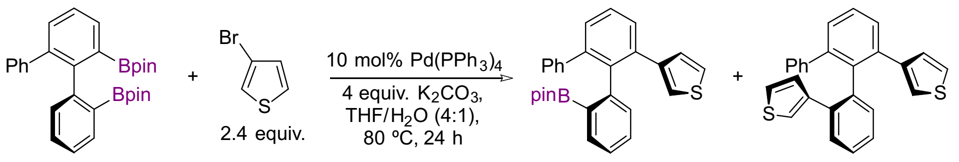

2e **7** 54% **7'** 28%

The reaction was conducted according to the general procedure using **2e** (145.0 mg, 0.301 mmol), 3-bromothiophene (67.5 μL, 118 mg, 0.724 mmol), $Pd(PPh_3)_4$ (34.5 mg, 0.0299 mmol), K_2CO_3 (165 mg, 1.19 mmol), water (0.6 mL), and THF (2.4 mL). The Schlenk bomb was sealed and the reaction mixture stirred (600 rpm) at 80 °C for 24 h. Upon heating the reaction turned dark green. The crude product was purified by flash column chromatography (silica gel, 2% EtOAc in hexane).

Yield of 7: 54%. White solid (51 mg, 0.12 mmol, 39%); an additional 15% (0.045 mmol) could be obtained from the mixed fraction (see below).

Yield of 7': 28%. Colourless oil (8 mg, 0.02 mmol, 6%), contains an unknown impuity; an additional 22 % (0.066 mmol) could be obtained from the mixed fraction (see below).

Mixed fraction: Colourless oil (45 mg) consisting of **7** (0.045 mmol) and **7'** (0.066 mmol).

Characterization of 7

^{1}H NMR (500 MHz, $CDCl_3$) δ 7.56 (d, J = 7.2 Hz, 1H, CH), 7.47 (d, J = 6.8 Hz, 1H, CH), 7.42 (t, J = 7.6 Hz, 1H, CH), 7.35 (d, J = 7.3 Hz, 1H, CH), 7.19-7.09 (m, 7H, CH), 7.02- 6.99 (m, 2H, CH), 6.80 (d, J = 1.8 Hz, 1H, CH), 6.71 (d, J = 4.8 Hz, 1H, CH), 1.11 (s, 12H, CH_3).

^{13}C{^{1}H} NMR (126 MHz, $CDCl_3$) δ 146.4 (C), 142.5 (C), 142.3 (C), 142.0 (C), 140.7 (C), 136.5 (C), 134.8 (CH), 131.5 (CH), 130.1 (2CH), 129.8 (CH), 129.4 (CH), 128.8 (CH), 128.3 (CH), 127.3 (2CH), 126.9 (CH), 126.1 (CH), 125.8 (CH), 123.7 (CH), 123.2 (CH), 83.1 (2C), 24.9 (2CH_3), 24.8 (2CH_3). One quaternary carbon signal is not observed.

HRMS (CI+) m/z: calcd for $[C_{28}H_{27}BO_2S+H]^+$ 439.1903, found 439.1914.

Crystals for XRD analysis were grown by slow evaporation of a concentrated hexane solution at room temperature.

Characterization of 7'

^{1}H NMR (500 MHz, $CDCl_3$) δ 7.45-7.42 (m, 2H, CH), 7.31 (dd, J = 5.9, 2.8 Hz, 1H, CH), 7.22 (d, J = 7.5 Hz, 1H, CH), 7.17 (t, J = 7.5 Hz, 1H, CH), 7.10 – 6.99 (m, 7H, CH), 6.84 (d, J = 6.8 Hz, 2H, CH), 6.68-6.67 (m, 1H, CH), 6.58 (d, J = 5.0 Hz, 1H, CH), 6.53- 6.51 (m, 2H, CH).

^{13}C{^{1}H} NMR (126 MHz, $CDCl_3$) δ 142.3 (C), 142.1 (C), 141.7 (C), 141.5 (C), 138.3 (C), 137.8 (C), 136.9 (C), 136.3 (C), 132.8 (CH), 129.7 (CH), 129.5 (2CH), 129.2 (CH), 129.0 (CH), 128.8 (CH), 128.1 (CH), 127.9 (CH), 127.4 (3CH), 126.6 (CH), 126.2 (CH), 123.9 (2CH), 122.9 (CH), 122.4 (CH).

HRMS (CI+) m/z: calcd for $[C_{26}H_{18}S_2+H]^+$ 395.0928, found 395.0931.

Arylation of 2e with 4 equiv. 3-bromothiophene at 100 °C

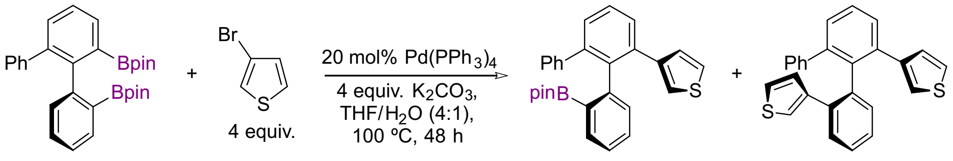

2e **7** 59% **7'** 35%

The reaction was conducted according to the general procedure using **2e** (144.7 mg, 0.3001 mmol), 3-bromothiophene (112 µL, 1.20 mmol), $Pd(PPh_3)_4$ (70.0 mg, 60.6 µmol), K_2CO_3 (167 mg, 1.21 mmol), water (0.6 mL), and THF (2.4 mL). The reaction mixture was stirred (600 rpm) at 100 °C for 48 h. Upon heating the reaction mixture turned black in colour. Additional 3-bromothiophene (112 µL, 1.21 mmol) and $Pd(PPh_3)_4$ (68.0 mg, 58.8 µmol) were added to the reaction mixture and the reaction mixture was heated to 100 °C for a further 24 h. The crude product was purified by flash column chromatography (silica gel, 1-1.5% EtOAc in hexane).

Yield of 7: 59%. White solid (50 mg, 0.11 mmol, 38%), contains a small impurity; an additional 21% (0.063 mmol) could be obtained from the mixed fraction (see below).

Yield of 7': 35%. Yellow solid (58 mg, 0.063 mmol, 11%), contains a small impurity; an additional 24% (0.072 mmol) could be obtained from the mixed fraction (see below).

Mixed fraction: Pale yellow solid (58 mg) consisting of **7** (0.063 mmol) and **7'** (0.072 mmol).

Spectroscopical characterization: see the previous section.

Arylation of 2e with 1.2 equiv. of 3-bromothiophene at 80 °C

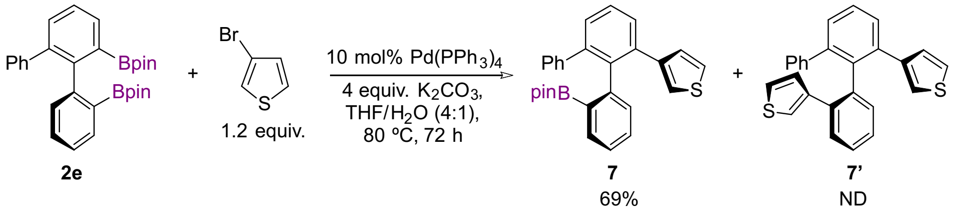

The reaction was conducted according to the general procedure using **2e** (90.8 mg, 0.188 mmol), 3-bromothiophene (37.0 mg, 0.230 mmol), $Pd(PPh_3)_4$ (22.0 mg, 0.0188 mmol), K_2CO_3 (104 mg, 0.752 mmol), water (0.38 mL), and THF (1.5 mL). The Schlenk bomb was sealed and the reaction mixture stirred (600 rpm) at 80 °C for 72 h. The crude product was purified by flash column chromatography (silica gel, 3% EtOAc in hexane).

Yield of 7: 69% (57 mg, 0.129 mmol); pale-yellow oil, solidifying upon standing, containing a minor impurity that could be removed by recrystallization from ethanol.

Spectroscopic characterization: see the previous section.

Palladium-catalyzed allylation of 7 with allyl chloride using 15 mol% Pd

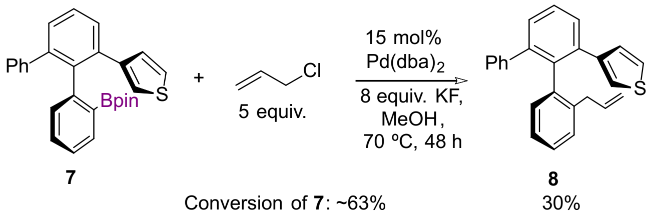

A Schlenk bomb was charged with a stir bar, **7** (57.0 mg, 0.118 mmol), $Pd(dba)_2$ (10.0 mg, 0.018 mmol, 15 mol% Pd), MeOH (2.4 mL) and allyl chloride (50 µl, 0.61 mmol). The reaction mixture was stirred for 10 min, then KF (50.0 mg, 0.944 mmol) was added. The vessel was sealed, and the reaction mixture stirred at 70 °C for 68 h. The crude mixture was cooled to room temperature, filtered through a silica plug, and the plug was washed through with EtOAc until the washings became colourless. The washings were combined and the solvent was removed under reduced pressure. The crude product was purified by flash column chromatography (silica gel, 3% EtOAc in hexane; two consecutive columns).

Yield of 8: 30% (12.5 mg, 0.035 mmol); colorless viscous oil, solidifying upon standing.

Starting material **7** was recovered in 37% yield (21.5 mg, 0.044 mmol).

^{1}H NMR (500 MHz, $CDCl_3$) δ 7.54 (dd, J = 7.7, 1.5 Hz, 1H), 7.47 (t, J = 7.6 Hz, 1H), 7.41 (dd, J = 7.6, 1.4 Hz, 1H), 7.15 – 7.11 (m, 3H), 7.10 – 7.04 (m, 4H), 7.01 – 6.90 (m, 3H), 6.83 – 6.80 (m, 1H), 6.71

(dt, J = 5.0, 1.1 Hz, 1H), 5.34 (ddt, J = 17.0, 10.1, 7.0 Hz, 1H), 4.91 – 4.81 (m, 2H), 2.91 (d, J = 6.9 Hz, 2H).

^{13}C {^{1}H} NMR (126 MHz, $CDCl_3$) δ 142.46, 142.04, 141.78, 139.03, 138.49, 138.00, 136.82, 136.80, 132.03, 129.84, 129.78, 129.53, 129.08, 128.99, 128.59, 127.75, 127.59, 127.25, 126.47, 125.47, 124.15, 123.36, 37.33.

HRMS (CI$^+$) m/z: calcd for $[C_{25}H_{20}S+H]^+$ 353.1359, found 353.1363 (M+H).

Palladium-catalyzed allylation of 7 with allyl chloride using 30 mol% Pd

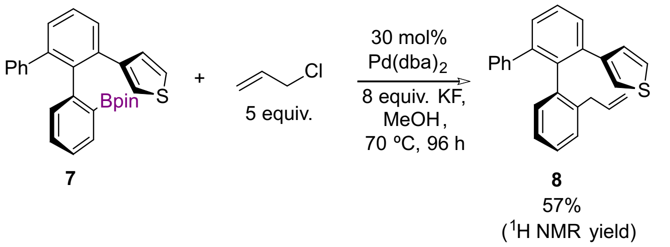

A Schlenk bomb was charged with a stir bar, **7** (11 mg, 0.025 mmol), $Pd(dba)_2$ (2.2 mg, 0.038 mmol, 30 mol% Pd), MeOH (0.5 mL) and allyl chloride (10 µl, 0.13 mmol). The reaction mixture was stirred for 10 min, then KF (12 mg, 0.20 mmol) was added. The vessel was sealed, and the reaction mixture stirred at 70 °C for 96 h. The crude mixture was then filtered through a silica plug, washing through with EtOAc until the washes became colourless. The crude mixture was then concentrated under reduced pressure. The title compound was obtained in 53% NMR yield determined using hexamethyldisiloxane (10 µl) as an internal standard.

Arylation of 2e with 3,4-dibromothiophene

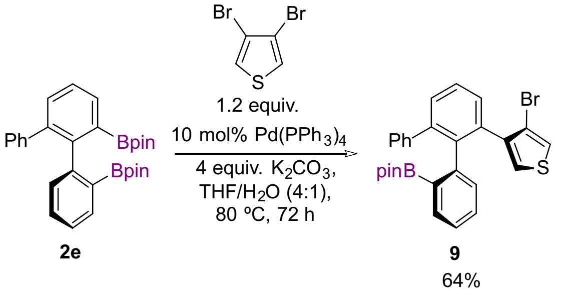

A Schlenk bomb was charged with **2e** (144.5 mg, 0.2996 mmol), 3,4-dibromothiophene (39.8 µL, 0.360 mmol), $Pd(PPh_3)_4$ (34.0 mg, 0.0294 mmol), K_2CO_3 (165 mg, 1.19 mmol), water (0.6 mL), and THF (2.4 mL) to give an orange suspension. The Schlenk bomb was sealed and the reaction mixture stirred (600 rpm) at 80 °C for 48 h. TLC analysis of the reaction mixture showed the precence of traces of **2e** and an additional amount of 3,4-dibromothiophene (33.0 µL, 0.298 mmol) and $Pd(PPh_3)_4$ (34.5 mg, 0.0299 mmol) were added to a reaction mixture. The Schlenlk bomb was then resealed and the reaction mixture was stirred (600 rpm) at 80 °C for a further 24 h. The reaction mixture was cooled to room termperature, and treated with a mixture of water (90 mL), aqueous HCl (2M, 3mL), and EtOAc (90 mL). The two phases were separated and the aqueous phase was extracted with EtOAc (2 x 90 mL). The combined

organic layers were dried over $MgSO_4$, filtered, and the solvent was removed under reduced pressure. The crude product was purified by flash column chromatography (silica gel, 1-2% EtOAc in hexane).

Yield of 9: 64% (99 mg, 0.19 mmol); a colourless oil.

^{1}H NMR (500 MHz, $CDCl_3$) δ 7.45 (t, *J* = 7.6 Hz, 2H, CH), 7.40-7.38 (m, 1H, CH), 7.33-7.32 (m, 1H, CH), 7.15 (td, *J* = 7.5 Hz, 1.3 Hz, 1H, CH), 7.12 (d, *J* = 3.5 Hz, 1H, CH), 7.10-7.06 (m, 6H, CH), 7.03 (t, *J* = 7.4 Hz, 1H, CH), 6.85 (d, *J* = 3.5 Hz, 1H, CH), 1.13 (s, 6H, CH_3), 1.12 (s, 6H, CH_3).

^{13}C{^{1}H} NMR (126 MHz, $CDCl_3$) δ 145.9 (C), 142.2 (C), 141.8 (C), 141.7 (2C), 135.0 (C), 134.4 (CH), 130.8 (CH), 130.2 (2CH), 129.6 (CH), 129.4 (CH), 129.3 (CH), 127.3 (2CH), 126.4 (CH), 126.2 (CH), 125.7 (CH), 124.9 (CH), 121.9 (CH), 112.8 (C), 83.2 (2C), 25.0 (2CH_3), 24.9 (2CH_3). One quaternary carbon signal is not observed.

HRMS (ESI+) m/z: calcd for $[C_{28}H_{26}BBrO_2S+H]^+$ 517.1008, found 517.1002.

7. Stoichiometric experiments on borylation of biphenylene

7.1 Stoichiometric reaction of $Pd(IAd)_2$ with biphenylene

In a glovebox, stock solutions of 1,3,5-trimethoxybenzene (15.5 mg, 92.2 μmol) in d_8-toluene (0.5 mL) and biphenylene (15.0 mg, 98.7 μmol) in d_8-toluene (0.6 mL) were prepared. A J Young tube was charged with $Pd(IAd)_2$ (4.7 mg, 6.0 μmol), d_8-toluene (0.7 mL), biphenylene stock solution (37 μL), and 1,3,5-trimethoxybenzene stock solution (15 μL) to give a pale-yellow reaction mixture. The tube was sealed, removed from the glovebox and heated at the temperatures listed in Table S3. ^{1}H NMR spectra recorded at the time-points indicated.

Table S3 The reaction of $Pd(IAd)_2$ with biphenylene in d_8-toluene.

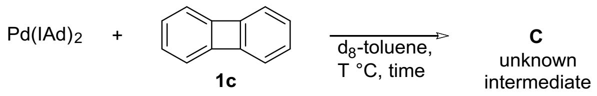

Entry	T, °C	Time	Conversion of biphenylene (1c), %[a]
1	50	2 h	0
2	80	10 min	5
3	80	1 h	18
4	80	2 h	42
5	110	10 min	88
6	110	20 min	100

[a] ^{1}H NMR yield calculated from the ratio of the integral of the multiplets at 6.37 and 6.48 ppm (8H) of biphenylene (**1e**) to the integral of the singlet at 6.13 ppm (3H) of 1,3,5-trimethoxybenzene (internal standard).

7.2 Stoichiometric reaction of $Pd(IAd)_2$ with B_2pin_2

In a glovebox, stock solutions of 1,3,5-trimethoxybenzene (14.8 mg, 88.0 μmol) in d_8-toluene (0.5 mL) and B_2pin_2 (15.0 mg, 59.2 μmol) in d_8-toluene (0.3 mL) were prepared. A J Young tube was charged with $Pd(IAd)_2$ (4.7 mg, 6.0 μmol), d_8-toluene (0.7 mL), B_2pin_2 stock solution (31 μL), and 1,3,5-trimethoxybenzene stock solution (15 μL) to give a pale-yellow mixture. The tube was sealed, removed from the glovebox, and heated at the temperatures listed in Table S4. ^{1}H NMR spectra were recorded at the time-points indicated.

Table S4 Reaction of $Pd(IAd)_2$ with B_2pin_2 in d_8-toluene, which was followed by NMR spectroscopy.

$$Pd(IAd)_2 + B_2pin_2 \xrightarrow[\text{T °C, time}]{d_8\text{-toluene,}} \mathbf{D}$$

D unknown intermediate

Entry	T, °C	Time	Conversion of B_2pin_2, %[a]
1	50	2 h	0
2	80	10 min	8
3	80	1 h	16
4	80	2 h	24
5	110	10 min	27
6	110	1 h	45
7	110	2 h	58
8	110	16 h	98

[a] ^1H NMR conversion calculated from the ratio of the integral of the singlet at 1.02 ppm (24H) of B_2pin_2 to the integral of the singlet at 3.32 ppm (9H) of 1,3,5-trimethoxybenzene (internal standard).

7.3 Thermal stability of $Pd(IAd)_2$ under reaction conditions

In a glovebox, a stock solution of 1,3,5-trimethoxybenzene (15.1 mg, 89.8 µmol) in d_8-toluene (0.5 mL) was prepared. A J Young tube was charged with $Pd(IAd)_2$ (4.9 mg, 6.3 µmol), d_8-toluene (0.7 mL), and 1,3,5-trimethoxybenzene stock solution (15 µL), giving a pale-yellow solution. The tube was sealed, removed from the glovebox, and heated at the temperatures listed in Table S5. ^1H NMR spectra were recorded at the time-points indicated.

Table S5 Decomposition of $Pd(IAd)_2$ upon heating.

Entry	T, °C	Time	$Pd(IAd)_2$ Conversion, %[a]
1	50	2 h	0
2	80	10 min	0
3	80	1 h	3
4	80	2 h	12
5	110	10 min	12
6	110	1 h	20
7	110	2 h	34
8	110	16 h	53

[a] ^1H NMR conversion calculated from the ratio of the integral of the singlet at 6.70 ppm (2H) of $Pd(IAd)_2$ to the integral of the singlet at 6.13 ppm (3H) of 1,3,5-trimethoxybenzene (internal standard).

7.4 Two-step stoichiometric reactions of $Pd(IAd)_2$ with biphenylene and B_2pin_2

7.4.1 General Procedure for the optimization of the reaction of $Pd(IAd)_2$ with biphenylene followed by the addition of B_2pin_2

In a glovebox, stock solutions of 1,3,5-trimethoxybenzene (ca.15 mg, ca. 89 μmol), biphenylene (ca.15 mg, ca. 99 μmol), and B_2pin_2 (ca.15 mg, ca. 59 μmol) in d_8-toluene (0.3–0.5 mL) were prepared. A J Young NMR tube was charged with $Pd(IAd)_2$ (ca. 4.7 mg, ca. 6.0 μmol), d_8-toluene (0.7 mL), 1,3,5-trimethoxybenzene stock solution (15 μL), and biphenylene stock solution (ca. 31 μL) to give a pale-yellow mixture. The NMR tube was sealed, removed from the glovebox, and heated in an oil bath at 110 °C for full conversion of biphenylene (1-1.5 h). B_2pin_2 stock solution (31 μL) was then added, and the mixture (still pale yellow) was reheated at the temperatures listed and times shown in Table S6 (the experiment was conducted in triplicate). ^1H NMR spectra were recorded at the time-points indicated.

Table S6, Run 1 (reagent mol ratio Pd : Biph : B_2pin_2 = 1.00 : 0.99 : 0.99)

The reaction was conducted according to the general procedure using 1,3,5-trimethoxybenzene (15.0 mg, 89.2 μmol), biphenylene (15.0 mg, 98.7 μmol), B_2pin_2 (15.0 mg, 59.3 μmol), and $Pd(IAd)_2$ (4.8 mg, 6.2 μmol).

Table S6, Run 2 (reagent mol ratio Pd : Biph : B_2pin_2 = 1.00 : 1.04 : 1.04)

The reaction was conducted according to the general procedure using 1,3,5- trimethoxybenzene (14.9 mg, 88.6 μmol), biphenylene (15.1 mg, 99.3 μmol), B_2pin_2 (15.0 mg, 59.1 μmol), and $Pd(IAd)_2$ (4.6 mg, 5.9 μmol.

Table S6, Run 3 (reagent mol ratio Pd : Biph : B_2pin_2 = 1.00 : 1.05 : 1.04)

The reaction was conducted according to the general procedure using 1,3,5- trimethoxybenzene (14.9 mg, 88.6 μmol), biphenylene (15.2 mg, 100.0 μmol), B_2pin_2 (15.1 mg, 59.5 μmol), and $Pd(IAd)_2$ (4.6 mg, 5.9 μmol).

Table S6, Run with 2 equiv. B_2pin_2 (reagent mol ratio Pd : Biph : B_2pin_2 = 1.00 : 1.03 : 2.08)

The reaction was conducted according to the general procedure using 1,3,5- trimethoxybenzene (15.2 mg, 90.4 μmol), biphenylene (15.1 mg, 99.3 μmol), B_2pin_2 (30.7 mg, 120.9 μmol), and $Pd(IAd)_2$ (4.7 mg, 6 μmol).

Table S6 Optimizaton of the reaction of biphenylene with Pd(IAd)$_2$ followed by reaction with 1 eq. B$_2$pin$_2$ to yield **2c**.

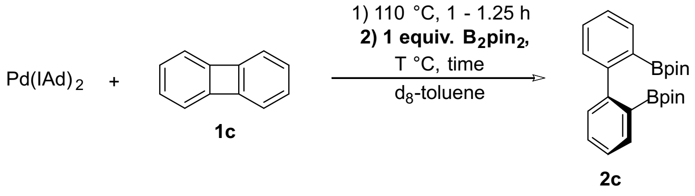

Run 1: 2nd Step (addition of B$_2$pin$_2$)			
**Entry**	**T, °C**	**Time**	**Yield of 2c, %** [a]
1	25	2 h	74
2	25	3 h	77
3	25	4 h	77
4	25	5 h	77
Run 2: 2nd Step (addition of B$_2$pin$_2$)			
**Entry**	**T, °C**	**Time**	**Yield of 2e, %** [a]
5	25	10 min	32
6	25	1 h	74
7	25	2 h	80
8	50	30 min	83
9	50	1.5 h	84
Run 3: 2nd Step (addition of B$_2$pin$_2$)			
**Entry**	**T, °C**	**Time**	**Yield of 2e, %**[a]
10	25	10 min	34
11	25	1 h	78
12	25	2 h	77
13	50	30 min	81
14	50	1.5 h	83

[a] ^{1}H NMR yield calculated from the ratio of the integral of the multiplet at 7.98 ppm (2H) of **2c** and the integral of the singlet at 6.13 ppm (3H) of 1,3,5-trimethoxybenzene (internal standard).

7.4.2 Procedure for the reaction of $Pd(IAd)_2$ with biphenylene (1e) followed by the addition of B_2pin_2

In a glovebox, stock solutions of 1,3,5-trimethoxybenzene (0.0195 g, 0.116 mmol in 0.5 mL d_8-toluene, 0.232 M), $Pd(IAd)_2$ (0.030 g, 0.039 mmol in 1.5 mL d_8-toluene, saturated solution, mild heating required), biphenylene (0.020 g, 0.131 mmol in 0.5 mL d_8-toluene, 0.263 M), and B_2pin_2 (0.036 g, 0.014 mmol in 0.5 mL d_8-toluene, 0.254 M) were prepared. Three J Young NMR tubes were each charged with $Pd(IAd)_2$ solution (0.50 mL, 0.013 mmol), biphenylene solution (0.050 mL, 0.013 mmol), and 1,3,5-trimethoxybenzene solution (0.050 mL, 0.0116 mmol) to give a pale-yellow mixture. An ^1H NMR spectrum was recorded at $t = 0$. The tubes were then heated at 110 °C for 1 h 15 min, with intermittent NMR monitoring, after which the reaction was almost complete.

The tubes were returned to the glovebox, and B_2pin_2 solution (0.050 mL, 0.0036 mmol) was added without mixing. The tubes were carefully removed from the glovebox, kept upright to minimize mixing, then shaken simultaneously immediately before insertion into the NMR spectrometer. ^1H NMR spectra were then recorded within 10 min (Table S7). The tubes were then reheated under the following conditions, and a ^1H NMR spectrum was recorded after each: 50 °C for 30 min; 50 °C for 90 min; 110 °C for 10 min.

Table S7 The reaction of biphenylene with $Pd(IAd)_2$ followed by reaction with 1 eq. B_2pin_2 to yield **2e**.

Run 1: 2nd Step (addition of B_2pin_2)				
**Entry**	**T, °C**	**Time, min**	**Yield of 2c, %[a]**	**Yield of $Pd(IAd)_2$, %[b]**
1	50	30	81	>99
Run 2: 2nd Step (addition of B_2pin_2)				
**Entry**	**T/ °C**	**Time, min**	**Yield of 2c, %**	
2	50	30	77	99%
Run 3: 2nd Step (addition of B_2pin_2)				
**Entry**	**T/ °C**	**Time, min**	**Yield of 2e, %**	
3	110	110	90	>99

[a] ^1H NMR yield calculated from the ratio of the integral of the multiplet at 7.98 ppm (2H) of **2e** to the integral of the singlet at 6.13 ppm (3H) of 1,3,5-trimethoxybenzene (internal standard). [b] ^1H NMR yield calculated from the ratio of the integral of the singlet at 6.70 ppm (2H) of $Pd(IAd)_2$ to the integral of the singlet at 6.13 ppm (3H) of 1,3,5-trimethoxybenzene (internal standard).

7.4.3 General Procedure for the optimization of the reaction of $Pd(IAd)_2$ with B_2pin_2, followed by the addition of biphenylene

In a glovebox, stock solutions of 1,3,5-trimethoxybenzene (ca. 15 mg, ca. 89 µmol) in d_8-toluene (0.5 mL) and biphenylene (ca. 15.0 mg, ca. 99 µmol) in d_8-toluene (0.5 mL), B_2pin_2 (ca. 15 mg, ca, 59 µmol) in d_8-toluene (0.3 mL) were prepared. A J Young tube was charged with $Pd(IAd)_2$ (4.7 mg, 6.0 µmol), d_8-toluene (0.7 mL), 1,3,5-trimethoxybenzene stock solution (15 µL), and B_2pin_2 stock solution (31 µL) to give a pale-yellow mixture. The reaction mixture was heated at 110 °C for 16 h. Upon heating a small amount of black precipitate formed in two out of three runs, but the solution remained pale-yellow. Biphenylene stock solution (31 µL) was then added, with no further colour change, and the reaction mixture was heated at the temperatures listed in Table S8. ^{1}H NMR spectra were recorded at the time-points indicated.

Note to Table S8

In all three runs, no clean signals for the desired diborylated product **2c** were observed more formal and in the ^{1}H NMR spectra; thus, the actual yields of **2c** are lower than those estimated from the ^{1}H NMR integration.

Table S8, Run 1 (reagent mol ratio Pd : B_2pin_2 : Biph = 1.00 : 1.04 : 1.03)

The reaction was conducted according to the general procedure using 1,3,5- trimethoxybenzene (15.1 mg, 89.8 µmol), biphenylene (15.0 mg, 98.7 µmol), B_2pin_2 (15.1 mg, 59.5 µmol), and $Pd(IAd)_2$ (4.6 mg, 5.9 µmol).

Table S8, Run 2 (reagent mol ratio Pd : B_2pin_2 : Biph = 1.00 : 1.02 : 1.01)

The reaction was conducted according to the general procedure using 1,3,5- trimethoxybenzene (14.8 mg, 88.0 µmol), biphenylene (14.9 mg, 98.0 µmol), B_2pin_2 (15.0 mg, 59.1 µmol), and $Pd(IAd)_2$ (4.7 mg, 6.0 µmol). The starting reaction mixture was pale-yellow.

Table S8, Run 3 (reagent mol ratio Pd : B_2pin_2 : Biph = 1.00 : 1.04 : 1.04)

The reaction was conducted according to the general procedure using 1,3,5- trimethoxybenzene (15.0 mg, 89.2 µmol), biphenylene (15.0 mg, 98.7 µmol), B_2pin_2 (15.0 mg, 59.1 µmol), and $Pd(IAd)_2$ (4.6 mg, 5.9 µmol). The starting reaction mixture was pale-yellow in colour.

Table S8 Optimization of the reaction of B_2pin_2 with $Pd(IAd)_2$ followed by reaction with 1 eq. biphenylene to yield **2c**.

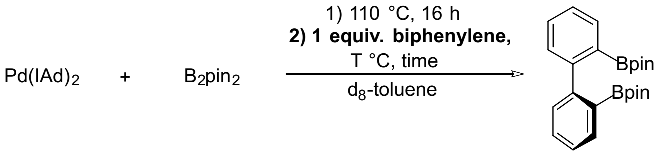

Run 1: 2nd Step (addition of biphenylene), B_2pin_2 after 1st Step: 2.7 μmol				
**Entry**	**T, °C**	**Time**	**Conversion of 1c, %**	**Yield of 2c, %**
1	50	2 h	0	0
2	80	1 h	11	0
3	80	2 h	27	16
4	80	4 h	58	54
5	110	0.5 h	97	55
6	110	1 h	100	55
Run 2: 2nd Step (addition of biphenylene), B_2pin_2 left after 1st Step: 0.12 μmol				
**Entry**	**T, °C**	**Time**	**Conversion of 1c, %**	**Yield of 2c, %**
7	50	2 h	0	0
8	80	1 h	9	0
9	80	2 h	18	3
10	80	4 h	42	13
11	110	0.5 h	78	17
12	110	1 h	91	17
13	110	1 h 15	94	18
Run 3: 2nd Step (addition of biphenylene), B_2pin_2 left after 1st Step: 1.55 μmol				
**Entry**	**T, °C**	**Time**	**Conversion of 1c, %**	**Yield of 2c, %**
14	50	2 h	0	0
15	80	1 h	40	27
16	80	2 h	60	37
17	80	4 h	76	39
18	110	0.5 h	100	39

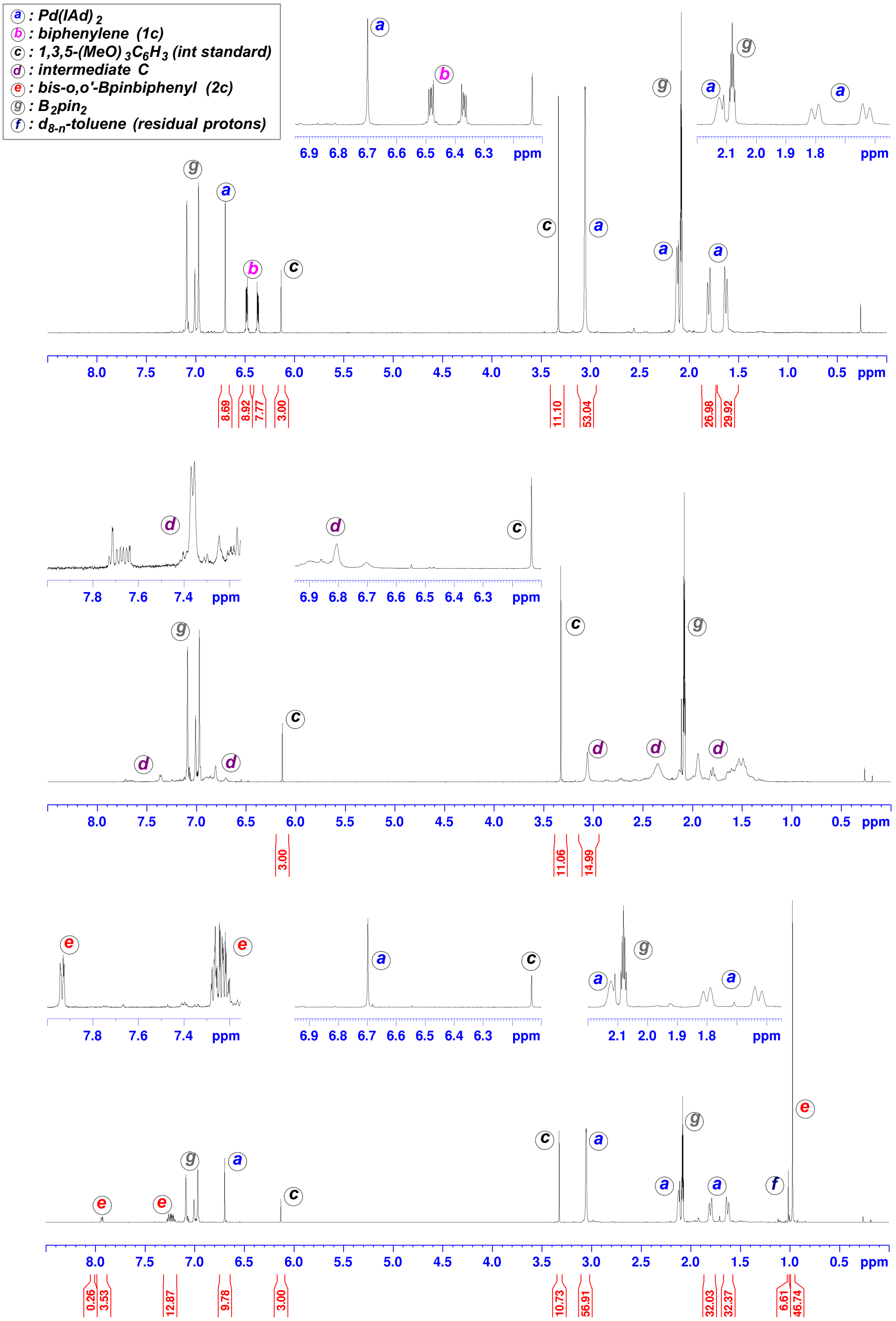

Figure S8. A representative ^{1}H NMR spectra for the reaction of $Pd(IAd)_2$ with biphenylene and then with B_2pin_2: (A) a mixture of 1 equiv. $Pd(IAd)_2$ and 1.05 equiv. biphenylene in d_8-toluene at t=0 h; (B) after heating at 110 °C for 75 min; (C) after the addition of 1.05 equiv. of B_2pin_2 and heating at 50 °C for 90 min.

8. NMR Spectra

NMR spectra of 1-fluorobiphenylene (**1a**)

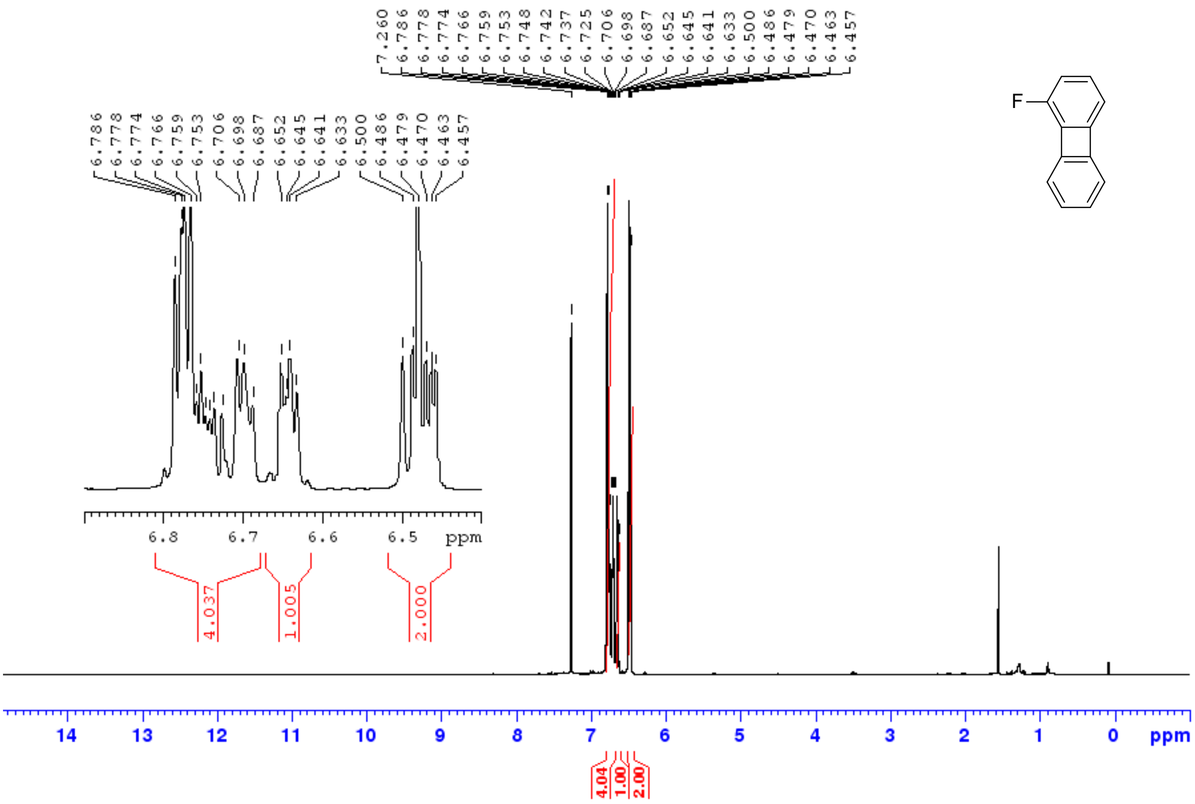

^{1}H NMR (400 MHz) spectrum of **1a** in $CDCl_3$

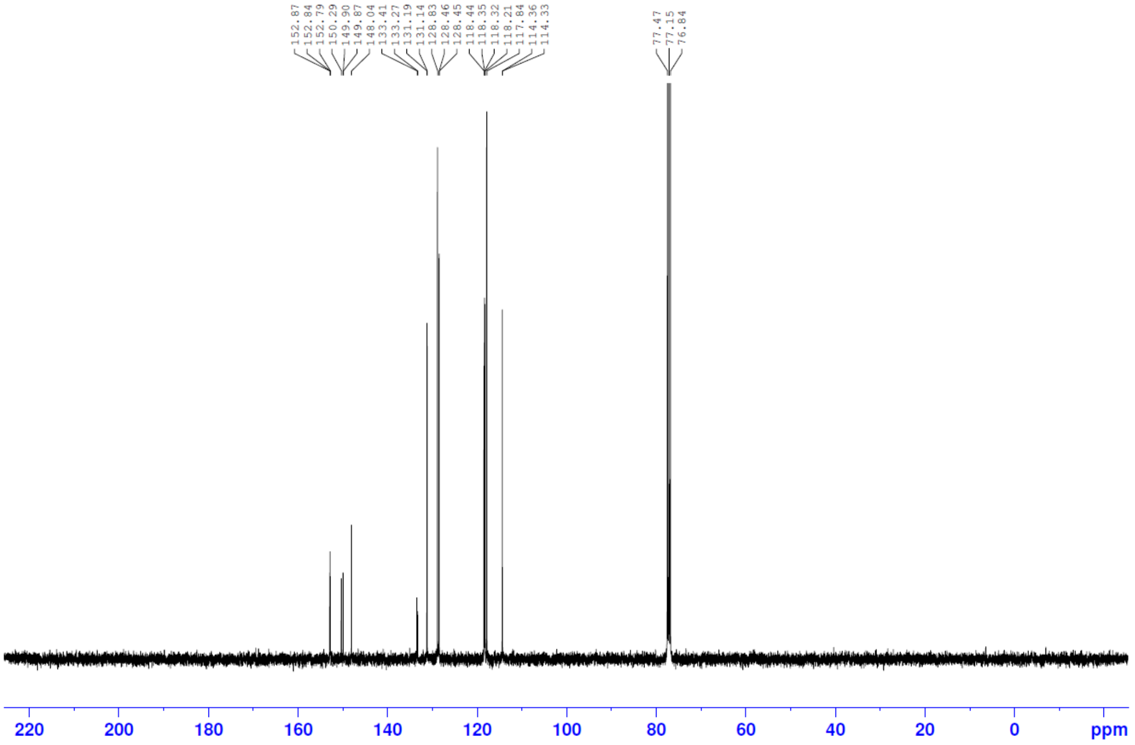

^{13}C NMR (100 MHz) spectrum of **1a** in $CDCl_3$

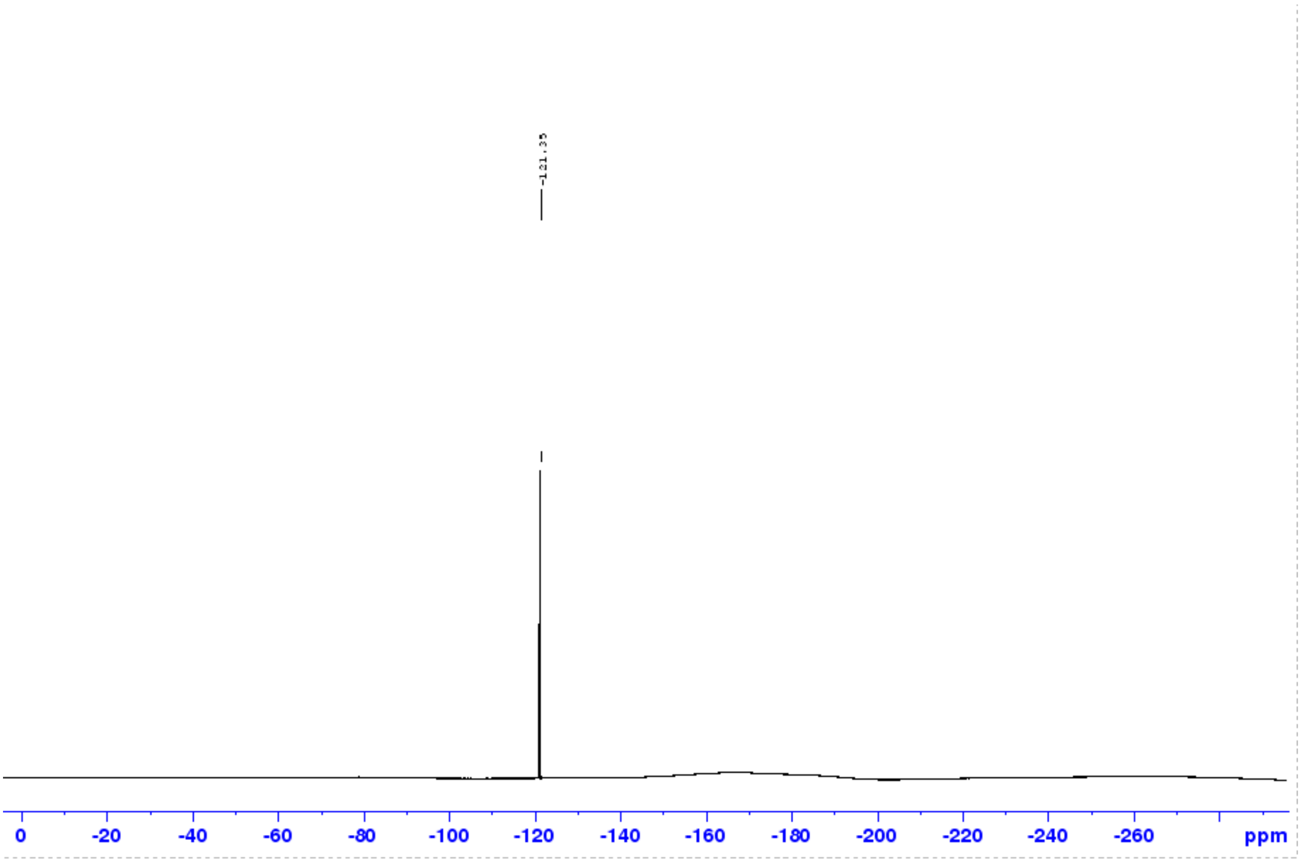

^{19}F NMR (376 MHz) spectrum of **1a** in $CDCl_3$

NMR spectra of 1-chlorobiphenylene (**1b**)

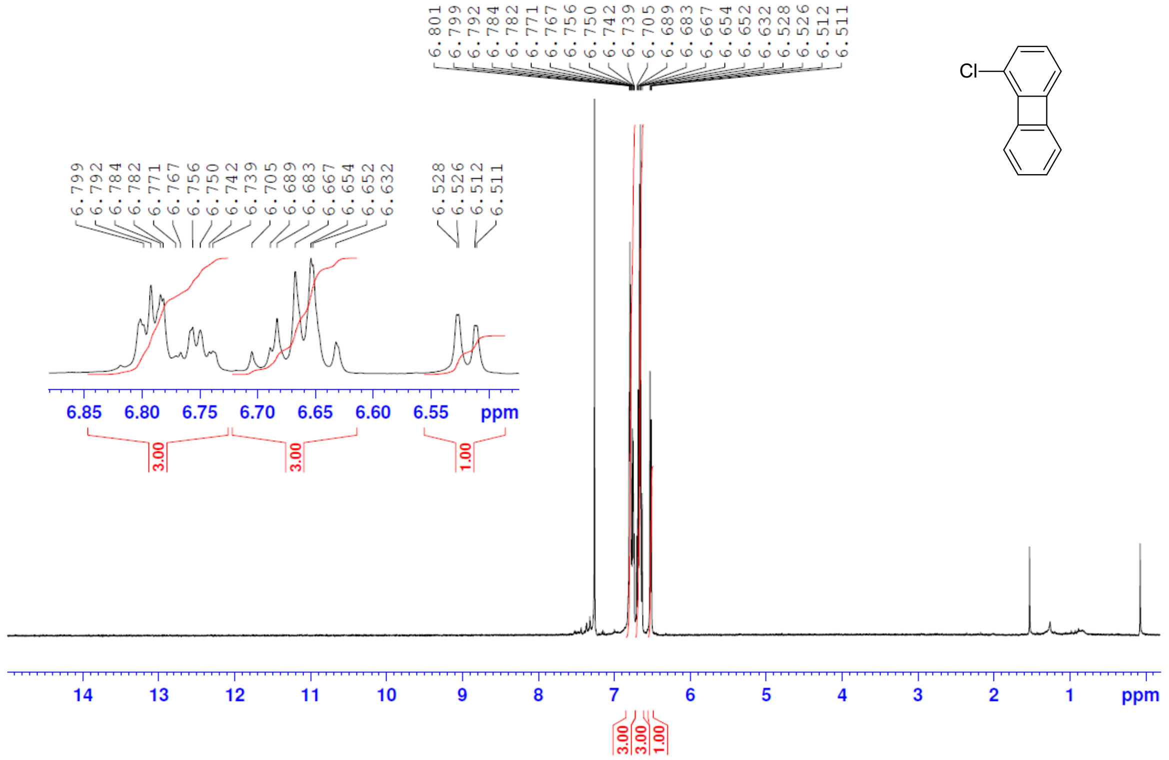

^{1}H NMR (400 MHz) spectrum of **1b** in $CDCl_3$

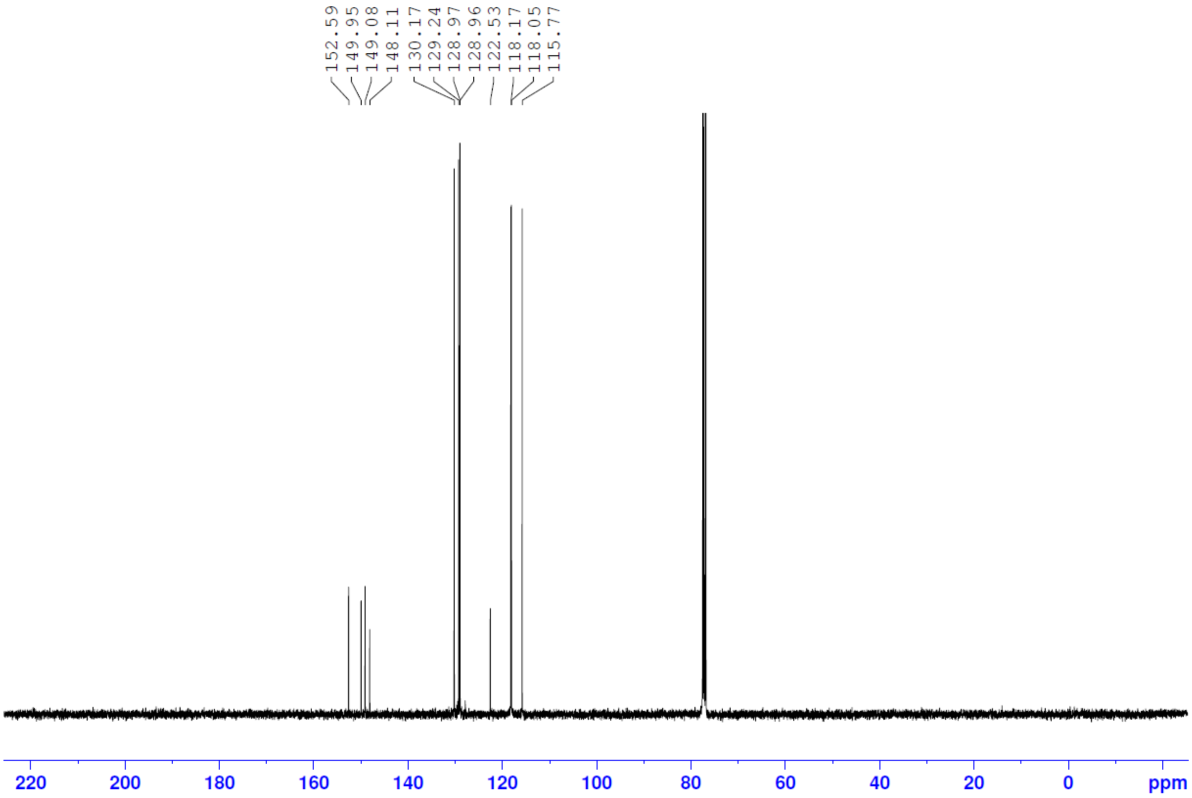

^{13}C NMR (100 MHz) spectrum of **1b** in $CDCl_3$

NMR spectra of 1-iodobiphenylene

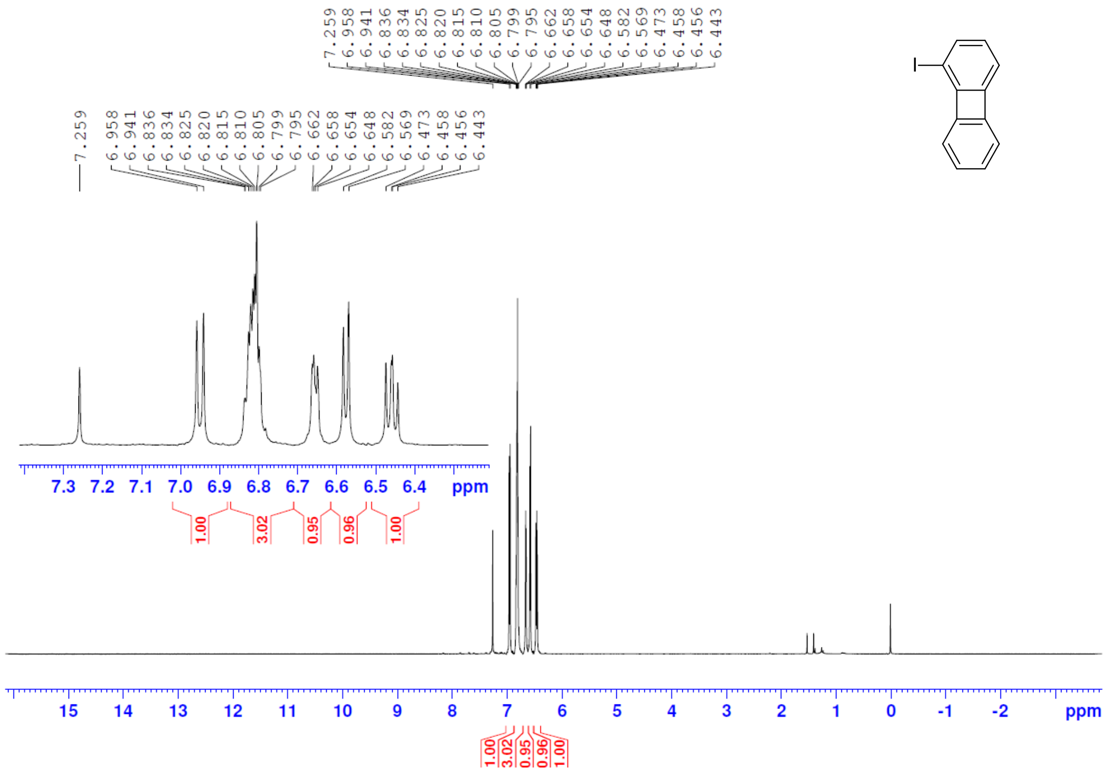

^{1}H NMR (400 MHz) spectrum of 1-iodobiphenylene in $CDCl_3$

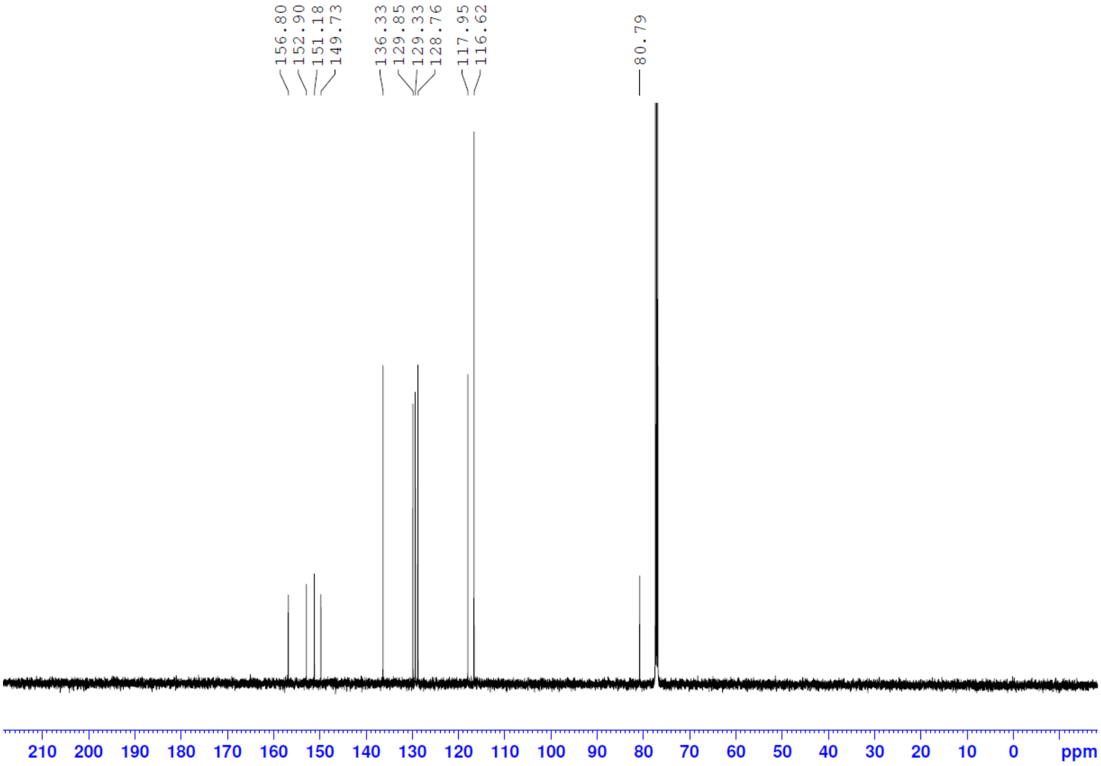

^{13}C NMR (100 MHz) spectrum of 1-iodobiphenylene in $CDCl_3$

NMR spectra of 1-methylbiphenylene (**1d**)

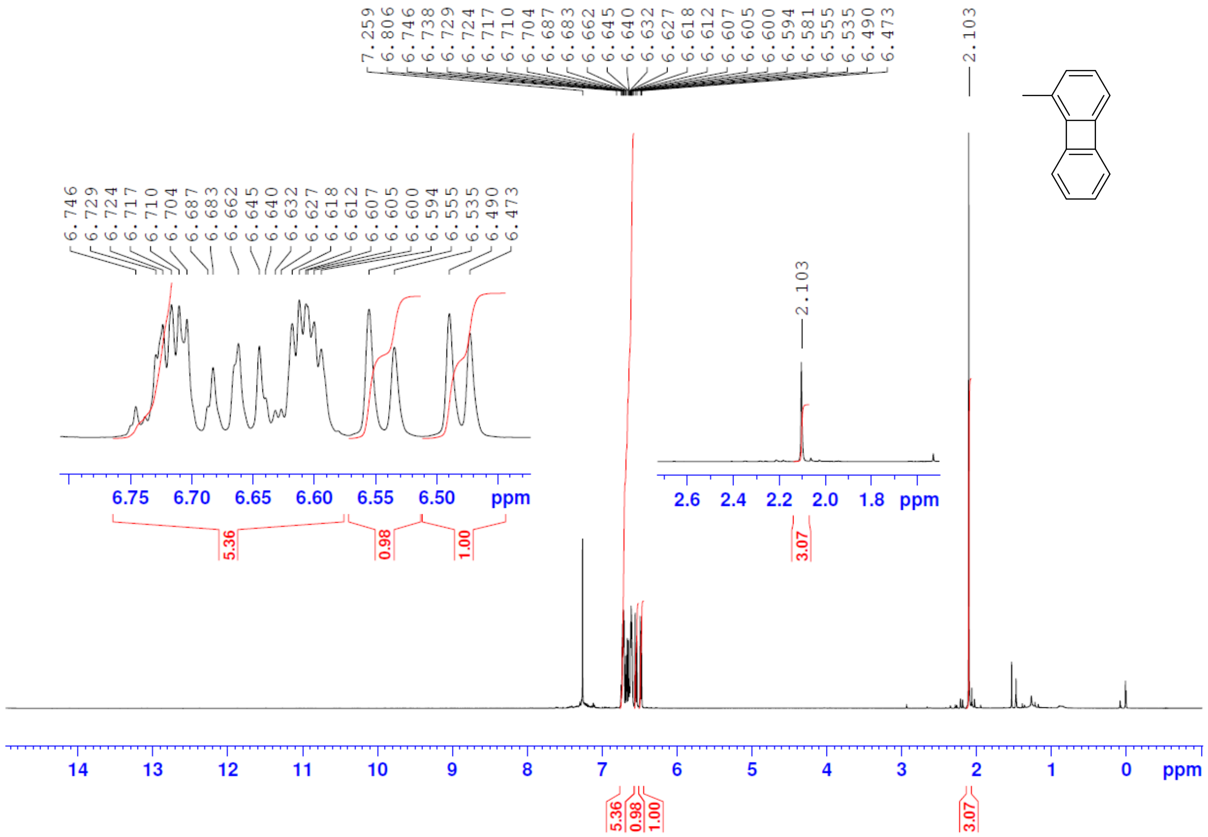

^{1}H NMR (400 MHz) spectrum of **1d** in $CDCl_3$

NMR spectra of 1-(trimethylsilyl)biphenylene (**1p**)

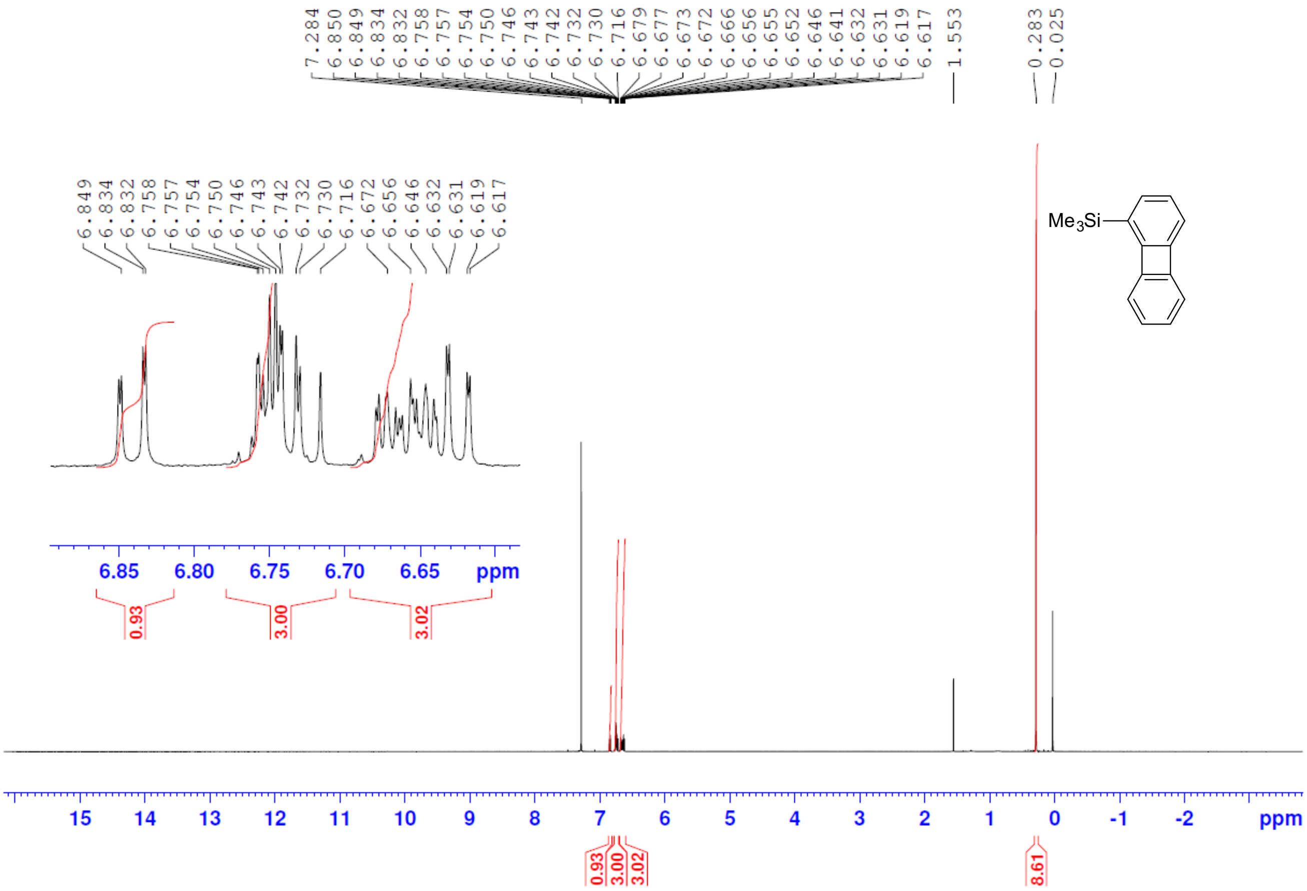

^{1}H NMR (400 MHz) spectrum of **1p** in $CDCl_3$

NMR spectra of 1-(diphenylphospino)biphenylene (**1t**)

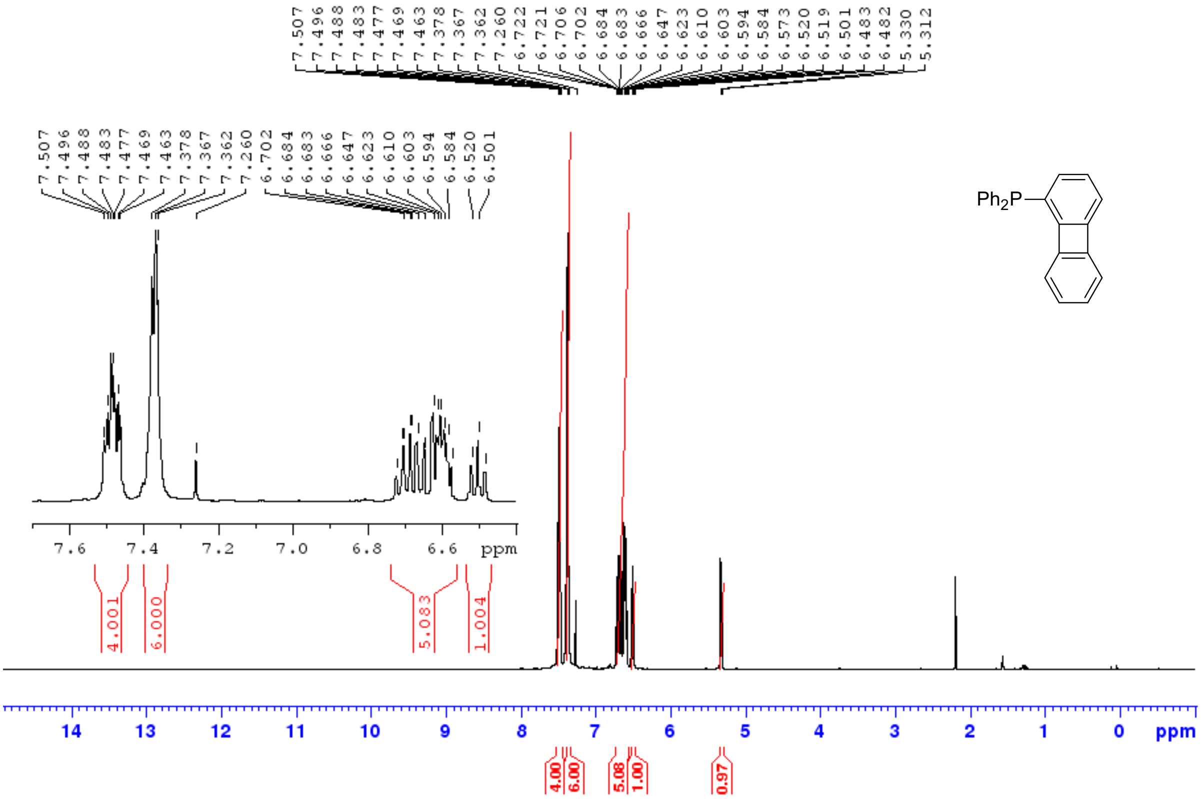

^{1}H NMR (400 MHz) spectrum of **1t** in $CDCl_3$

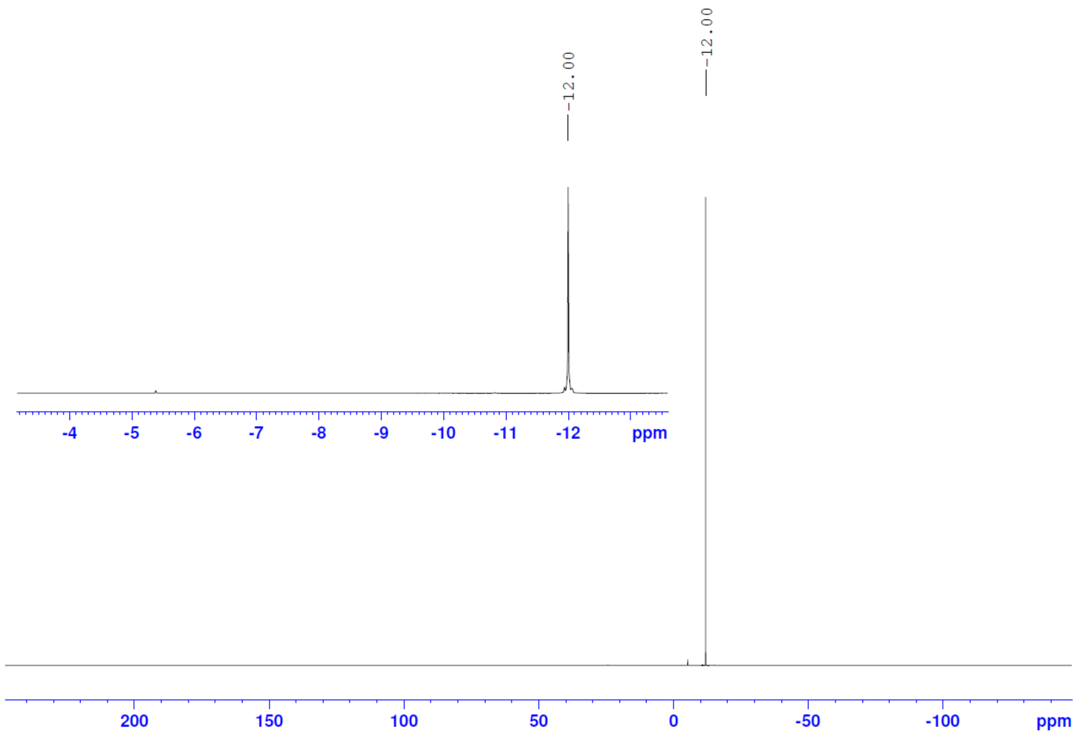

^{31}P NMR (162 MHz) spectrum of **1t** in $CDCl_3$

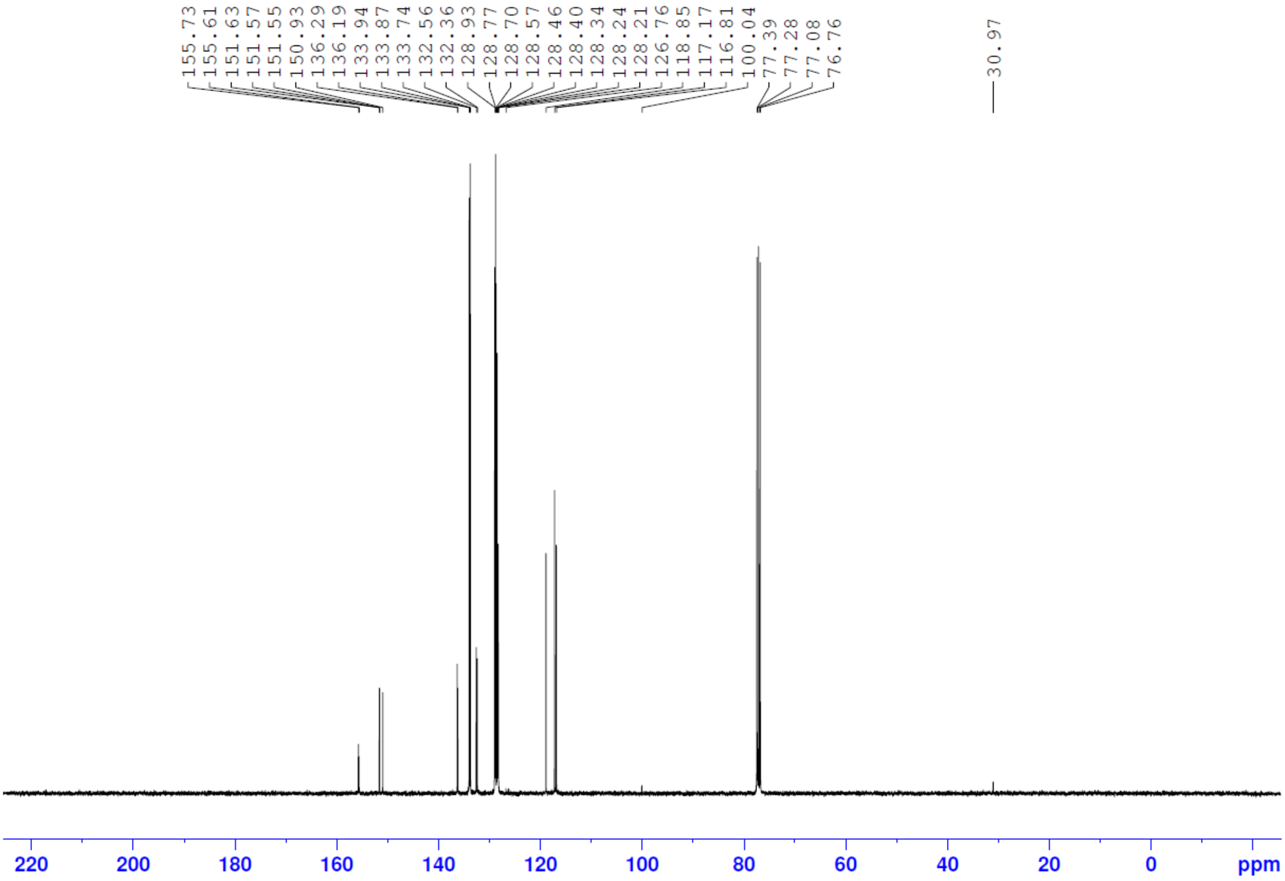

^{13}C NMR (100 MHz) spectrum of **1t** in $CDCl_3$

NMR spectra of 2-(biphenylen-1-yl)-4,4,5,5-tetramethyl-1,3,2-dioxaborolane (**1q**)

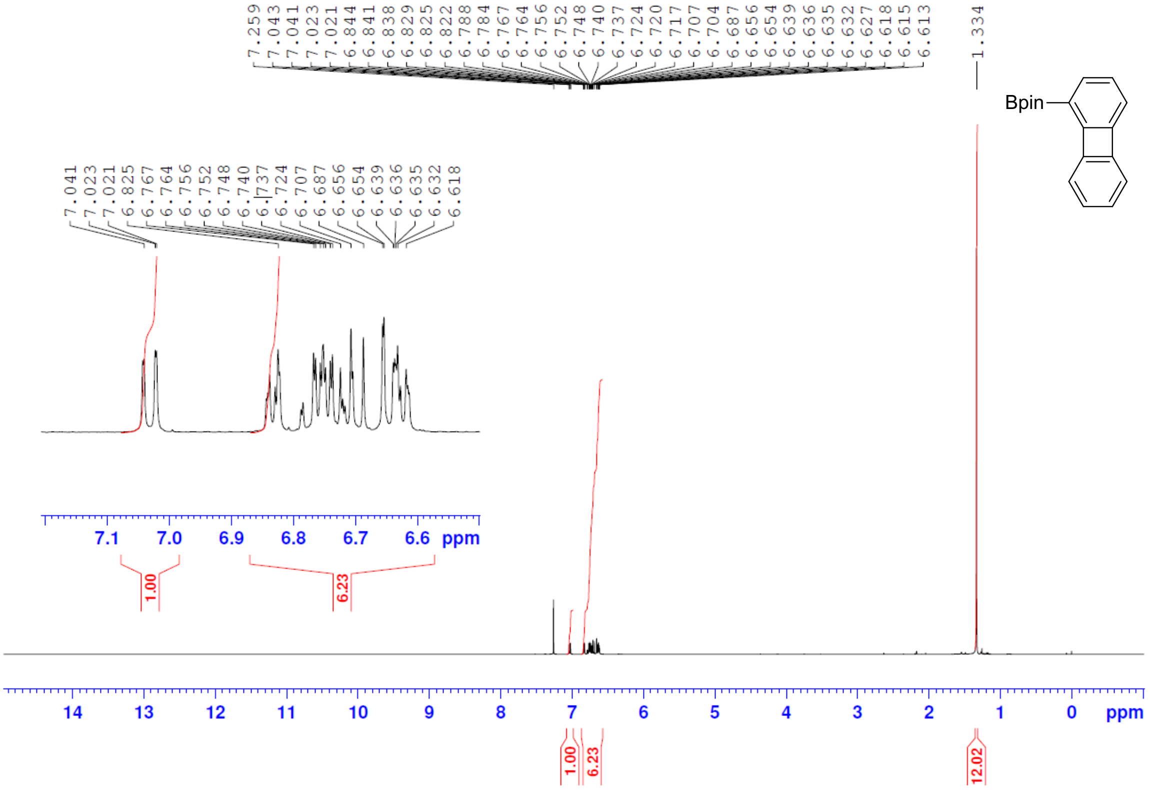

^{1}H NMR (400 MHz) spectrum of **1q** in $CDCl_3$

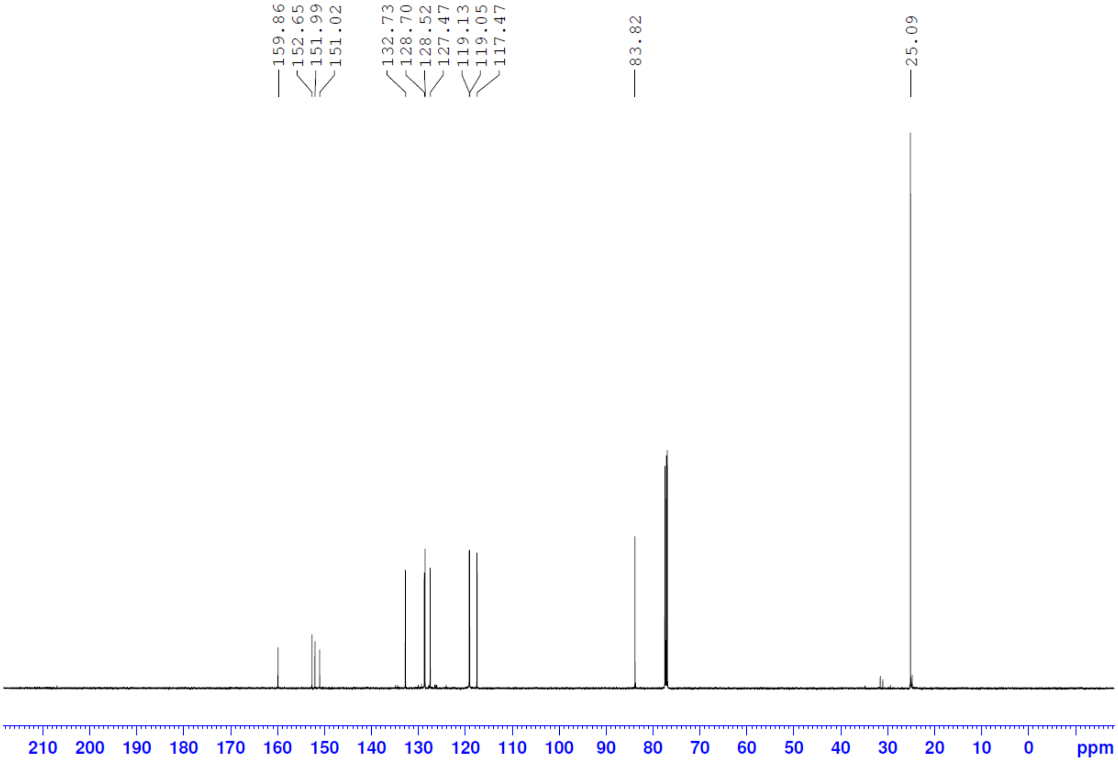

^{13}C NMR (100 MHz) spectrum of **1q** in $CDCl_3$

NMR spectra of 3-(biphenylen-1-yl)thiophene (**1r**)

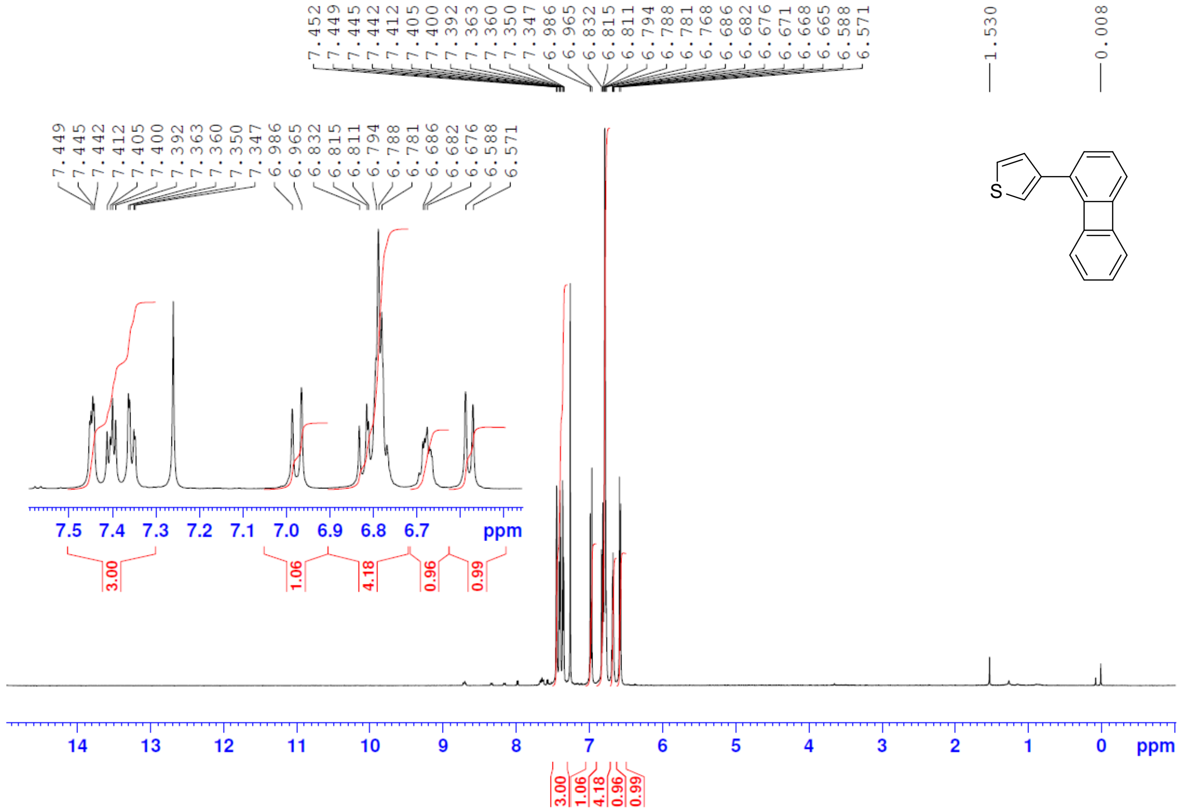

^{1}H NMR (400 MHz) spectrum of **1r** in $CDCl_3$

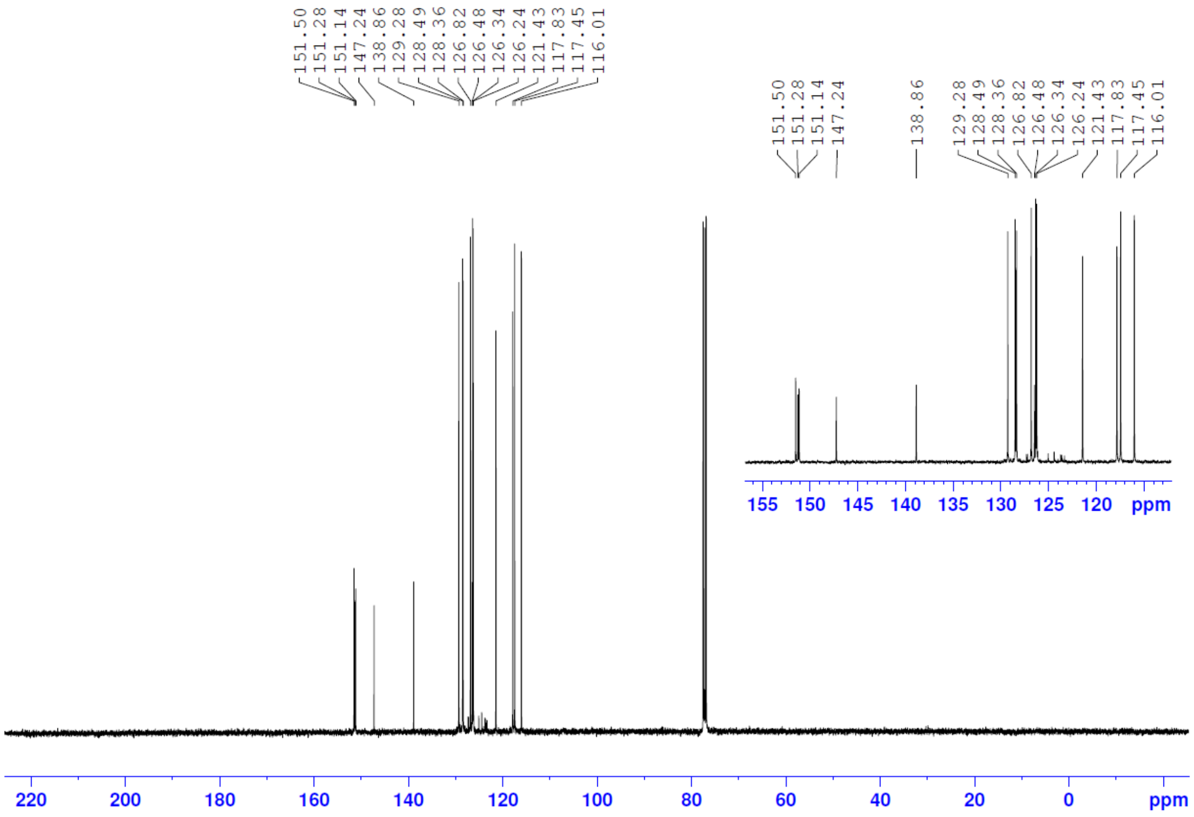

^{13}C NMR (100 MHz) spectrum of **1r** in $CDCl_3$

NMR spectra of 1-phenylbiphenylene (**1e**)

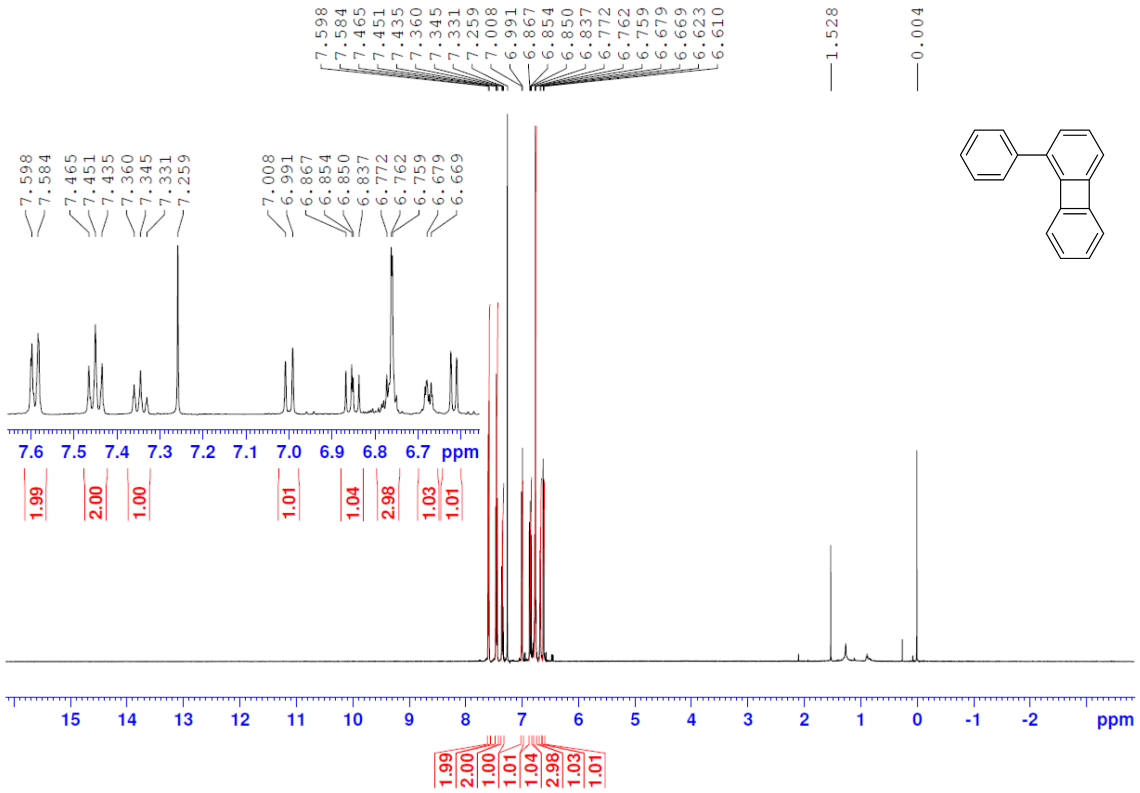

^{1}H NMR (400 MHz) spectrum of **1e** in $CDCl_3$

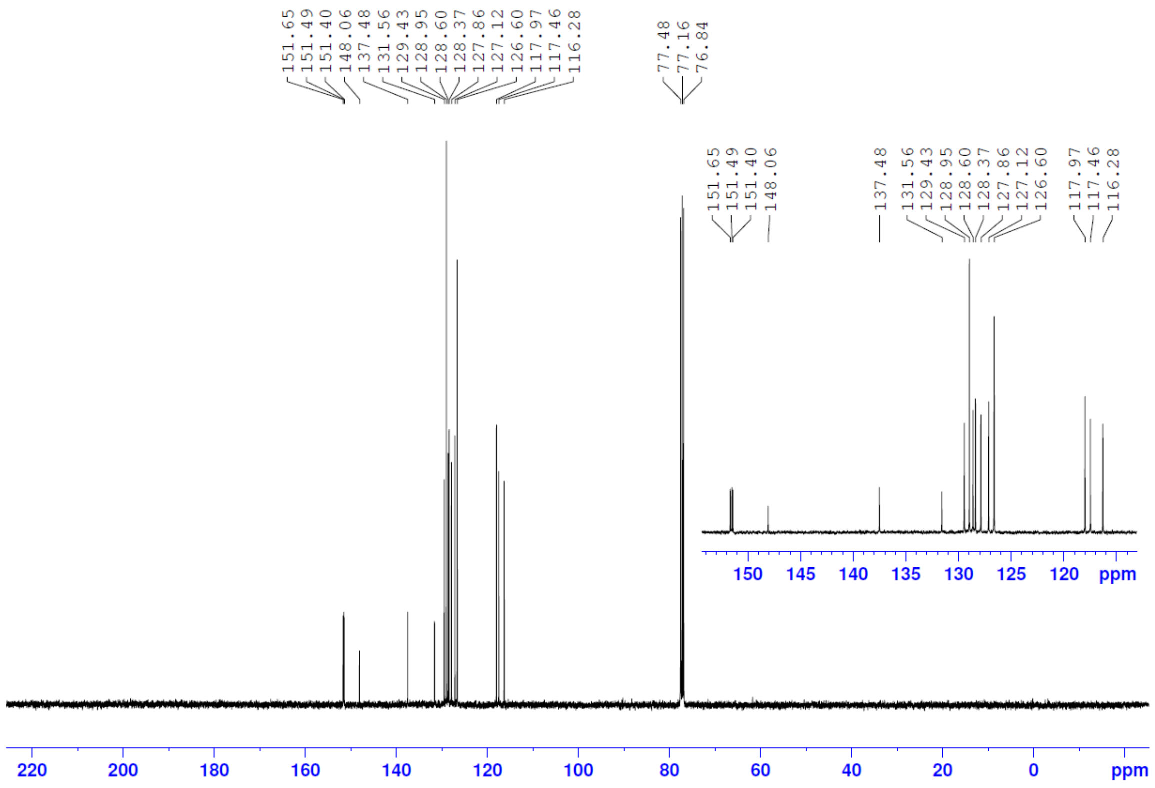

^{13}C NMR (100 MHz) spectrum of **1e** in $CDCl_3$

NMR spectra of 1-(4-tolyl)biphenylene (**1m**)

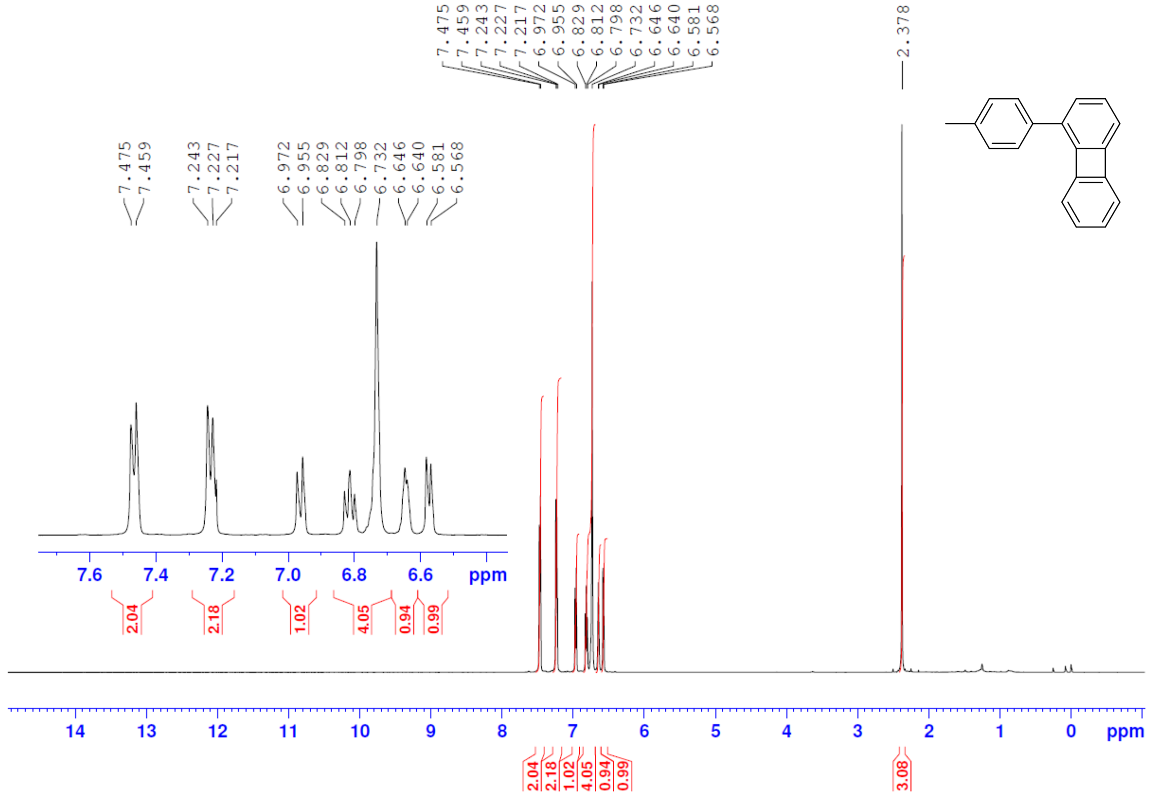

^{1}H NMR (400 MHz) spectrum of **1m** in $CDCl_3$

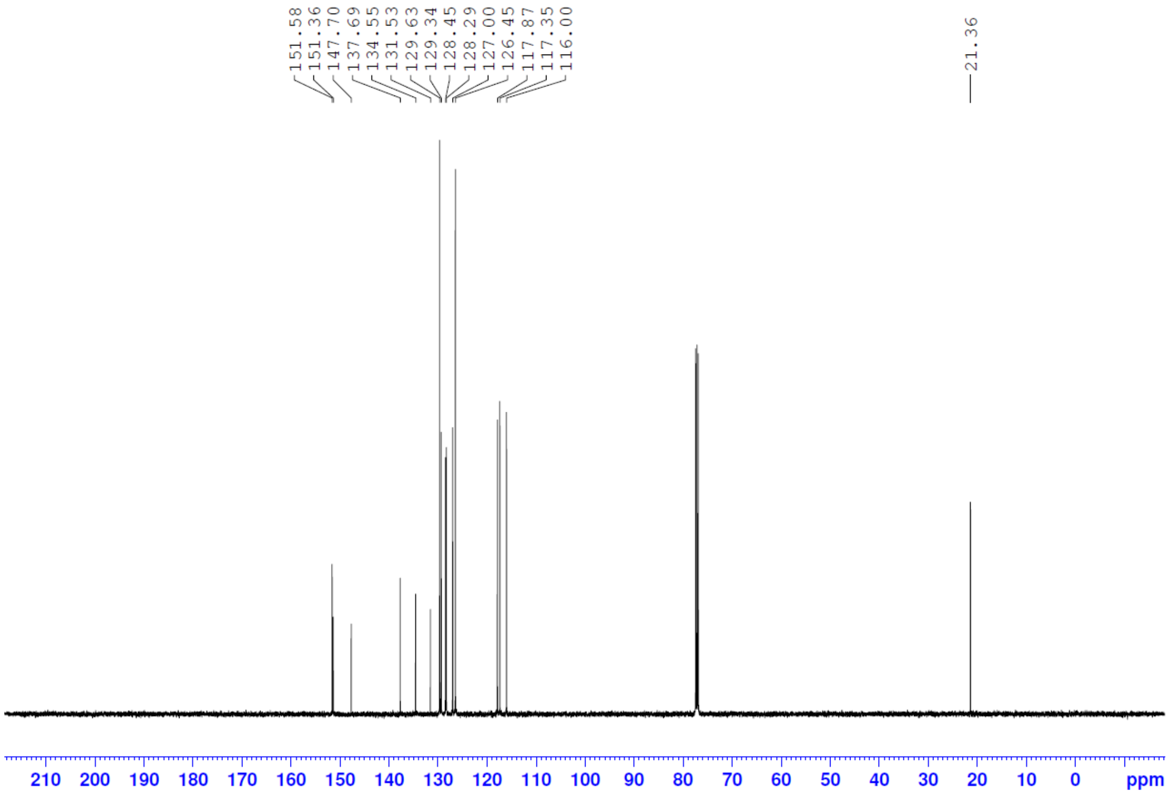

^{13}C NMR (100 MHz) spectrum of **1m** in $CDCl_3$

NMR spectra of 1-(2-methoxyphenyl)biphenylene (**1g**)

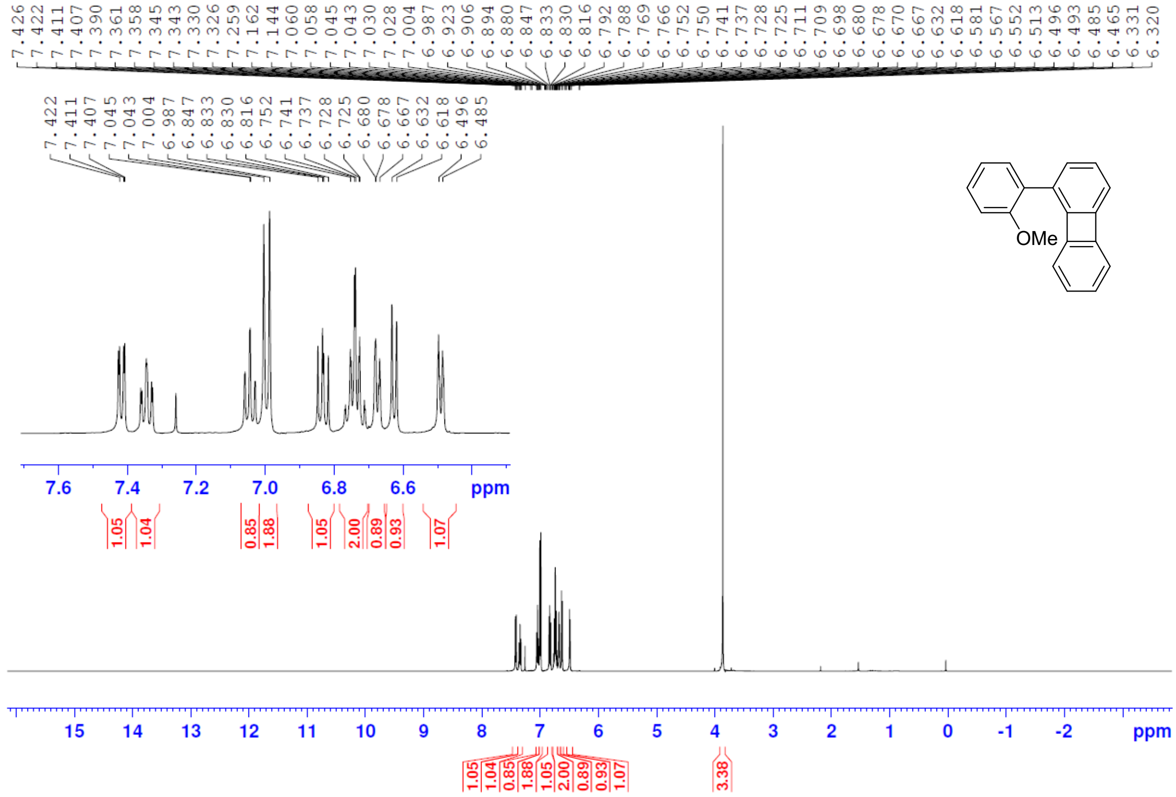

^{1}H NMR (400 MHz) spectrum of **1g** in $CDCl_3$

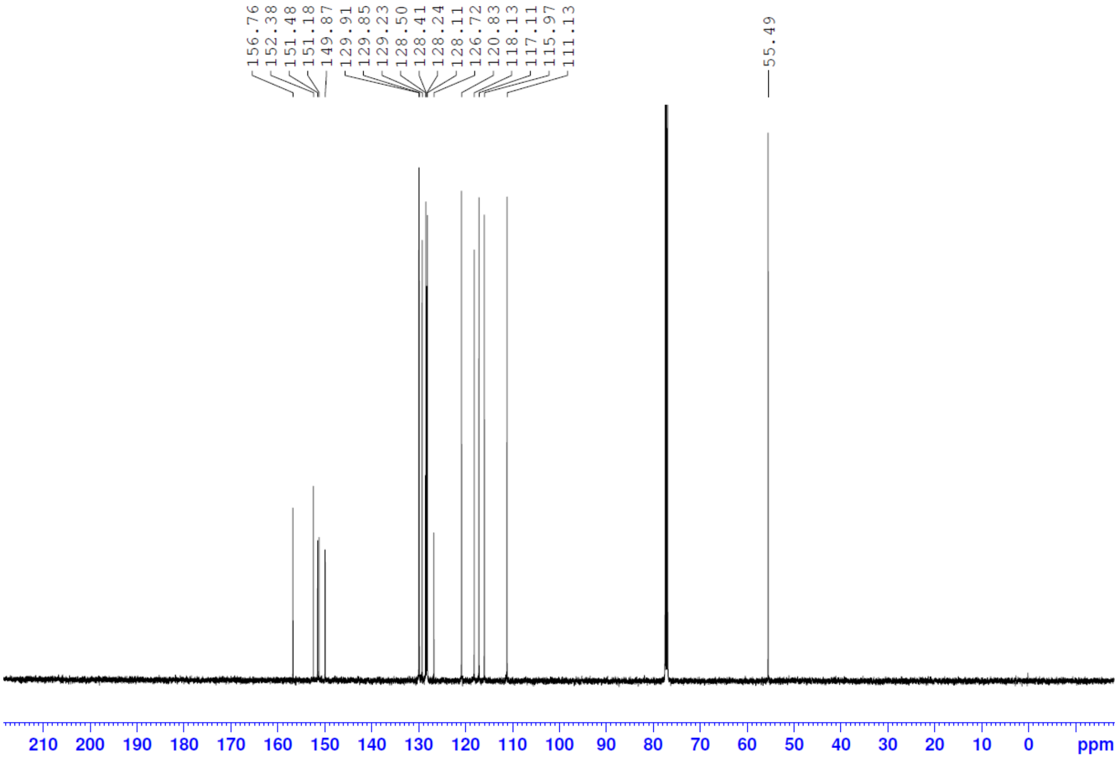

^{13}C NMR (100 MHz) spectrum of **1g** in $CDCl_3$

NMR spectra of 1-(4-methoxyphenyl)biphenylene (**1k**)

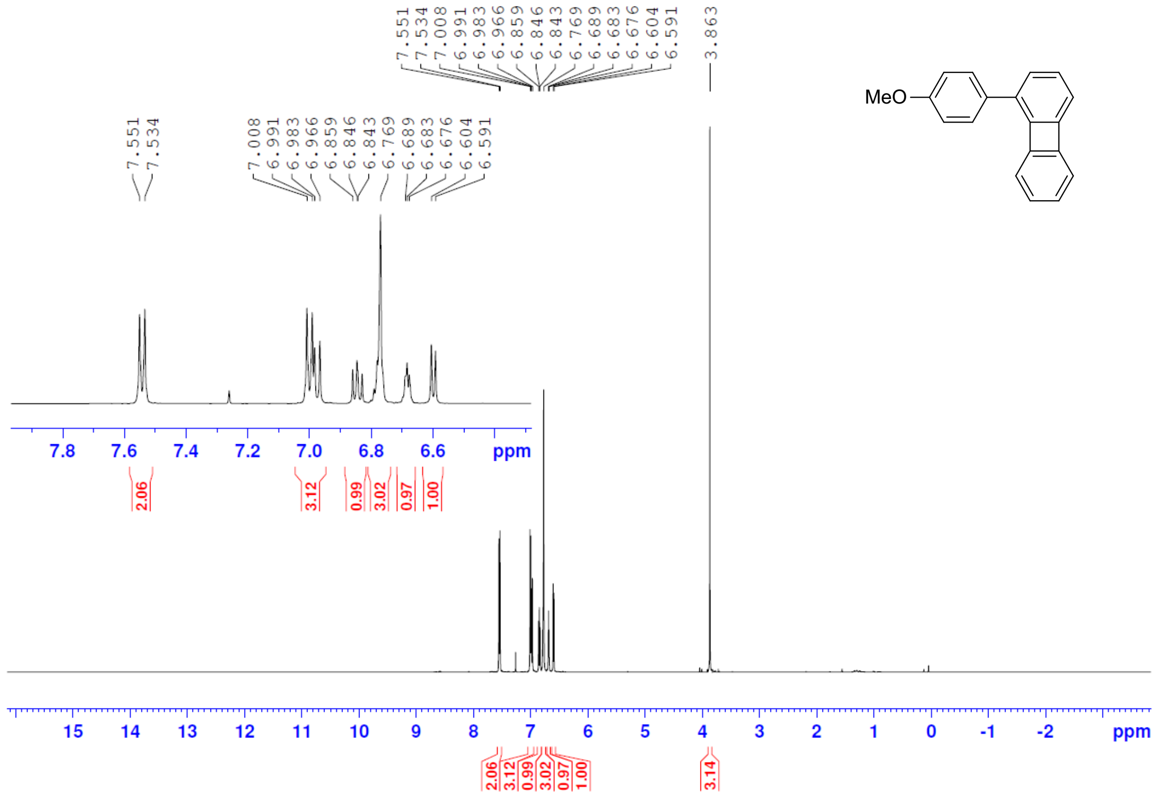

^{1}H NMR (400 MHz) spectrum of **1k** in $CDCl_3$

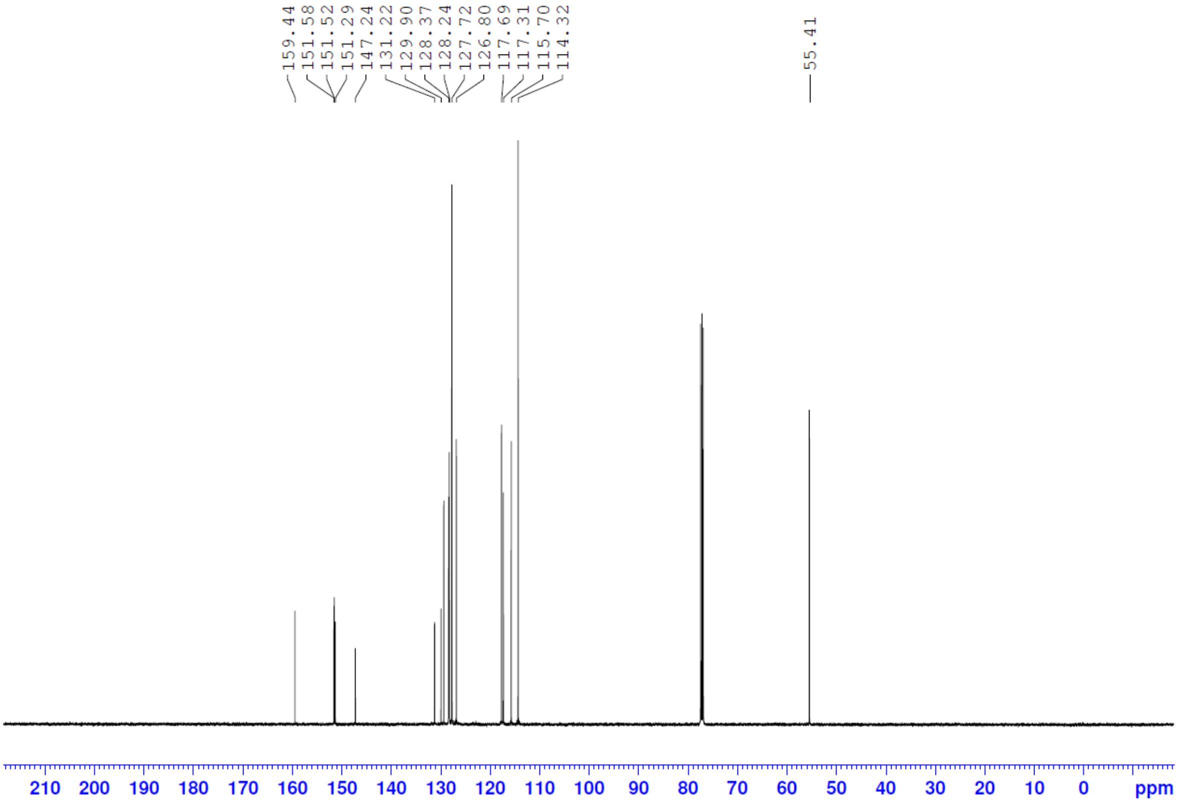

^{13}C NMR (100 MHz) spectrum of **1k** in $CDCl_3$

NMR spectra of 4-(biphenylen-1-yl)-N,N-dimethylaniline (**1l**)

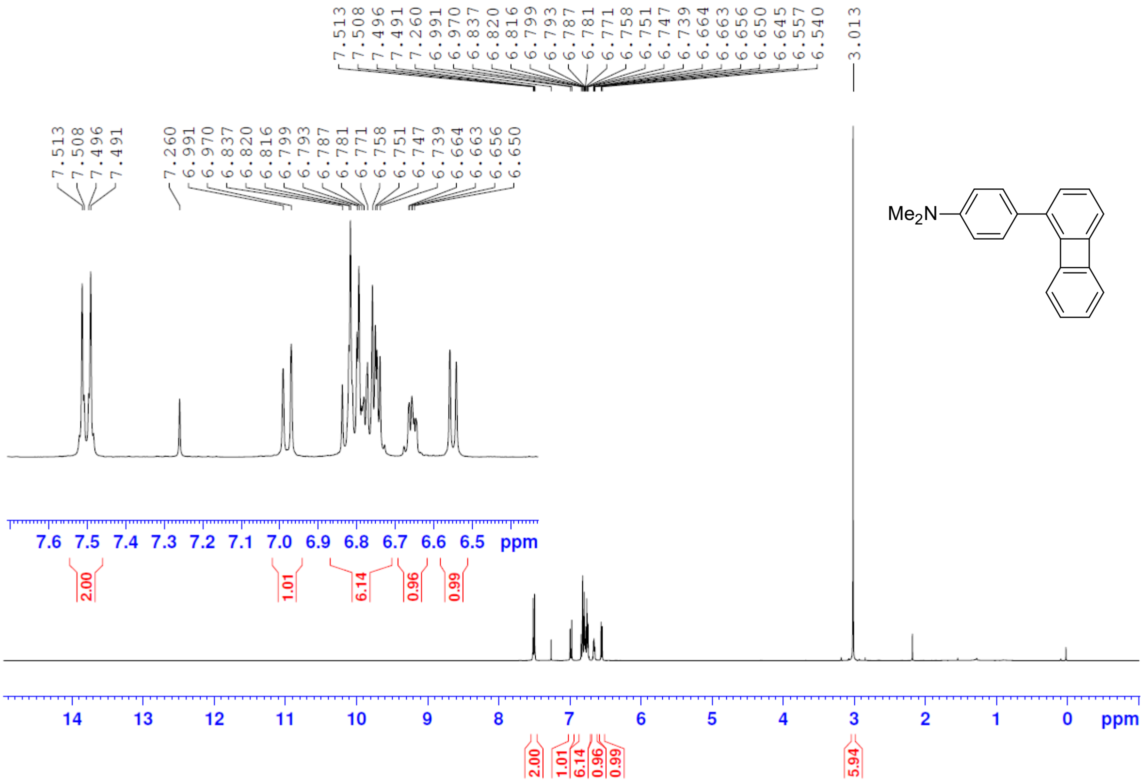

^{1}H NMR (400 MHz) spectrum of **1l** in $CDCl_3$

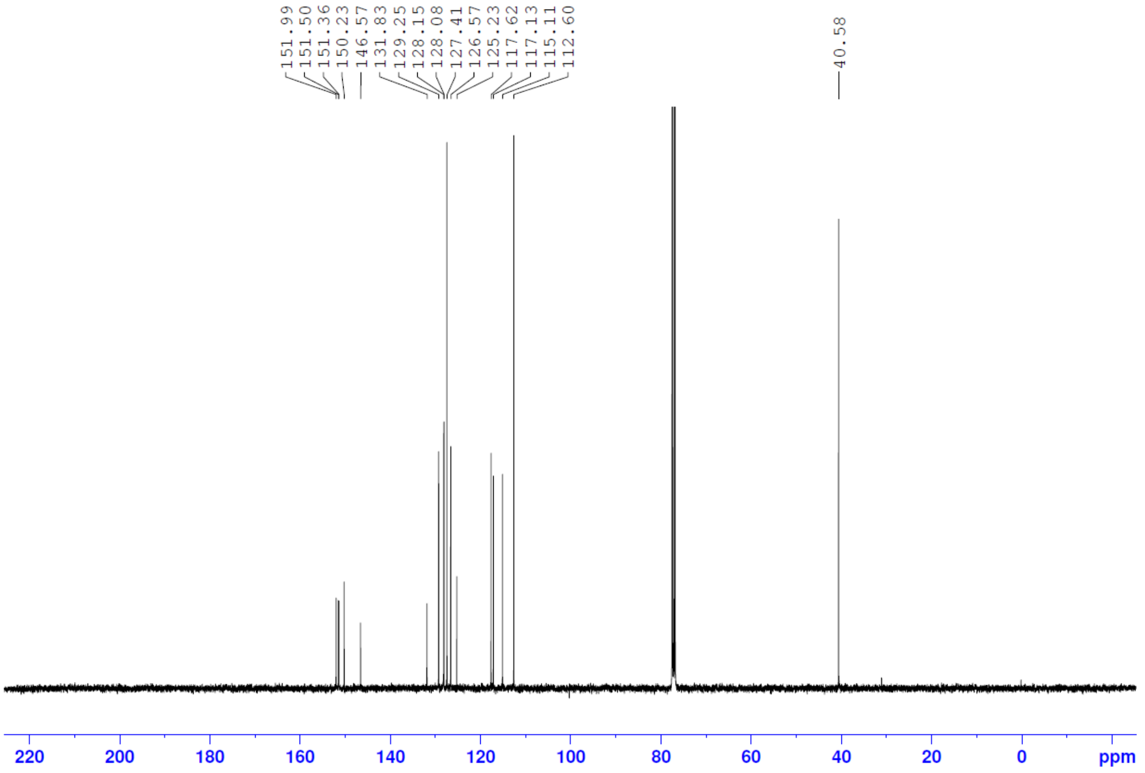

^{13}C NMR (100 MHz) spectrum of **1l** in $CDCl_3$

NMR spectra of 1-(4-(trifluoromethyl)phenyl)biphenylene (**1n**)

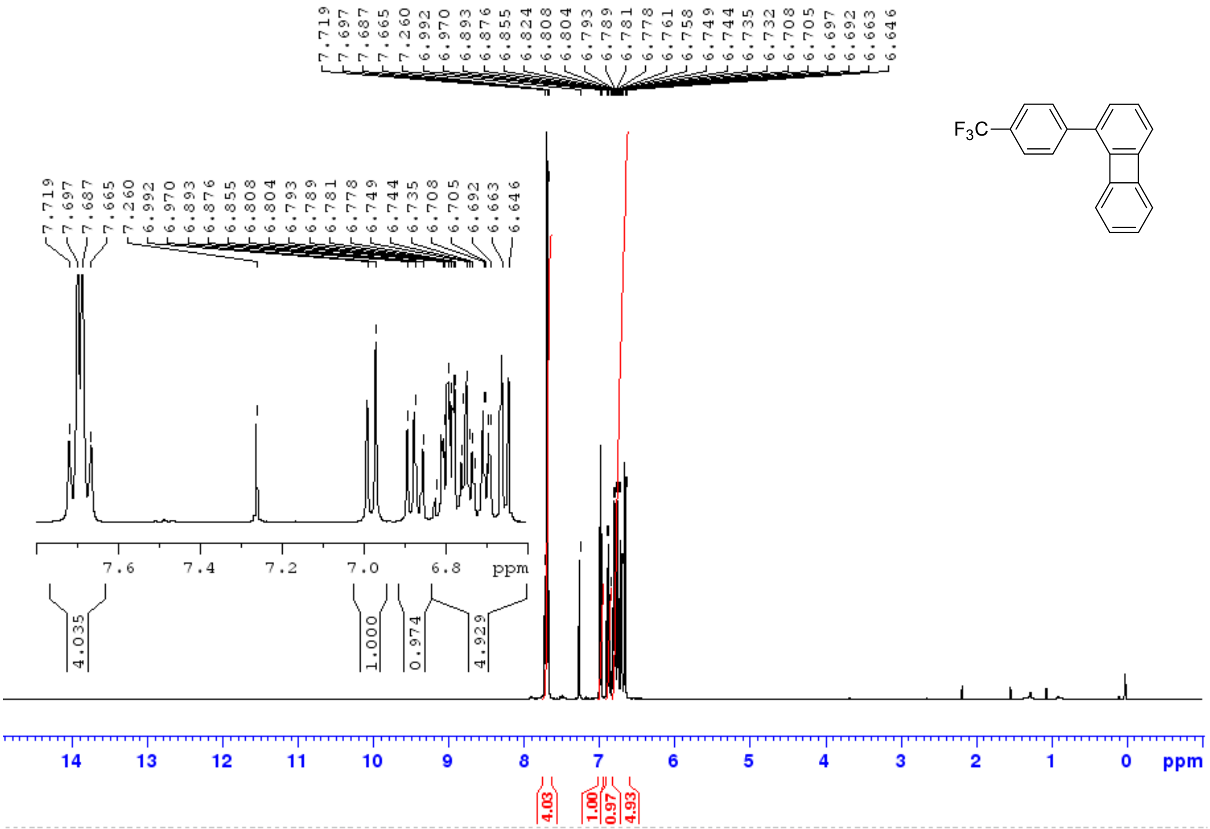

^{1}H NMR (400 MHz) spectrum of **1n** in $CDCl_3$

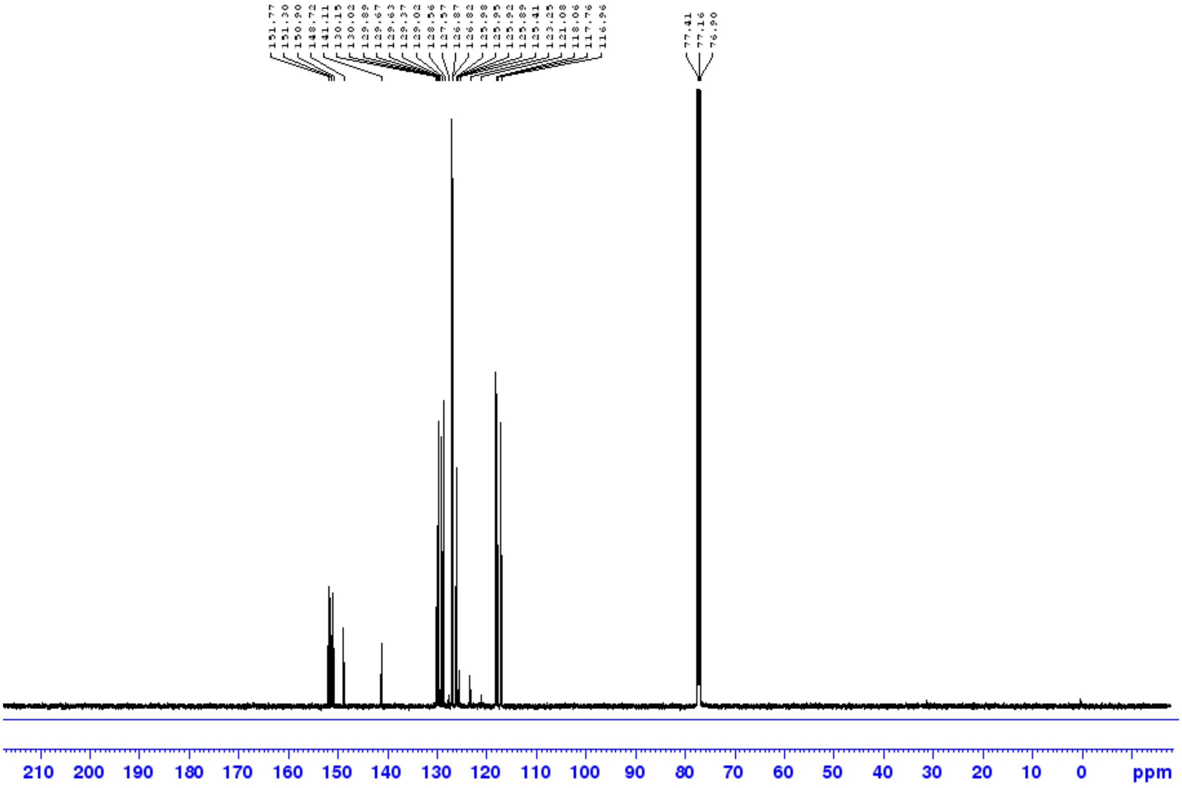

^{13}C NMR (100 MHz) spectrum of **1n** in $CDCl_3$

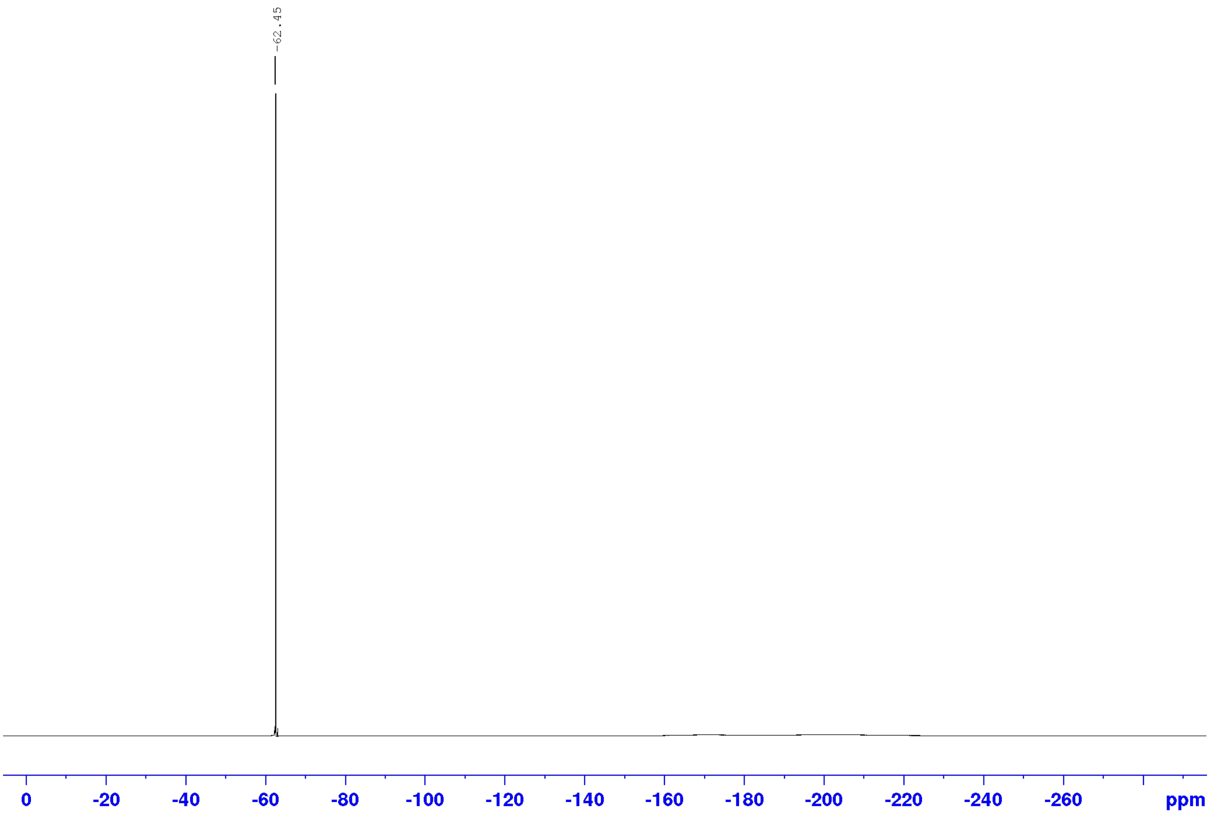

^{19}F NMR (376 MHz) spectrum of **1n** in $CDCl_3$

NMR spectra of 1-(2-tolyl)biphenylene (**1h**)

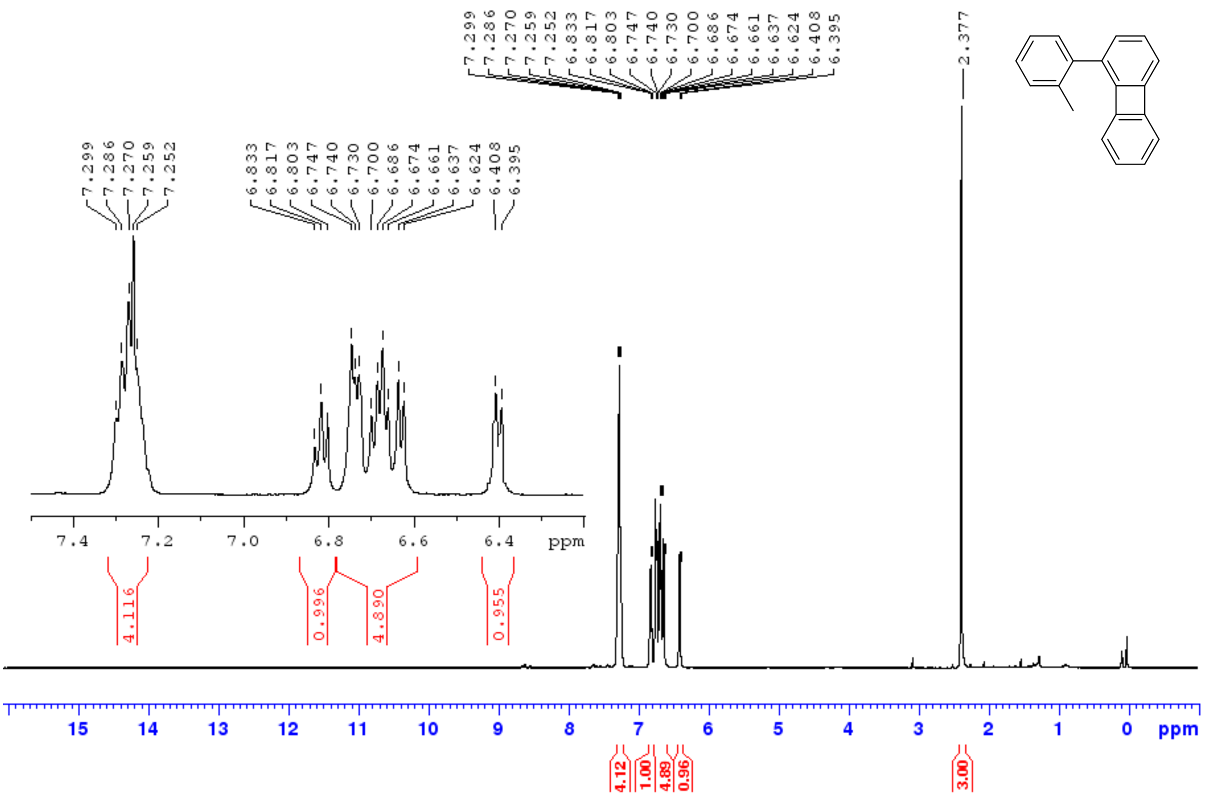

^{1}H NMR (400 MHz) spectrum of **1h** in $CDCl_3$

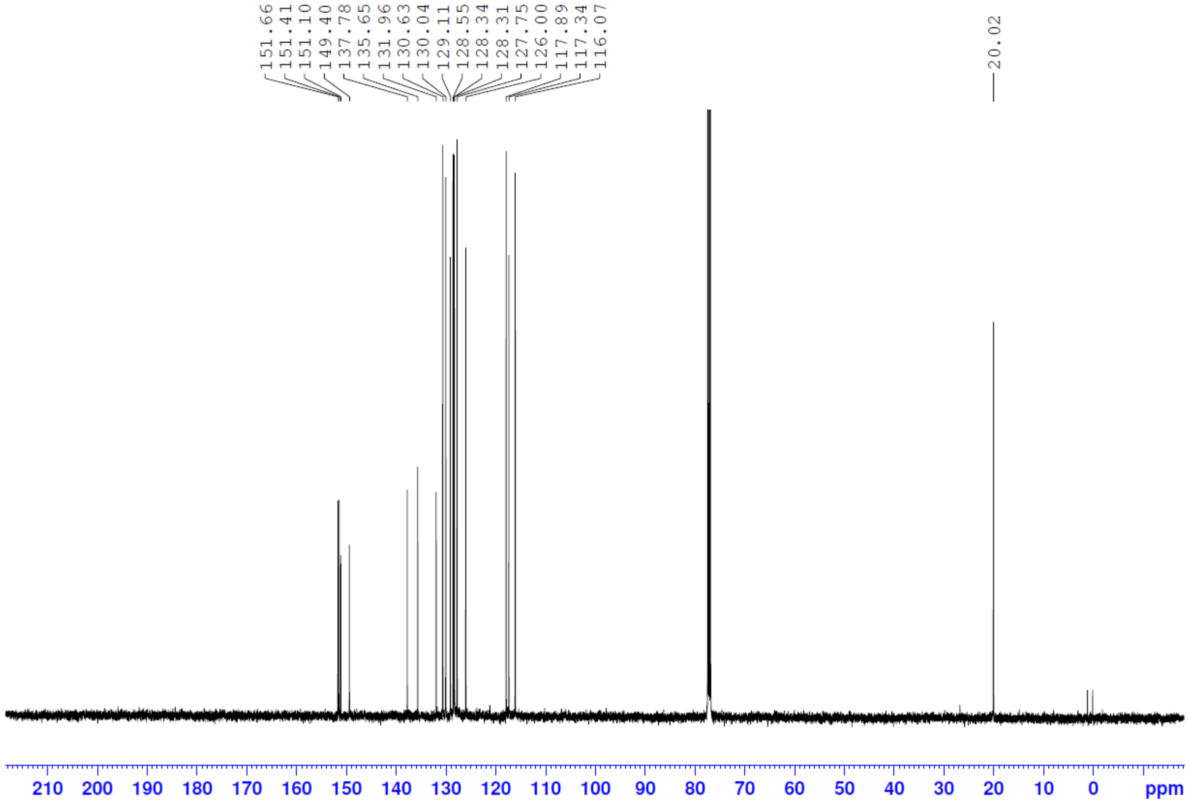

^{13}C NMR (100 MHz) spectrum of **1h** in $CDCl_3$

NMR spectra of 1-(2,6-dimethylphenyl)biphenylene (**1i**)

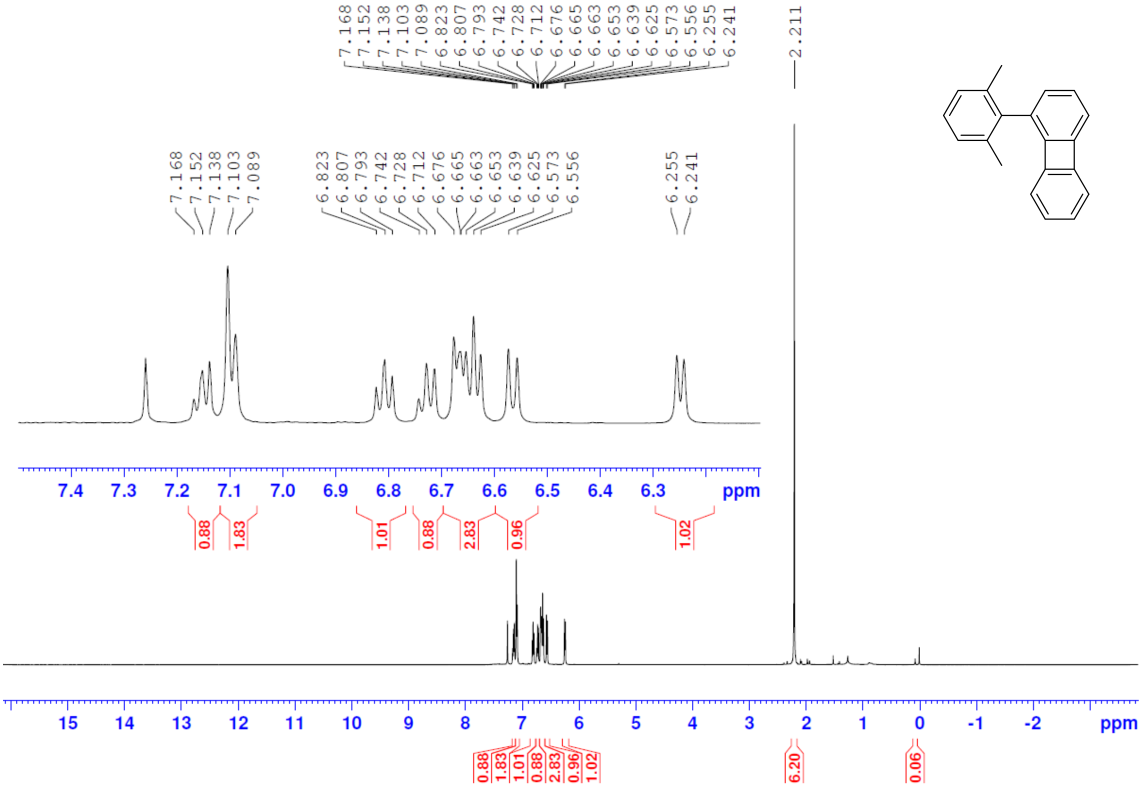

^{1}H NMR (400 MHz) spectrum of **1i** in $CDCl_3$

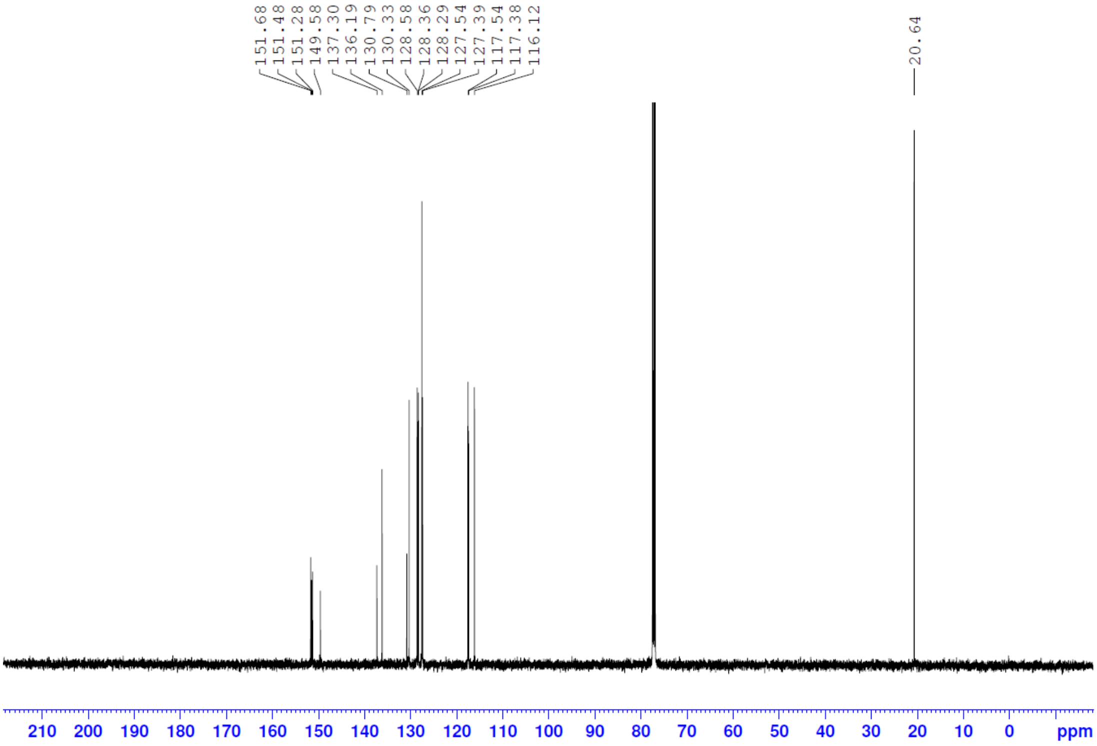

^{13}C NMR (100 MHz) spectrum of **1i** in $CDCl_3$

NMR spectra of 2-(biphenylen-1-yl)pyridine (**1s**)

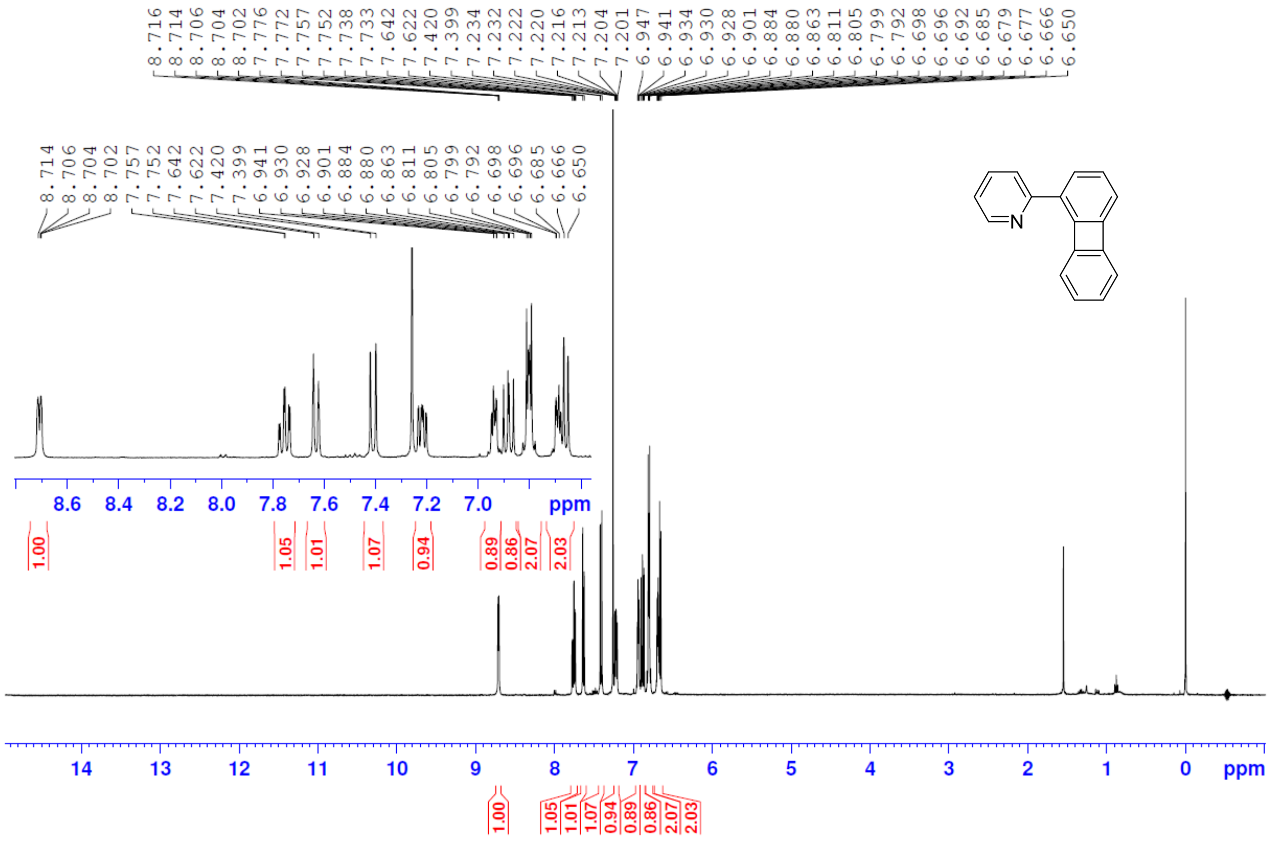

^{1}H NMR (400 MHz) spectrum of **1s** in $CDCl_3$

NMR spectra of 1-(naphthalen-1-yl)biphenylene (**1f**)

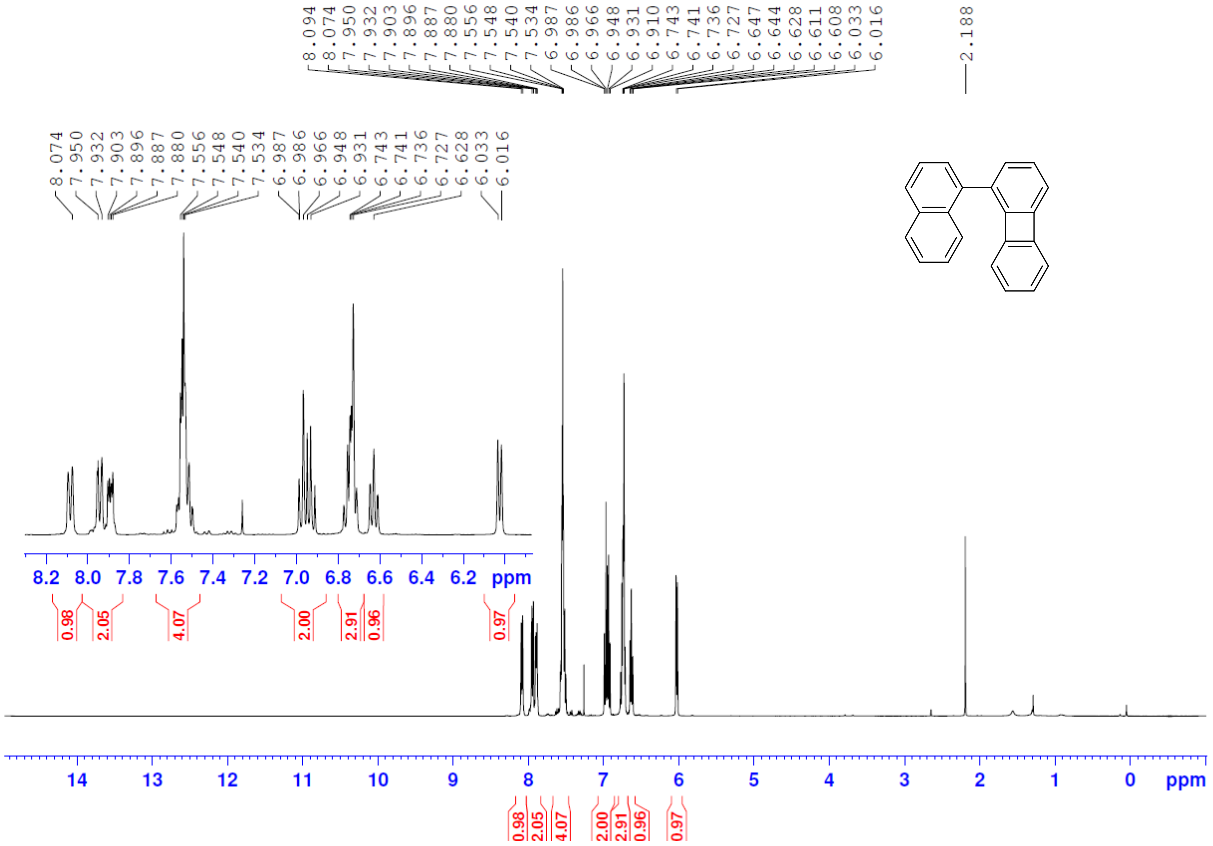

^{1}H NMR (400 MHz) spectrum of **1f** in $CDCl_3$

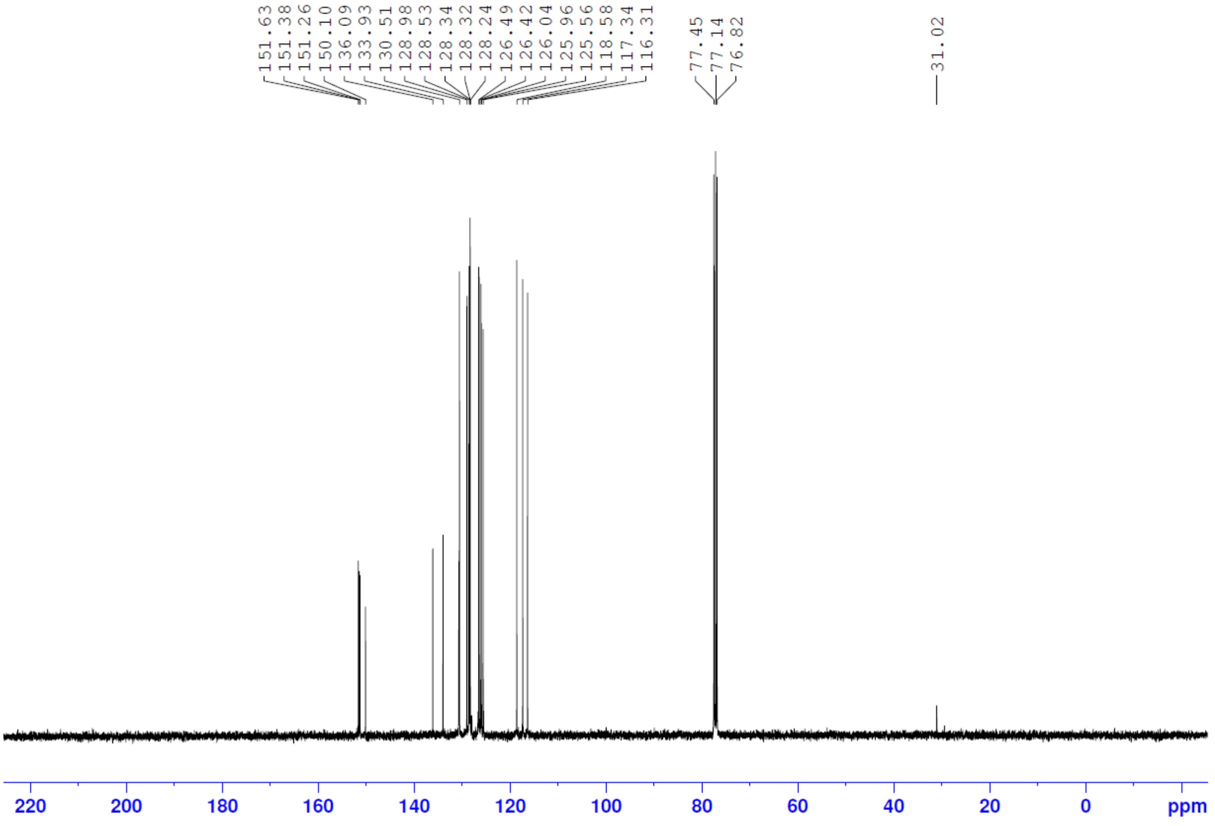

^{13}C NMR (100 MHz) spectrum of **1f** in $CDCl_3$

NMR spectra of 1-(2-isopropylphenyl)biphenylene (**1j**)

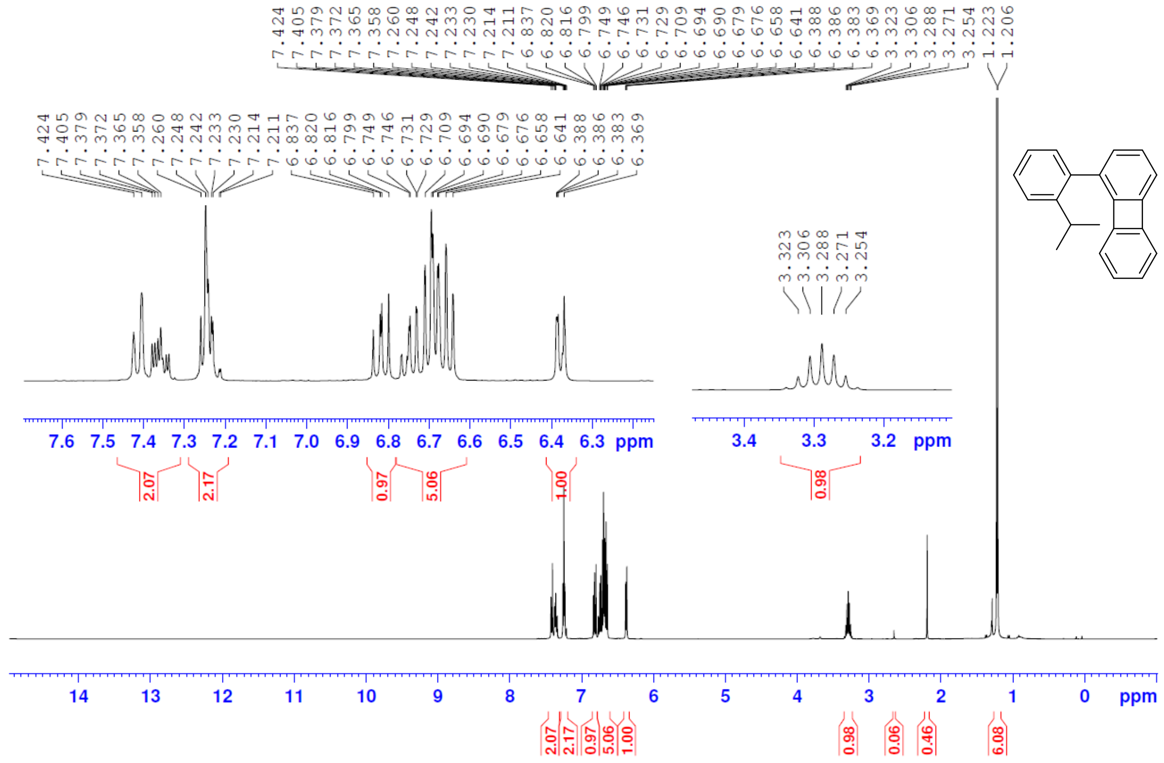

^{1}H NMR (400 MHz) spectrum of **1j** in $CDCl_3$

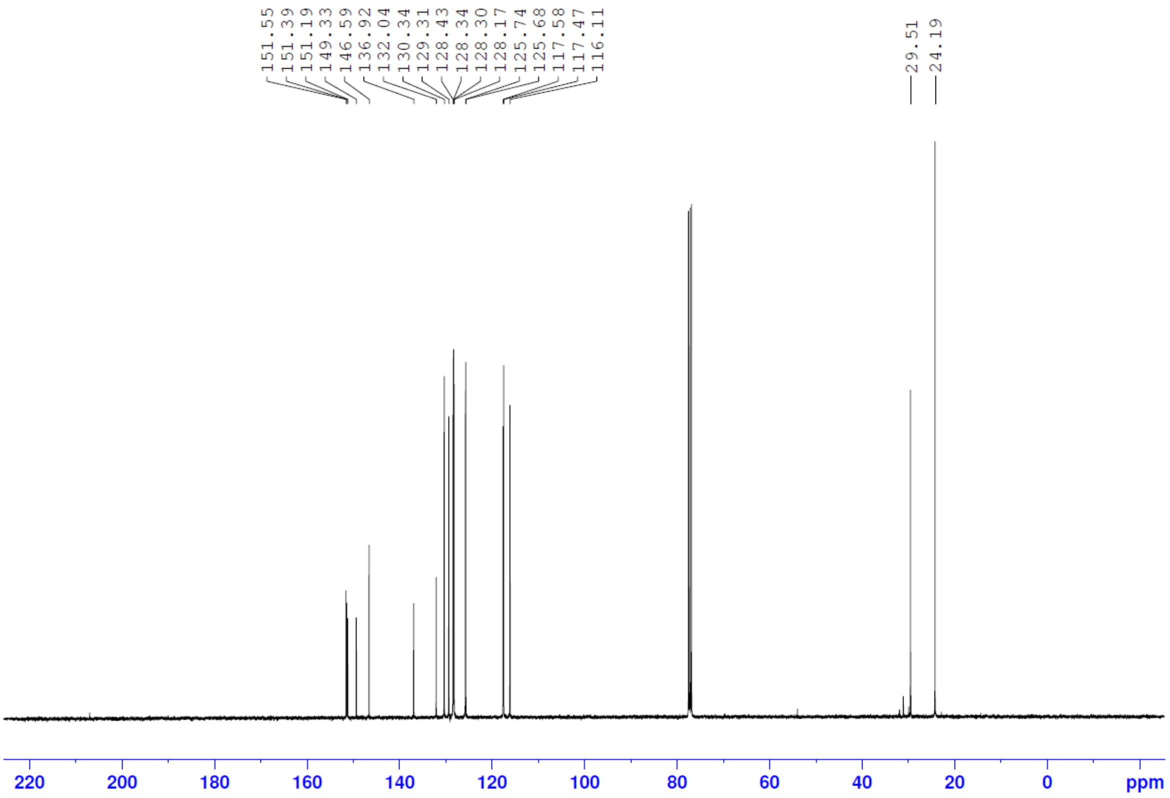

^{13}C NMR (100 MHz) spectrum of **1j** in $CDCl_3$

NMR spectra of **2a** (both regioisomers)

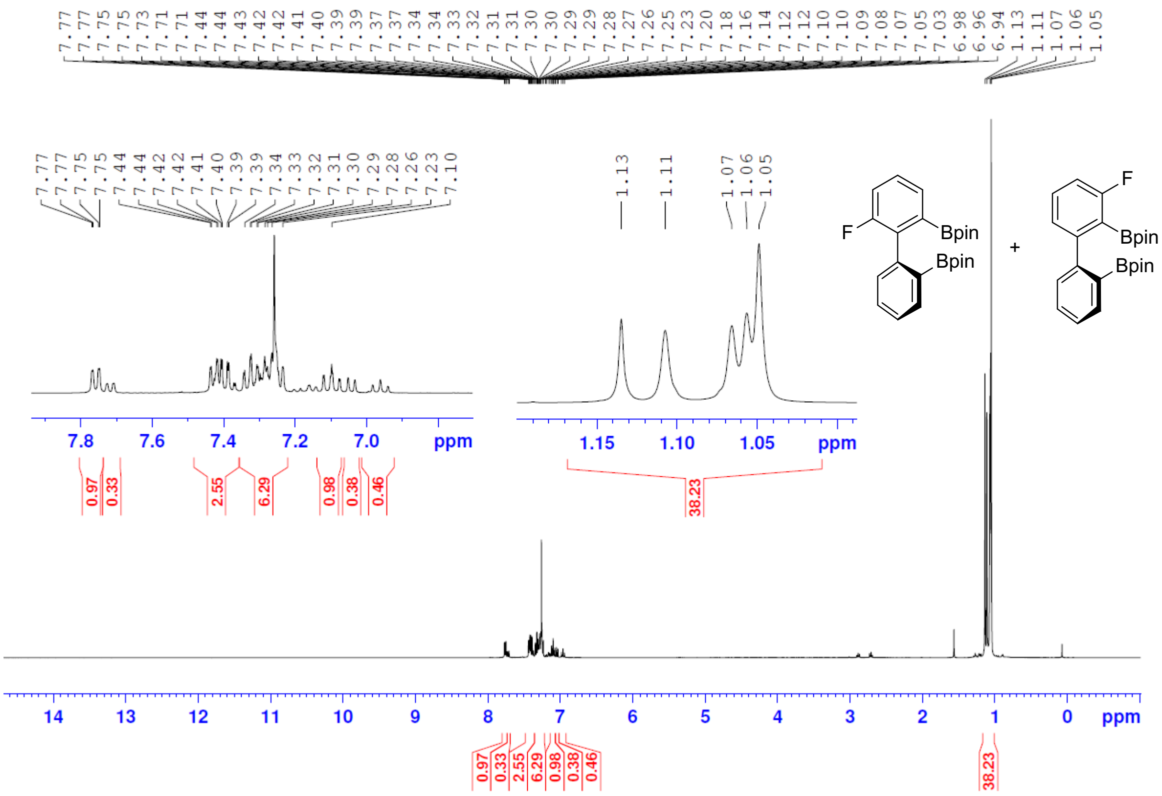

^{1}H NMR (400 MHz) spectrum of **2a** (both regioisomers) in $CDCl_3$

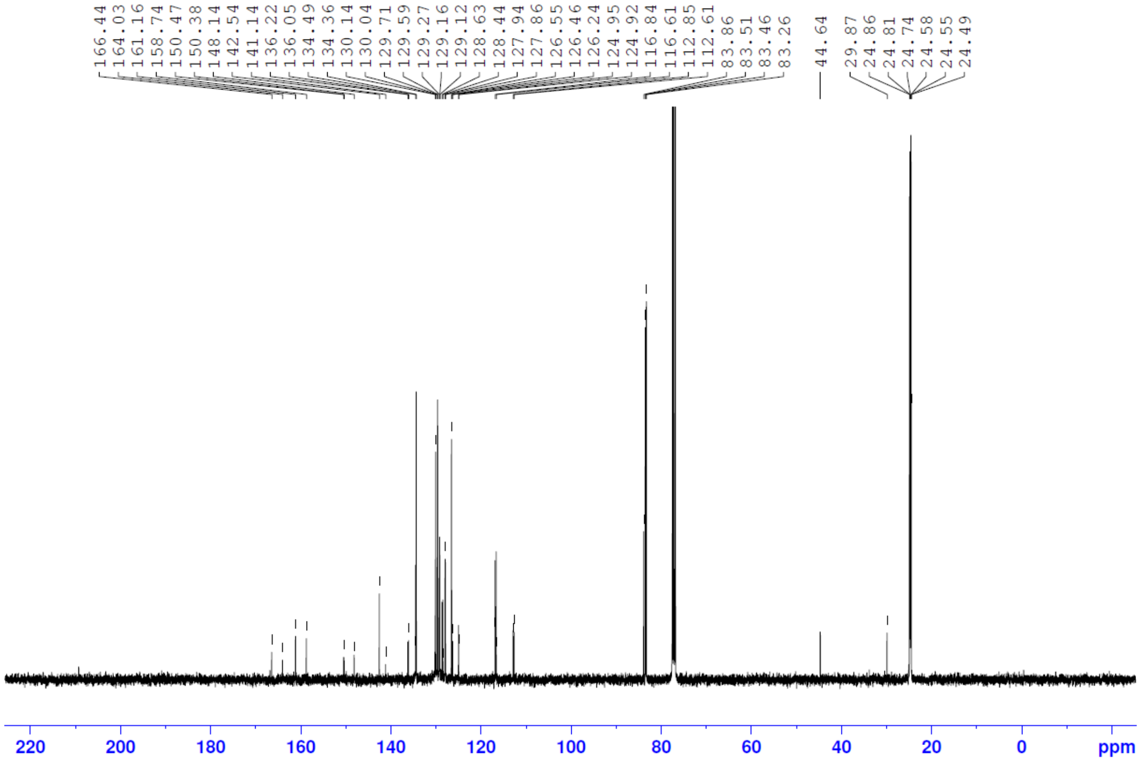

^{13}C NMR (100 MHz) spectrum of **2a** (both regioisomers) in $CDCl_3$

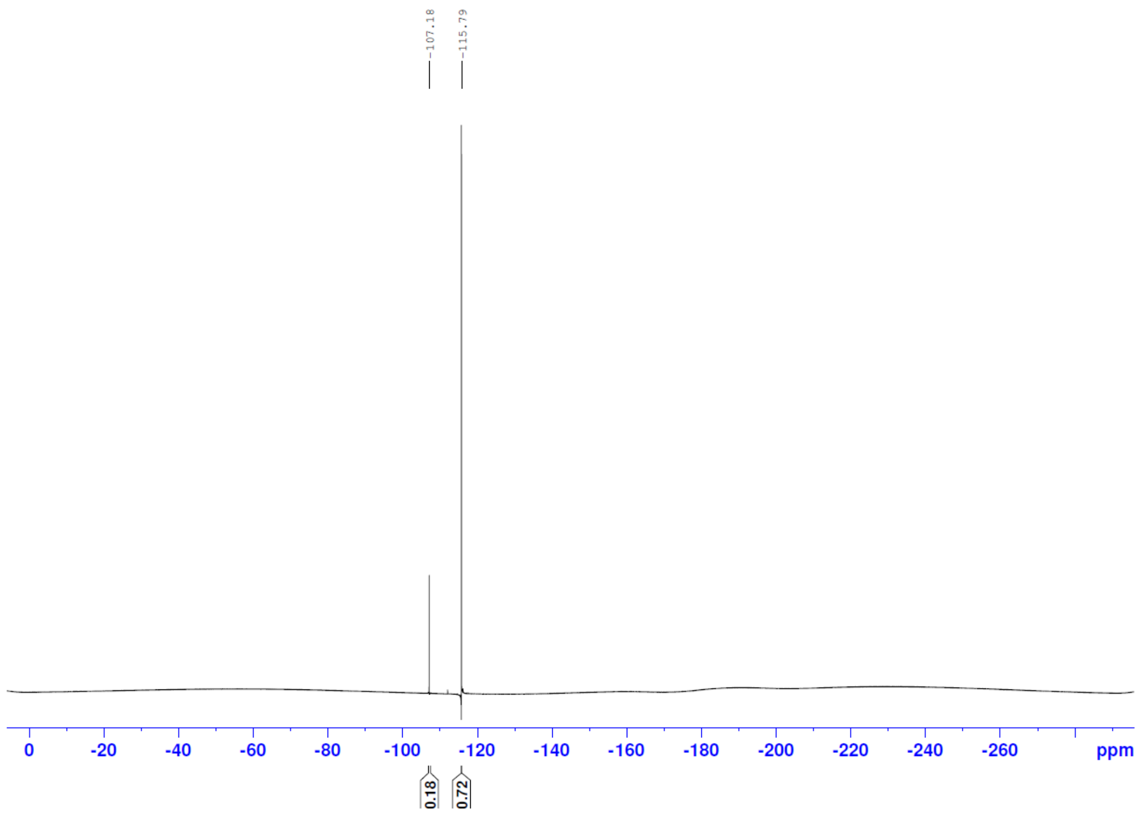

^{19}F NMR (376 MHz) spectrum of **2a** (both regioisomers) in $CDCl_3$

NMR spectra of **2c**

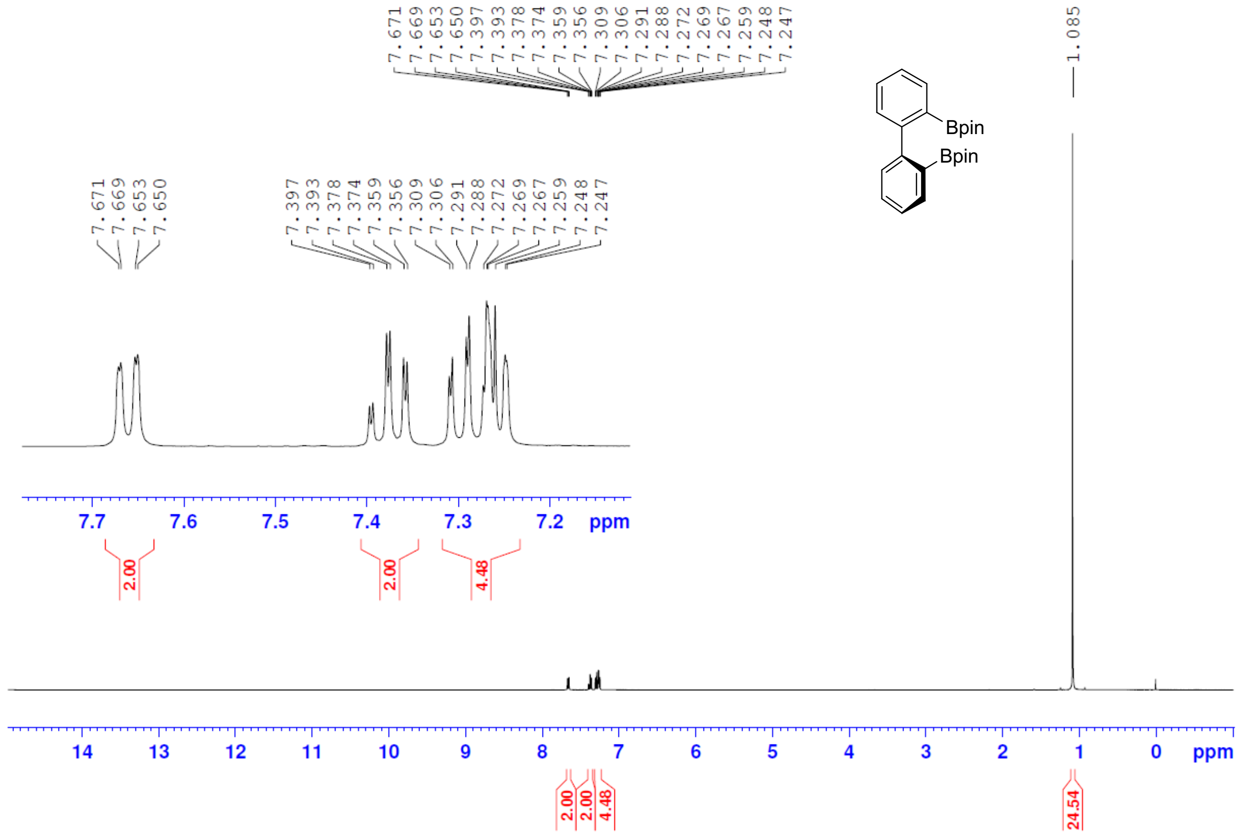

^{1}H NMR (400 MHz) spectrum of **2c** in $CDCl_3$

NMR spectra of **2d** (major regioisomer)

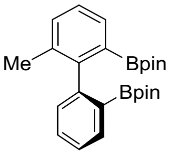

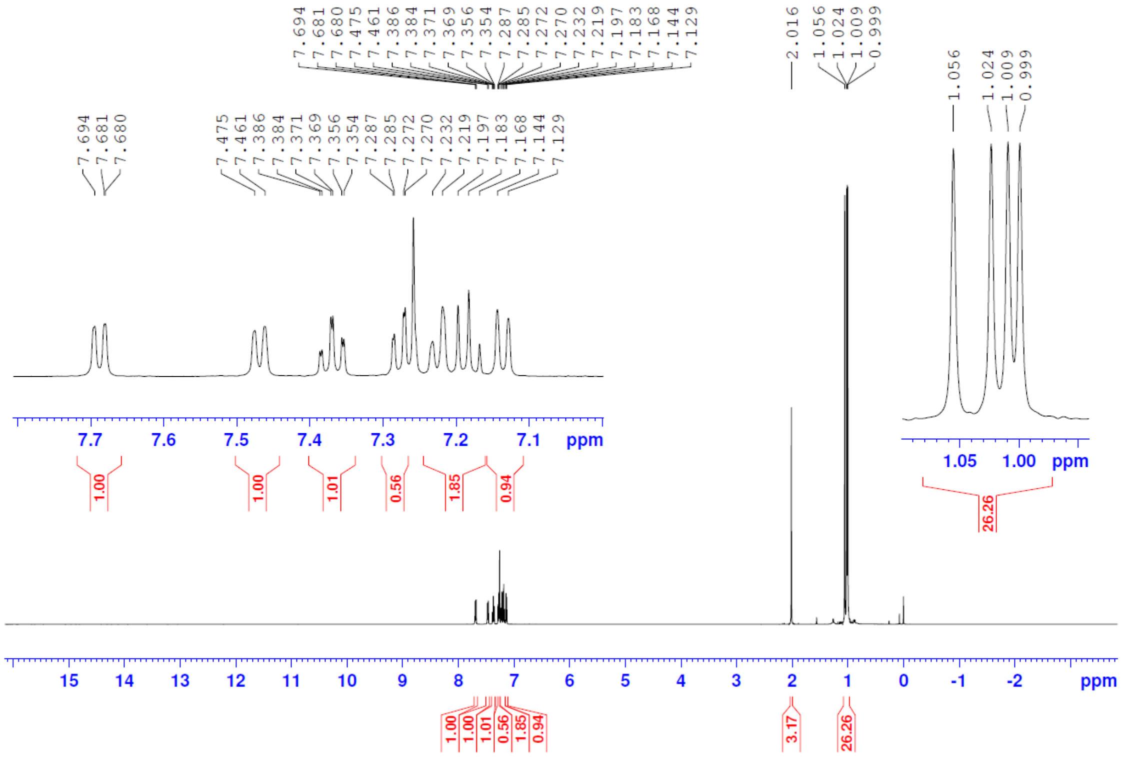

^{1}H NMR (400 MHz) spectrum of **2d** (major regioisomer) in $CDCl_3$

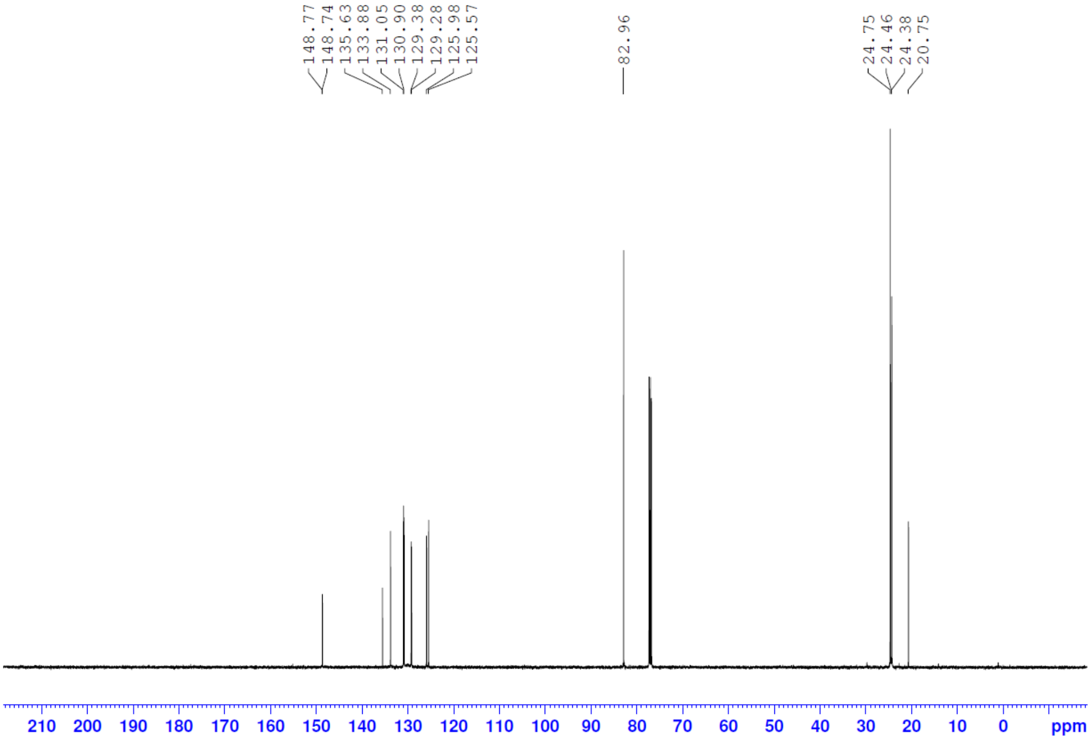

^{13}C NMR (100 MHz) spectrum of **2d** (major regioisomer) in $CDCl_3$

NMR spectra of **2e** (major regioisomer)

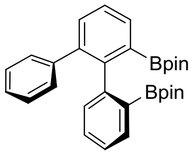

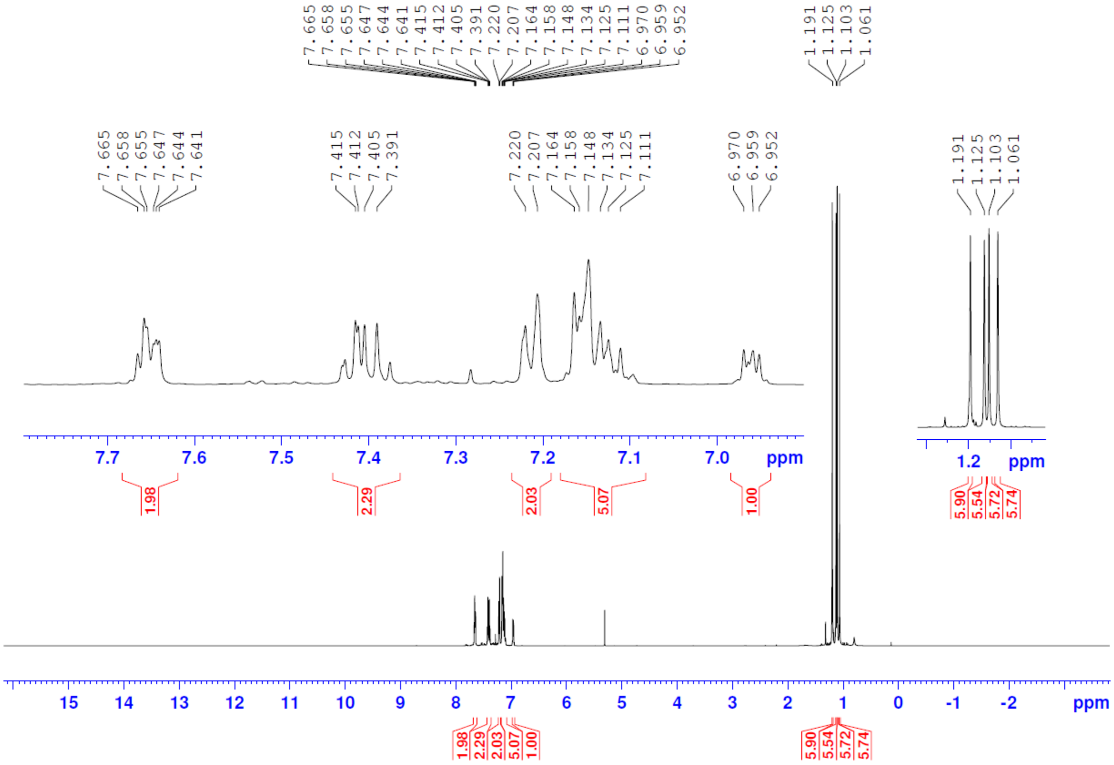

^{1}H NMR (400 MHz) spectrum of **2e** (major regioisomer) in $CDCl_3$

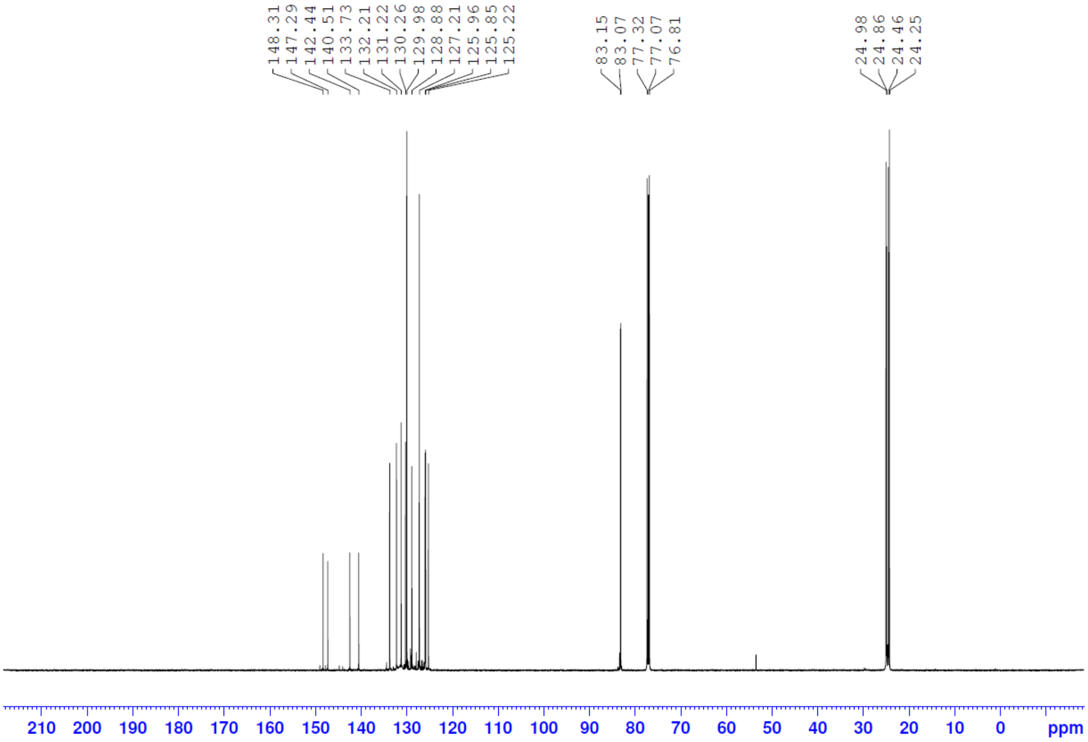

^{13}C NMR (100 MHz) spectrum of **2e** (major regioisomer) in $CDCl_3$

NMR spectra of **2e** (minor regioisomer)

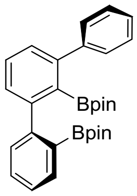

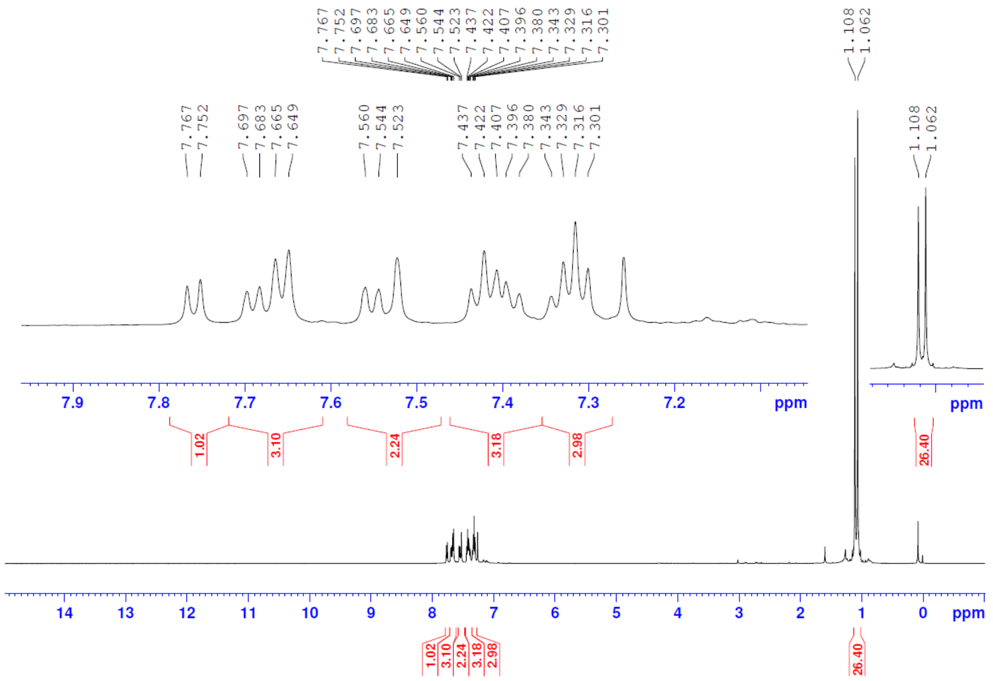

^{1}H NMR (400 MHz) spectrum of **2e** (minor regioisomer) in $CDCl_3$

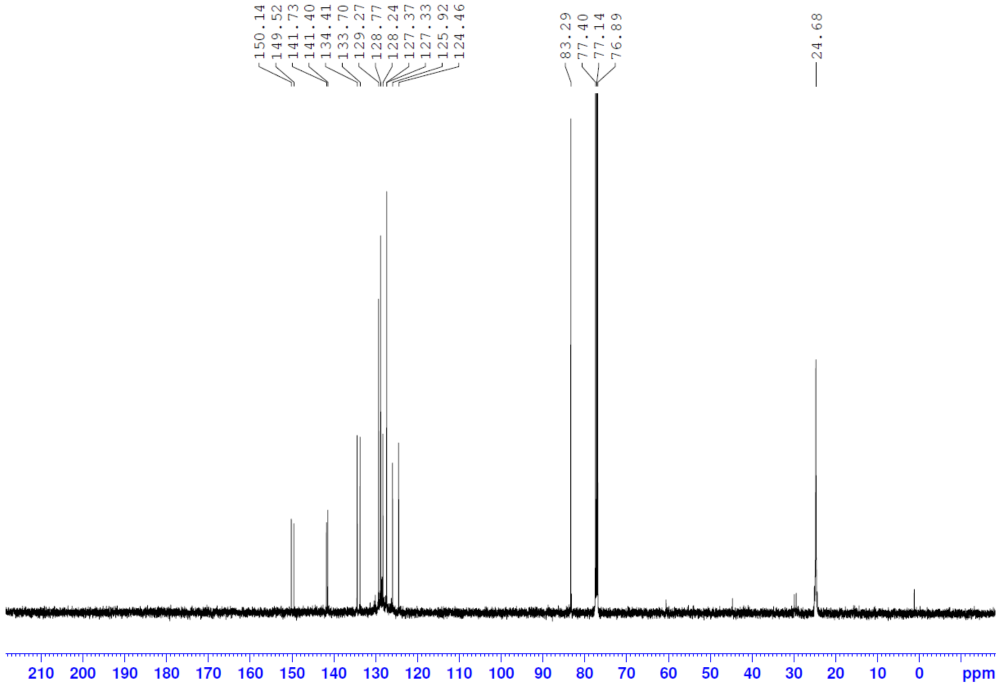

^{13}C NMR (100 MHz) spectrum of **2e** (minor regioisomer) in $CDCl_3$

NMR spectra of **2f** (major regioisomer)

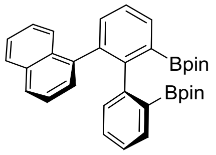

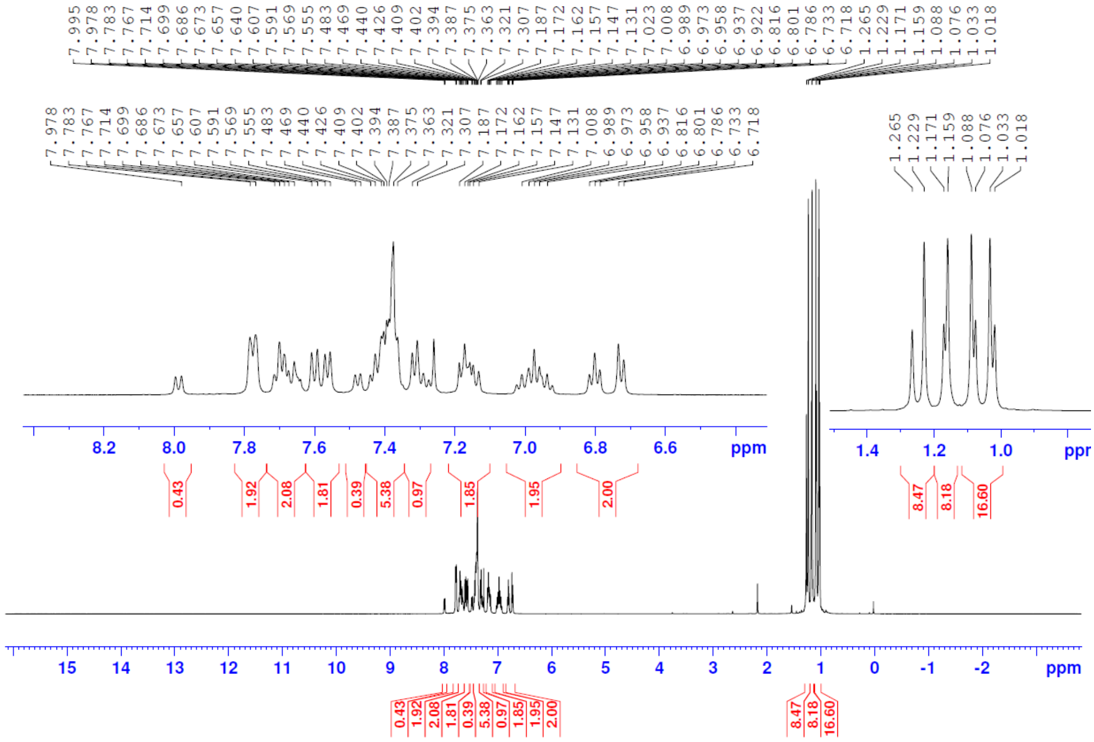

^{1}H NMR (400 MHz) spectrum of **2f** (major regioisomer) in $CDCl_3$

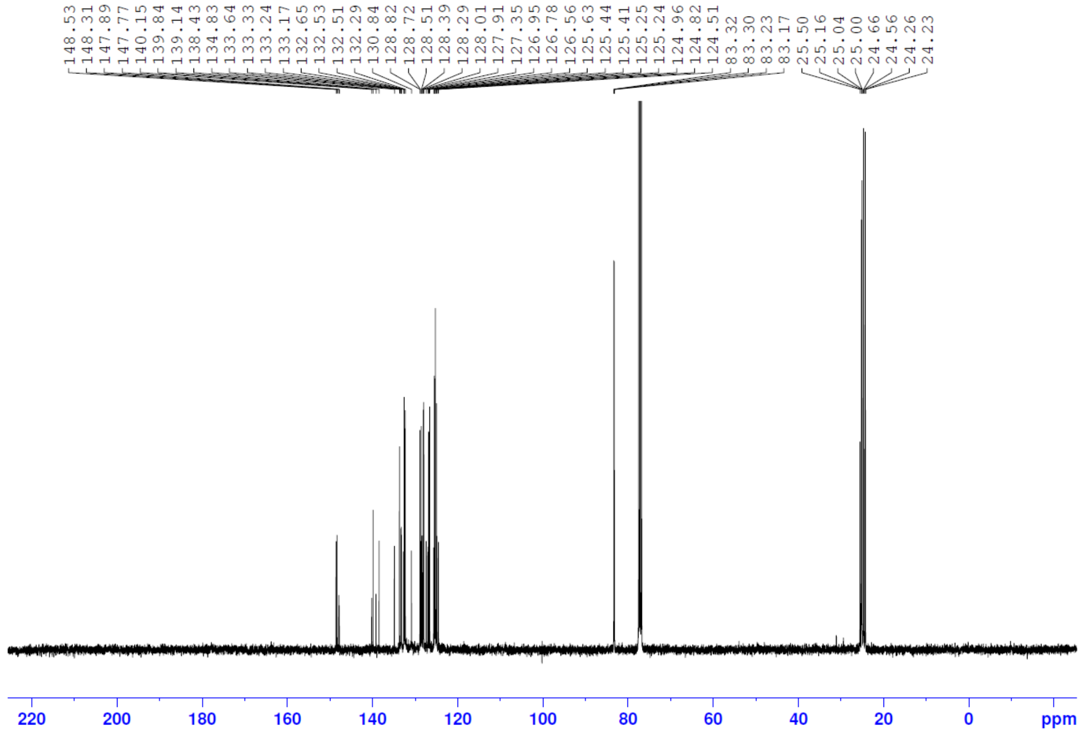

^{13}C NMR (100 MHz) spectrum of **2f** (major regioisomer) in $CDCl_3$

NMR spectra of **2f** (minor regioisomer)

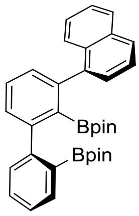

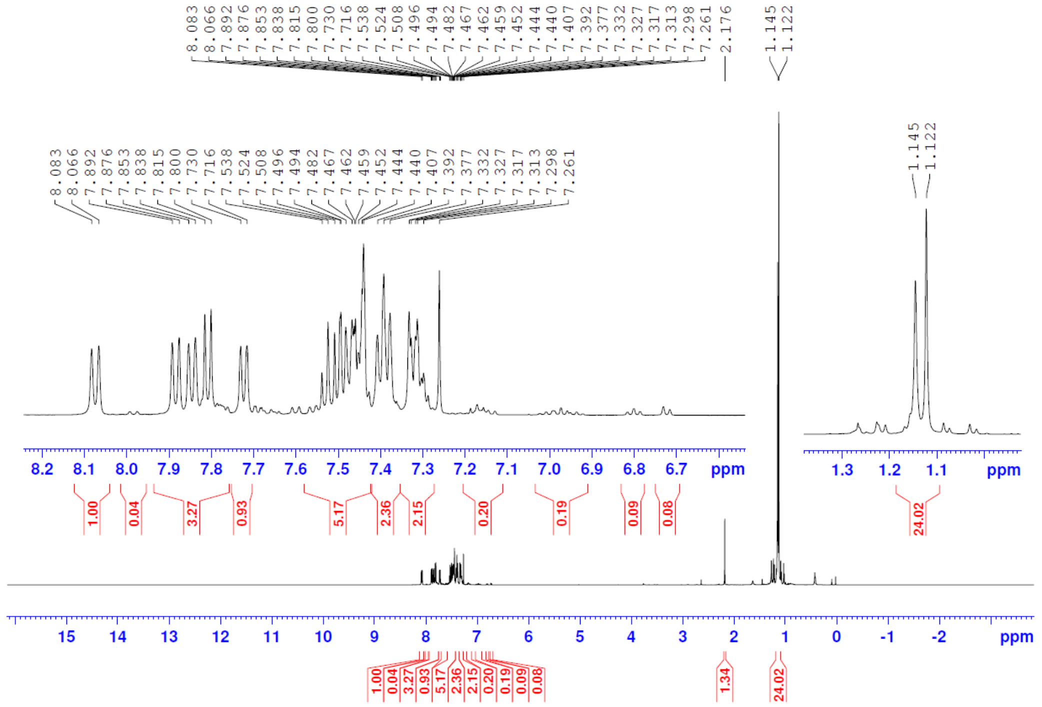

^{1}H NMR (400 MHz) spectrum of **2f** (minor regioisomer) in $CDCl_3$

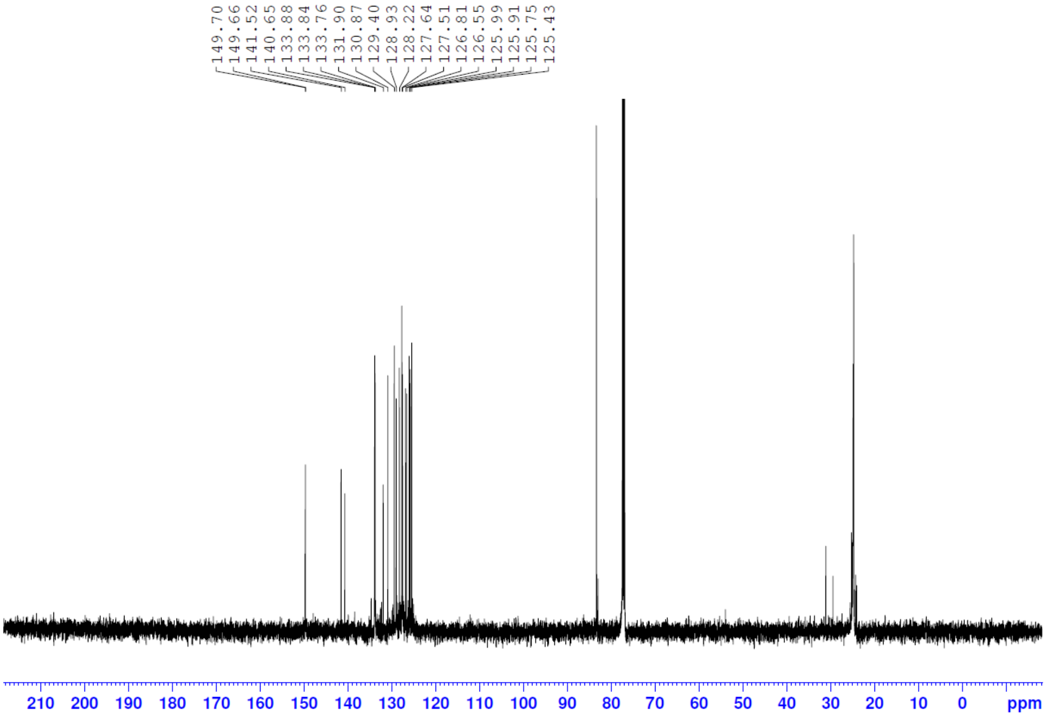

^{13}C NMR (100 MHz) spectrum of **2f** (minor regioisomer) in $CDCl_3$

NMR spectra of **2g** (major regioisomer)

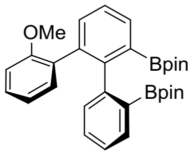

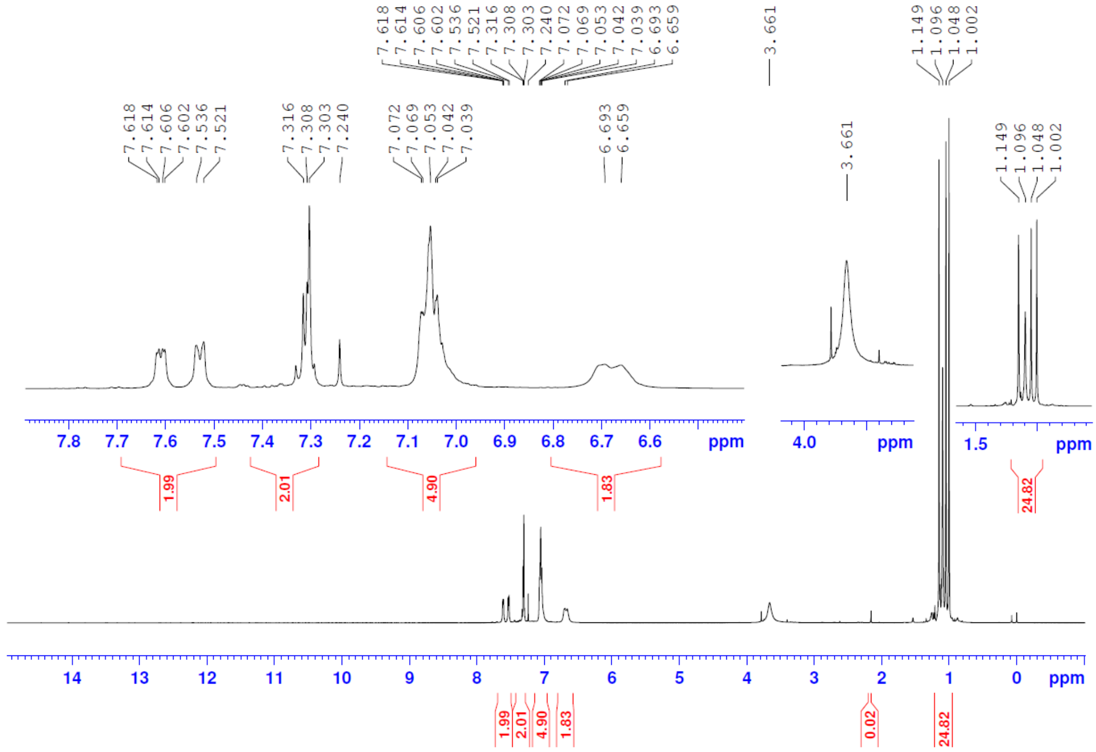

^{1}H NMR (400 MHz) spectrum of **2g** (major regioisomer) in $CDCl_3$

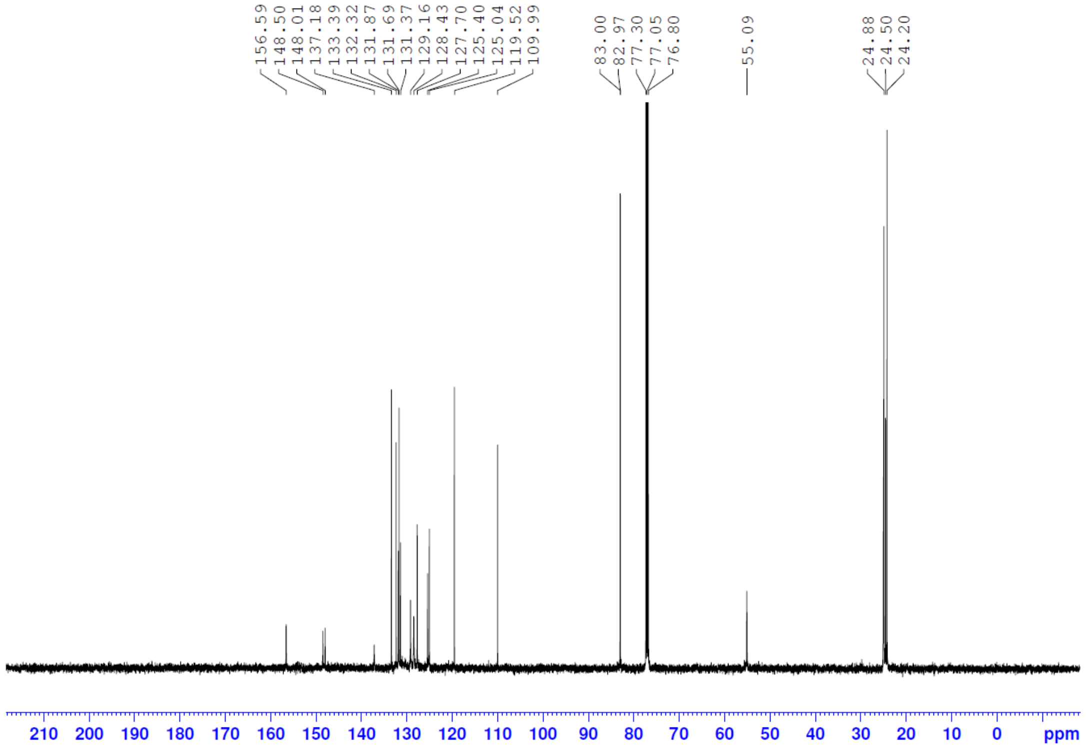

^{13}C NMR (100 MHz) spectrum of **2g** (major regioisomer) in $CDCl_3$

NMR spectra of **2g** (minor regioisomer)

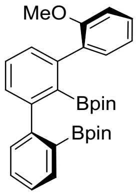

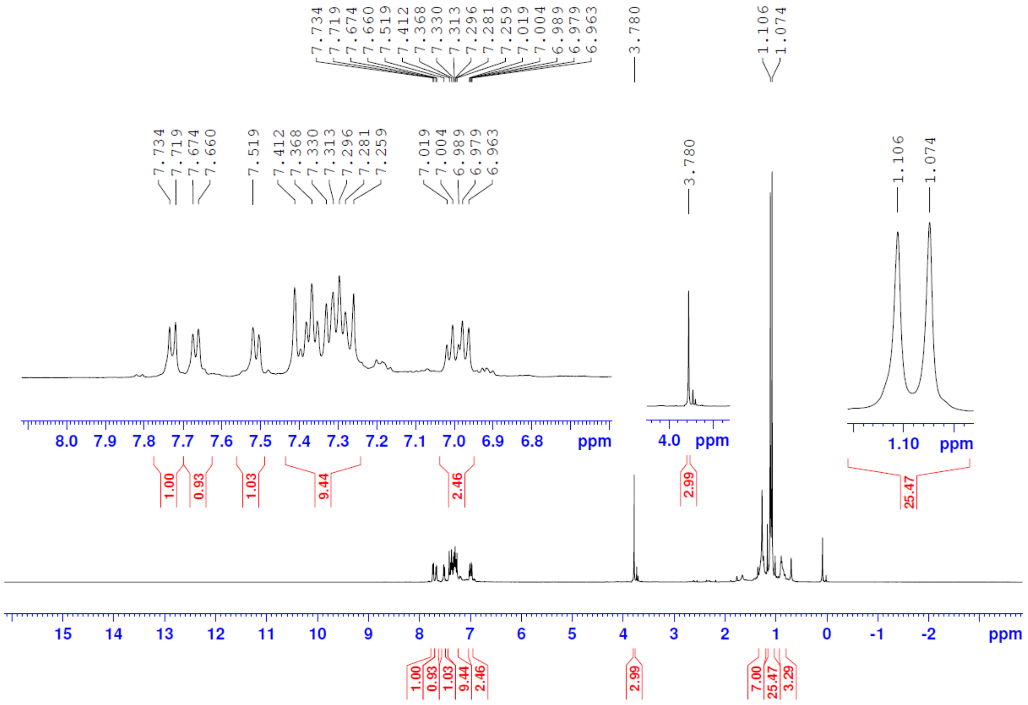

^{1}H NMR (400 MHz) spectrum of **2g** (minor regioisomer) in $CDCl_3$

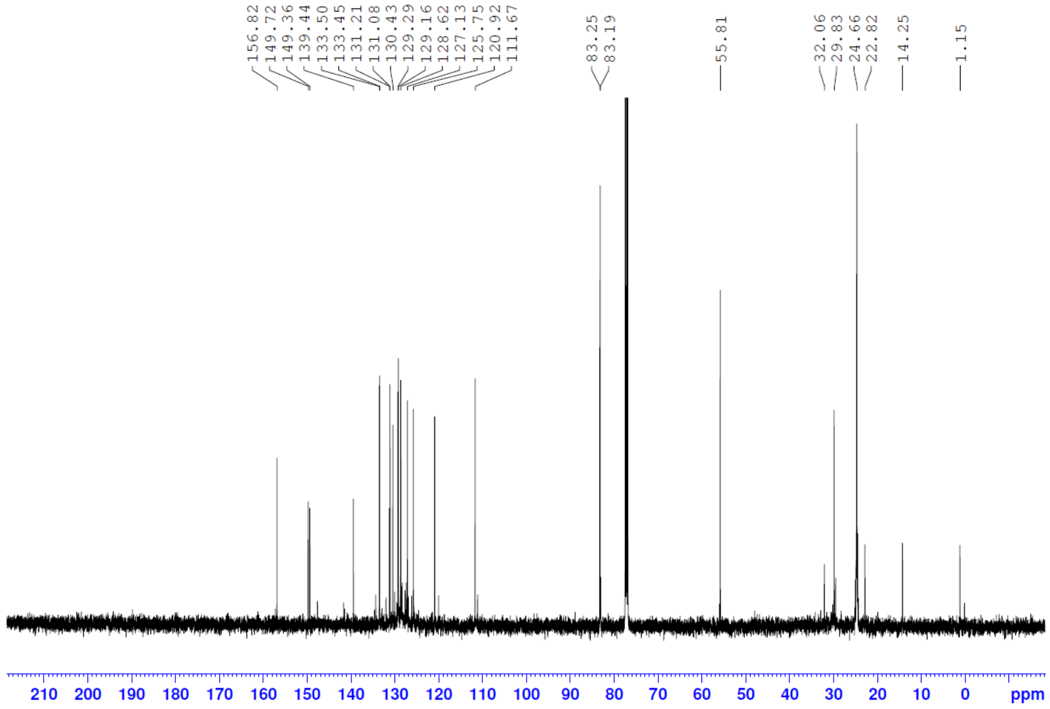

^{13}C NMR (100 MHz) spectrum of **2g** (minor regioisomer) in $CDCl_3$

NMR spectra of **2h** (major regioisomer)

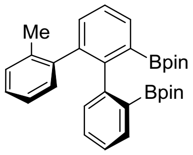

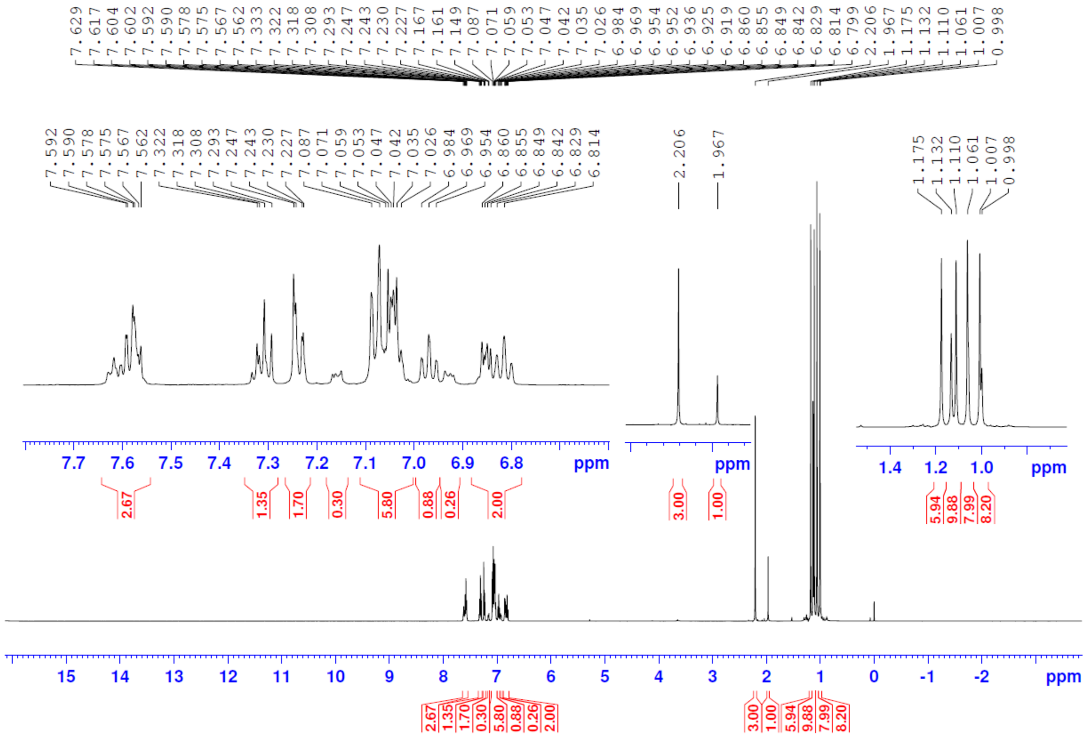

^{1}H NMR (400 MHz) spectrum of **2h** (major regioisomer) in $CDCl_3$

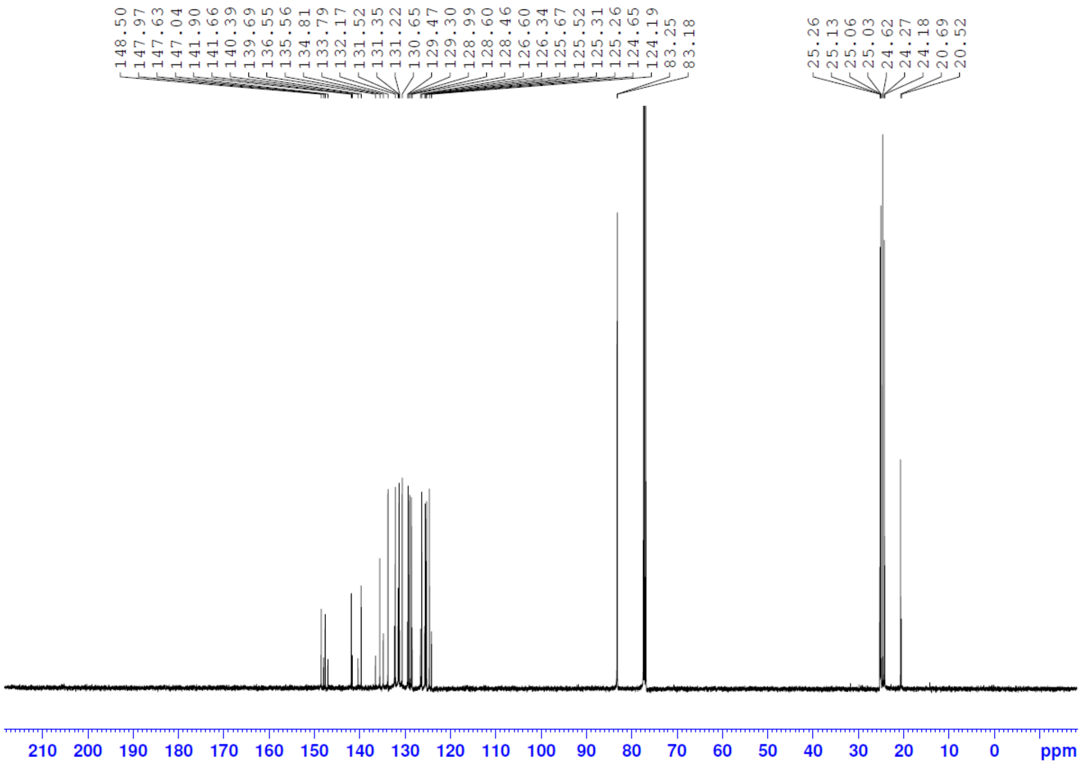

^{13}C NMR (100 MHz) spectrum of **2h** (major regioisomer) in $CDCl_3$

NMR spectra of **2h** (minor regioisomer)

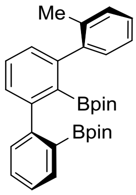

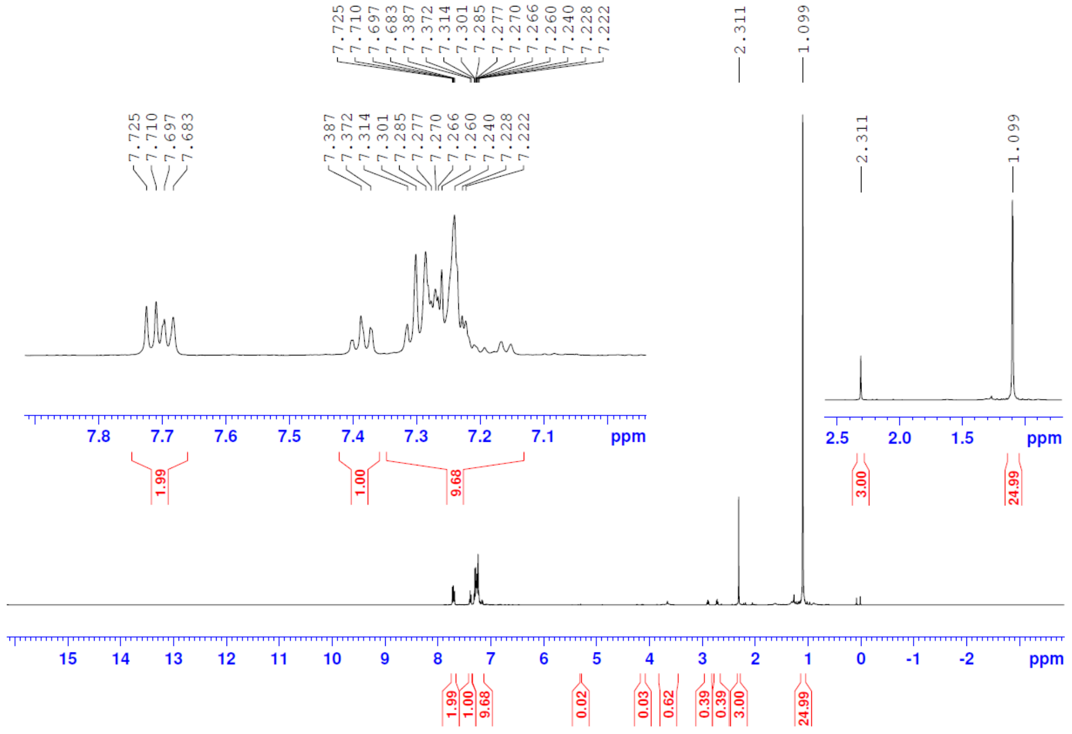

^{1}H NMR (400 MHz) spectrum of **2h** (minor regioisomer) in $CDCl_3$

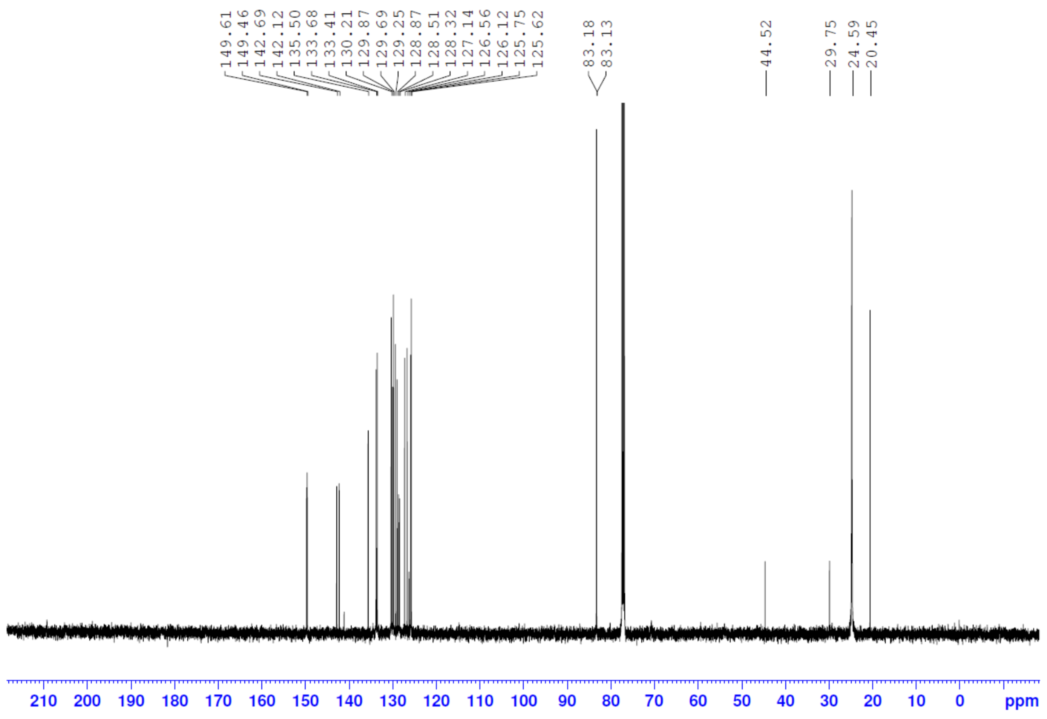

^{13}C NMR (100 MHz) spectrum of **2h** (minor regioisomer) in $CDCl_3$

NMR spectra of **2i** (both regioisomers)

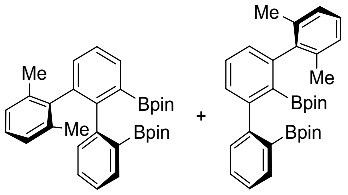

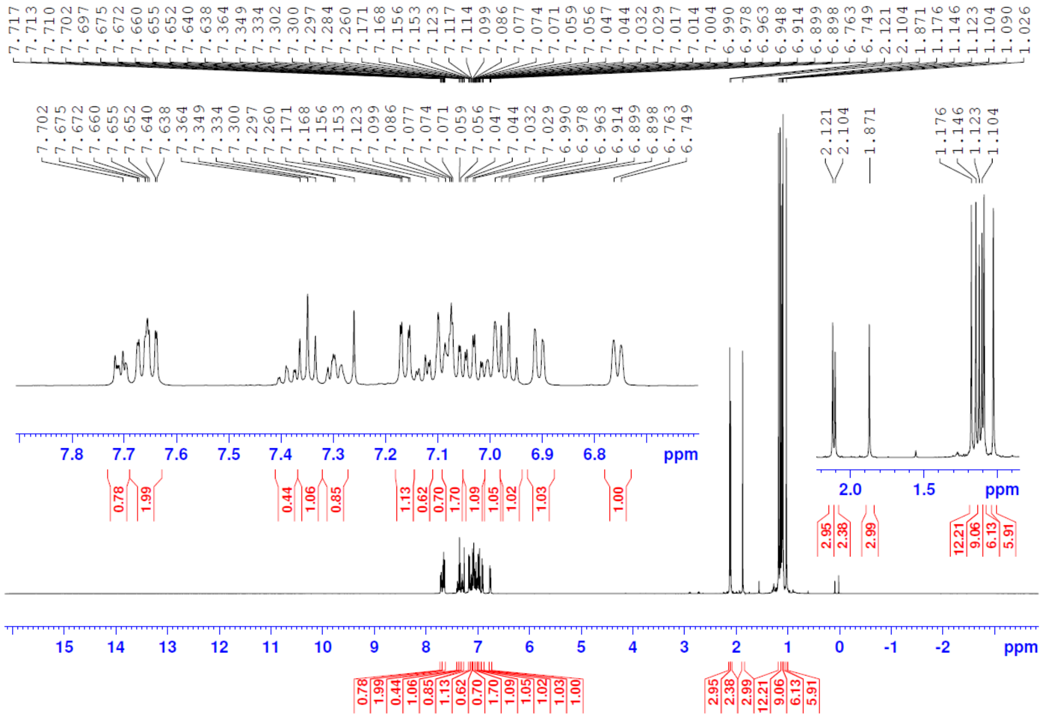

^{1}H NMR (400 MHz) spectrum of **2i** (both regioisomers) in $CDCl_3$

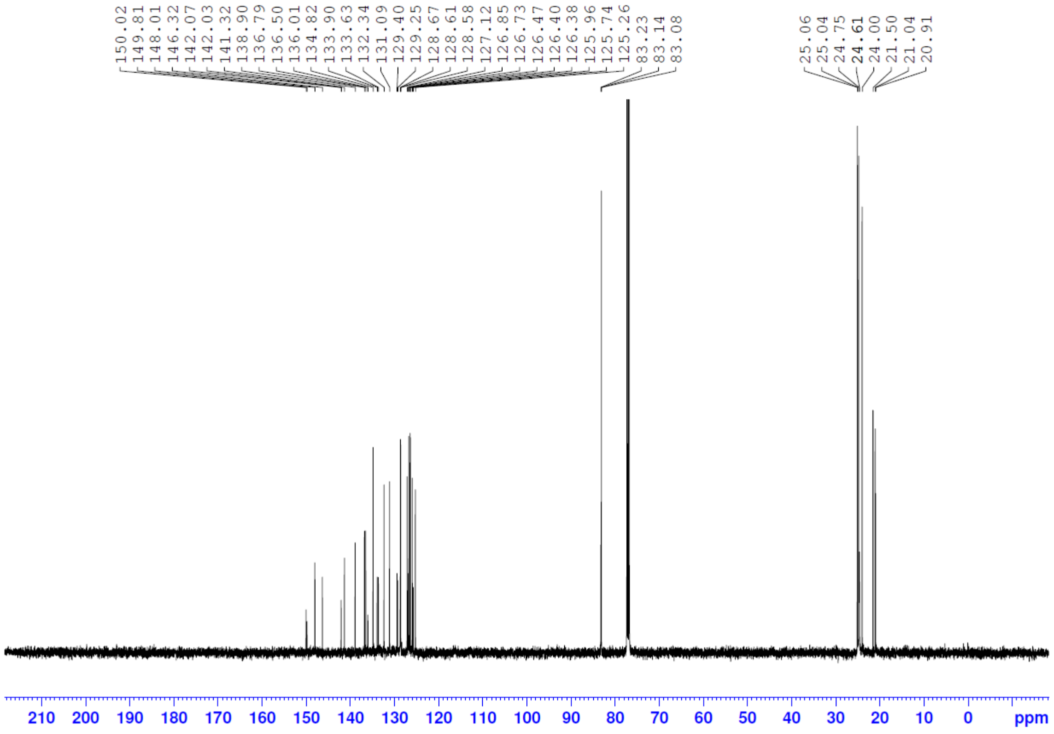

^{13}C NMR (100 MHz) spectrum of **2i** (both regioisomers) in $CDCl_3$

NMR spectra of **2j** (major regioisomer)

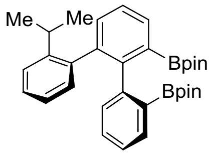

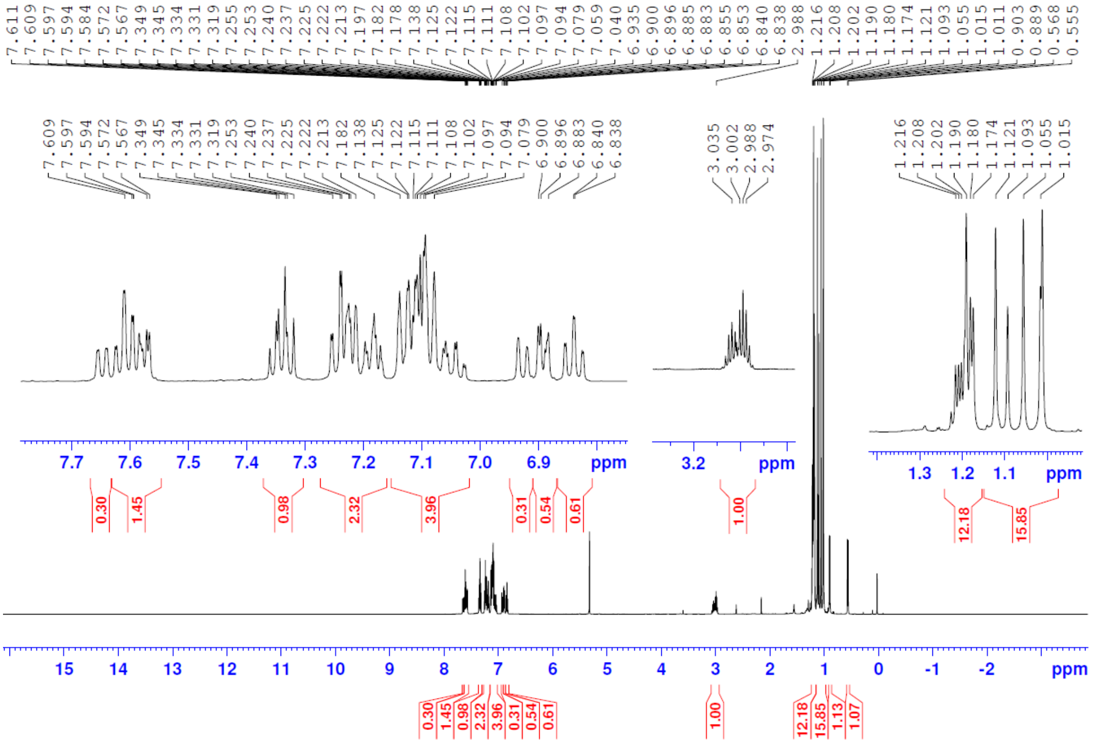

^{1}H NMR (400 MHz) spectrum of **2j** (major regioisomer) in $CDCl_3$

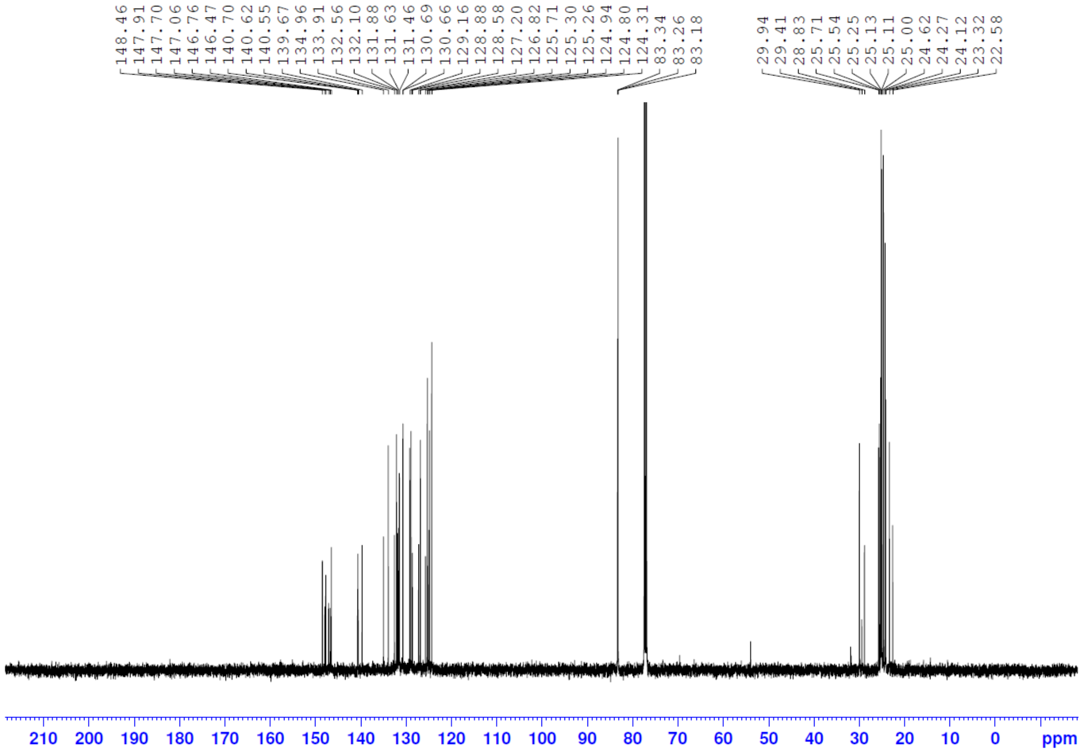

^{13}C NMR (100 MHz) spectrum of **2j** (major regioisomer) in $CDCl_3$

NMR spectra of **2k** (major regioisomer)

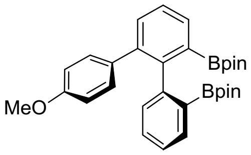

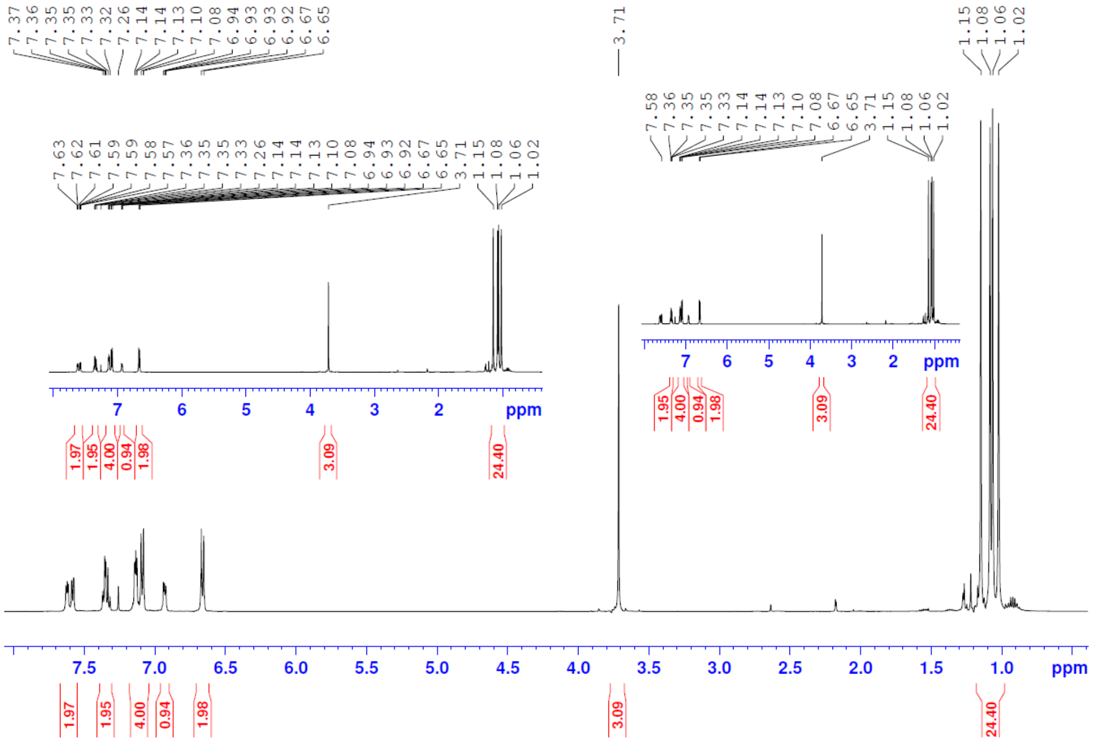

^{1}H NMR (400 MHz) spectrum of **2k** (major regioisomer) in $CDCl_3$

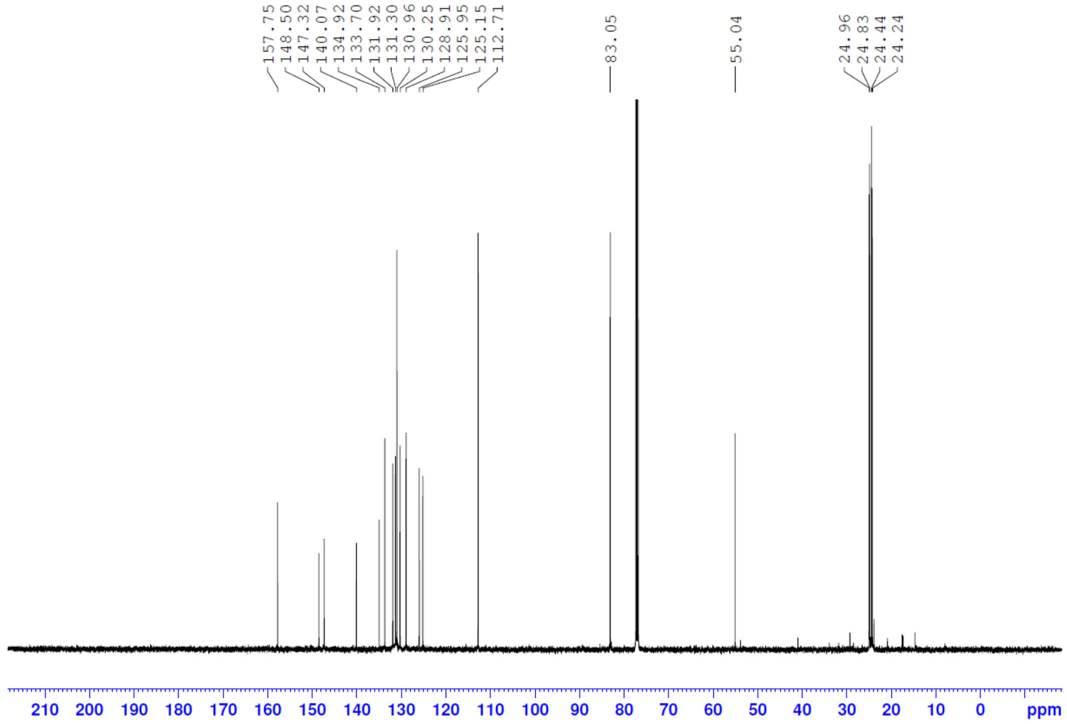

^{13}C NMR (100 MHz) spectrum of **2k** (major regioisomer) in $CDCl_3$

NMR spectra of **2l** (major regioisomer)

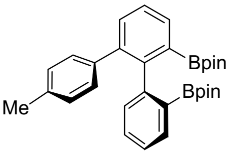

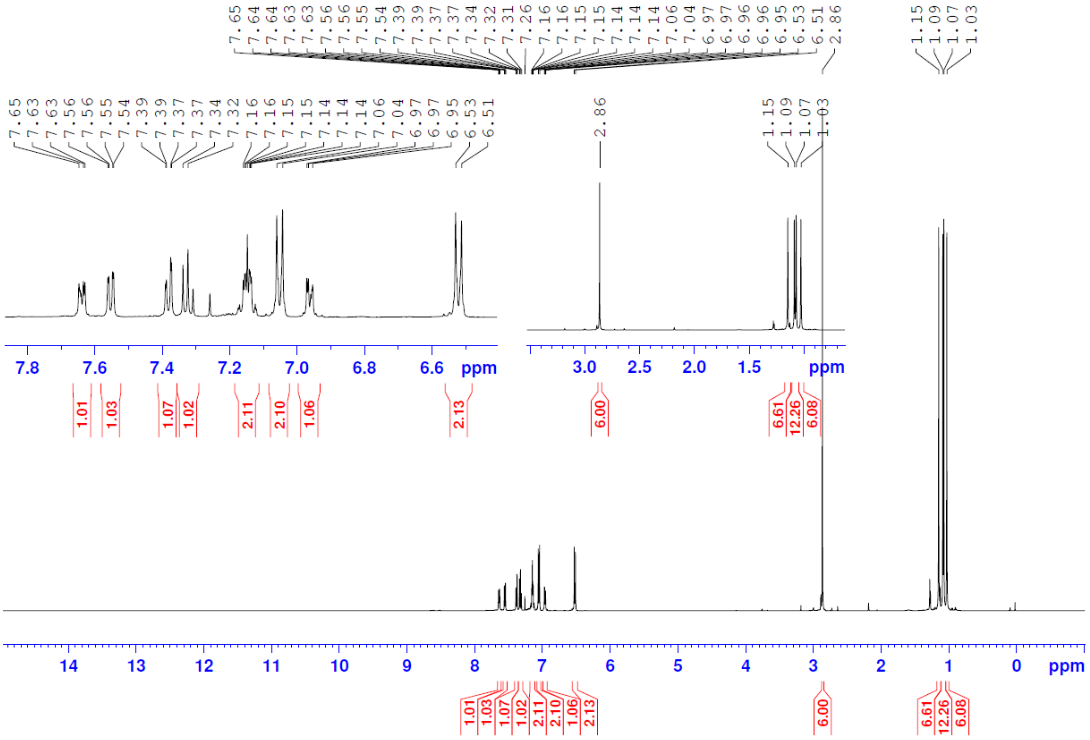

^{1}H NMR (400 MHz) spectrum of **2l** (major regioisomer) in $CDCl_3$

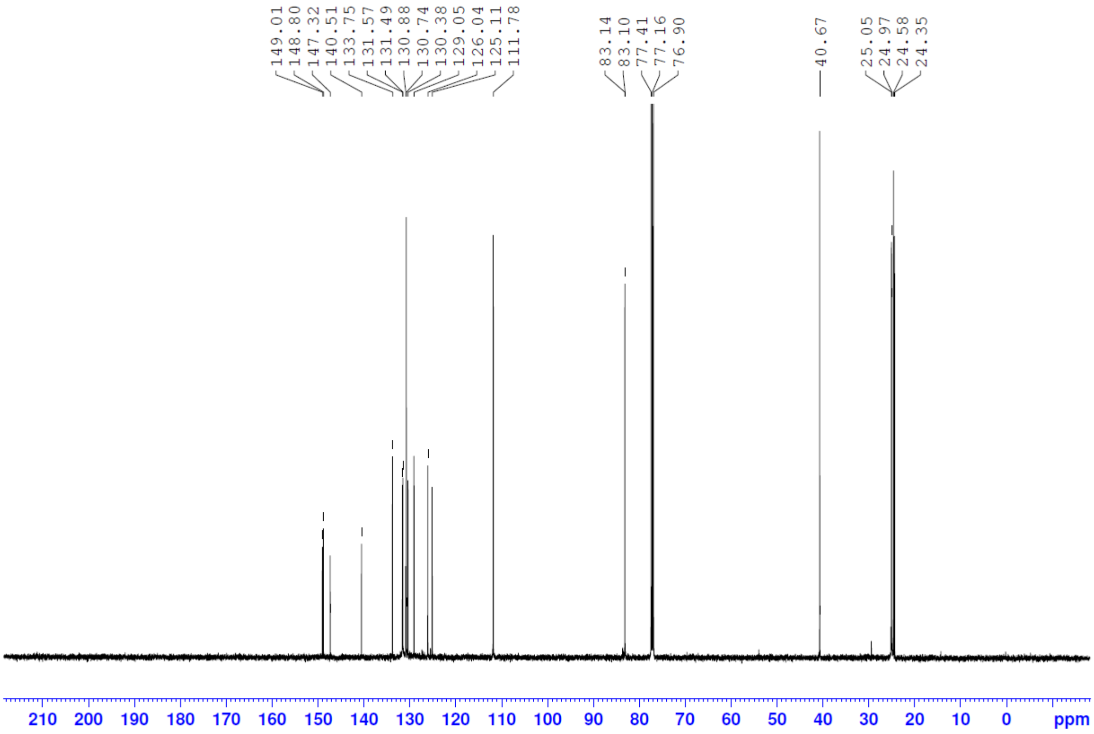

^{13}C NMR (100 MHz) spectrum of **2l** (major regioisomer) in $CDCl_3$

NMR spectra of **2m** (major regioisomer)

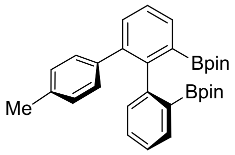

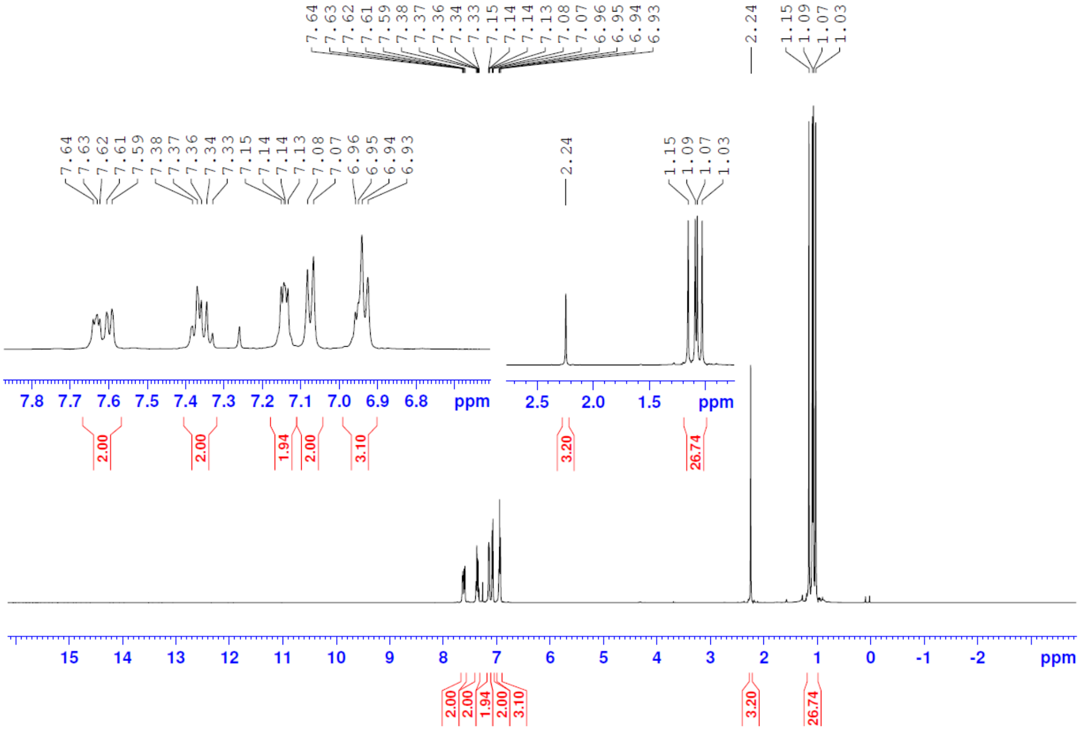

^{1}H NMR (400 MHz) spectrum of **2m** (major regioisomer) in $CDCl_3$

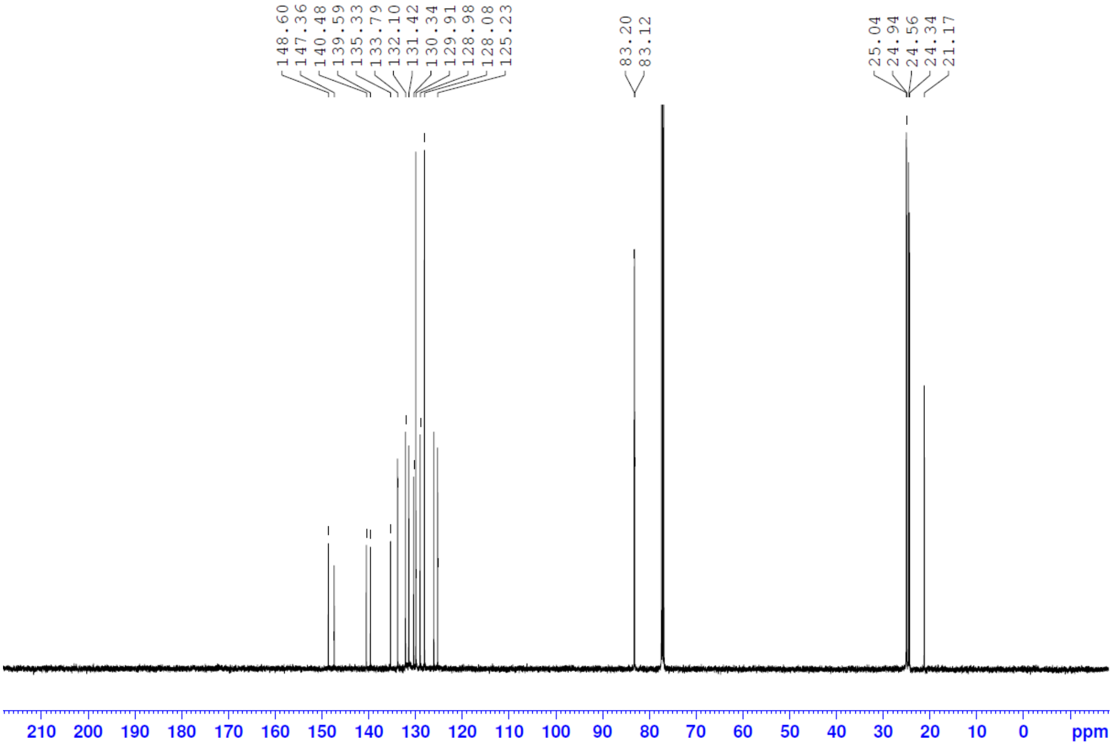

^{13}C NMR (100 MHz) spectrum of **2m** (major regioisomer) in $CDCl_3$

NMR spectra of **2m** (minor regioisomer)

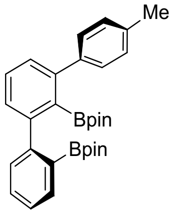

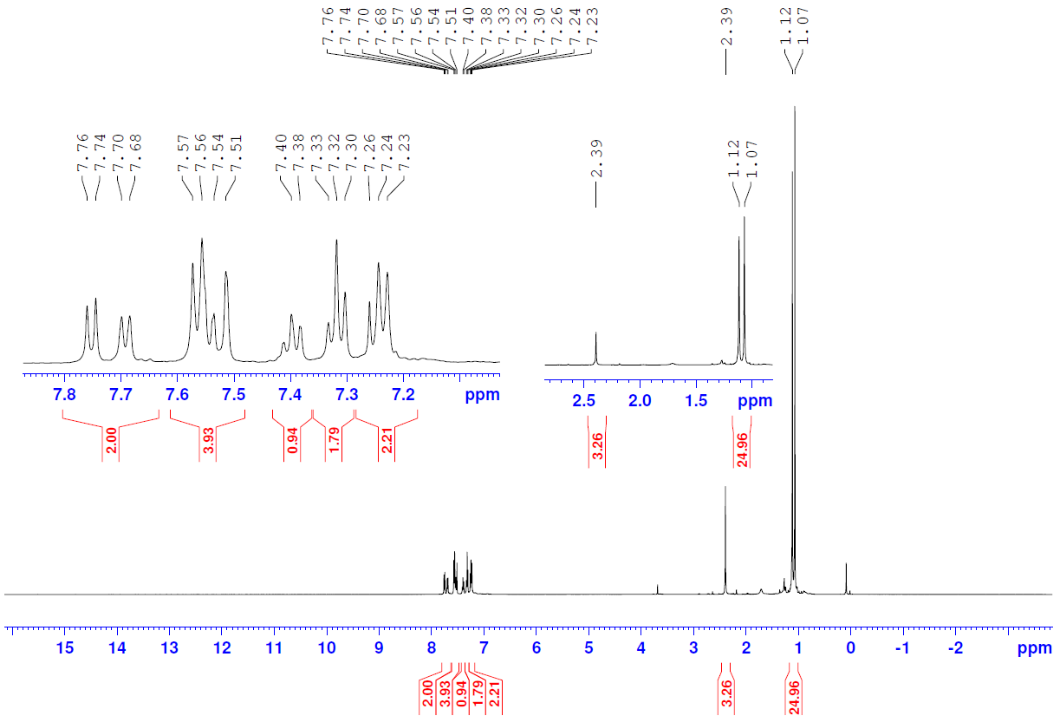

^{1}H NMR (400 MHz) spectrum of **2m** (minor regioisomer) in $CDCl_3$

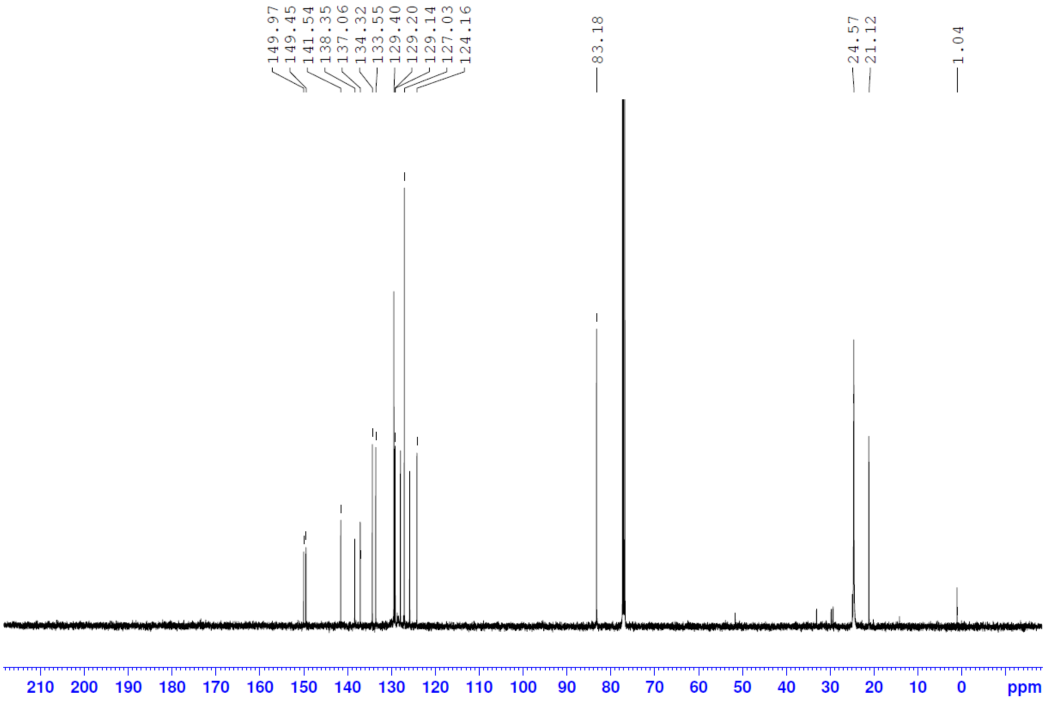

^{13}C NMR (100 MHz) spectrum of **2m** (minor regioisomer) in $CDCl_3$

NMR spectra of **2n** (major regioisomer)

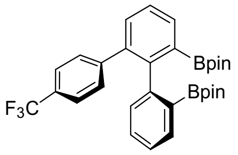

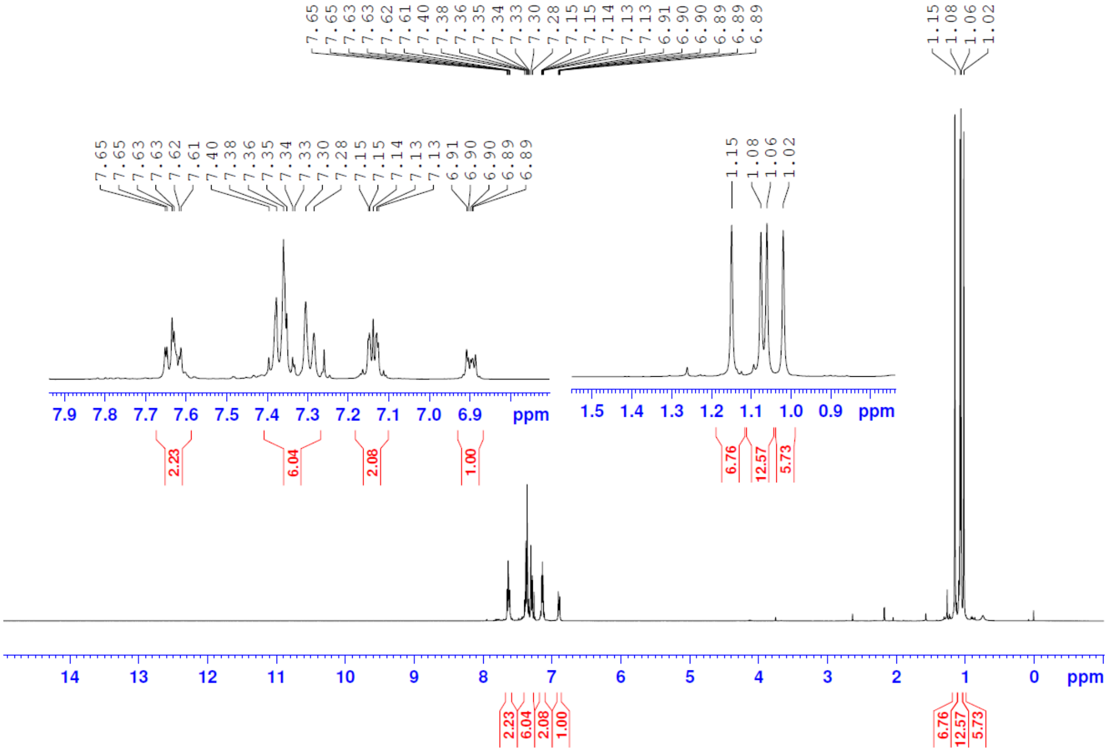

^{1}H NMR (400 MHz) spectrum of **2n** (major regioisomer) in $CDCl_3$

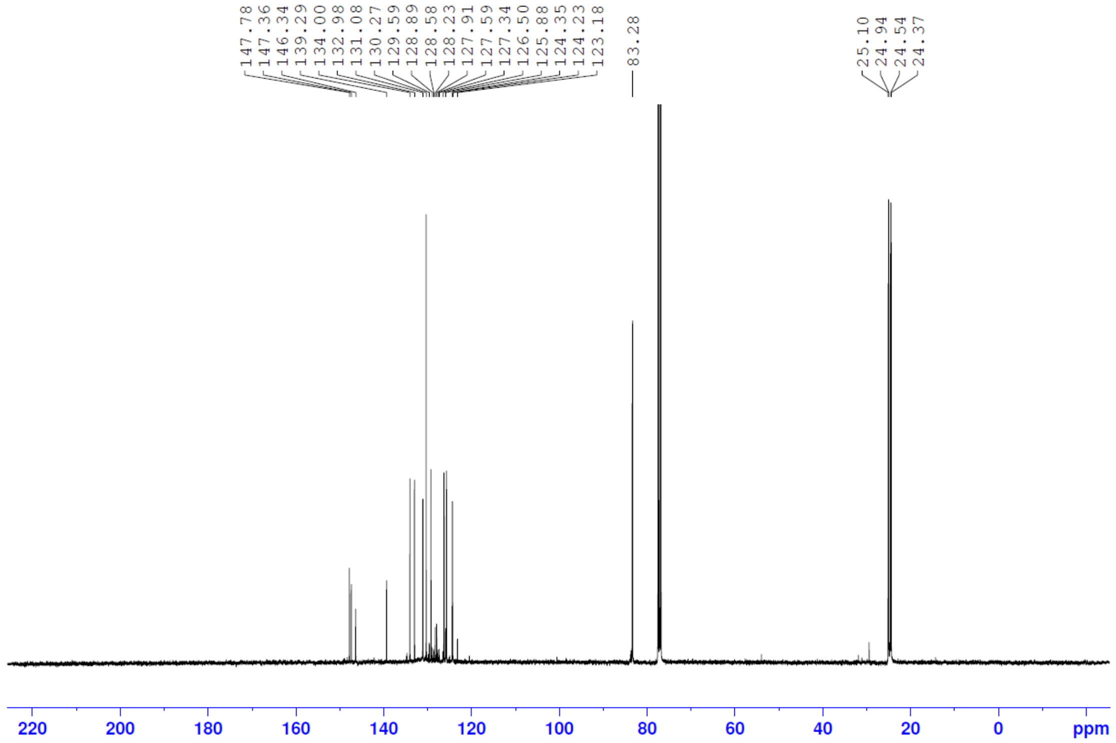

^{13}C NMR (100 MHz) spectrum of **2n** (major regioisomer) in $CDCl_3$

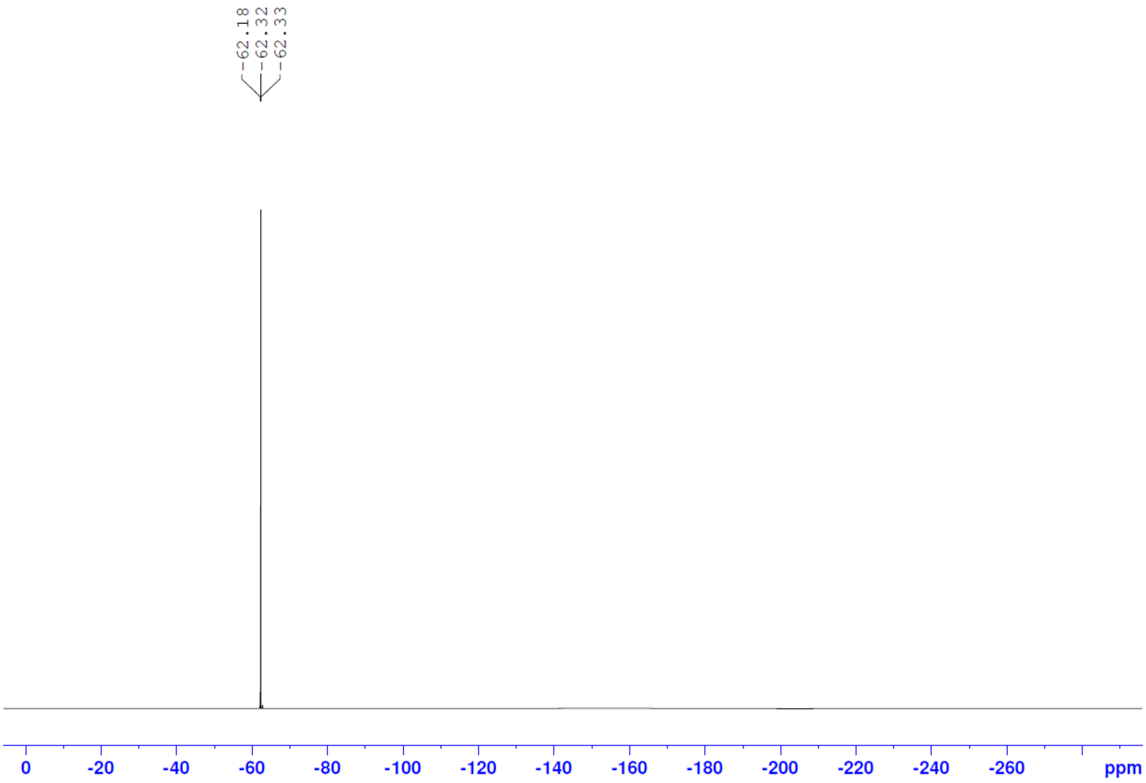

^{19}F NMR (376 MHz) spectrum of **2n** (major regioisomer) in $CDCl_3$

NMR spectra of **2n** (minor regioisomer)

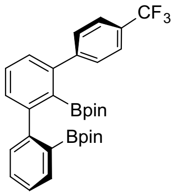

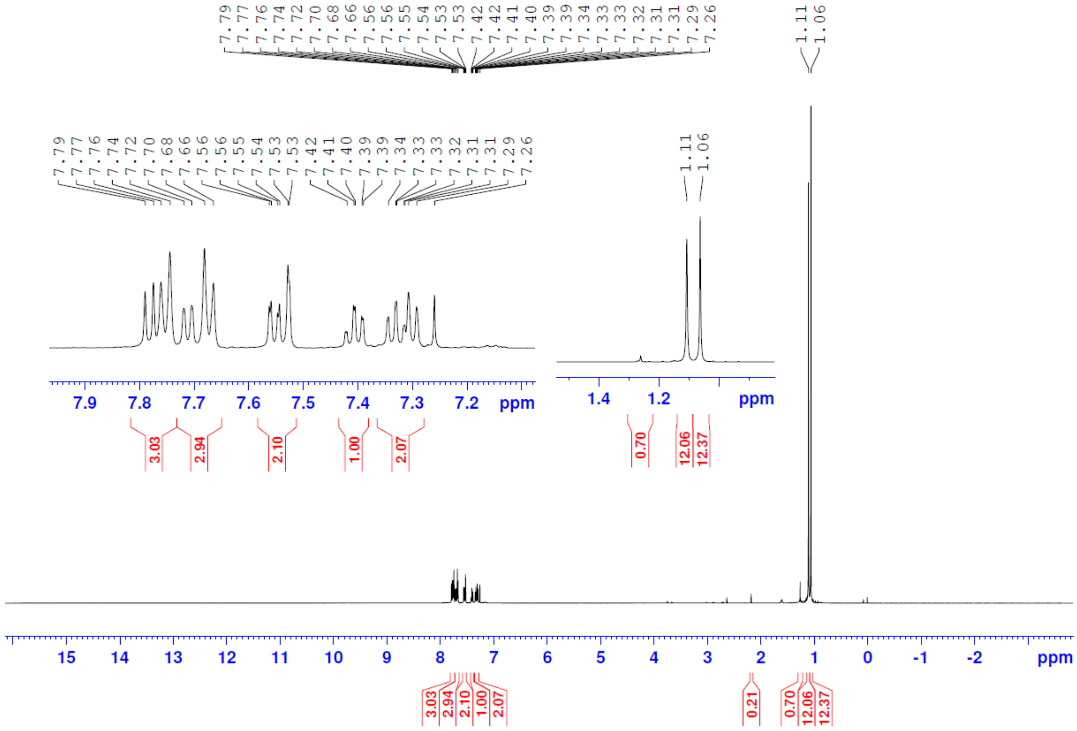

^{1}H NMR (400 MHz) spectrum of **2n** (minor regioisomer) in $CDCl_3$

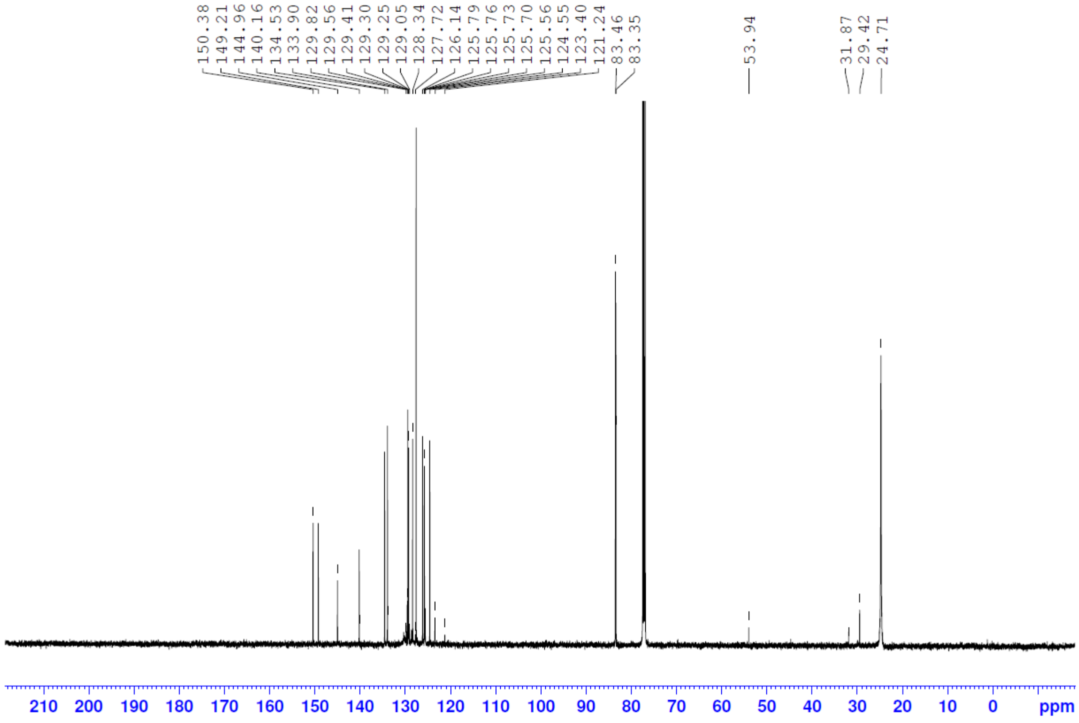

^{13}C NMR (100 MHz) spectrum of **2n** (minor regioisomer) in $CDCl_3$

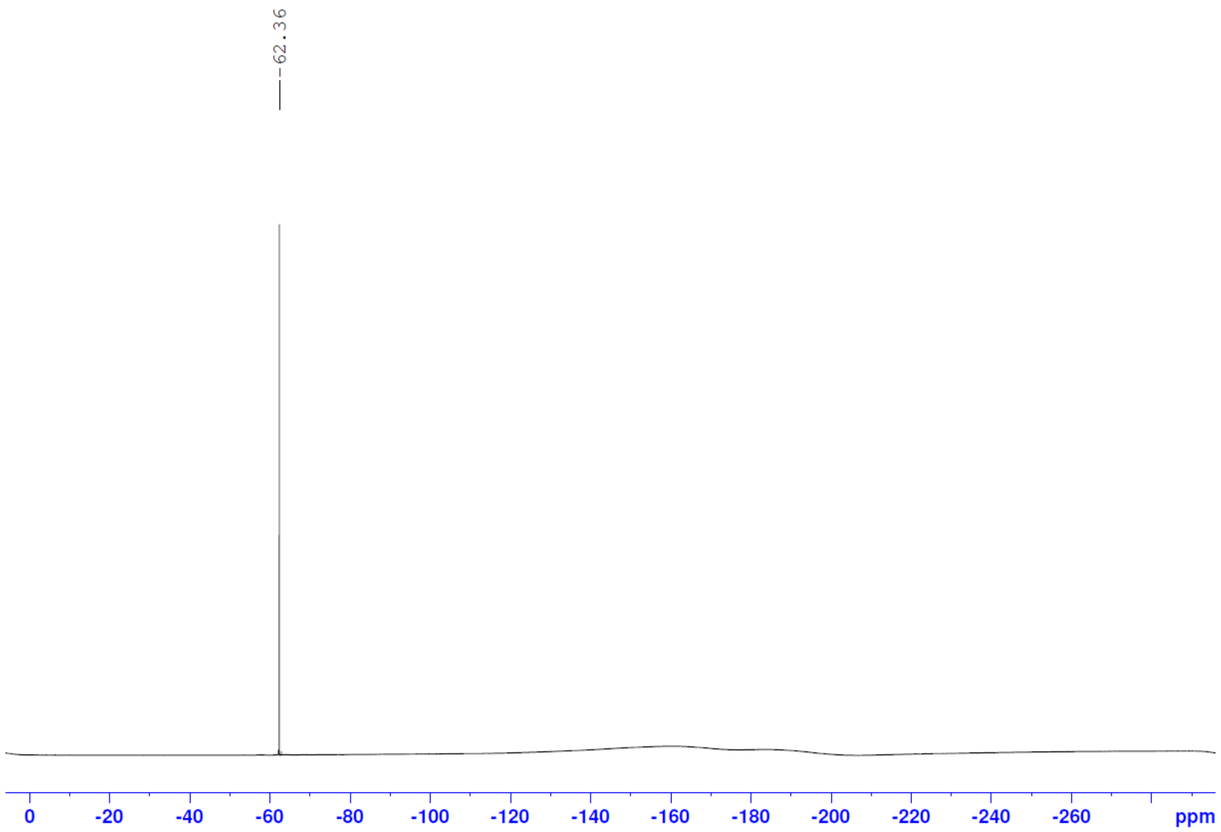

^{19}F NMR (376 MHz) spectrum of **2n** (minor regioisomer) in $CDCl_3$

NMR spectra of **2o** (major regioisomer)

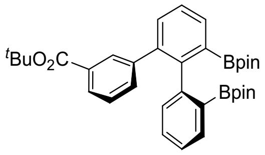

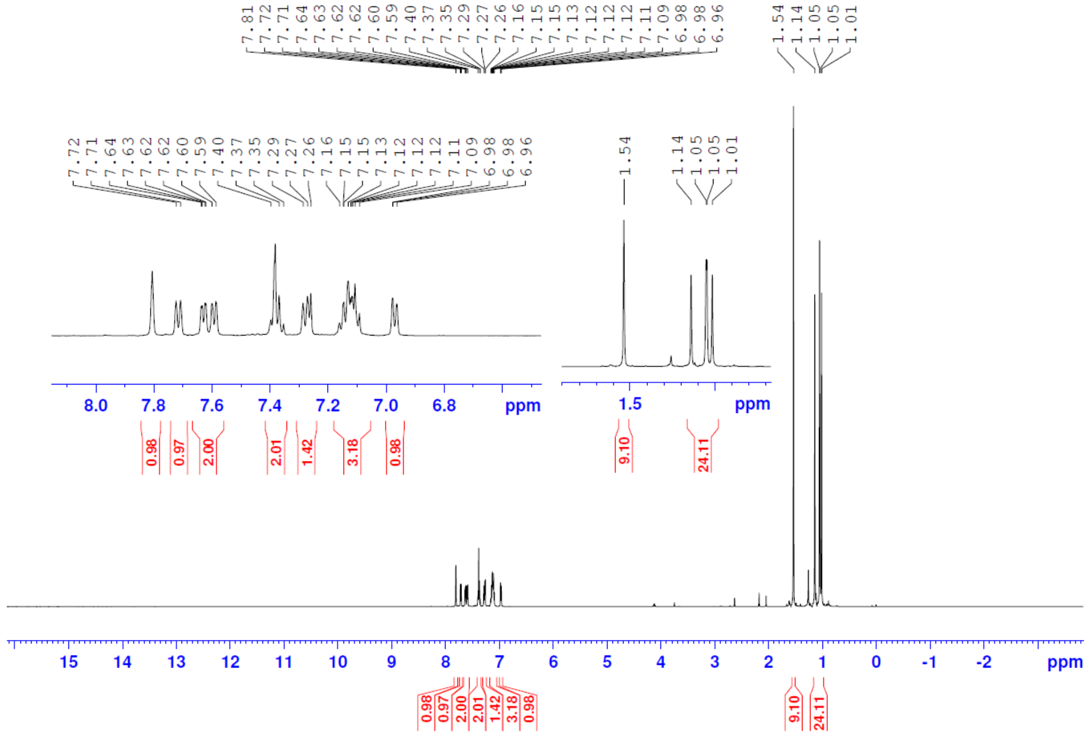

^{1}H NMR (400 MHz) spectrum of **2o** (major regioisomer) in $CDCl_3$

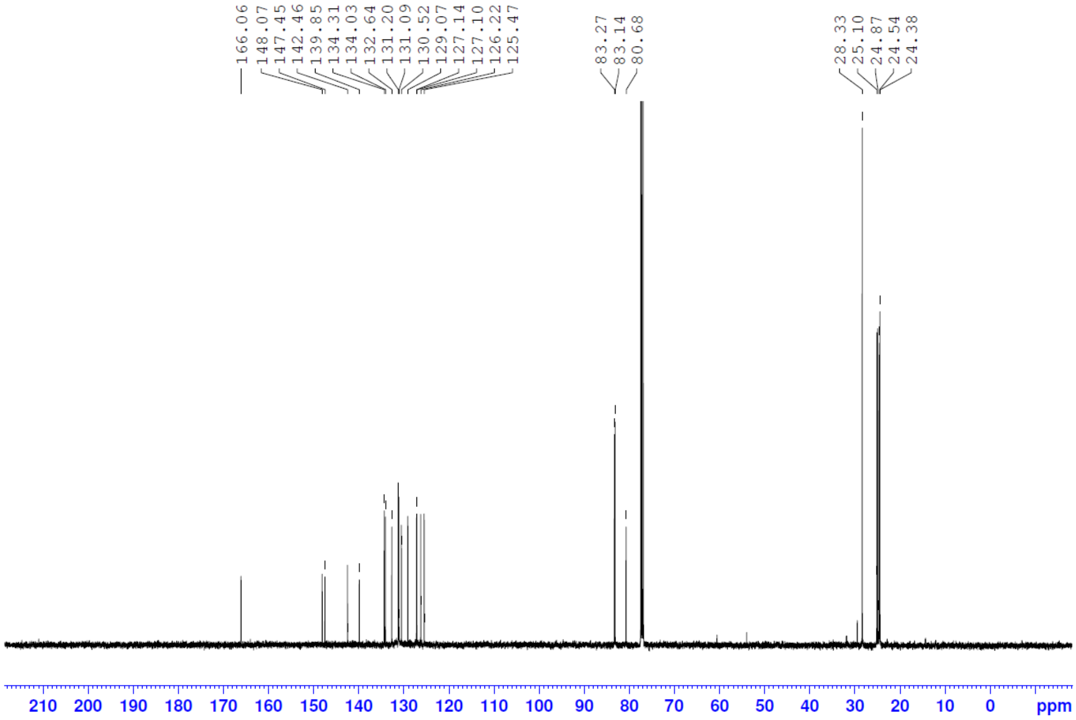

^{13}C NMR (100 MHz) spectrum of **2o** (major regioisomer) in $CDCl_3$

NMR spectra of **2o** (minor regioisomer)

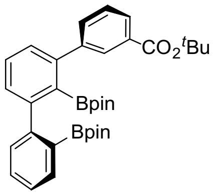

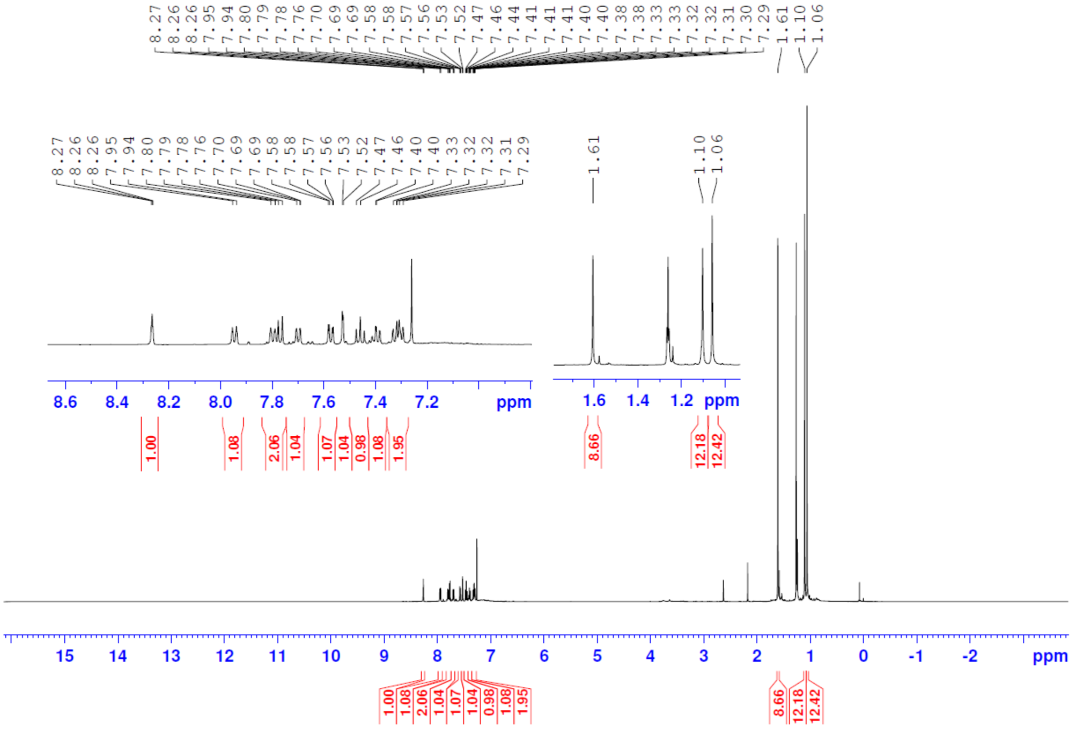

^{1}H NMR (400 MHz) spectrum of **2o** (minor regioisomer) in $CDCl_3$

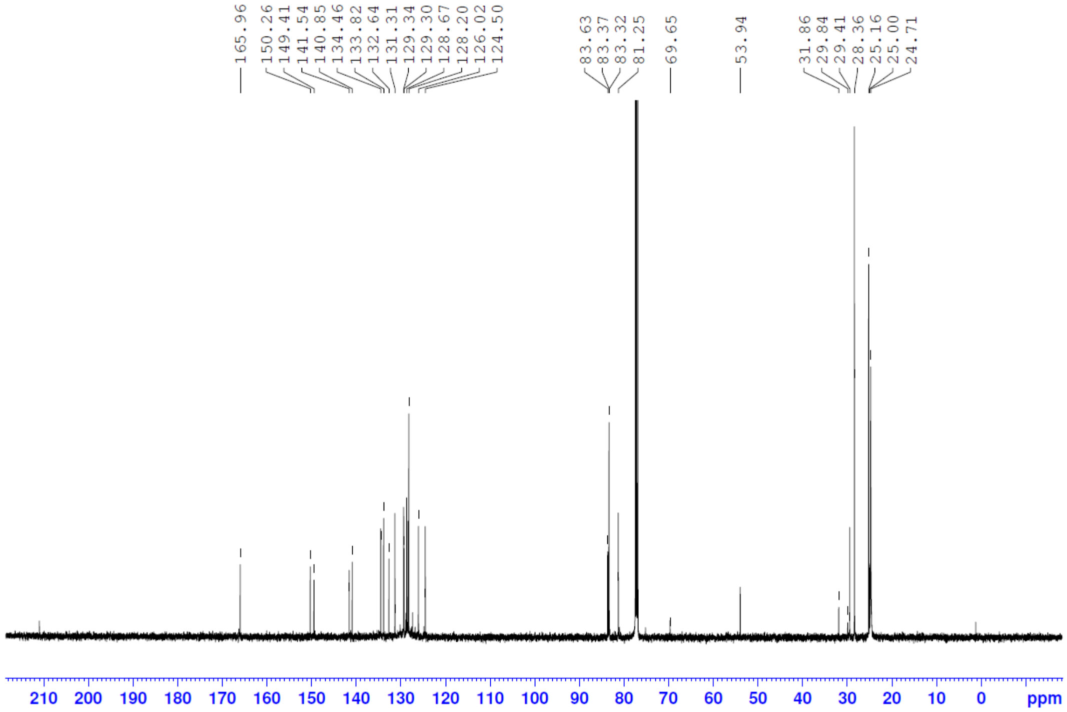

^{13}C NMR (100 MHz) spectrum of **2o** (minor regioisomer) in $CDCl_3$

NMR spectra of **2p** (major regioisomer)

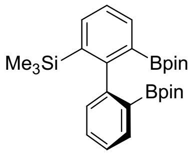

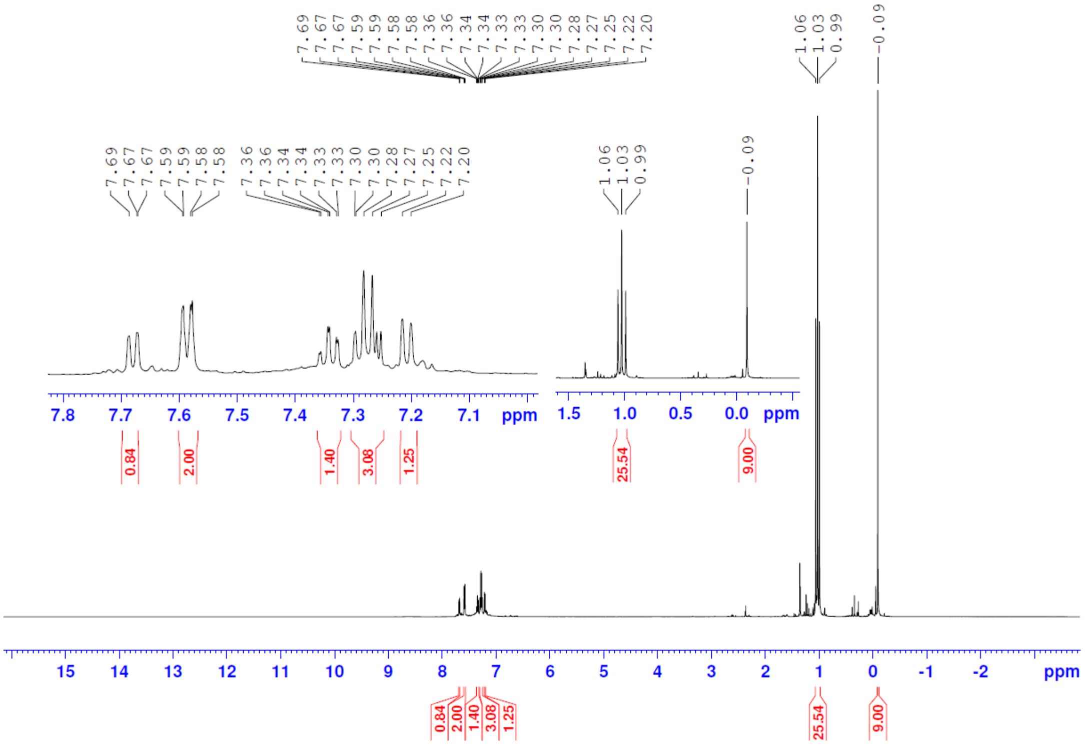

^{1}H NMR (400 MHz) spectrum of **2p** (major regioisomer) in $CDCl_3$

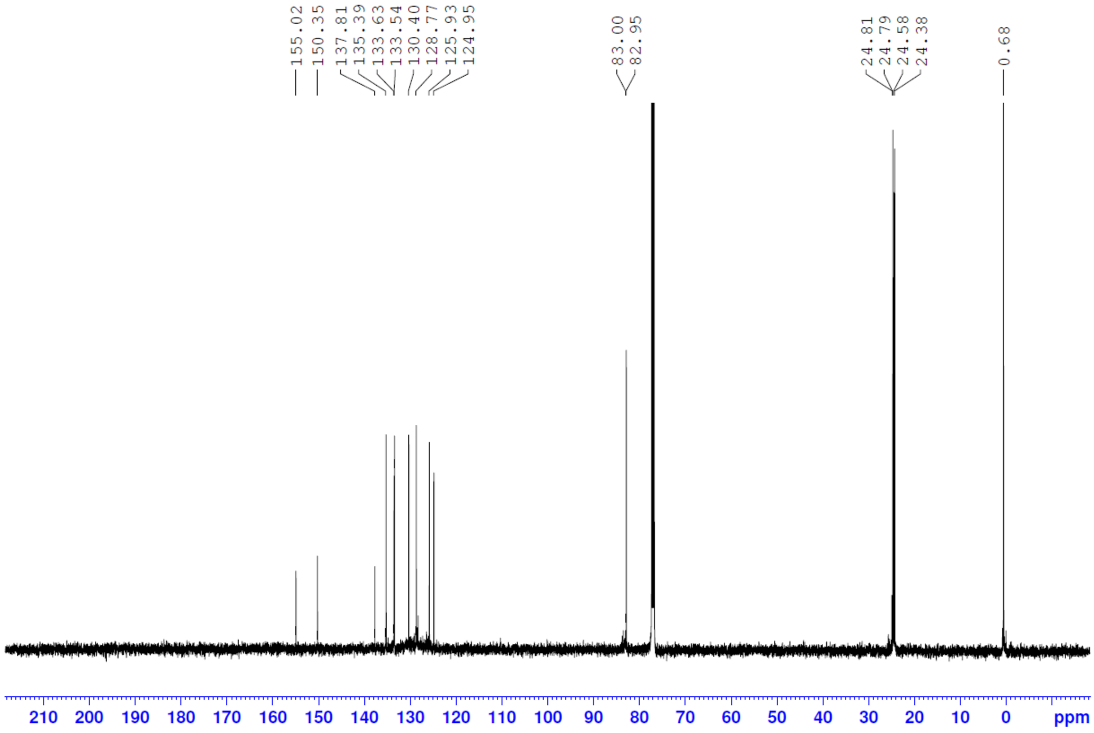

^{13}C NMR (100 MHz) spectrum of **2p** (major regioisomer) in $CDCl_3$

NMR spectra of **2q** (major regioisomer)

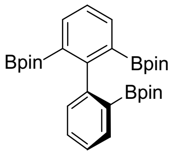

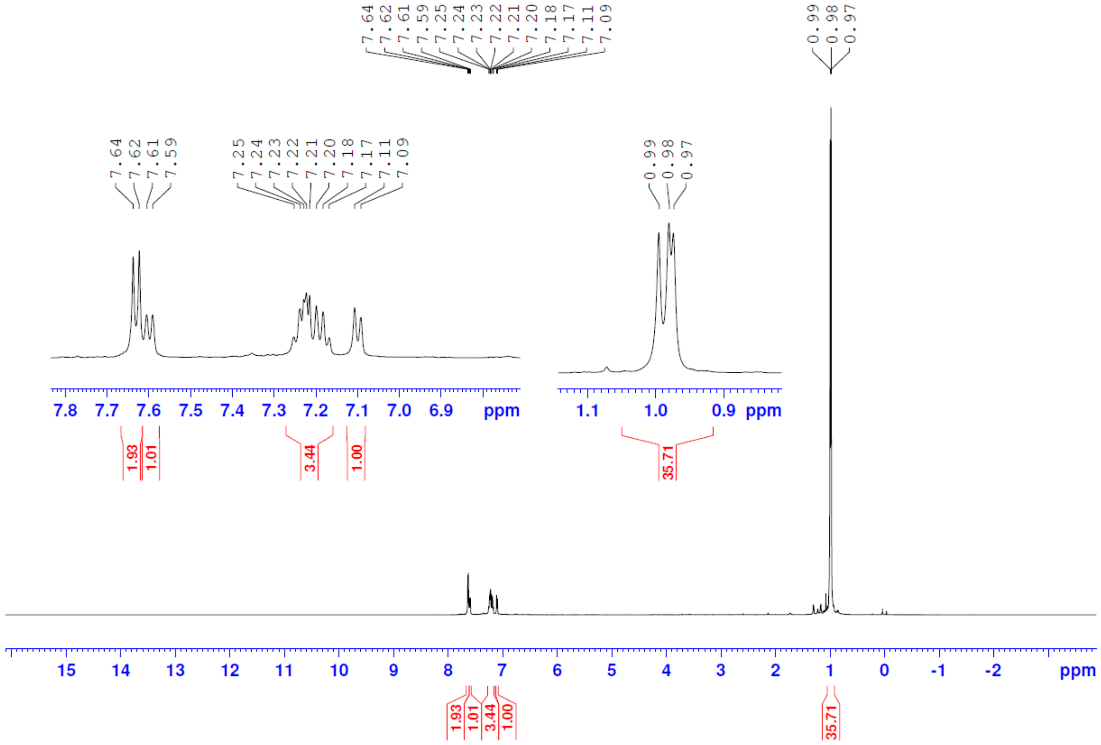

^{1}H NMR (400 MHz) spectrum of **2q** (major regioisomer) in $CDCl_3$

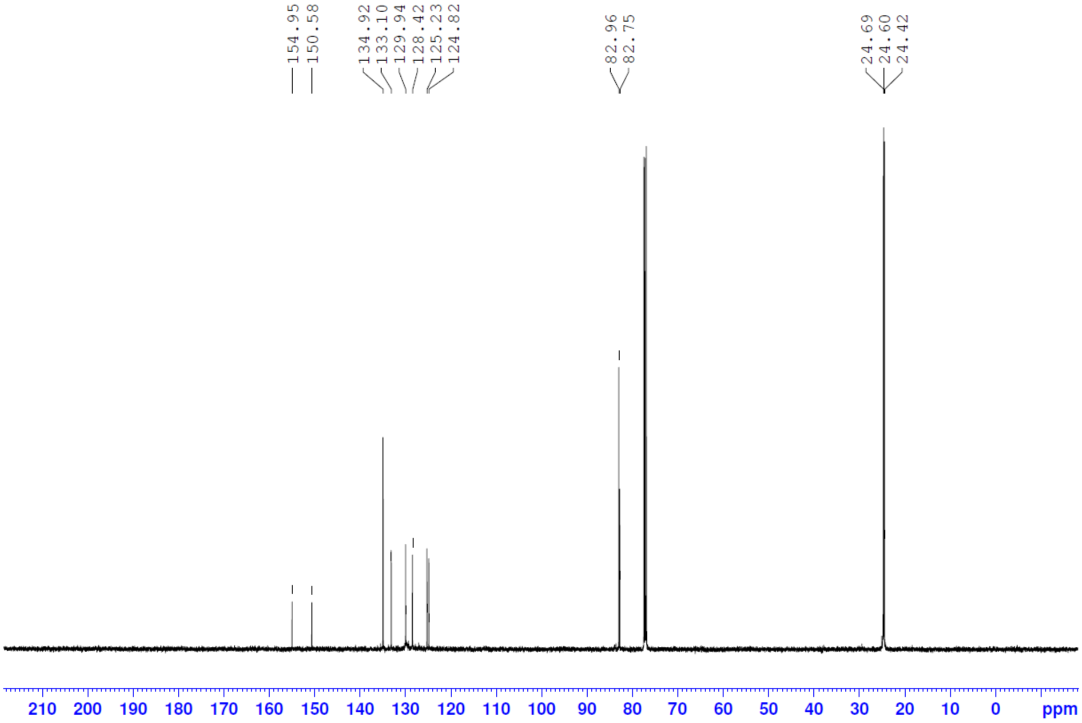

^{13}C NMR (100 MHz) spectrum of **2q** (major regioisomer) in $CDCl_3$

NMR spectra of **2q** (major regioisomer)

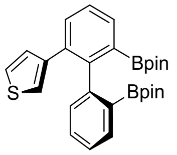

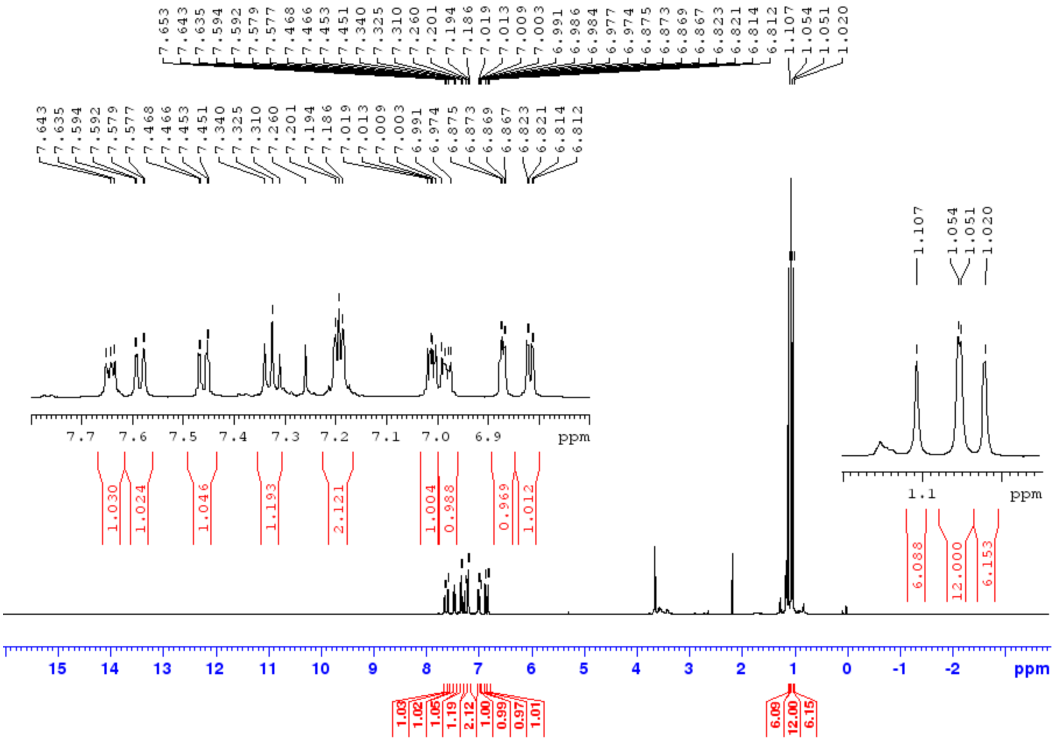

^{1}H NMR (400 MHz) spectrum of **2r** (major regioisomer) in $CDCl_3$

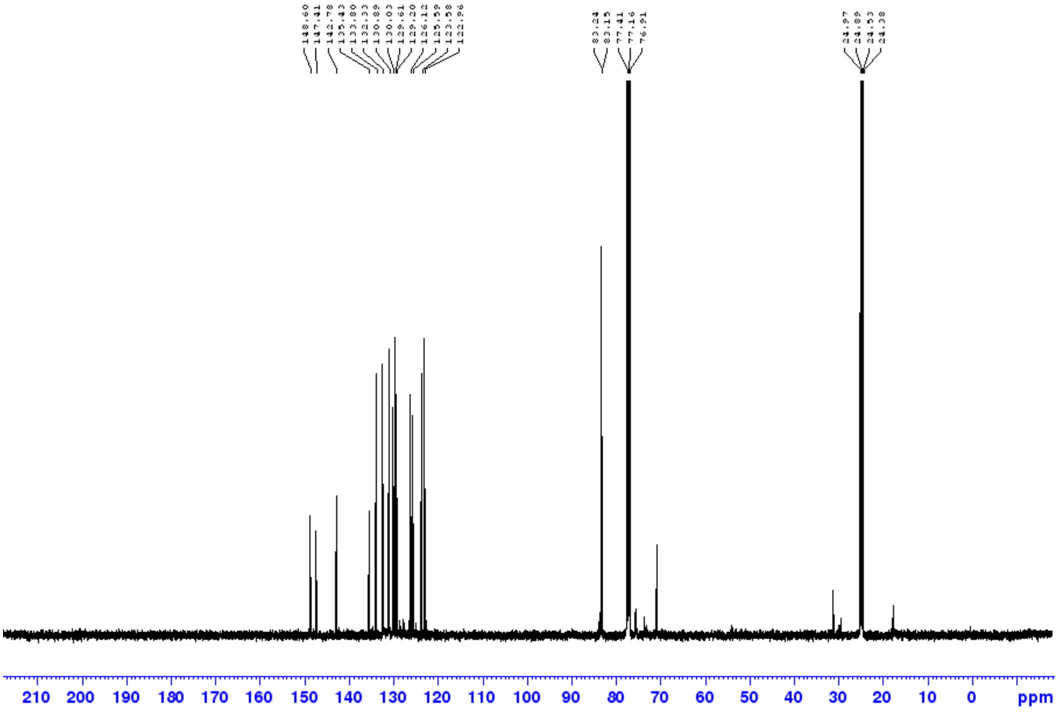

^{13}C NMR (100 MHz) spectrum of **2r** (major regioisomer) in $CDCl_3$

NMR spectra of **7**

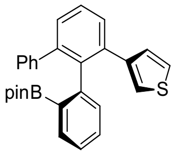

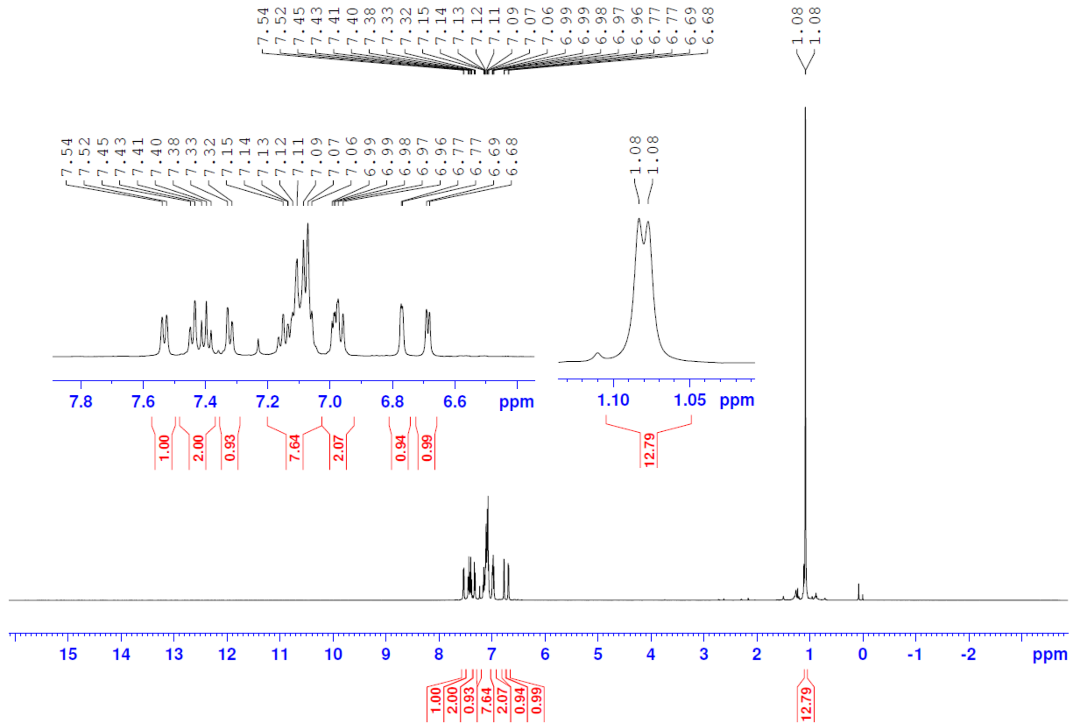

^{1}H NMR (400 MHz) spectrum of **7** in $CDCl_3$

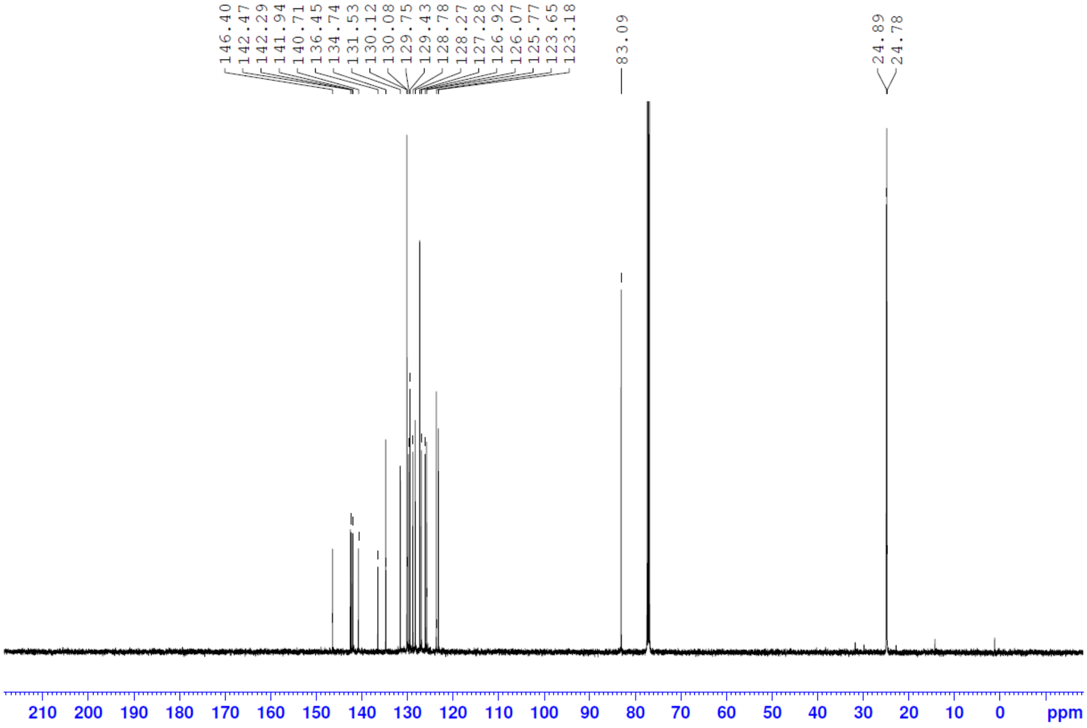

^{13}C NMR (100 MHz) spectrum of **7** in $CDCl_3$

NMR spectra of **7’**

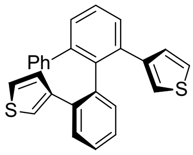

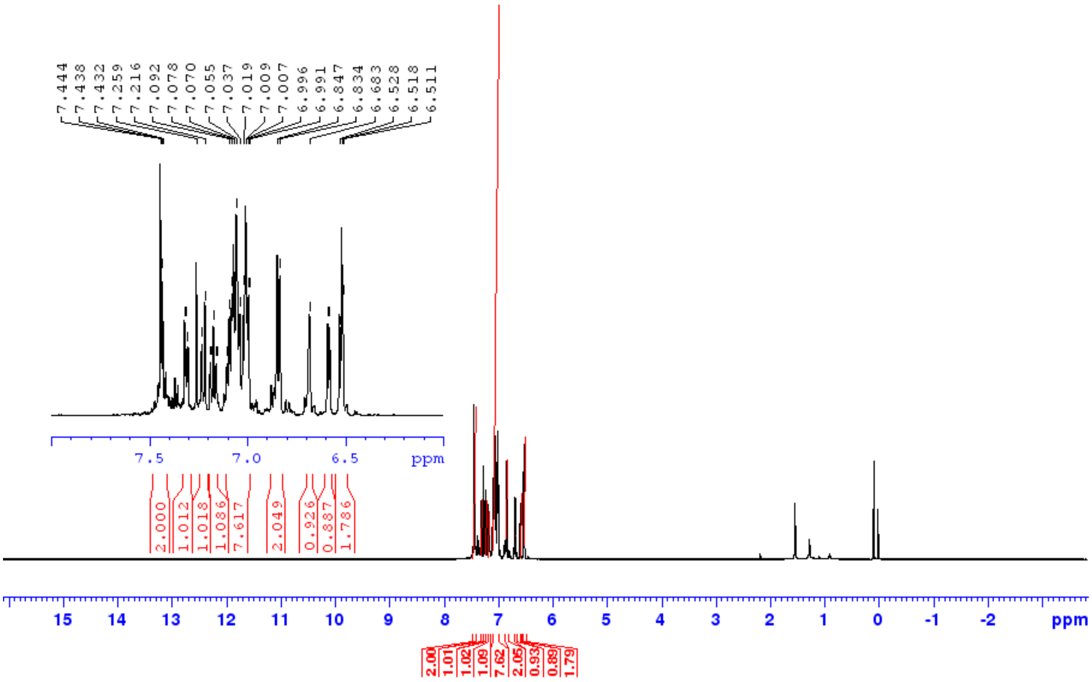

^{1}H NMR (400 MHz) spectrum of **7’** in $CDCl_3$

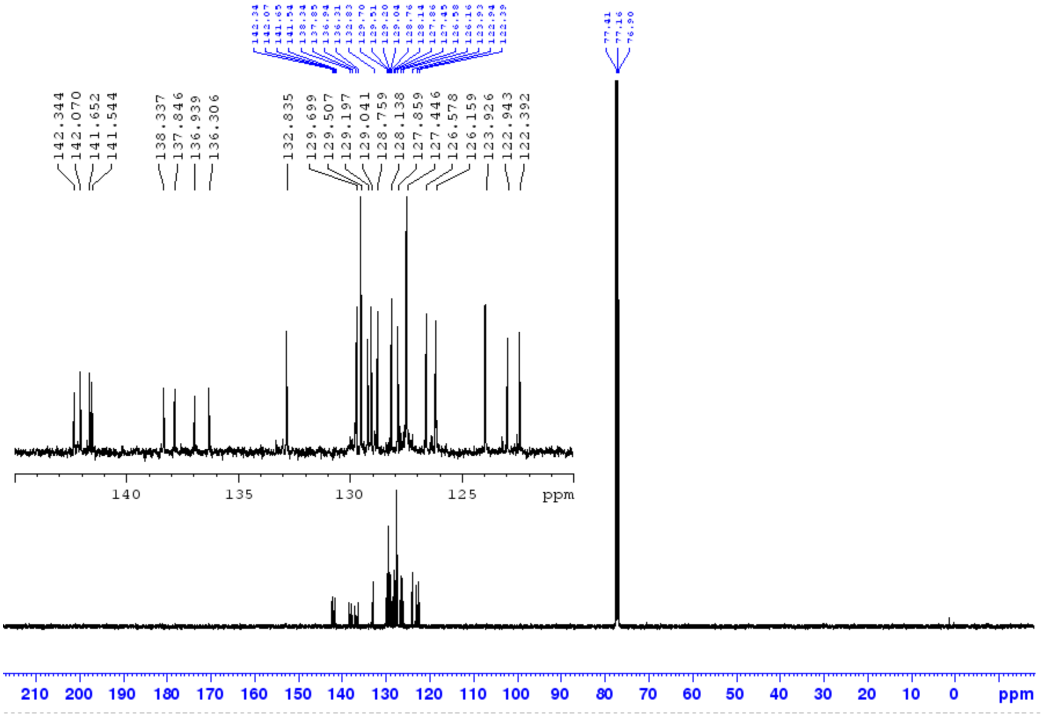

^{13}C NMR (100 MHz) spectrum of **7’** in $CDCl_3$

NMR spectra of **8**

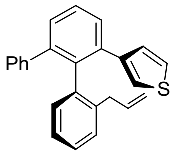

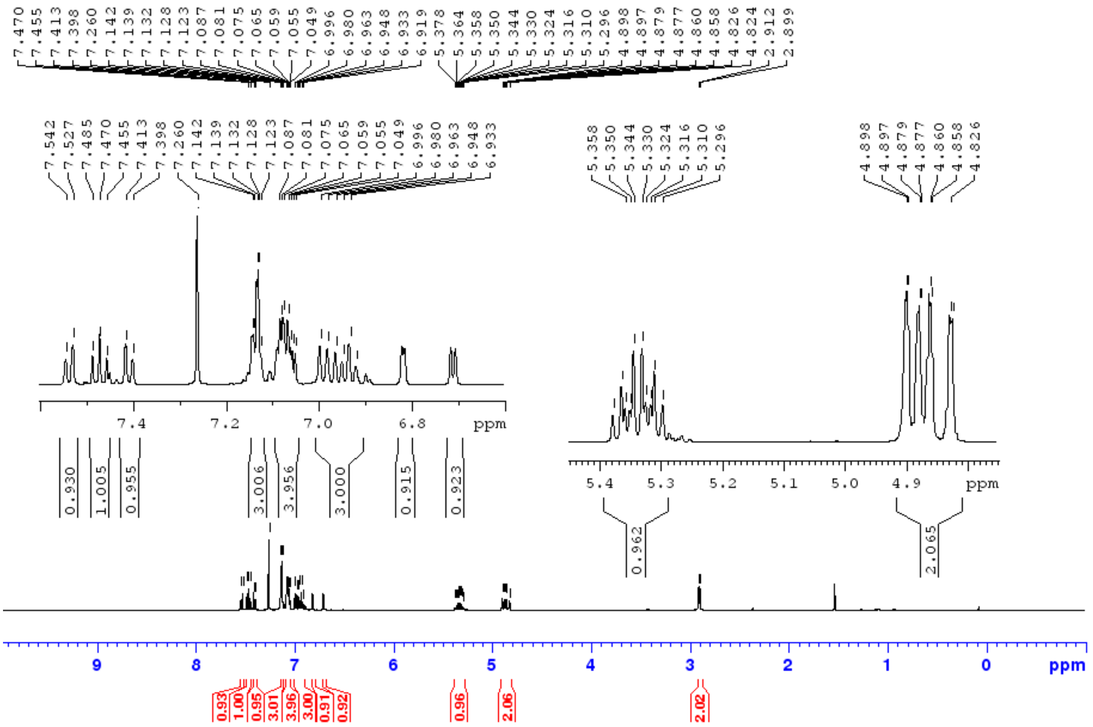

^{1}H NMR (400 MHz) spectrum of **8** in $CDCl_3$

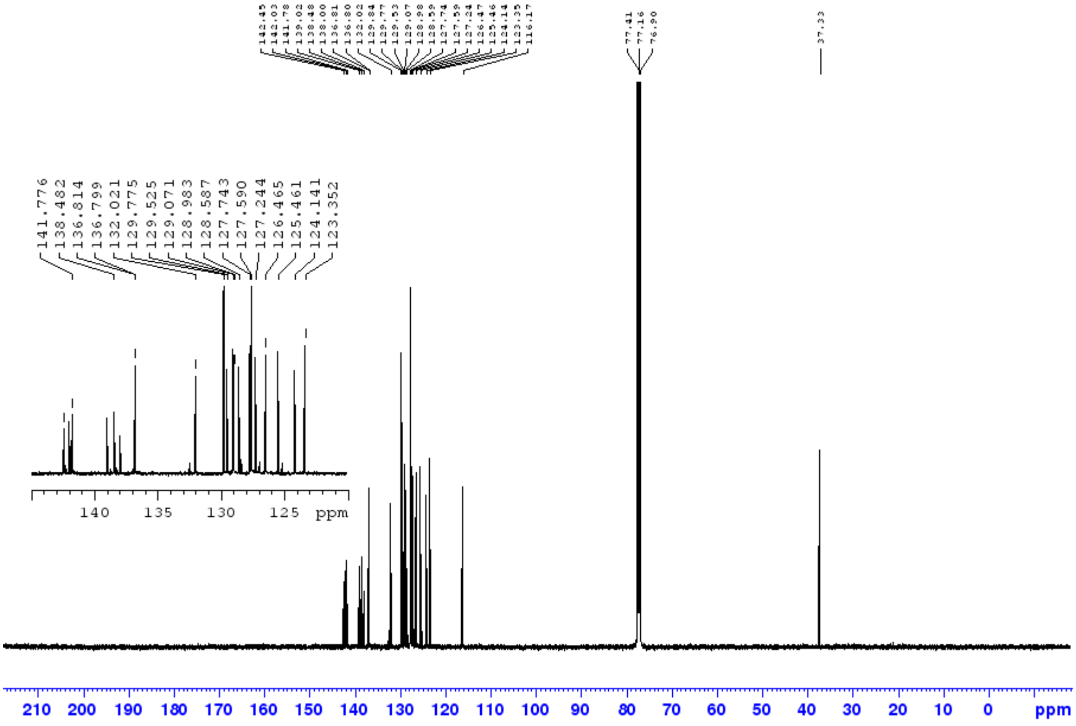

^{13}C NMR (100 MHz) spectrum of **8** in $CDCl_3$

9. References

[103] S. L. Wang, M. L. Pan, W. S. Su and Y. T. Wu, *Angew. Chem. Int. Ed. Engl.* **2017**, *56*, 14694-14697.

[104] F. R. B. Leroux, L.; Heiss, C.; Colobert, F.; Lanfranchi, D. A., *Adv. Synth. Catal.* **2007**, *349*, 2705-2713.

[105] O. V. Dolomanov, L. J. Bourhis, R. J. Gildea, J. A. K. Howard and H. Puschmann, *J.Appl. Cryst.* **2009**, *42*, 339-341.

[106] G. M. Sheldrick, *Acta Cryst. A* **2015**, *71*, 3-8.

[107] A. L. B. Polishchuk, K. L.; Friedman, L. A.; Jones, M. Jr, *J. Phys. Org. Chem.* **2004**, *13*, 798-806.

[108] J. M. Breunig, P. Gupta, A. Das, S. Tussupbayev, M. Diefenbach, M. Bolte, M. Wagner, M. C. Holthausen and H. W. Lerner, *Chem. Asian J.* **2014**, *9*, 3163-3173.

[109] H. Takano, K. S. Kanyiva and T. Shibata, *Org. Lett.* **2016**, *18*, 1860-1863.

[110] Y. Koga, M. Kamo, Y. Yamada, T. Matsumoto and K. Matsubara, *Eur. J. Inorg. Chem.* **2011**, 2869-2878.

[111] S. P. Nolan and D. J. Nelson, *Chem. Soc. Rev.* **2013**, *42*, 6723-6753.

[112] F. Bru, R. S. C. Charman, L. Bourda, K. Van Hecke, L. Grimaud, D. J. Liptrot and C. S. J. Cazin, *Dalton Trans.* **2024**, *53*, 16030-16037.

[113] H. V. Huynh, *Chem. Lett.* **2021**, *50*, 1831–1841.

[114] G.-L. Chai, J.-W. Han and H. N. C. Wong, *Synthesis* **2017**, *49*, 181-187.

[115] E. G. Ijpeij, F. H. Beijer, H. J. Arts, C. Newton, J. G. de Vries and G.-J. M. Gruter, *J. Org. Chem.* **2002**, *67*, 169-176.

[116] J. H. Jang, S. Ahn, S. E. Park, S. Kim, H. R. Byon and J. M. Joo, *Org. Lett.* **2020**, *22*, 1280-1285.

[117] J. F. Hartwig in *Organotransition Metal Chemistry: From Bonding to Catalysis*, University Science Books **2010**, pp. 691-699.

[118] K. A. Spence, M. M. Mehta and N. K. Garg, *Org. Synth.* **2022**, *99*, 174-189.
